# Mapping the value for money of precision medicine: a systematic literature review and meta-analysis

**DOI:** 10.3389/fpubh.2023.1151504

**Published:** 2023-11-24

**Authors:** Wenjia Chen, Nigel Chong Boon Wong, Yi Wang, Yaroslava Zemlyanska, Dimple Butani, Suchin Virabhak, David Bruce Matchar, Thittaya Prapinvanich, Yot Teerawattananon

**Affiliations:** ^1^Saw Swee Hock School of Public Health, National University of Singapore, Singapore, Singapore; ^2^Health Intervention and Technology Assessment Program (HITAP), Ministry of Public Health, Bangkok, Thailand; ^3^Precision Health Research, Singapore (PRECISE), Singapore, Singapore; ^4^Duke-NUS Medical School, Singapore, Singapore; ^5^Yale-NUS College, Singapore, Singapore

**Keywords:** precision medicine, medical genetics, economic evaluation, value for money, systematic review, meta-analysis, cost effectiveness

## Abstract

**Objective:**

This study aimed to quantify heterogeneity in the value for money of precision medicine (PM) by application types across contexts and conditions and to quantify sources of heterogeneity to areas of particular promises or concerns as the field of PM moves forward.

**Methods:**

A systemic search was performed in Embase, Medline, EconLit, and CRD databases for studies published between 2011 and 2021 on cost-effectiveness analysis (CEA) of PM interventions. Based on a willingness-to-pay threshold of one-time GDP *per capita* of each study country, the net monetary benefit (NMB) of PM was pooled using random-effects meta-analyses. Sources of heterogeneity and study biases were examined using random-effects meta-regressions, jackknife sensitivity analysis, and the biases in economic studies checklist.

**Results:**

Among the 275 unique CEAs of PM, publicly sponsored studies found neither genetic testing nor gene therapy cost-effective in general, which was contradictory to studies funded by commercial entities and early stage evaluations. Evidence of PM being cost-effective was concentrated in a genetic test for screening, diagnosis, or as companion diagnostics (pooled NMBs, $48,152, $8,869, $5,693, *p* < 0.001), in the form of multigene panel testing (pooled NMBs = $31,026, *p* < 0.001), which only applied to a few disease areas such as cancer and high-income countries. Incremental effectiveness was an essential value driver for varied genetic tests but not gene therapy.

**Conclusion:**

Precision medicine’s value for money across application types and contexts was difficult to conclude from published studies, which might be subject to systematic bias. The conducting and reporting of CEA of PM should be locally based and standardized for meaningful comparisons.

## Introduction

Precision medicine (PM) is a novel medical approach that tailors intervention decisions based on expression profiling of individual phenotypes and genotypes or directly corrects pathogenic gene mutations (1, 2). The rapid evolvement of PM technology (3, 4) has led to global efforts of introducing PM into the existing healthcare settings to transform healthcare (5–8). However, the clinical adoption rate of PM remains low (9–13), and due to the lack of knowledge about PM’s value for money, the incentives among key stakeholders are poorly aligned to catalyze its development and adoption (9, 12, 14).

Cost-effectiveness analysis (CEA) provides a systemic framework to inform such decisions which, over a relevant time horizon of expected PM benefits and within the context of societal willingness-to-pay thresholds (WTP) for such benefits, assesses the cost of an intervention relative to the expected health gains in standard terms, such as quality-adjusted life year (QALY) (15). CEAs are commonly used to inform public and private sectors’ reimbursement decisions, clinical guidelines, benefit designs, and price negotiations (16) (“conventional CEA”), and help in decisions regarding product profile development and research priorities at an early clinical cycle (“early CEA”) (17). To guide research, practice, and policy related to PM, it is valuable to have a detailed understanding of the CEA literature, focusing on how a reported value is related to contexts and conditions of PM interventions, as well as specifications and potential biases of CEAs. Previous reports have described the general relationship between various characteristics of PMs studied and estimated cost-effectiveness (18, 19). However, previous reports have not formally assessed this literature using meta-analytic approaches.

This study aimed to quantify heterogeneity in the value for money of PM by pooling the net monetary benefits (NMBs) across the types of PM application [(1) screening for genetic conditions that predispose to disease, (2) early diagnosis, (3) prediction of disease progression, (4) companion diagnostic for targeting drug selection, and (5) gene therapy for established condition], as well as other contexts related to PM technology, disease domain, clinical stage, country capacity, and funder types. A secondary objective was to quantify sources of heterogeneity in PM’s value for money in the areas of particular promise or concern as the field of PM moves forward.

## Methods

The review was reported following the Preferred Reporting Items for Systematic Reviews and Meta-Analyses guidelines (20), and the protocol was recently published (PROSPERO: 2021 CRD42021272956) (21).

### Search strategy and study selection

We conducted the systematic search and study selection using the Covidence platform®. Embase, MEDLINE Ovid, EconLit, CRD, and Web of Science databases were searched to identify relevant studies published between January 1, 2011 and July 8, 2021, limited to studies published in or translated into English. In addition, we searched gray literature from reimbursement dossiers of several HTA agencies. Appendix 1 presents the details of the search strategies and search results for each database. To satisfy the inclusion criteria, the study had to be original research of cost-effectiveness pertaining to human subjects, reporting costs and either LYs, QALYs, disability-adjusted life years (DALYs), or incremental cost-effectiveness ratios (ICERs), and the intervention of interest had to conform to the working definition of PM (2). Selected studies with overlapped contents were excluded by five independent reviewers.

### Data extraction

Data were independently extracted by five reviewers which included characteristics of study (author’s name, publication year, geographic region, country-income level, type of funders, and conflict of interest), study population (target population, cascade testing, age, sex, disease areas, and associated prevalence and mortality rates), PM intervention (intervention type, profiling method, developmental stage, clinical pathways, test accuracy, uptake, and treatment compliance), comparators, outcomes (economic and effectiveness parameters, surrogate outcome, data source, and willingness-to-pay thresholds), and modeling (study perspective, time horizon, model type, discount rates, measures of dispersion, and uncertainty). Meanwhile, the risk of bias in the CEAs was assessed using the modified economic evaluations bias (ECOBIAS) checklist (22), which assesses sources of heterogeneity and bias in the overall structure and model of economic evaluations.

### Data harmonization and statistical analysis

The statistical analysis was performed using Stata MP version 17. The primary outcome was the net monetary benefit (NMB), which measures the difference between a monetized equivalent of incremental effectiveness (i.e., multiplied by a WTP threshold) and the incremental cost of new technology. Based on the central limit theorem, NBM is distributed normally and thus commonly used for quantitative analysis of CEAs (19, 23, 24). Although the standard practice typically uses nationally specific WTP thresholds, to enable global comparison that involves low- and middle-income countries (LMICs), we followed the recommendation of the World Health Organization (WHO) and World Bank (25) that defined the WTP threshold as the one-time national gross domestic product (GDP) *per capita* as of the study year. To standardize costing data, all NMBs were first inflated to the 2020 currency of that study country and then converted to 2020 USD ($) according to the consumer price index and exchange rate from the World Bank (26).

Following the latest guideline for data harmonization in meta-analyses of CEAs (27), we prepared NMB data, with details and the published protocol described in Appendix 2 (21). Through data harmonization, the NMB and its variance were consistently calculated by comparing PM to a conventional intervention strategy. Based on the COMER methodology (28), we performed a random-effects meta-analysis to calculate weighted-pooled summary estimates of NMB using the DerSimonian and Laird (DL) method (29).


$$\mathrm{Pooled}\;NMB_{r}=\frac{\sum_{i=1}^{N}w_{i}NMB_{i}}{\sum_{i=1}^{N}w_{i}+\gamma^{2}}\text{,}$$

where ɤ^2^ $$=\frac{Q-\left(N-1\right)}{\sum_{i=1}^{N}w_{i}-\frac{\sum_{i=1}^{N}{w_{i}}^{2}}{\sum_{i=1}^{N}w_{i\text{.}}}}$$

where $w_{i}$ refers to the inverse of variance. Heterogeneity was tested using the Cochran Q test and *I*(2) statistics (30), with *I*(2) = 25–74% indicating moderate heterogeneity and *I*(2) ≥ 75% indicating high heterogeneity.

Subgroup analyses were performed in ≥two datapoints to investigate the context-specific value for money of PM. We estimated the weighted-pooled NMB by subgroups, namely, PM applications, technology [single-gene profiling, multigene panel, whole-genome sequencing (GS), and whole-exome sequencing (ES)], clinical stage (first-clinical-use vs. market access), 16 major disease areas defined by International Disease Classification diagnosis codes, version 10 (ICD-10) (31), WHO region (32), World Bank country-income level (*per capita* Gross National Income in 2020 USD when most information was available) (33), and funder type (public vs. non-profit private, for-profit private, and mixed or unspecified funding sources).

To assess the robustness and conclusiveness of pooled NMB findings, the jackknife sensitivity analysis was performed for each abovementioned subgroup, which omitted one study at a time and repeated the meta-analysis in the rest of the studies (34). This examined whether pooled NMB was consistent across the studies or excessively affected by any influential CEAs.

Following expert recommendation, publication bias was assessed using funnel plots and Egger’s test (27). A funnel plot put NMB estimates on the *x*-axis against the quantified uncertainty interval on the *y*-axis. Egger’s test assessed whether the funnel was symmetrical, or there was heterogeneity and/or missing studies.

To identify and quantify sources of heterogeneity in the pooled NMB of each PM type, first, we ranked the frequency of the most sensitive parameters to ICER that were reported in the sensitivity analyses of CEAs. Second, we performed univariate random-effects meta-regressions to examine the impact of 19 influencing factors that explain NMB heterogeneity due to study year, target population (age, sex, disease incidence rate, and use of cascade testing), and intervention characteristics (PM cost, incremental effectiveness, integrations of test uptake, test accuracy, and treatment compliance) and that explain value bias as a function of methods (study perspective, time horizon, model type, respective sources of cost and effectiveness data, any use of surrogate outcome, % of “yes” answers in overall ECOBIAS assessment, % “yes” answers in model-specific ECOBIAS assessment, and any conflict of interest). Third, because many covariates were found to be associated with NMB in the univariate meta-regressions (*p* < 0·05), we used a generalized Lasso approach with 10-fold cross validation to select essential covariates to be included in the multivariate meta-regression (the best-fitting model) (35). Finally, essential covariates were included in a multivariate, random-effect meta-regression (35) to quantify the impact of essential value drivers on the NMB of each PM type. Of note, we compared three random-effect meta-regression models, namely REML, DL, and empirical Bayes, and selected the model that yielded the greatest reduction in between-study heterogeneity [*τ*(2)] of NMBs.

## Results

### Literature search and study characteristic

**Figure 1:**
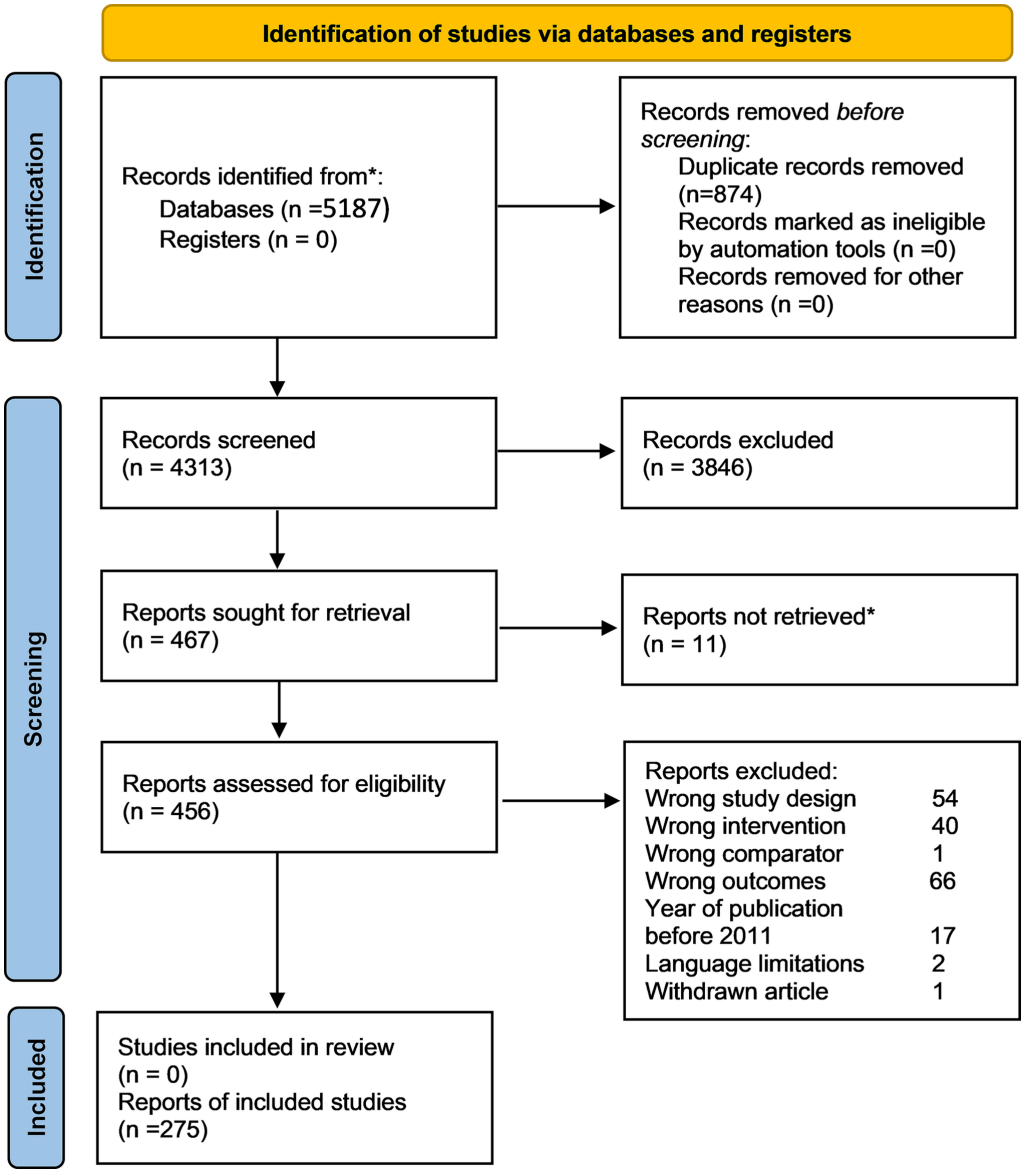
PRISMA 2020 flow diagram of systematic search and selection. Numbers refer to unique study records, not datasets, except where otherwise indicated.

The literature search initially identified 5,187 articles. The final analysis included 275 unique CEAs with 463 cost-effectiveness estimates of varied PM applications because one CEA may include multiple test-treatment strategies, comparators, and settings (Figure 1, Flowchart of literature search and selection; Appendix 3, Full list of included studies).

Table 1 presents the study characteristics. Appendix 4 provides more details. Among the 238 CEAs on genetic testing and 37 CEAs on gene therapy, most were performed in high-income countries, in Western countries and applied to cancer. The median unit cost ranged between $220 and $3,091 for genetic tests and was $321,268 for gene therapy. The median ∆QALY was the lowest in the prognostic test compared to other test types (0.07 vs. 0.23–0.73) and the highest (3.83) in gene therapy. The pattern of risk of bias was persistent across varied PM application types, mainly focusing on narrow perspective, cost measurement omission, intermittent data collection, double counting, limited sensitivity analysis, and limited scope (Appendix 5).

**Table 1:** General and economic characteristics of cost-effectiveness analyses reporting precision medicine interventions.

Characteristic	Screening test (*N* = 52)	Diagnostic test (*N* = 27)	Prognostic test (*N* = 53)	Companion test (*N* = 106)	Gene therapy (*N* = 37)
PM Unit Cost, Median (IQR)	385 (147–1,204)	1,059 (424–3,696)	3,091 (754–3,750)	220 (108–439)	321,268 (4,051–607,118)
WHO region, *n* (%)					
African Region (AFR)	0 (0%)	0 (0%)	0 (0%)	1 (0.9%)	0 (0%)
Region of the Americas (AMR)	24 (46%)	15 (56%)	26 (49%)	46 (43%)	21 (57%)
South-East Asian Region (SEAR)	0 (0%)	0 (0%)	0 (0%)	10 (9.4%)	1 (2.7%)
European Region (EUR)	17 (33%)	10 (37%)	22 (42%)	23 (22%)	7 (19%)
Eastern Mediterranean Region (EMR)	1 (1.9%)	0 (0%)	0 (0%)	0 (0%)	1 (2.7%)
Western Pacific Region (WPR)	10 (19%)	2 (7.4%)	5 (9.4%)	26 (25%)	7 (19%)
Study perspective, *n* (%)					
Societal	8 (15%)	5 (19%)	7 (13%)	21 (20%)	5 (14%)
Healthcare	41 (79%)	19 (70%)	42 (79%)	79 (75%)	30 (81%)
Other (e.g., patient perspective)	3 (5.8%)	3 (11%)	4 (7.5%)	6 (5.7%)	2 (5.4%)
Effectiveness outcomes, *n* (%)					
QALYs	47 (90%)	26 (96%)	50 (94%)	104 (98%)	37 (100%)
Life years	5 (9.6%)	1 (3.7%)	3 (5.7%)	2 (1.9%)	0 (0%)
CEA type by PM stage, *n* (%)					
Early CEA to guide R&D	12 (23%)	4 (15%)	12 (23%)	31 (29%)	10 (27%)
Conventional CEA to inform reimbursement	40 (77%)	23 (85%)	41 (77%)	75 (71%)	27 (73%)
Time horizon, *n* (%)					
Short term (0 < T ≤ 3 years)	1 (1.9%)	3 (11%)	1 (1.9%)	25 (24%)	2 (5.4%)
Intermediate (3 < T ≤ 10 years)	4 (7.7%)	3 (11%)	16 (30%)	19 (18%)	6 (16%)
Long term (10 < T ≤ 30 years)	3 (5.8%)	5 (19%)	6 (11%)	11 (10%)	4 (11%)
Lifetime (T > 30 years)	43 (83%)	16 (59%)	29 (55%)	49 (46%)	24 (65%)
Not reported	1 (1.9%)	0 (0%)	1 (1.9%)	2 (1.9%)	1 (2.7%)
Conclusion, *n* (%)					
Cost-effective/cost-saving	39 (75%)	21 (78%)	36 (68%)	66 (62%)	23 (62%)
Not cost-effective	10 (19%)	5 (19%)	11 (21%)	31 (29%)	10 (27%)
Inconclusive	3 (5.8%)	1 (3.7%)	6 (11%)	9 (8.5%)	4 (11%)

CEA, Cost-effectiveness analysis; IQR, Inter-quartile range; LY, Life year; QALY, Quality-adjusted life year; NA, Not applicable; QALY, Quality-adjusted life year.

### Context-level variations in PM’s value for money

#### Genetic testing

**Figure 2:**
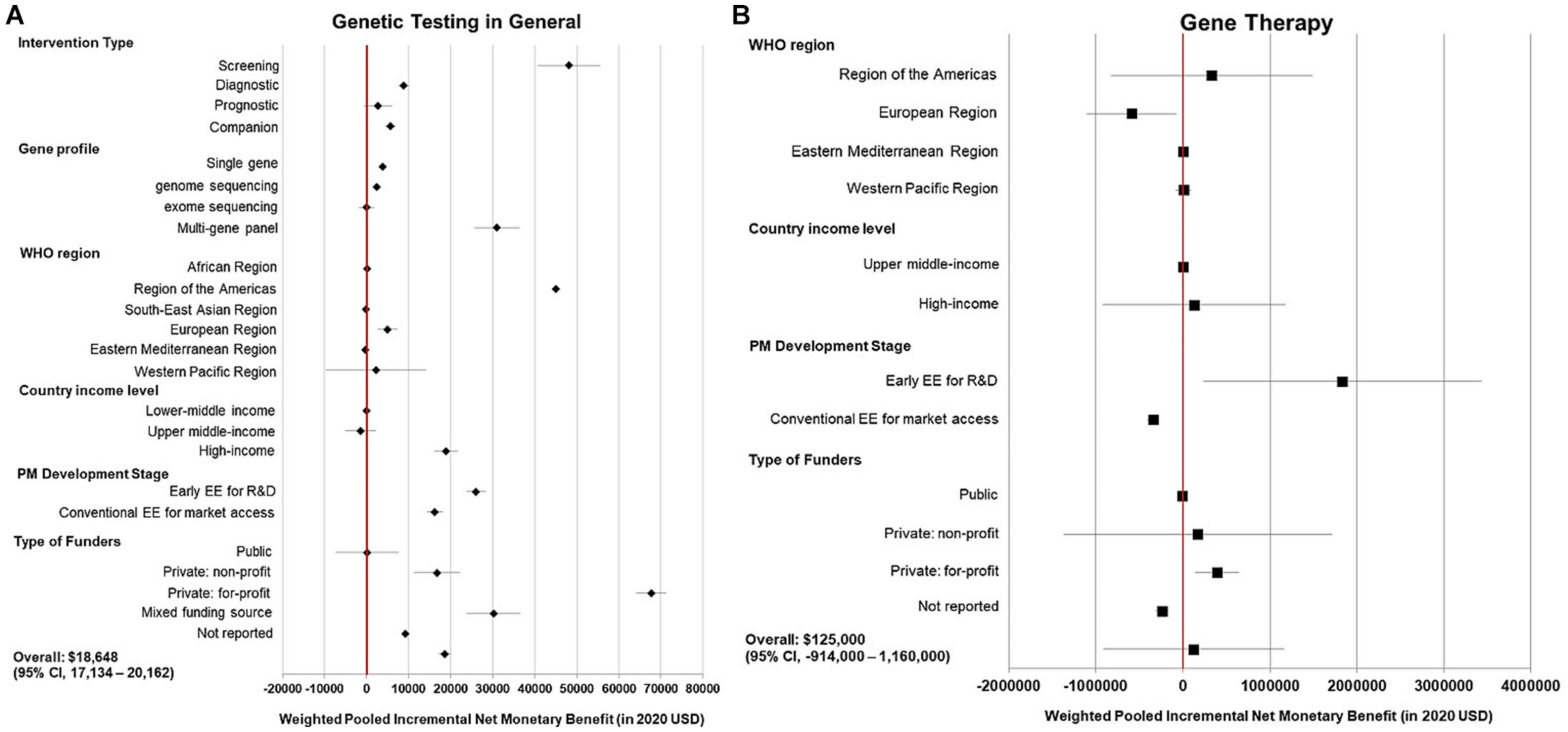
Summary forest plot showing the weighted-pooled summary estimates of incremental net monetary benefit of precision medicine. **(A)** Left panel, genetic testing in general; **(B)** Right panel, gene therapy. The error bars show the 95% confidence interval. The red vertical line marks the border for significance.

High heterogeneity was detected from the meta-analysis of 369 cost-effectiveness estimates (*I*(2) = 100%). By clinical applications, pooled NMBs descended from genetic tests use for screening ($48,152 [95% CI 40,725–55,579]), diagnosis ($8,869 [7,570—10,168]), companion diagnostic for targeted therapy ($5,693 [4,548—6,839]), and to not being significantly greater than 0 for prognostic tests ($2,694 [−601 to 5,988], *p* = 0.11). By profiling technology, multigene panel testing had higher pooled NMB than single gene testing and GS ($31,026 [25,602–36,449] vs. $3,893[3,058–4,727] and $2,429[1,886–2,972], respectively), whereas the pooled NMB of ES was not significantly positive (*p* = 1.00; Figure 2A).

**Figure 3:**
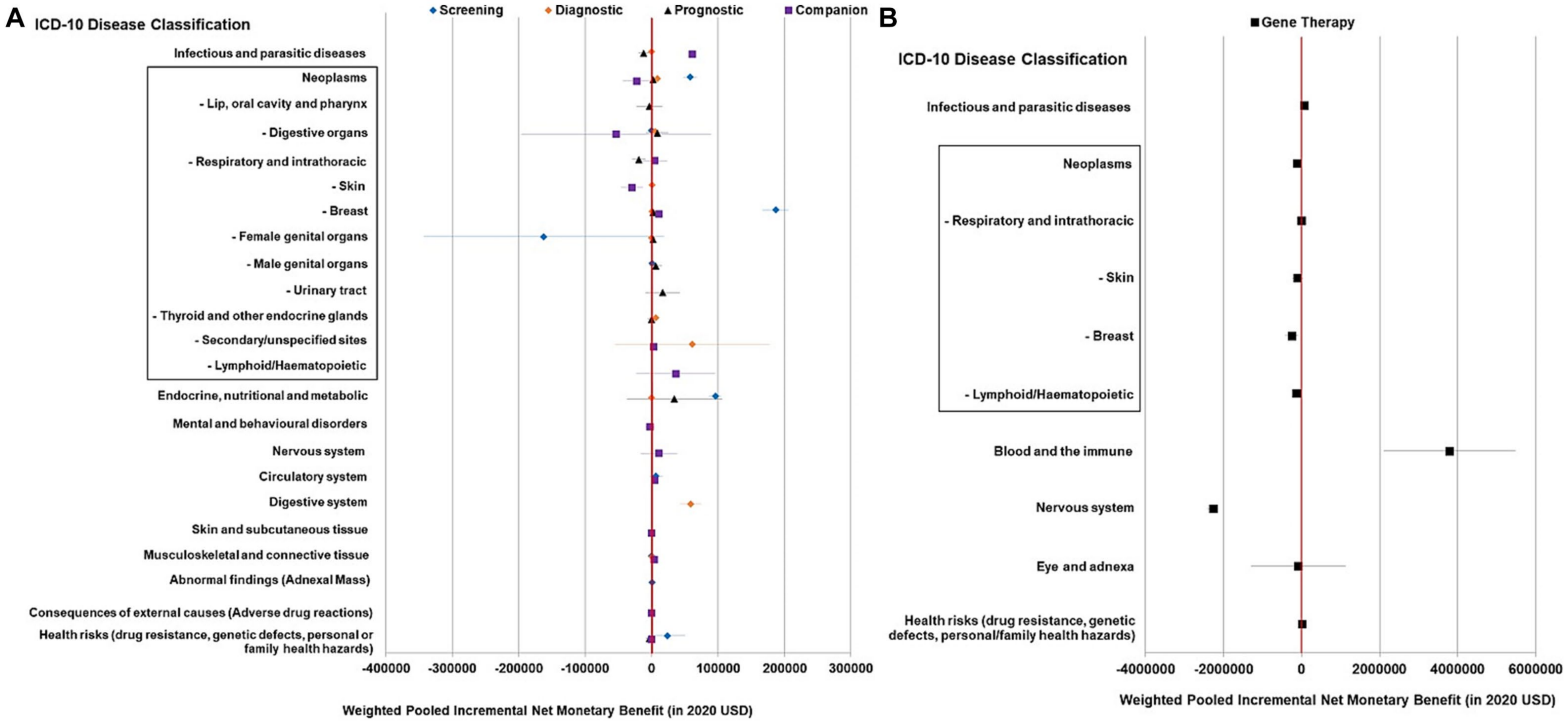
Summary forest plot showing the weighted-pooled summary estimates (in ≥ two datapoints) of incremental net monetary benefit of precision medicine across major ICD disease domains. **(A)** Genetic testing for different purposes; **(B)** Gene therapy. The error bars show the 95% confidence interval. The box shows neoplasm/cancer and detailed sub-categories. The red vertical line marks the border for significance.

Within each test type, only certain disease areas showed evidence of cost-effectiveness in general. Genetic tests had positive pooled NMBs when used for screening in endocrine and metabolic diseases (especially familial hypercholesterolemia) and cancer, in particular breast cancer ($96,018, $57,889, and $187,000, respectively), for diagnosis in Barrett’s esophagus (a pre-malignant digestive condition) and cancer, most commonly thyroid cancer ($58,975, $8,422, and $6,051, respectively), and as a companion diagnostic in chronic infectious diseases (chronic hepatitis C, HIV), gout, and rheumatoid arthritis ($61,333, $4,850, and $4,173, respectively). Nonetheless, the pooled NMBs of the prognostic test were not statistically positive in varied disease areas (Figure 3A).

#### Gene therapy

In the meta-analysis of 56 cost-effectiveness estimates, the pooled NMB of gene therapy was not significantly greater than 0 in a variety of contexts (Figures 2B, 3B).

### System-level variations in PM’s value for money

At the structural level, for both genetic tests and gene therapy, commercially funded studies yielded high pooled NMBs, whereas publicly sponsored studies found no evidence of PM being cost-effective in general (Figures 2A,B). Early CEAs also reported a higher pooled NMB compared to conventional CEAs both in genetic tests ($26,009 vs. $16,215; Figure 2A) and gene therapy ($1,830,000 vs. $0 [insignificant value]; Figure 2B).

At the country level, genetic tests had positive pooled NMBs in studies from America ($44,972), Europe ($5,005), and high-income countries ($18,930), whereas an inconclusive value in Western Pacific (*p* = 0.72) and middle-income countries (*p* = 0·87 and 0·44). Gene therapy had a negative pooled NMB in studies in Europe (−$588,000) and an inconclusive value in the Americas, Eastern Mediterranean (Qatar), and Western Pacific (*p* = 0.57, 0.60, and 0.88, respectively).

### Consistency, robustness, and publication bias of PM’s value for money

In the jackknife sensitivity analysis, cost-effectiveness findings were valid and consistent in the above-described subgroups, i.e., both the pooled NMB and the corresponding 95% CI remained in the original position and direction regardless of the omission of any single datapoint (Appendix 6).

As seen by the asymmetry on the funnel plots (Appendix 7), publication bias was present in pooled NMBs of genetic tests in general (Egger’s test, coefficient = −0.75, SE = 0.10, *p* < 0.001), particularly in screening, diagnosis, and companion diagnostics (Egger’s test, all value of *p*s < 0.05), whereas there was no evidence of publication bias in pooled NMBs of prognostic tests and gene therapy (Egger’s test, *p* = 0.296 and 0.608, respectively).

### Sources of heterogeneity in PM’s value for money

The ICERs of varied genetic tests but not gene therapy were highly sensitive to disease progression rate and test cost; the ICERs of diagnostic, prognostic, companion tests, and gene therapy but not screening tests were highly sensitive to treatment cost and effectiveness; and the ICERs of screening and diagnostic tests but not prognostic or companion tests were highly sensitive to test accuracy (Appendix 9).

In the univariate meta-regressions of NMBs of genetic tests (Appendix 8), 18 out of 19 selected covariates were significantly associated with NMBs of studies of each test type. Multivariate meta-regression results based on Lasso-selected essential features are presented in Table 2. Overall, 97.2% of variability [i.e., *R*(2)] in screening tests’ NMBs was explained by incremental effectiveness (*p* < 0.001) and target age (*p* = 0.32), 95.9% of variability in the diagnostic tests’ NMBs was explained by incremental effectiveness, target sex, source of cost data, model type, and overall study bias (all had *p* < 0.001), and 48.5% of the variability in the prognostic tests’ NMBs was explained by incremental effectiveness (*p* < 0.001), target sex (*p* = 0.07), study perspective (*p* < 0.001), test accuracy (*p* = 0.26), treatment compliance (*p* = 0.001), and publication year (*p* = 0.33), whereas the only essential predictor of the companion tests’ NMBs was incremental effectiveness (*p* < 0.001) when treatment cost was absent, explaining 11.8% of the variability. Test cost was not identified as an essential value driver for any genetic test type.

**Table 2:** Parameter estimates from multivariate meta-regression model on the net monetary benefit of genetic testing.

	Screening		Diagnostic		Prognostic		Companion	
Essential risk factors	coefficient (95% CI)	*p* value	coefficient (95% CI)	*p* value	coefficient (95% CI)	*p* value	coefficient (95% CI)	*p* value
Incremental QALY/LY	60,181 (59,752, 60,609)	**<0·001** ^*^	41,943 (40,381, 43,504)	**<0·001**	24,515 (20,581, 28,450)	**<0·001**	27,375 (26,496, 28,255)	**<0·001**
Year of publication					−490 (−1,477, 496)	0·33		
Target age		0·317						
Adult	(Reference)							
Pediatric	−2,898 (−9,329, 3,533)	0·377						
All ages/not specified	−2,931 (−7,286, 1,424)	0·187						
Target sex				**<0·001**		0·068		
Mixed-sex			(Reference)		(Reference)			
All-male			−239,149 (−1,188,713, 710,415)	0·622	10,650 (−8, 21,308)	**0·05**		
All-female			4,161 (2,371, 5,951)	**<0·001**	6,004 (−326, 12,334)	0·063		
Perspective adopted						**0·0002**		
Social					(Reference)			
Healthcare					−15,259 (−22,434, −8,084)	**<0·001**		
Other (e.g., patient perspective)					−14,298 (−25,778, −2,819)	**0·015**		
Type of analysis used for model				**<0·001**				
Decision tree model			(Reference)					
Markov model			−16,034 (−18,868, −13,200)	**<0·001**				
Hybrid model (Decision tree + Markov)			−23,880 (−26,425, −21,335)	**<0·001**				
Discrete event simulation			−18,942 (−22,109, −15,775)	**<0·001**				
Test accuracy integrated					4,877 (−3,527, 13,281)	0·255		
Treatment compliance integrated					14,970 (6,150, 23,789)	**0.001**		
Source of cost data				**<0.001**				
Primary data collected			(Reference)					
Other studies (same setting)			−21,507 (−24,773, −18,241)	**<0.001**				
Secondary sources (same setting)			−2,188 (−4,923, 548)	0.117				
Other studies (other settings)			4,380 (1,607, 7,154)	**0.002**				
% Yes among all ECOBIAS variables, per 1% increase			699 (580, 818)	**<0.001**				

^*^Bold characters represent a significant effect of a predictor at an alpha level of 0.05. CI, Confidence interval; QALY, Quality-adjusted life year; LY, Life year. Shaded areas indicate that the variable was not essential in predicting the NMB of the corresponding genetic test type.

In particular, one extra unit of incremental effectiveness was associated with a marginal increase in NMB of $60,181 (95% CI 59,752–60,609) for the screening test, $41,943 (95% CI 40,381–43,504) for the diagnostic test, $24,515 (95% CI 20,581–28,450) for the prognostic test, and $27,375 (95% CI 26,496–28,255) for the companion diagnostic test.

In the univariate meta-regressions of gene therapy, treatment cost, study perspective, and target patient sex were significant value drivers (*p* < 0.001 for all), but incremental effectiveness barely explained any NMB variability [*R*(2) = 0%, *p* = 0.79; Appendix 8].

## Discussion

In this systematic review and meta-analysis of 275 CEAs on PM published during 2011–2021, the value for money of genetic tests was highly context-specific: While genetic tests appeared cost-effective for screening, diagnosis, or companion diagnosis, such evidence was mainly based on established profiling methods and treatments, well-studied disease indications, and from high-income countries. Evidence in new technologies (e.g., ES and gene therapy) and LMICs remained scarce and inconclusive. Incremental effectiveness and target population but not test cost were the essential drivers of value for money of varied genetic tests. Importantly, studies funded by public agencies generally found NMBs of PM to be not significantly greater than 0, whereas commercially funded and/or early stage studies consistently support PM as cost-effective.

Our findings were generally in line with previous literature. Kasztura et al. (18) reviewed 83 economics studies (2014–2017) on PM and concluded that most previous reviews found inconclusive evidence regarding PM’s cost-effectiveness. Vellekoop et al. (19) explored heterogeneity in NMBs of 128 CEAs (2009–2019) on PM. The medians of ∆QALY, ∆cost, and NMB of our study were comparable to results of Vellekoop et al. (19) (0.05 vs. 0.03, $445 vs. $575, and $135 vs. $18, respectively). The study by Vellekoop et al. (19) found gene therapies barely cost-effective in general whereas industry sponsorship was positively associated with cost-effectiveness, and our results confirmed both. As an update and extension, we quantified sources of heterogeneity in PM’s value for money on an extensive collection of covariates which, for the first time, enabled in-depth investigation into heterogeneity by application type across disease areas, technologies, clinical stages, as well as sources of heterogeneity related to intervention characteristics, model specifications, and study biases.

Across clinical applications, genetic tests reported differential value for money. Of note, test cost was similar across genetic test types and had no major influence on their NMBs. However, one unit increase in ∆QALY would lead to 2–3 times higher ∆NMB if use for screening and diagnosis compared to prognosis or companion diagnostics, which indicated that PM-enabled early intervention (through risk detection or early diagnosis) was more efficient than PM-enabled treatment stratification (by predicted clinical risk or treatment response) in controlling the costs of disease management in general. In support of this, only screening and diagnostic tests appeared as cost-effective in cancer in published studies, whereas prognostic and companion tests were as plentiful in number but appeared to not be cost-effective. In particular, the prognostic test typically stratifies severe subgroups to advanced treatment which may be still patented and costly, rendering in not-cost-effective profiles in general. Furthermore, the cost-effectiveness of the same type of genetic test varied across disease areas probably because it was largely dependent on incremental effectiveness, which can explain the substantial value difference of genetic screening in breast cancer (a genetic test was used for primary screening) vs. cervical cancer (a genetic test was used as an add-on to pap smear screening). Nonetheless, what is subject to change is PM’s inconclusive cost-effectiveness profiles in new innovations, new disease indications, and new markets. Over time, the costs of new PM innovation (in particular gene therapy which on average costs $321,268 per patient) can reduce substantially when the scale and scope of production increases, and evidence in new indications and new markets can accumulate. These may render currently not-cost-effective PM interventions to become good value for money in the future.

Our study revealed significant systematic biases. The substantial discrepancies in PM’s value for money between early and conventional CEAs, and between commercially funded and publicly sponsored CEAs, can be related to study manipulation as a result of overambition or over-optimism from the R&D community and commercial entities, especially at an early stage when best guesses were commonly adopted, or publication bias such that positive results were more likely to be submitted for publication. For instance, study perspective was found to be an essential value driver of the prognostic genetic test and gene therapy but not of genetic tests used for screening, diagnosis, or companion diagnostics. This pattern could indicate a greater share of analyses leveraging societal perspectives for interventions that were relatively less cost-effective from a healthcare system’s perspective. Therefore, our study supports the call from a recent perspective in Nature Reviews (36) that a reference case should be developed to standardize the evaluation and report on the economic impact of PM. For this reason, we are conducting an in-depth analysis of methodological variations that can lead to the development of a reference case for PM evaluation. The results will be published in a separate study.

This study has several limitations. First, it was impossible to capture all sources of heterogeneity due to systemic differences in health service utilization across settings. Second, we excluded non-English publications. Third, using the same WTP for LYs as for QALYs or DALYs may be inappropriate, but over 96% of included studies measured QALYs. Fourth, we were unable to extract treatment cost from the complex, stratified, and/or changing treatment regimens in many studies. Last but not least, the cost-effectiveness findings did not apply to LMICs because no data were available from low-income countries and the studies from middle-income countries provided no support for the cost-effectiveness of PM in general.

To conclude, a large body of evidence suggests that the value for money of PM applications is concentrated in established technologies, disease domains, and markets, which is mainly influenced by incremental effectiveness in favor of early intervention over treatment stratification at diseased stages. It takes time for PM in new innovations, new indications, and new markets to accumulate evidence to affirm its value of money. Moreover, current CEAs of PM are prone to study manipulation and systematic bias. Thus, it is difficult to make an overall conclusion on PM’s value for money across application types and disease areas. To enable meaningful comparisons for truly informed decision-making, policymakers and stakeholders should conduct local studies, with appropriate consensus approaches to standardize the conducting and reporting of CEA of PM.

## Data availability statement

The data analyzed in this study is subject to the following licenses/restrictions: the data are currently available upon request. The data will be later deposited in a central depository in National University of Singapore, Saw Swee Hock School of Public Health, for public access. Requests to access these datasets should be directed to wenjiach@nus.edu.sg.

## Author contributions

WC and YT conceptualized the study and identified the research question and method of this study. NW, YW, YZ, DB, and TP performed literature search and data extraction. WC and NW designed the data analysis plan and completed the analysis. SV and DM provided critical policy feedback and supported the design, method, and search strategy of study. WC, YW, SV, DM, and YT supervised the entire research process with support reviewing the search strategy and the research questions. WC and YZ wrote the first draft of the manuscript. All authors contributed to the article and approved the submitted version.

## References

[1] Schleidgen S Klingler C Bertram T Rogowski WH Marckmann G. What is personalized medicine: sharpening a vague term based on a systematic literature review. BMC Med Ethics. (2013) 14:55. doi: 10.1186/1472-6939-14-55, PMID: 24359531 PMC3878093

[2] Vellekoop H Huygens S Versteegh M Szilberhorn L Zelei T Nagy B . Guidance for the harmonisation and improvement of economic evaluations of personalised medicine. PharmacoEconomics. (2021) 39:771–88. doi: 10.1007/s40273-021-01010-z, PMID: 33860928 PMC8200346

[3] EMBL-EBI (2022). What is Next Generation DNA Sequencing? | Functional genomics II. Available at: https://www.ebi.ac.uk/training/online/courses/functional-genomics-ii-common-technologies-and-data-analysis-methods/next-generation-sequencing/ (Accessed March 15, 2022).

[4] YAN SK LIU RH JIN HZ LIU XR YE J SHAN L . “Omics” in pharmaceutical research: overview, applications, challenges, and future perspectives. Chin J Nat Med. (2015) 13:3–21. doi: 10.1016/S1875-5364(15)60002-4, PMID: 25660284

[5] Precision Health Research, Singapore (PRECISE) (2021). Available at: https://www.npm.sg/ (Accessed August 10, 2021).

[6] Thailand Pharmacogenomics Network (2021). Available at: http://www.thailandpg.org/ (Accessed August 10, 2021).

[7] Ginsburg GS Phillips KA. Precision medicine: from science to value. Health Aff. (2018) 37:694–701. doi: 10.1377/hlthaff.2017.1624, PMID: 29733705 PMC5989714

[8] NCBI (2021). Genetic Testing Registry (GTR). Available at: https://www.ncbi.nlm.nih.gov/gtr/ (Accessed August 10, 2021).

[9] Davis JC Furstenthal L Desai AA Norris T Sutaria S Fleming E . The microeconomics of personalized medicine: today’s challenge and tomorrow’s promise. Nat Rev Drug Discov. (2009) 8:279–86. doi: 10.1038/nrd2825, PMID: 19300459

[10] Leapman MS Wang R Ma S Gross CP Ma X. Regional adoption of commercial gene expression testing for prostate Cancer. JAMA Oncol. (2021) 7:52–8. doi: 10.1001/jamaoncol.2020.6086, PMID: 33237277 PMC7689565

[11] Elverum K Whitman M. Delivering cellular and gene therapies to patients: solutions for realizing the potential of the next generation of medicine. Gene Ther. (2020) 27:537–44. doi: 10.1038/s41434-019-0074-7, PMID: 31024072 PMC7744278

[12] Virelli CR Mohiuddin AG Kennedy JL. Barriers to clinical adoption of pharmacogenomic testing in psychiatry: a critical analysis. Transl Psychiatry. (2021) 11:509. doi: 10.1038/s41398-021-01600-7, PMID: 34615849 PMC8492820

[13] Mrazek DA. Psychiatric pharmacogenomic testing in clinical practice. Dialogues Clin Neurosci. (2010) 12:69–76. doi: 10.31887/DCNS.2010.12.1/dmrazek, PMID: 20373668 PMC3181940

[14] Messner DA Koay P Al Naber J Cook-Deegan R Majumder M Javitt G . Barriers to clinical adoption of next-generation sequencing: a policy Delphi panel’s solutions. Perinat Med. (2017) 14:339–54. doi: 10.2217/pme-2016-0104, PMID: 29230253 PMC5722256

[15] Garber AM Sculpher MJ. Chapter eight—cost effectiveness and payment policy In: Pauly MV Mcguire TG Barros PP, editors. Handbook of Health Economics, vol. 2: North Holland: Elsevier. 471–97.

[16] Pietzsch JB Paté-Cornell ME. Early technology assessment of new medical devices. Int J Technol Assess Health Care. (2008) 24:36–44. doi: 10.1017/S0266462307080051 18218167

[17] IJzerman MJ Koffijberg H Fenwick E Krahn M. Emerging use of early health technology assessment in medical product development: a scoping review of the literature. PharmacoEconomics. (2017) 35:727–40. doi: 10.1007/s40273-017-0509-1, PMID: 28432642 PMC5488152

[18] Kasztura M Richard A Bempong NE Loncar D Flahault A. Cost-effectiveness of precision medicine: a scoping review. Int J Public Health. (2019) 64:1261–71. doi: 10.1007/s00038-019-01298-x, PMID: 31650223 PMC6867980

[19] Vellekoop H Versteegh M Huygens S Corro Ramos I Szilberhorn L Zelei T . The net benefit of personalized medicine: a systematic literature review and regression analysis. Value Health. (2022) 25:1428–38. doi: 10.1016/j.jval.2022.01.006, PMID: 35248467

[20] Shamseer L Moher D Clarke M Ghersi D Liberati A Petticrew M . Preferred reporting items for systematic review and meta-analysis protocols (PRISMA-P) 2015: elaboration and explanation. BMJ. (2015) 349:g7647. doi: 10.1136/bmj.g7647, PMID: 25555855

[21] Chen W Anothaisintawee T Butani D Wang Y Zemlyanska Y Wong CBN . Assessing the cost-effectiveness of precision medicine: protocol for a systematic review and meta-analysis. BMJ Open. (2022) 12:e057537. doi: 10.1136/bmjopen-2021-057537, PMID: 35383079 PMC8984003

[22] Adarkwah CC van Gils PF Hiligsmann M Evers SMAA. Risk of bias in model-based economic evaluations: the ECOBIAS checklist. Expert Rev Pharmacoecon Outcomes Res. (2016) 16:513–23. doi: 10.1586/14737167.2015.1103185, PMID: 26588001

[23] Haider S Chaikledkaew U Thavorncharoensap M Youngkong S Islam MA Thakkinstian A. Systematic review and Meta-analysis of cost-effectiveness of rotavirus vaccine in low-income and lower-midle-income countries. Open Forum Infect Dis. (2019) 6:ofz117. doi: 10.1093/ofid/ofz117, PMID: 31049363 PMC6488528

[24] Bagepally BS Gurav YK Anothaisintawee T Youngkong S Chaikledkaew U Thakkinstian A. Cost utility of sodium-glucose cotransporter 2 inhibitors in the treatment of metformin monotherapy failed type 2 diabetes patients: a systematic review and Meta-analysis. Value Health. (2019) 22:1458–69. doi: 10.1016/j.jval.2019.09.2750, PMID: 31806203

[25] Robinson LA Hammitt JK Chang AY Resch S. Understanding and improving the one and three times GDP per capita cost-effectiveness thresholds. Health Policy Plan. (2017) 32:141–5. doi: 10.1093/heapol/czw096, PMID: 27452949

[26] World Bank (2021). Inflation, consumer prices (annual %)|Data. Available at: https://data.worldbank.org/indicator/FP.CPI.TOTL.ZG (Accessed August 3, 2021)

[27] Bagepally BS Chaikledkaew U Chaiyakunapruk N Attia J Thakkinstian A. Meta-analysis of economic evaluation studies: data harmonisation and methodological issues. BMC Health Serv Res. (2022) 22:202. doi: 10.1186/s12913-022-07595-1, PMID: 35168619 PMC8845252

[28] Crespo C Monleon A Díaz W Ríos M. Comparative efficiency research (COMER): meta-analysis of cost-effectiveness studies. BMC Med Res Methodol. (2014) 14:139. doi: 10.1186/1471-2288-14-139, PMID: 25533141 PMC4292992

[29] DerSimonian R Laird N. Meta-analysis in clinical trials. Control Clin Trials. (1986) 7:177–88. doi: 10.1016/0197-2456(86)90046-2 3802833

[30] West SL Gartlehner G Mansfield AJ Poole C Tant E Lenfestey N . (2021). Table 7, Summary of common statistical approaches to test for heterogeneity. Published September 2010. Available at: http://www.ncbi.nlm.nih.gov/books/NBK53317/table/ch3.t2/ (Accessed August 3, 2021).

[31] World Health Organization (2021). International Classification of Diseases (ICD). Available at: https://www.who.int/standards/classifications/classification-of-diseases (Accessed August 10, 2021).

[32] World Health Organization (2021). WHO country-region. Available at: https://www.who.int/countries (Accessed August 10, 2021).

[33] World Bank (2021). World Bank Country and Lending Groups. Available at: https://datahelpdesk.worldbank.org/knowledgebase/articles/906519-world-bank-country-and-lending-groups (Accessed August 10, 2021).

[34] Miller RG. The jackknife-a review. Biometrika. (1974) 61:1–15. doi: 10.1093/biomet/61.1.1

[35] Ghosh D Zhu Y Coffman DL. Penalized regression procedures for variable selection in the potential outcomes framework. Stat Med. (2015) 34:1645–58. doi: 10.1002/sim.6433, PMID: 25628185 PMC4390482

[36] Payne K Gavan SP Wright SJ Thompson AJ. Cost-effectiveness analyses of genetic and genomic diagnostic tests. Nat Rev Genet. (2018) 19:235–46. doi: 10.1038/nrg.2017.108, PMID: 29353875

