# Supplementary material for: Mapping the value for money of precision medicine: a systematic literature review and meta-analysis
Source: Front Public Health. 2023 Nov 24;11:1151504. doi: 10.3389/fpubh.2023.1151504 (PMC10704154; doi:10.3389/fpubh.2023.1151504)
Supplement: Supplementary file 1 [file Table_1.DOCX]

**Appendix 4. Summary of the general and economic characteristics of current CEAs on PM**

- Intervention:
  - Among these CEAs, 18·9% were focused on screening tests for disease risk (n=52), 9·8% on diagnostic tests (n=27), 19·3% on prognostic tests (n=53), 38·5% on companion tests of drug response or adverse drug reactions (n=106), both for targeted treatment, and 13·5% on gene therapy (n=37).
  - Within genetic testing, most were single gene (n=90, 37·8%) or multigene panel (n=122, 51·3%) profiling, 9·2% (n=22) were WGS, and 1·7% were WES (n=4).
- Patient population:
  - Most CEAs were performed in high income countries (89%), in particular in the Americas (48%), Europe (29%) and Western Pacific regions (18%). Companion tests were the most common in Southeast Asian (n=10) and African (n=1) studies.
  - Screening, diagnostic, prognostic tests and gene therapy mainly targeted cancer patients (54-87%), whereas companion tests have a wider application across disease domains (e.g., cancer, cardiovascular, infectious/parasitic, mental etc).
- Comparator: >90% of CEAs compared PM across varied application purposes with standard of care.
- Time: CEAs on screening tests, diagnostics and gene therapy mainly projected long-term to lifetime outcomes (>3/4), whereas a considerable proportion of CEAs on treatment-targeting tests (prognostic and companion) more often projected short-to intermediate-term outcomes in comparison (>30%).
- Outcome:
  - Median unit cost was highest in gene therapy ($321,268, IQR: 4,051– 607,118), followed by prognostic ($3,091, IQR: 754–3,750), diagnostic ($1,059, IQR: 424– 3,696), screening ($385, IQR: 147–1,204) and least in companion tests ($220, IQR: 108–439).
  - Median incremental QALYs per person were 1.68 for gene therapy (IQR: 0·32–6·92) and around 0·03 across varied genetic tests, i·e., screening (0·027, IQR: 0·004–0·230), diagnostic (0·04, IQR: 0·002–0·156), prognostic (0·059, IQR: 0·004–0·16), companion (0·031, IQR: 0·003–0·158).
- Adaptability: 67% of CEA concluded the PM was cost-effective, 24% found it not cost-effective, while the rest (8%) were inconclusive. Only around 1/10 of those findings were transferable to other settings.
- Modelling:
  - Stage: 3/4 were conventional CEAs for market access, 1/4 were early cycle CEAs.
  - Perspective: the majority (77%) used a healthcare system’s perspective, while less than 20% used a societal perspective
  - Model type: ranged from simple decision model and Markov model to complex hybrid model and discrete event simulation across varied PM categories.
  - Sensitivity analysis: the vast majority performed at least one type of sensitivity analysis, and 84% of studies conducted one-way sensitivity analysis.
  - Budget impact analysis: less than 5% included budget impact analysis.

**Time trend of economic evaluations on precision medicine**


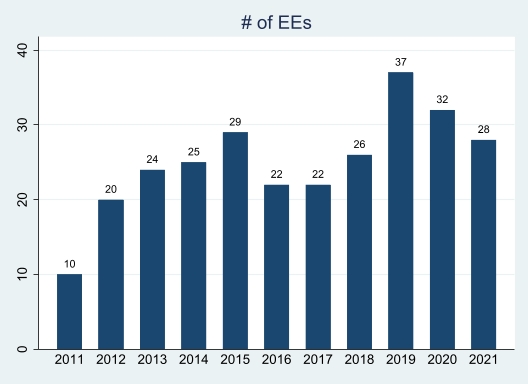


Between 2011 and 2021, the number of CEAs appeared to slowly rise amid temporal fluctuations.

**Appendix 2 Table 1. General and economic characteristics of cost effectiveness analyses reporting precision medicine interventions**

| **Characteristic** | **Genetic testing (N=238)** | **Gene therapy (N=37)** |
| --- | --- | --- |
|  |  |  |
| **Incremental Effectiveness (in QALY or LY)** | | |
| Mean (SD) | 0·34 (1·78) | 3·83 (4·71) |
| **PM Unit Cost** | | |
| Mean (SD) | 1,949 (9,394) | 447,943 (585,619) |
| **WHO region** | | |
| African Region (AFR) | 1 (0·4%) | 0 (0%) |
| Region of the Americas (AMR) | 111 (47%) | 21 (57%) |
| South-East Asian Region (SEAR) | 10 (4·2%) | 1 (2·7%) |
| European Region (EUR) | 72 (30%) | 7 (19%) |
| Eastern Mediterranean Region (EMR) | 1 (0·4%) | 1 (2·7%) |
| Western Pacific Region (WPR) | 43 (18%) | 7 (19%) |
| **Country-income level** | | |
| Lower-middle income | 5 (2·1%) | 0 (0%) |
| Upper middle-income | 23 (9·7%) | 3 (8·1%) |
| High-income | 210 (88%) | 34 (92%) |
| **Age** | | |
| Pediatric | 9 (3·8%) | 7 (19%) |
| Adult | 191 (80%) | 20 (54%) |
| All ages/Not specified | 38 (16%) | 10 (27%) |
| **Sex** | | |
| All-Male | 11 (4·6%) | 5 (14%) |
| All-Female | 60 (25%) | 3 (8·1%) |
| Mixed-Sex | 167 (70%) | 29 (78%) |
| **ICD 10 Disease categories** | | |
| I Certain infectious and parasitic diseases (A00-B99) | 15 (6·3%) | 2 (5·4%) |
| II Neoplasms (C00-D48) | 126 (53%) | 23 (62%) |
| III Diseases of the blood and blood-forming organs and certain disorders involving the immune mechanism  (D50-D89) | 3 (1·3%) | 4 (11%) |
| IV Endocrine, nutritional and metabolic diseases (E00-E90) | 11 (4·6%) | 1 (2·7%) |
| V Mental and behavioural disorders (F00-F99) | 10 (4·2%) | 0 (0%) |
| VI Diseases of the nervous system (G00-G99) | 7 (2·9%) | 1 (2·7%) |
| VII Diseases of the eye and adnexa (H00-H59) | 0 (0%) | 4 (11%) |
| IX Diseases of the circulatory system (I00-I99) | 40 (17%) | 1 (2·7%) |
| X Diseases of the respiratory system (J00-J99) | 1 (0·4%) | 0 (0%) |
| XI Diseases of the digestive system (K00-K93) | 3 (1·3%) | 0 (0%) |
| XII Diseases of the skin and subcutaneous tissue (L00-L99) | 2 (0·8%) | 0 (0%) |
| XIII Diseases of the musculoskeletal system and connective tissue (M00-M99) | 7 (2·9%) | 0 (0%) |
| XVIII Symptoms, signs and abnormal clinical and laboratory findings, not elsewhere classified (R00-R99) | 1 (0·4%) | 0 (0%) |
| XIX Injury, poisoning and certain other consequences of external causes (S00-T98) | 3 (1·3%) | 0 (0%) |
| XXI Factors influencing health status and contact with health services (Z00-Z99) | 9 (3·8%) | 1 (2·7%) |
| **Type of funders** | | |
| Public | 90 (38%) | 10 (27%) |
| Private: Not-for profit | 15 (6·3%) | 8 (22%) |
| Private: For-profit | 45 (19%) | 11 (30%) |
| Mixed funding source | 19 (8·0%) | 1 (2·7%) |
| Not stated | 69 (29%) | 7 (19%) |
| **Perspective** | | |
| Societal | 41 (17%) | 5 (14%) |
| Healthcare | 181 (76%) | 30 (81%) |
| Other (e·g·, patient perspective) | 16 (7%) | 2 (5·4%) |
| **Type of comparators** | | |
| Standard of care | 227 (95%) | 36 (97%) |
| Others | 11 (5%) | 1 (2·7%) |
| **Type of outcomes** | | |
| QALYs | 227 (95%) | 37 (100%) |
| Life-years | 11 (5%) | 0 (0%) |
| **EE type** | | |
| By PM stage | | |
| Early EE | 59 (25%) | 10 (27%) |
| Traditional EE | 179 (75%) | 27 (73%) |
| By study design | | |
| Model-based | 230 (97%) | 34 (92%) |
| Along-side clinical trial | 8 (3%) | 3 (8·1%) |
| **Model type** | | |
| Markov model | 73 (31%) | 16 (43%) |
| Decision tree model | 44 (18%) | 3 (8·1%) |
| Hybrid model (Decision tree + Markov) | 93 (39%) | 11 (30%) |
| Discrete event simulation | 22 (9·2%) | 4 (11%) |
| Other type of analysis (non-model) | 1 (0·4%) | 1 (2·7%) |
| Not specified | 5 (2·1%) | 2 (5·4%) |
| **Time horizon** | | |
| Short term (0<T≤3 yrs) | 30 (13%) | 2 (5·4%) |
| Intermediate (3<T≤10 yrs) | 42 (18%) | 6 (16%) |
| Long term (10<T≤30 yrs) | 25 (11%) | 4 (11%) |
| Lifetime (T>30 yrs) | 137 (58%) | 24 (65%) |
| Not reported | 4 (1·7%) | 1 (2·7%) |
| **Conclusion** | | |
| Cost-effective/cost-saving | 162 (68%) | 23 (62%) |
| Not cost-effective | 57 (24%) | 10 (27%) |
| Inconclusive | 19 (8%) | 4 (11%) |
| **Generalizability** | | |
| Yes | 26 (11%) | 5 (14%) |
| No | 84 (35%) | 10 (27%) |
| Not reported | 128 (54%) | 22 (59%) |
| **Presence of conflict of interest** | | |
| Yes | 55 (23%) | 10 (27%) |
| No | 132 (55%) | 21 (57%) |
| Not reported | 51 (21%) | 6 (16%) |
| **PM-specific consideration** | | |
| Test accuracy | | |
| Considered | 51 (21%) | 1 (2·7%) |
| No | 187 (79%) | 26 (70%) |
| Not reported | 0 (0%) | 10 (27%) |
| Test uptake | | |
| Integrated | 54 (23%) | 5 (14%) |
| No | 184 (77%) | 21 (57%) |
| Not reported | 0 (0%) | 11 (30%) |
| Treatment compliance | | |
| Integrated | 42 (18%) | 5 (14%) |
| No | 195 (82%) | 31 (84%) |
| Not reported | 1 (0·4%) | 1 (2·7%) |
| **Budget impact analysis** | | |
| Performed | 12 (5·0%) | 1 (2·7%) |
| No | 221 (93%) | 34 (92%) |
| Not reported | 5 (2·1%) | 2 (5·4%) |
| **Sensitivity analysis** | | |
| One-way | 202 (85%) | 30 (81%) |
| Multi-way | 38 (16%) | 4 (11%) |
| Probabilistic | 160 (67%) | 28 (76%) |
| Other analysis (VOI, scenario) | 22 (9·2%) | 6 (16%) |

**Appendix 2 Table 2. General and economic characteristics of cost effectiveness analyses reporting precision medicine interventions, by intervention scope of genetic testing.**

| **Characteristic** | **Screening (N=52)** | **Diagnostic (N=27)** | **Prognostic (N=53)** | **Companion (N=106)** |
| --- | --- | --- | --- | --- |
|  |  |  |  |  |
| **Incremental QALY** | | | | |
| Mean (SD) | 0·73 (3·45) | 0·23 (0·57) | 0·07 (0·49) | 0·31 (1·08) |
| **PM Unit Cost** | | | | |
| Mean (SD) | 4,190 (19,839) | 2,269 (2,328) | 2,488 (1,633) | 500 (752) |
| **WHO region** | | | | |
| African Region (AFR) | 0 (0%) | 0 (0%) | 0 (0%) | 1 (0·9%) |
| Region of the Americas (AMR) | 24 (46%) | 15 (56%) | 26 (49%) | 46 (43%) |
| South-East Asian Region (SEAR) | 0 (0%) | 0 (0%) | 0 (0%) | 10 (9·4%) |
| European Region (EUR) | 17 (33%) | 10 (37%) | 22 (42%) | 23 (22%) |
| Eastern Mediterranean Region (EMR) | 1 (1·9%) | 0 (0%) | 0 (0%) | 0 (0%) |
| Western Pacific Region (WPR) | 10 (19%) | 2 (7·4%) | 5 (9·4%) | 26 (25%) |
| **Country-income level** | | | | |
| Lower-middle income | 1 (1·9%) | 0 (0%) | 0 (0%) | 4 (3·8%) |
| Upper middle-income | 1 (1·9%) | 2 (7·4%) | 2 (3·8%) | 18 (17%) |
| High-income | 50 (96%) | 25 (93%) | 51 (96%) | 84 (79%) |
| **Age** | | | | |
| Pediatric | 5 (9·6%) | 1 (3·7%) | 1 (1·9%) | 2 (1·9% |
| Adult | 41 (79%) | 23 (85%) | 45 (85%) | 82 (77%) |
| All ages/Not specified | 6 (12%) | 3 (11%) | 7 (13%) | 22 (21%) |
| **Sex** | | | | |
| All-Male | 5 (9·6%) | 1 (3·7%) | 4 (7·5%) | 1 (0·9%) |
| All-Female | 18 (35%) | 6 (22%) | 29 (55%) | 7 (6·6%) |
| Mixed-Sex | 29 (56%) | 20 (74%) | 20 (38%) | 98 (92%) |
| **ICD 10 Disease categories** | | | | |
| I Certain infectious and parasitic diseases  (A00-B99) | 1 (1·9%) | 1 (3·7%) | 2 (3·8%) | 11 (10%) |
| II Neoplasms (C00-D48) | 28 (54%) | 19 (70%) | 46 (87%) | 33 (31%) |
| III Diseases of the blood and blood-forming organs and certain disorders involving the immune mechanism (D50-D89) | 2 (3·8%) | 0 (0%) | 0 (0%) | 1 (0·9%) |
| IV Endocrine, nutritional and metabolic diseases (E00-E90) | 7 (13%) | 2 (7·4%) | 2 (3·8%) | 0 (0%) |
| V Mental and behavioural disorders (F00-F99) | 0 (0%) | 0 (0%) | 0 (0%) | 10 (9·4%) |
| VI Diseases of the nervous system (G00-G99) | 1 (1·9%) | 1 (3·7%) | 0 (0%) | 5 (4·7%) |
| VII Diseases of the eye and adnexa (H00-H59) | 0 (0%) | 0 (0%) | 0 (0%) | 0 (0%) |
| IX Diseases of the circulatory system (I00-I99) | 7 (13%) | 1 (3·7%) | 1 (1·9%) | 31 (29%) |
| X Diseases of the respiratory system (J00-J99) | 0 (0%) | 0 (0%) | 0 (0%) | 1 (0·9%) |
| XI Diseases of the digestive system (K00-K93) | 1 (1·9%) | 1 (3·7%) | 1 (1·9%) | 0 (0%) |
| XII Diseases of the skin and subcutaneous tissue (L00-L99) | 0 (0%) | 0 (0%) | 0 (0%) | 2 (1·9%) |
| XIII Diseases of the musculoskeletal system and connective tissue (M00-M99) | 1 (1·9%) | 1 (3·7%) | 0 (0%) | 5 (4·7%) |
| XVIII Symptoms, signs and abnormal clinical and laboratory findings, not elsewhere classified  (R00-R99) | 1 (1·9%) | 0 (0%) | 0 (0%) | 0 (0%) |
| XIX Injury, poisoning and certain other consequences of external causes (S00-T98) | 0 (0%) | 0 (0%) | 0 (0%) | 3 (2·8%) |
| XXI Factors influencing health status and contact with health services (Z00-Z99) | 3 (5·8%) | 1 (3·7%) | 1 (1·9%) | 4 (3·8%) |
| **Type of funders** | | | | |
| Public | 22 (42%) | 9 (33%) | 17 (32%) | 42 (40%) |
| Private: Not-for profit | 5 (9·6%) | 0 (0%) | 5 (9·4%) | 5 (4·7%) |
| Private: For-profit | 7 (13%) | 7 (26%) | 14 (26%) | 17 (16%) |
| Mixed funding source | 3 (5·8%) | 2 (7·4%) | 6 (11%) | 8 (7·5%) |
| Not stated | 15 (29%) | 9 (33%) | 11 (21%) | 34 (32%) |
| **Perspective** | | | | |
| Societal | 8 (15%) | 5 (19%) | 7 (13%) | 21 (20%) |
| Healthcare | 41 (79%) | 19 (70%) | 42 (79%) | 79 (75%) |
| Other (e·g·, patient perspective) | 3 (5·8%) | 3 (11%) | 4 (7·5%) | 6 (5·7%) |
| **Type of comparators** | | | | |
| Standard of care | 47 (90%) | 24 (89%) | 51 (96%) | 105 (99%) |
| Others | 5 (9·6%) | 3 (11%) | 2 (3·8%) | 1 (0·9%) |
| **Type of outcomes** | | | | |
| QALYs | 47 (90%) | 26 (96%) | 50 (94%) | 104 (98%) |
| Life-years | 5 (9·6%) | 1 (3·7%) | 3 (5·7%) | 2 (1·9%) |
| **EE type** | | | | |
| By PM stage | | | | |
| Early EE | 12 (23%) | 4 (15%) | 12 (23%) | 31 (29%) |
| Traditional EE | 40 (77%) | 23 (85%) | 41 (77%) | 75 (71%) |
| By study design | | | | |
| Model-based | 52 (100%) | 27 (100%) | 52 (98%) | 99 (93%) |
| Along-side clinical trial | 0 (0%) | 0 (0%) | 1 (1·9%) | 7 (6·6%) |
| **Model type** | | | | |
| Markov model | 15 (29%) | 6 (22%) | 20 (38%) | 32 (30%) |
| Decision tree model | 12 (23%) | 5 (19%) | 4 (7·5%) | 23 (22%) |
| Hybrid model (Decision tree + Markov) | 22 (42%) | 10 (37%) | 20 (38%) | 41 (39%) |
| Discrete event simulation | 3 (5·8%) | 5 (19%) | 8 (15%) | 6 (5·7%) |
| Other type of analysis (non-model) | 0 (0%) | 0 (0%) | 0 (0%) | 1 (0·9%) |
| Not specified | 0 (0%) | 1 (3·7%) | 1 (1·9%) | 3 (2·8%) |
| **Time horizon** | | | | |
| Short term (0<T≤3 yrs) | 1 (1·9%) | 3 (11%) | 1 (1·9%) | 25 (24%) |
| Intermediate (3<T≤10 yrs) | 4 (7·7%) | 3 (11%) | 16 (30%) | 19 (18%) |
| Long term (10<T≤30 yrs) | 3 (5·8%) | 5 (19%) | 6 (11%) | 11 (10%) |
| Lifetime (T>30 yrs) | 43 (83%) | 16 (59%) | 29 (55%) | 49 (46%) |
| Not reported | 1 (1·9%) | 0 (0%) | 1 (1·9%) | 2 (1·9%) |
| **Conclusion** | | | | |
| Cost-effective/cost-saving | 39 (75%) | 21 (78%) | 36 (68%) | 66 (62%) |
| Not cost-effective | 10 (19%) | 5 (19%) | 11 (21%) | 31 (29%) |
| Inconclusive | 3 (5·8%) | 1 (3·7%) | 6 (11%) | 9 (8·5%) |
| **Generalizability** | | | | |
| Yes | 9 (17%) | 2 (7·4%) | 5 (9·4%) | 10 (9·4%) |
| No | 16 (31%) | 12 (44%) | 18 (34%) | 38 (36%) |
| Not reported | 27 (52%) | 13 (48%) | 30 (57%) | 58 (55%) |
| **Presence of conflict of interest** | | | | |
| Yes | 14 (27%) | 5 (19%) | 15 (28%) | 21 (20%) |
| No | 28 (54%) | 14 (52%) | 24 (45%) | 66 (62%) |
| Not reported | 10 (19%) | 8 (30%) | 14 (26%) | 19 (18%) |
| **PM-specific consideration** | | | | |
| Test accuracy | | | | |
| Considered | 21 (40%) | 13 (48%) | 8 (15%) | 9 (8·5%) |
| No | 31 (60%) | 14 (52%) | 45 (85%) | 97 (92%) |
| Not reported | 0 (0%) | 0 (0%) | 0 (0%) | 0 (0%) |
| Test uptake | | | | |
| Integrated | 20 (38%) | 7 (26%) | 7 (13%) | 20 (19%) |
| No | 32 (62%) | 20 (74%) | 46 (87%) | 86 (81%) |
| Not reported | 0 (0%) | 0 (0%) | 0 (0%) | 0 (0%) |
| Treatment compliance | | | | |
| Integrated | 18 (35%) | 3 (11%) | 7 (13%) | 14 (13%) |
| No | 34 (65%) | 24 (89%) | 46 (87%) | 91 (86%) |
| Not reported | 0 (0%) | 0 (0%) | 0 (0%) | 1 (0·9%) |
| **Budget impact analysis** | | | | |
| Performed | 2 (3·8%) | 3 (11%) | 4 (7·5%) | 3 (2·8%) |
| No | 49 (94%) | 23 (85%) | 48 (91%) | 101 (95%) |
| Not reported | 1 (1·9%) | 1 (3·7%) | 1 (1·9%) | 2 (1·9%) |
| **Sensitivity analysis** | | | | |
| One-way | 50 (96%) | 20 (74%) | 41 (77%) | 91 (86%) |
| Multi-way | 4 (7·7%) | 5 (19%) | 10 (19%) | 19 (18%) |
| Probabilistic | 35 (67%) | 18 (67%) | 33 (62%) | 74 (70%) |
| Other analysis (VOI, scenario) | 2 (3·8%) | 3 (11%) | 7 (13%) | 10 (9·4%) |
