# Supplementary material for: Mapping the value for money of precision medicine: a systematic literature review and meta-analysis
Source: Front Public Health. 2023 Nov 24;11:1151504. doi: 10.3389/fpubh.2023.1151504 (PMC10704154; doi:10.3389/fpubh.2023.1151504)
Supplement: Supplementary file 2 [file Table_2.DOCX]

**Appendix 5. ECOBIAS assessment of the systematic review**

**N (%) of “Yes” responses for each domain (excluding NA)**

|  |  | **Screening test**  **(N=52)** | **Diagnostic test**  **(N=27)** | **Prognostic test**  **(N=53)** | **Companion test**  **(N=106)** | **Gene therapy**  **(N=37)** | **p-value** |
| --- | --- | --- | --- | --- | --- | --- | --- |
| Narrow perspective bias | Was a societal perspective adopted? | 10 (19.61) | 6 (24.00) | 11 (21.57) | 26 (25.24) | 9 (25.00) | 0.944 |
|  | If not, has a different perspective been justified? | 13 (31.71) | 10 (52.63) | 11 (27.50) | 24 (31.17) | 14 (51.85) | 0.327 |
| Inefficient Comparator Bias | Was the best alternative chosen as comparator? | 45 (95.74) | 25 (92.59) | 45 (97.83) | 100 (95.24) | 34 (94.44) | 0.747 |
|  | Was current practice chosen as a comparator? | 47 (94.00) | 24 (88.89) | 51 (96.23) | 105 (100.00) | 36 (100.00) | **0.009** |
|  | Have all comparators been described in sufficient detail? | 47 (90.38) | 24 (88.89) | 47 (88.68) | 101 (95.28) | 32 (88.89) | 0.199 |
| Cost Measurement Omission Bias | All costs relevant to the disease and intervention identified and considered? | 15 (28.85) | 16 (59.26) | 23 (44.23) | 43 (40.57) | 13 (37.14) | 0.173 |
| Intermittent Data Collection Bias | Was the resource use measured continuously? | 13 (27.66) | 8 (34.78) | 11 (25.58) | 29 (27.88) | 6 (17.14) | 0.560 |
| Invalid Valuation Bias | Is the price calculation presented in a detailed manner? | 33 (63.46) | 16 (59.26) | 41 (77.36) | 76 (71.70) | 22 (59.46) | 0.076 |
|  | Have reference prices been used? | 44 (84.62) | 22 (84.62) | 43 (82.69) | 91 (87.50) | 30 (81.08) | 0.820 |
| Ordinal ICER bias | Have cardinal scales for the outcomes measure in a CEA been used? | 47 (90.38) | 23 (85.19) | 47 (88.68) | 101 (96.19) | 34 (91.89) | 0.270 |
| Double-counting bias | Are variables adequately checked for double-counting? | 3 (6.25) | 2 (9.52) | 4 (9.52) | 5 (4.95) | 1 (3.23) | 0.745 |
| Inappropriate discounting bias | Have discounting rates from guidelines been applied? (3%, 5%, 3-5%) | 47 (92.16) | 24 (92.31) | 42 (95.45) | 81 (79.41) | 32 (94.12) | **0.018** |
| Limited sensitivity analysis bias | Sensitivity analysis: 4 principles of uncertainty considered? | 12 (23.08) | 3 (11.11) | 8 (15.09) | 12 (11.32) | 7 (18.92) | 0.126 |
| Sponsor bias | Have sponsorships been disclosed? | 39 (82.98) | 20 (76.92) | 46 (90.20) | 76 (75.25) | 30 (83.33) | 0.216 |
| Structural assumptions bias | Is the model structure in line with coherent theory? | 43 (87.76) | 27 (100.00) | 48 (97.96) | 96 (92.31) | 35 (94.59) | 0.161 |
|  | Do treatment pathways reflect the nature of disease? | 51 (100.00) | 25 (96.15) | 51 (100.00) | 103 (98.10) | 37 (100.00) | 0.127 |
| No treatment comparator bias | Is there an adequate comparator, i.e. care as usual? | 49 (96.08) | 25 (92.59) | 50 (94.34) | 105 (99.06) | 36 (97.30) | 0.138 |
| Wrong model bias | Is the model chosen adequate regarding the decision problem? | 47 (94.00) | 24 (100.00) | 48 (97.96) | 101 (96.19) | 35 (97.22) | 0.690 |
| Limited time horizon bias | Was a lifetime horizon chosen? | 33 (63.46) | 18 (66.67) | 24 (46.15) | 58 (54.72) | 26 (70.27) | 0.202 |
|  | Were shorter time horizons adequately justified? | 8 (42.11) | 4 (44.44) | 10 (35.71) | 28 (58.33) | 7 (63.64) | 0.661 |
| Bias related to data identification | Are the methods of data identification transparent? | 40 (76.92) | 24 (88.89) | 43 (82.69) | 86 (81.13) | 22 (59.46) | 0.102 |
|  | Are all choices justified adequately? | 35 (67.31) | 22 (81.48) | 41 (78.85) | 78 (73.58) | 25 (67.57) | 0.461 |
|  | Do the input parameters come from high-quality and well-designed studies? | 25 (49.02) | 13 (54.17) | 37 (74.00) | 80 (76.19) | 20 (55.56) | **<0.001** |
| Bias related to baseline data | Are probabilities, for example, based on natural history data? | 42 (87.50) | 19 (70.37) | 36 (76.60) | 86 (82.69) | 27 (72.97) | 0.254 |
|  | Is transformation of rates into transition probabilities done accurately? | 40 (93.02) | 17 (94.44) | 27 (90.00) | 76 (89.41) | 25 (96.15) | 0.804 |
| Bias related to treatment effects | Relative treatment effects synthesized using meta-analytic techniques? | 19 (39.58) | 5 (23.81) | 26 (55.32) | 51 (50.00) | 11 (31.43) | 0.077 |
|  | Are extrapolations documented and well justified? | 35 (71.43) | 12 (54.55) | 35 (71.43) | 75 (72.12) | 25 (69.44) | 0.321 |
|  | Are alternative assumptions explored regarding extrapolation? | 10 (20.41) | 4 (16.67) | 13 (27.66) | 23 (22.12) | 15 (41.67) | 0.244 |
| Bias related to quality-of-life weights (utilities) | Are the utilities incorporated appropriate for the specific decision problem? | 44 (95.65) | 23 (85.19) | 44 (89.80) | 97 (93.27) | 33 (97.06) | 0.373 |
| Non-transparent data incorporation bias | Is the process of data incorporation transparent? | 37 (72.55) | 24 (88.89) | 43 (82.69) | 90 (84.91) | 30 (83.33) | 0.426 |
|  | Are all data and their sources described in detail? | 42 (80.77) | 22 (81.48) | 47 (88.68) | 91 (86.67) | 31 (83.78) | 0.807 |
| Limited scope bias | Scope: 4 principles of uncertainty considered? | 10 (19.23) | 4 (14.81) | 8 (15.09) | 14 (13.33) | 7 (18.92) | 0.651 |
