# Supplementary material for: Mapping the value for money of precision medicine: a systematic literature review and meta-analysis
Source: Front Public Health. 2023 Nov 24;11:1151504. doi: 10.3389/fpubh.2023.1151504 (PMC10704154; doi:10.3389/fpubh.2023.1151504)
Supplement: Supplementary file 3 [file Table_3.DOCX]

**Appendix 7. Publication bias assessment**

**Appendix Figure 7.1. Funnel plot of pooling NMBs of genetic test**

**
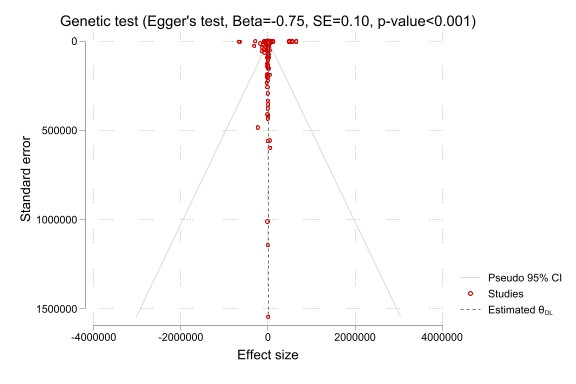
**

**Appendix Figure 7.2. Funnel plot of pooling NMBs of genetic test for screening**

**
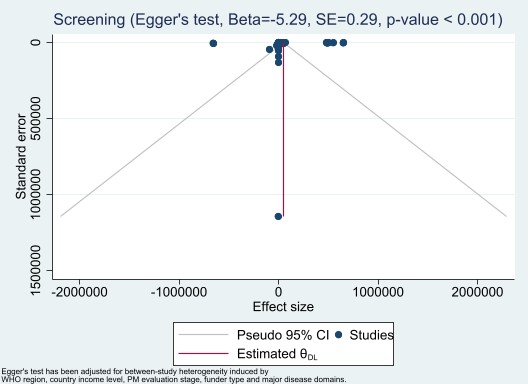
**

**Appendix Figure 7.3. Funnel plot of pooling NMBs of genetic test for diagnosis**

**
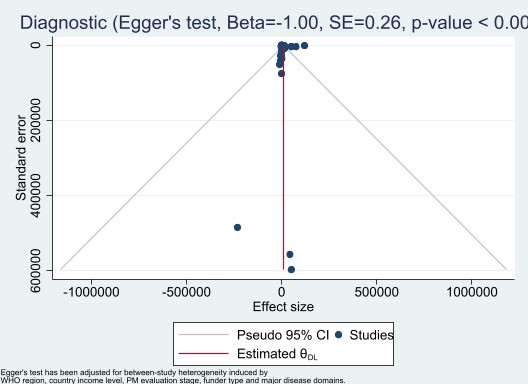
**

**Appendix Figure 7.4. Funnel plot of pooling NMBs of genetic test for prognostic diagnosis**

**
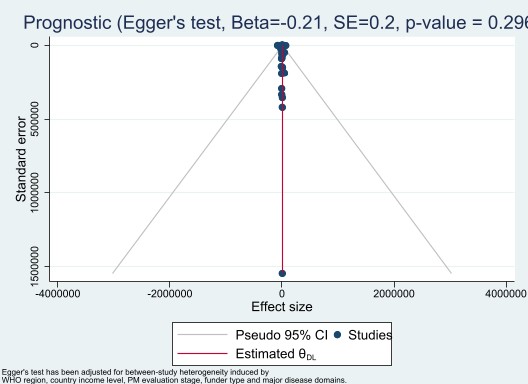
**

**Appendix Figure 7.5. Funnel plot of pooling NMBs of genetic test for companion diagnostics**

**
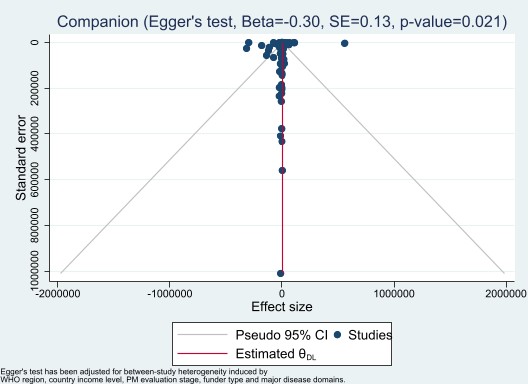
**

**Appendix Figure 7.6. Funnel plot of pooling NMBs of gene therapy**

**
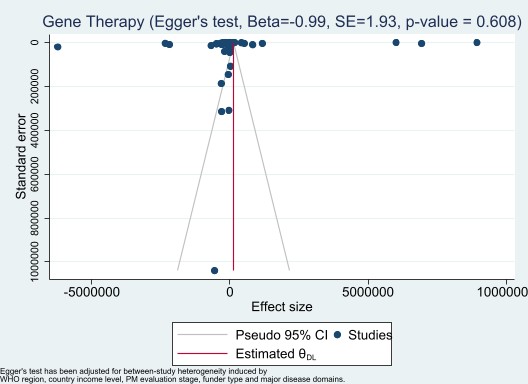
**
