# Supplementary material for: Mapping the value for money of precision medicine: a systematic literature review and meta-analysis
Source: Front Public Health. 2023 Nov 24;11:1151504. doi: 10.3389/fpubh.2023.1151504 (PMC10704154; doi:10.3389/fpubh.2023.1151504)
Supplement: Supplementary file 4 [file Table_4.DOCX]

**Appendix 8. Univariate, random-effect meta-regression results**

**Appendix 5 Table 1. Parameter estimates from the univariate, random-effect meta-regression on incremental net monetary benefit of genetic testing and gene therapy**

|  | **Genetic Testing** | | **Gene Therapy** | |
| --- | --- | --- | --- | --- |
| **Variable** | **Coef (95% CI)** | **p-value** | **Coef (95% CI)** | **p-value** |
| **Incremental effectiveness (QALY or LY)** | 55830 (55196, 56463) | **<0·001** | -35723 (-294185, 222740) | 0·786 |
| **PM Unit Cost** | 0·1 (-0·1, 0·3) | 0·223 | 2 (1·4, 2·7) | **<0·001** |
| **Year of publication** | -1236 (-1984, -489) | **0·001** | 81133 (-310033, 472300) | 0·684 |
| **Disease Incidence per 100,000 population** | -1·3 (-2·2, -0·4) | **0·004** | -1094 (-17751, 15563) | 0·898 |
| **Perspective adopted** | | | | |
| Social | (Reference) | | | |
| Healthcare | -13881 (-18526, -9237) | **<0·001** | 659125 (-414200, 1732449) | 0·229 |
| Other (e·g·, patient perspective) | -73294 (-81097, -65492) | **<0·001** | 4508393 (2913384, 6103402) | **<0·001** |
| **Type of analysis used for model** | | | | |
| Decision tree model | (Reference) | | | |
| Markov model | -9131 (-13236, -5026) | **<0·001** | 697600 (-2171708, 3566908) | 0·634 |
| Hybrid model (Decision tree + Markov) | 13507 (9671, 17343) | **<0·001** | -52850 (-2931252, 2825553) | 0·971 |
| Discrete event simulation | 55355 (50455, 60256) | **<0·001** | -680467 (-4219640, 2858707) | 0·706 |
| Other type of analysis (non-model) | 11479 (-169021, 191980) | 0·901 | 507681 (-5390846, 6406209) | 0·866 |
| Not specified | -8926 (-30096, 12245) | 0·409 | 415781 (-3613715, 4445276) | 0·84 |
| **Time Horizon** | 494 (449, 539) | **<0·001** | 2400 (-21841, 26642) | 0·846 |
| **Test accuracy integrated** | 6222 (2673, 9772) | **0·001** | -124988 (-7966179, 7716203) | 0·975 |
| **Test uptake integrated** | 20199 (15136, 25263) | **<0·001** | -35708 (-3112328, 3040911) | 0·982 |
| **Treatment compliance integrated** | 87020 (84208, 89833) | **<0·001** | -1271926 (-4376369, 1832516) | 0·422 |
| **Cascade testing included** | 62441 (57948, 66934) | **<0·001** | NA (None included) | |
| **Source of intervention effectiveness data** | | | | |
| Meta-analysis of RCTs, measuring final outcomes | (Reference) | | | |
| Single RCT, measuring final outcomes | -32051 (-36388, -27714) | **<0·001** | 432289 (-1224937, 2089514) | 0·609 |
| RCTs, measuring surrogate outcomes | -24849 (-32298, -17400) | **<0·001** | 2330443 (403841, 4257045) | **0·018** |
| Case control or cohort studies | -13054 (-17187, -8922) | **<0·001** | 996657 (-675120, 2668435) | 0·243 |
| Non-analytic studies | -33830 (-43170, -24491) | **<0·001** | 379695 (-4015333, 4774722) | 0·866 |
| Expert opinion | -38826 (-57102, -20549) | **<0·001** | 709126 (-3685888, 5104139) | 0·752 |
| Not clearly stated | -32018 (-38770, -25267) | **<0·001** | NA | |
| **Source of cost data** | | | | |
| Primary data collected | (Reference) | | | |
| Other studies (same setting) | 12818 (8826, 16810) | **<0·001** | 1024220 (-1982825, 4031265) | 0·504 |
| Secondary sources (same setting) | -20012 (-25144, -14879) | **<0·001** | 651860 (-3002839, 4306558) | 0·727 |
| Other studies (other settings) | -14615 (-27538, -1692) | **0·027** | NA | |
| Secondary sources (other settings) | -14848 (-40100, 10404) | 0·249 | 182029 (-7973598, 8337655) | 0·965 |
| **Were surrogate outcomes used?** | -22047 (-28105, -15990) | **<0·001** | -360649 (-3997636, 3276338) | 0·846 |
| **% Yes among all ECOBIAS Variables, per 1% increase** | 1306 (1178, 1434) | **<0·001** | -16079 (-115000, 82711) | 0·75 |
| **% Yes among model-specific ECOBIAS Variables, per 1% increase** | 622 (488, 755) | **<0·001** | 2213 (-63691, 68118) | 0·948 |
| **Conflict of interest** | | | | |
| No | (Reference) | | | |
| Yes | 15170 (11768, 18571) | **<0·001** | -11186 (-1680532, 1658161) | 0·99 |
| Not reported | 50420 (47305, 53535) | **<0·001** | -204855 (-2547997, 2138288) | 0·864 |
| **Target age** | | | | |
| Adult | (Reference) | | | |
| Pediatric | -15481 (-23263, -7699) | **<0·001** | -1486289 (-4025567, 1052988) | 0·251 |
| All ages/Not specified | -26680 (-30861, -22499) | **<0·001** | -757962 (-3230749, 1714825) | 0·548 |
| **Target sex** | | | | |
| Mixed-Sex | (Reference) | | | |
| All-Male | -8181 (-19401, 3039) | 0·153 | 4317063 (3277951, 5356175) | **<0·001** |
| All-Female | 25659 (20854, 30463) | **<0·001** | 59519 (-1614835, 1733872) | 0·944 |

**Appendix 5 Table 2. Parameter estimates from the univariate, random-effect meta-regression on incremental net monetary benefit of screening test**

|  | **Screening** | |
| --- | --- | --- |
| **Variable** | **Coef (95% CI)** | **p-value** |
| Incremental effectiveness (QALY or LY) | 60256 (59806, 60706) | <0·001 |
| PM Unit Cost | -0·2 (-0·6, 0·3) | 0·474 |
| Year of publication | -5947 (-8605, -3290) | <0·001 |
| Disease Incidence per 100,000 population | -5 (-6·8, -3·2) | <0·001 |
| **Perspective adopted** | | |
| Social | (Reference) | |
| Healthcare | -38832 (-61521, -16144) | 0·001 |
| Other (e·g·, patient perspective) | -416003 (-455105, -376902) | <0·001 |
| **Type of analysis used for model** | | |
| Decision tree model | (Reference) | |
| Markov model | 3405 (-12039, 18848) | 0·666 |
| Hybrid model (Decision tree + Markov) | 49104 (35131, 63077) | <0·001 |
| Discrete event simulation | 153526 (136771, 170281) | <0·001 |
| Time Horizon | 2077 (1872, 2281) | <0·001 |
| Test accuracy integrated | -8037 (-24848, 8773) | 0·349 |
| Test uptake integrated | -26998 (-42131, -11865) | <0·001 |
| Treatment compliance integrated | 157749 (140758, 174740) | <0·001 |
| Cascade testing included | 46533 (29360, 63707) | <0·001 |
| **Source of intervention effectiveness data** | | |
| Meta-analysis of RCTs, measuring final outcomes | (Reference) | |
| Single RCT, measuring final outcomes | -81536 (-114112, -48960) | <0·001 |
| RCTs, measuring surrogate outcomes | -85021 (-141190, -28852) | 0·003 |
| Case control or cohort studies | -68129 (-87792, -48466) | <0·001 |
| Non-analytic studies | -91325 (-147238, -35412) | 0·001 |
| Expert opinion | -91826 (-169736, -13915) | 0·021 |
| Not clearly stated | -91201 (-125765, -56638) | <0·001 |
| **Source of cost data** | | |
| Primary data collected | (Reference) | |
| Other studies (same setting) | 69155 (44964, 93347) | **<0·001** |
| Secondary sources (same setting) | 2105 (-28547, 32758) | 0·893 |
| Secondary sources (other settings) | -574 (-79269, 78121) | 0·989 |
| **Were surrogate outcomes used?** | -47557 (-84160, -10955) | **0·011** |
| % Yes among all ECOBIAS Variables, per 1% increase | 3491 (2775, 4206) | **<0·001** |
| % Yes among model-specific ECOBIAS Variables, per 1% increase | 1954 (1375, 2533) | **<0·001** |
| **Conflict of interest** | | |
| No | (Reference) | |
| Yes | 25535 (19388, 31681) | **<0·001** |
| Not reported | 97055 (91255, 102856) | **<0·001** |
| **Target age** | | |
| Adult | (Reference) | |
| Pediatric | -55920 (-86822, -25018) | **<0·001** |
| All ages/Not specified | -58253 (-86514, -29993) | **<0·001** |
| **Target sex** | | |
| Mixed-Sex | (Reference) | |
| All-Male | -26536 (-59315, 6243) | 0·113 |
| All-Female | 60401 (41249, 79553) | **<0·001** |

**Appendix 5 Table 3. Parameter estimates from the univariate, random-effect meta-regression on incremental net monetary benefit of diagnostic test**

|  | **Diagnostic** | |
| --- | --- | --- |
| **Variable** | **Coef (95% CI)** | **p-value** |
| **Incremental effectiveness (QALY or LY)** | 42588 (41199, 43977) | **<0·001** |
| **PM Unit Cost** | 4·3 (3·7, 5) | **<0·001** |
| **Year of publication** | -2769 (-3203, -2335) | **<0·001** |
| **Disease Incidence per 100,000 population** | 49 (46, 53) | **<0·001** |
| **Perspective adopted** | | |
| Social | (Reference) | |
| Healthcare | 8646 (1664, 15627) | **0·015** |
| Other (e·g·, patient perspective) | 1078 (-12268, 14424) | 0·874 |
| **Type of analysis used for model** | | |
| Decision tree model | (Reference) | |
| Markov model | -19611 (-23603, -15618) | **<0·001** |
| Hybrid model (Decision tree + Markov) | -20818 (-24814, -16821) | **<0·001** |
| Discrete event simulation | 4627 (189, 9066) | **0·041** |
| Not specified | -253669 (-1203247, 695909) | 0·601 |
| **Time Horizon** | 173 (139, 208) | **<0·001** |
| **Test accuracy integrated** | 3573 (958, 6189) | **0·007** |
| **Test uptake integrated** | 2670 (-277, 5616) | 0·076 |
| **Treatment compliance integrated** | -11876 (-14982, -8770) | **<0·001** |
| **Cascade testing included** | -11944 (-15104, -8783) | **<0·001** |
| **Source of intervention effectiveness data** | | |
| Meta-analysis of RCTs, measuring final outcomes | (Reference) | |
| Single RCT, measuring final outcomes | 33271 (27758, 38783) | **<0·001** |
| Case control or cohort studies | 11380 (7432, 15329) | **<0·001** |
| Non-analytic studies | -1346 (-5715, 3023) | 0·546 |
| Not clearly stated | 3385 (-1845, 8615) | 0·205 |
| **Source of cost data** | | |
| Primary data collected | (Reference) | |
| Other studies (same setting) | 8343 (3575, 13110) | **0·001** |
| Secondary sources (same setting) | 9485 (4918, 14051) | **<0·001** |
| Other studies (other settings) | -3312 (-8495, 1872) | 0·211 |
| **Were surrogate outcomes used?** | 4607 (-1717, 10930) | 0·153 |
| **% Yes among all ECOBIAS Variables, per 1% increase** | 839 (675, 1003) | **<0·001** |
| **% Yes among model-specific ECOBIAS Variables, per 1% increase** | 611 (525, 696) | **<0·001** |
| **Conflict of interest** | | |
| No | (Reference) | |
| Yes | -6025 (-10387, -1662) | **0·007** |
| Not reported | 23729 (20030, 27428) | **<0·001** |
| **Target age** | | |
| Adult | (Reference) | |
| Pediatric | 35737 (-762276, 833751) | 0·93 |
| All ages/Not specified | -11109 (-14306, -7912) | **<0·001** |
| **Target sex** | | |
| Mixed-Sex | (Reference) | |
| All-Male | -245644 (-1195219, 703931) | 0·612 |
| All-Female | -13222 (-15990, -10453) | **<0·001** |

**Appendix 5 Table 4. Parameter estimates from the univariate, random-effect meta-regression on incremental net monetary benefit of prognostic test**

|  | **Prognostic** | |
| --- | --- | --- |
| **Variable** | **Coef (95% CI)** | **p-value** |
| **Incremental effectiveness (QALY or LY)** | 27497 (23400, 31595) | **<0·001** |
| **PM Unit Cost** | 0 (-2·2, 2·2) | 0·992 |
| **Year of publication** | -128 (-1316, 1059) | 0·832 |
| **Disease Incidence per 100,000 population** | 24 (-91, 140) | 0·68 |
| **Perspective adopted** | | |
| Social | (Reference) | |
| Healthcare | -15972 (-25383, -6562) | **0·001** |
| Other (e·g·, patient perspective) | -11489 (-25469, 2491) | 0·107 |
| **Type of analysis used for model** | | |
| Decision tree model | (Reference) | |
| Markov model | -1351 (-23532, 20830) | 0·905 |
| Hybrid model (Decision tree + Markov) | -14365 (-36845, 8114) | 0·21 |
| Discrete event simulation | -6197 (-28894, 16499) | 0·593 |
| **Time Horizon** | 28 (-60, 116) | 0·534 |
| **Test accuracy integrated** | 12287 (485, 24089) | **0·041** |
| **Test uptake integrated** | 3252 (-6194, 12699) | 0·5 |
| **Treatment compliance integrated** | 34186 (23746, 44626) | **<0·001** |
| **Cascade testing included** | -468 (-25908, 24972) | 0·971 |
| **Source of intervention effectiveness data** | | |
| Meta-analysis of RCTs, measuring final outcomes | (Reference) | |
| Single RCT, measuring final outcomes | 9115 (201, 18030) | **0·045** |
| RCTs, measuring surrogate outcomes | 6219 (-14402, 26841) | 0·554 |
| Case control or cohort studies | 11629 (1575, 21683) | **0·023** |
| Non-analytic studies | 9217 (-18133, 36567) | 0·509 |
| Expert opinion | -5000 (-33002, 23002) | 0·726 |
| Not clearly stated | 11254 (-3588, 26096) | 0·137 |
| **Source of cost data** | | |
| Primary data collected | (Reference) | |
| Other studies (same setting) | -9448 (-18237, -659) | **0·035** |
| Secondary sources (same setting) | -23886 (-36198, -11574) | **<0·001** |
| **Were surrogate outcomes used?** | -5253 (-15440, 4933) | 0·312 |
| **% Yes among all ECOBIAS Variables, per 1% increase** | 253 (-43, 549) | 0·094 |
| **% Yes among model-specific ECOBIAS Variables, per 1% increase** | 299 (12, 586) | **0·041** |
| **Conflict of interest** | | |
| No | (Reference) | |
| Yes | 13855 (3638, 24072) | **0·008** |
| Not reported | 2486 (-6750, 11723) | 0·598 |
| **Target age** | | |
| Adult | (Reference) | |
| Pediatric | 70804 (46104, 95504) | **<0·001** |
| All ages/Not specified | 14275 (4325, 24224) | **0·005** |
| **Target sex** | | |
| Mixed-Sex | (Reference) | |
| All-Male | 1015 (-14611, 16642) | 0·899 |
| All-Female | 21 (-8793, 8836) | 0·996 |

**Appendix 7 Table 5. Parameter estimates from the univariate, random-effect meta-regression on incremental net monetary benefit of companion test**

|  | **Companion** | |
| --- | --- | --- |
| **Variable** | **Coef (95% CI)** | **p-value** |
| **Incremental effectiveness (QALY or LY)** | 27375 (26496, 28255) | **<0·001** |
| **PM Unit Cost** | 7·4 (5·4, 9·5) | **<0·001** |
| **Year of publication** | 2011 (1509, 2512) | **<0·001** |
| **Disease Incidence per 100,000 population** | -0·1 (-0·6, 0·4) | 0·754 |
| **Perspective adopted** | | |
| Social | (Reference) | |
| Healthcare | -5593 (-9268, -1918) | **0·003** |
| Other (e·g·, patient perspective) | 117932 (111050, 124813) | **<0·001** |
| **Type of analysis used for model** | | |
| Decision tree model | (Reference) | |
| Markov model | -29197 (-32926, -25469) | **<0·001** |
| Hybrid model (Decision tree + Markov) | 4125 (1087, 7164) | **0·008** |
| Discrete event simulation | 23456 (15038, 31874) | **<0·001** |
| Other type of analysis (non-model) | 11015 (-168557, 190586) | 0·904 |
| Not specified | -9341 (-21517, 2834) | 0·133 |
| **Time Horizon** | 92 (64, 120) | **<0·001** |
| **Test accuracy integrated** | -67864 (-72161, -63566) | **<0·001** |
| **Test uptake integrated** | 53759 (50722, 56796) | **<0·001** |
| **Treatment compliance integrated** | -13615 (-16988, -10241) | **<0·001** |
| **Cascade testing included** | NA (None included) | |
| **Source of intervention effectiveness data** | | |
| Meta-analysis of RCTs, measuring final outcomes | (Reference) | |
| Single RCT, measuring final outcomes | -9520 (-12489, -6550) | **<0·001** |
| RCTs, measuring surrogate outcomes | 9112 (4870, 13355) | **<0·001** |
| Case control or cohort studies | 31242 (27812, 34672) | **<0·001** |
| Not clearly stated | -1821 (-7746, 4104) | 0·547 |
| **Source of cost data** | | |
| Primary data collected | (Reference) | |
| Other studies (same setting) | -23934 (-26994, -20874) | **<0·001** |
| Secondary sources (same setting) | -45773 (-49774, -41772) | **<0·001** |
| **Were surrogate outcomes used?** | -20139 (-24640, -15637) | **<0·001** |
| **% Yes among all ECOBIAS Variables, per 1% increase** | -656 (-797, -516) | **<0·001** |
| **% Yes among model-specific ECOBIAS Variables, per 1% increase** | -543 (-652, -435) | **<0·001** |
| **Conflict of interest** | | |
| No | (Reference) | |
| Yes | 4398 (1392, 7405) | **0·004** |
| Not reported | 41036 (38206, 43866) | **<0·001** |
| **Target age** | | |
| Adult | (Reference) | |
| Pediatric | -9697 (-15206, -4189) | **0·001** |
| All ages/Not specified | -16150 (-18931, -13369) | **<0·001** |
| **Target sex** | | |
| Mixed-Sex | (Reference) | |
| All-Male | -5973 (-408237, 396292) | 0·977 |
| All-Female | -1755 (-5142, 1632) | 0·31 |
