# Supplementary material for: Mapping the value for money of precision medicine: a systematic literature review and meta-analysis
Source: Front Public Health. 2023 Nov 24;11:1151504. doi: 10.3389/fpubh.2023.1151504 (PMC10704154; doi:10.3389/fpubh.2023.1151504)
Supplement: Supplementary file 5 [file Table_5.DOCX]

Appendix 7. Top 20 most sensitive parameters in the sensitivity analyses of 275 CEAs. X-axis shows the proportion of studies reported the corresponding item as the most sensitive parameter to the Incremental Cost-effectiveness Ratio.
