# Supplementary material for: Mapping the value for money of precision medicine: a systematic literature review and meta-analysis
Source: Front Public Health. 2023 Nov 24;11:1151504. doi: 10.3389/fpubh.2023.1151504 (PMC10704154; doi:10.3389/fpubh.2023.1151504)

**Appendix 1. Search strategy and results**

**Econlit**

AB,TI(“biomedical technology assessment” OR “economic evaluation” OR “quality adjusted life year” OR “program cost effectiveness” OR (technology NEAR/3 assessment*) OR (economic* NEAR/3 (evaluat* OR value)) OR ((cost OR costs) NEAR/3 (benefit* OR effectiv* OR efficien* OR efficac* OR minim* OR utilit* OR consequen*)) OR (qualit* NEAR/3 adjust* NEAR/3 (life-year* OR lifeyear*)) OR qaly*)

AND

AB,TI(“economic model” OR “simulation” OR (model AND (economics OR “economic aspect”)) OR “decision tree” OR ((model OR modeling OR modelling OR simulation* OR microsimulation*) NEAR/6 (econom* OR pharmacoeconom* OR cost OR costs)) OR (decision NEAR/3 (analy* OR tree OR trees)) OR discrete-event* OR “state transition” OR markov OR ((individual* OR patient-level*) NEAR/3 (sampl* OR simulation*)) OR (dynamic NEAR/3 transmission*) OR probabilistic* OR partition*-survival*)

AND

AB,TI(“personalized medicine” OR “pharmacogenetics” OR “genetic variation” OR “genetic procedures” OR “genotype” OR “biological marker" OR “gene therapy” OR “immunotherapy” OR “pharmacogenomics” OR “pharmacokinetics” OR (“genetic algorithm” AND (risk OR therapy)) OR “omics” OR “pharmacogenetic testing" OR ((genetic OR gene OR genomic) NEAR/3 (variation OR technique OR procedure OR procedures OR test OR testing OR therapy OR sequence OR sequencing OR profile OR profiling)) OR genotype* OR (biologic* NEAR/3 marker*) OR biomarker* OR immunotherap* OR immunetherap* OR pharmacogenomic* OR pharmacogenetic* OR pharmacokinetic* OR pharmacometabolom* OR metabolom* OR proteomic* OR pharmacoproteomic* OR lipidomic* OR pharmacolipidomic* OR omics)


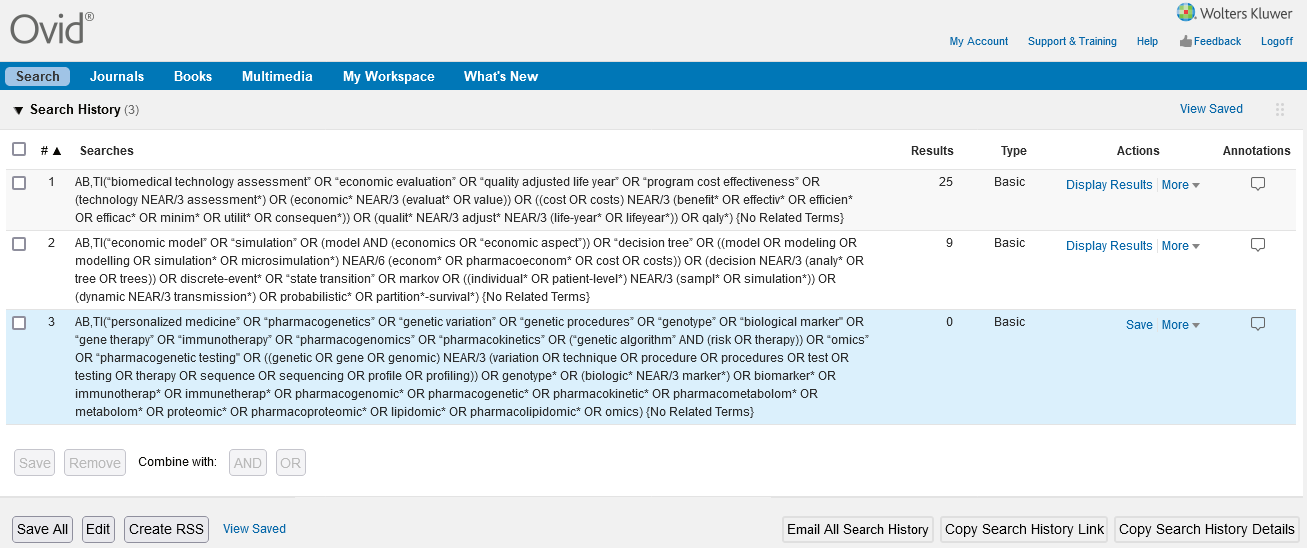


**CRD**

(((personalized OR personalised OR individualised OR individualized OR precision OR stratif* OR targeted* OR algorithm*) NEAR6 (medicine* OR therap* OR treat* OR risk OR regimen* OR dosing* OR duration OR decision*)) OR ((genetic* OR gene OR genom* OR molecular*) NEAR3 (variation* OR technique* OR procedure* OR test OR testing OR therap* OR sequenc* OR profil*)) OR genotype* OR (biologic* NEAR3 marker*) OR biomarker* OR telemonitor* OR (mobile NEAR3 (applicat* OR app OR apps)) OR wearable* OR (personal* NEAR3 digital* NEAR3 assistant*) OR (handheld NEAR3 computer*) OR ((mobile OR cell*) NEAR1 phone*) OR smartphone* OR immunotherap* OR immunetherap* OR immun*-therap* OR pharmacogenomic* OR pharmacogenetic* OR pharmacokinetic* OR (pharmac* NEAR3 (genomic* OR genetic* OR kinetic*)) OR (digital NEAR3 (health OR medicine)) OR mHealth* OR eHealth* OR m-Health* OR e-Health* OR tracker* OR (data NEAR3 analytic*) OR (artificial* NEAR3 intelligen*) OR (machine* NEAR3 learning) OR ((remote OR self) NEAR3 monitor*) OR pharmacometabolom* OR metabolom* OR proteomic* OR pharmacoproteomic* OR lipidomic* OR pharmacolipidomic* OR omics OR (model* NEAR3 (guide* OR base*) NEAR3 (medicine* OR therap* OR treat* OR risk OR regimen* OR dosing* OR duration)) OR (risk NEAR3 score*)):ab,ti

OR MeSH DESCRIPTOR Precision Medicine IN NHSEED,HTA

OR MeSH DESCRIPTOR Pharmacogenetics IN NHSEED,HTA

OR MeSH DESCRIPTOR Pharmacogenetics IN NHSEED,HTA

OR MeSH DESCRIPTOR Genetic Variation IN NHSEED, HTA

OR MeSH DESCRIPTOR Genotype IN NHSEED,HTA

OR MeSH DESCRIPTOR Biomarkers IN NHSEED,HTA

OR MeSH DESCRIPTOR Mobile Applications IN NHSEED,HTA

OR MeSH DESCRIPTOR Computers, Handheld IN NHSEED,HTA

OR MeSH DESCRIPTOR Cell Phones IN NHSEED,HTA

OR MeSH DESCRIPTOR Genetic Therapy EXPLODE ALL TREES IN NHSEED,HTA

OR MeSH DESCRIPTOR Molecularly Targeted Therapy EXPLODE ALL TREES IN NHSEED,HTA

OR MeSH DESCRIPTOR Pharmacokinetics EXPLODE ALL TREES IN NHSEED,HTA

OR MeSH DESCRIPTOR Fitness Trackers IN NHSEED,HTA

OR MeSH DESCRIPTOR Artificial Intelligence IN NHSEED,HTA

OR MeSH DESCRIPTOR Machine Learning IN NHSEED,HTA

OR MeSH DESCRIPTOR Algorithms IN NHSEED, HTA

OR MeSH DESCRIPTOR Pharmacogenomic Testing IN NHSEED,HTA

AND * IN NHSEED, HTA WHERE LPD FROM 01/01/2009 TO 08/07/2021


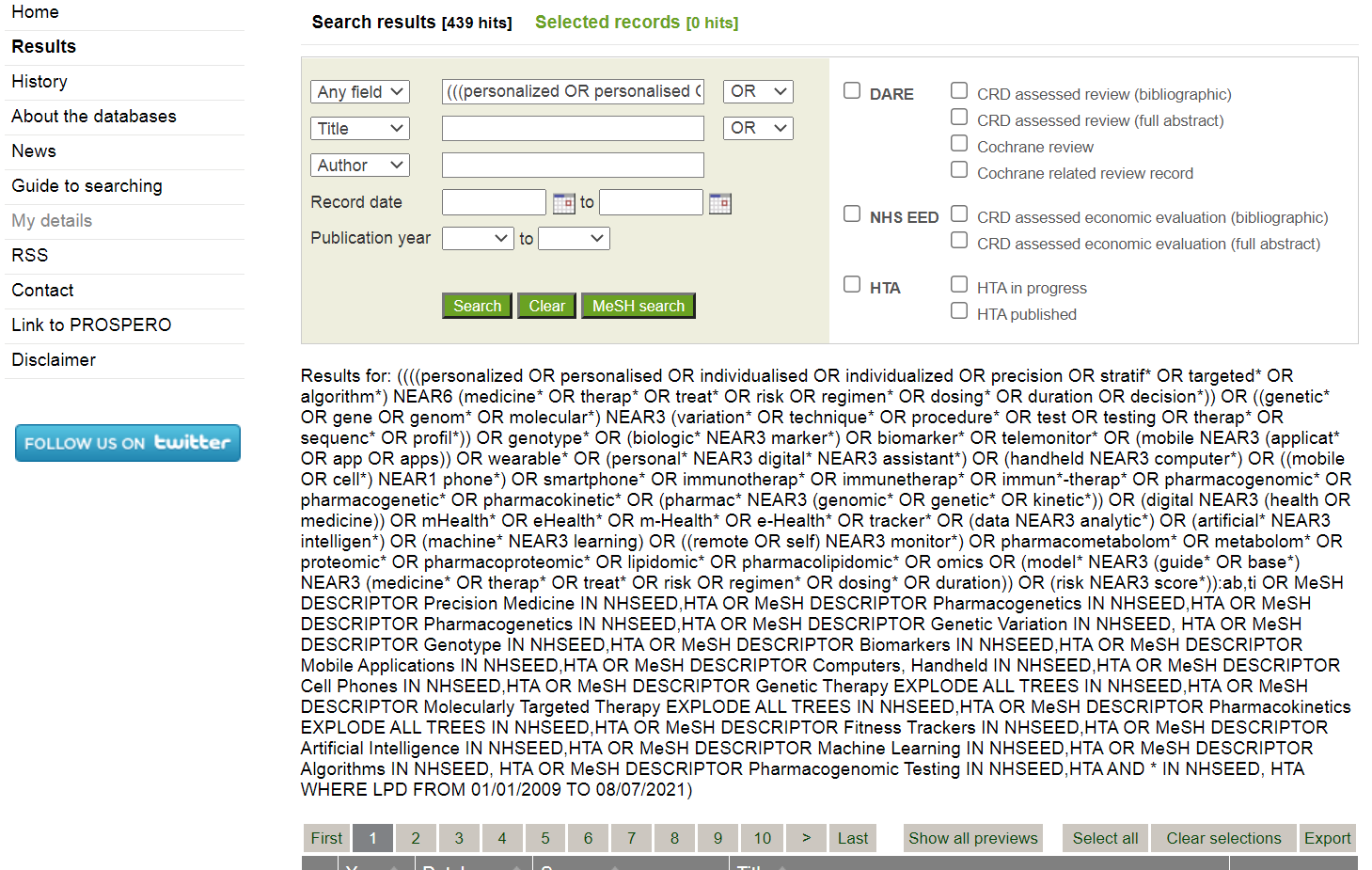


**Embase and Medline**

('biomedical technology assessment'/exp OR 'biomedical technology assessment' OR 'economic evaluation'/exp OR 'economic evaluation' OR 'quality adjusted life year'/exp OR 'quality adjusted life year' OR 'program cost effectiveness'/exp OR 'program cost effectiveness' OR ((technology NEAR/3 assessment*):ab,ti) OR ((economic* NEAR/3 (evaluat* OR value)):ab,ti) OR (((cost OR costs) NEAR/3 (benefit* OR effectiv* OR efficien* OR efficac* OR minim* OR utilit* OR consequen*)):ab,ti) OR ((qualit* NEAR/3 adjust* NEAR/3 ('life year*' OR lifeyear*)):ab,ti) OR qaly*:ab,ti)

AND ('economic model'/exp OR 'economic model' OR 'simulation'/exp OR 'simulation' OR (('model'/exp OR model) AND ('economics'/exp OR economics OR 'economic aspect'/exp OR 'economic aspect')) OR 'decision tree'/exp OR 'decision tree' OR (((model OR modeling OR modelling OR simulation* OR microsimulation*) NEAR/6 (econom* OR pharmacoeconom* OR cost OR costs)):ab,ti) OR ((decision NEAR/3 (analy* OR tree OR trees)):ab,ti) OR 'discrete event*':ab,ti OR 'state transition':ab,ti OR markov:ab,ti OR (((individual* OR 'patient level*') NEAR/3 (sampl* OR simulation*)):ab,ti) OR ((dynamic NEAR/3 transmission*):ab,ti) OR probabilistic*:ab,ti OR 'partition* survival*':ab,ti)

('personalized medicine'/exp OR 'personalized medicine' OR 'pharmacogenetics'/exp OR 'pharmacogenetics' OR 'genetic variation'/exp OR 'genetic variation' OR 'genetic procedures'/exp OR 'genetic procedures' OR 'genotype'/exp OR 'genotype' OR 'biological marker'/exp OR 'biological marker' OR 'gene therapy'/exp OR 'gene therapy' OR 'molecularly targeted therapy'/exp OR 'molecularly targeted therapy' OR 'immunotherapy'/exp OR 'immunotherapy' OR 'pharmacogenomics'/exp OR 'pharmacogenomics' OR 'pharmacokinetics'/exp OR 'pharmacokinetics' OR 'omics'/exp OR 'omics' OR 'pharmacogenetic testing'/exp OR 'pharmacogenetic testing' OR (((genetic* OR gene OR genom* OR molecular*) NEAR/3 (variation* OR technique* OR procedure* OR test OR testing OR therap* OR sequenc* OR profil*)):ab,ti) OR genotype*:ab,ti OR ((biologic* NEAR/3 marker*):ab,ti) OR biomarker*:ab,ti OR immunotherap*:ab,ti OR immunetherap*:ab,ti OR 'immun* therap*':ab,ti OR pharmacogenomic*:ab,ti OR pharmacogenetic*:ab,ti OR pharmacokinetic*:ab,ti OR ((pharmac* NEAR/3 (genomic* OR genetic* OR kinetic*)):ab,ti) OR pharmacometabolom*:ab,ti OR metabolom*:ab,ti OR proteomic*:ab,ti OR pharmacoproteomic*:ab,ti OR lipidomic*:ab,ti OR pharmacolipidomic*:ab,ti OR omics:ab,ti)

AND (2009:py OR 2010:py OR 2011:py OR 2012:py OR 2013:py OR 2014:py OR 2015:py OR 2016:py OR 2017:py OR 2018:py OR 2019:py OR 2020:py OR 2021:py)

AND ('article'/it OR 'article in press'/it OR 'review'/it)

NOT ([animals]/lim NOT [humans]/lim) NOT [conference abstract]/lim

AND [english]/lim AND ([embase]/lim OR [medline]/lim)


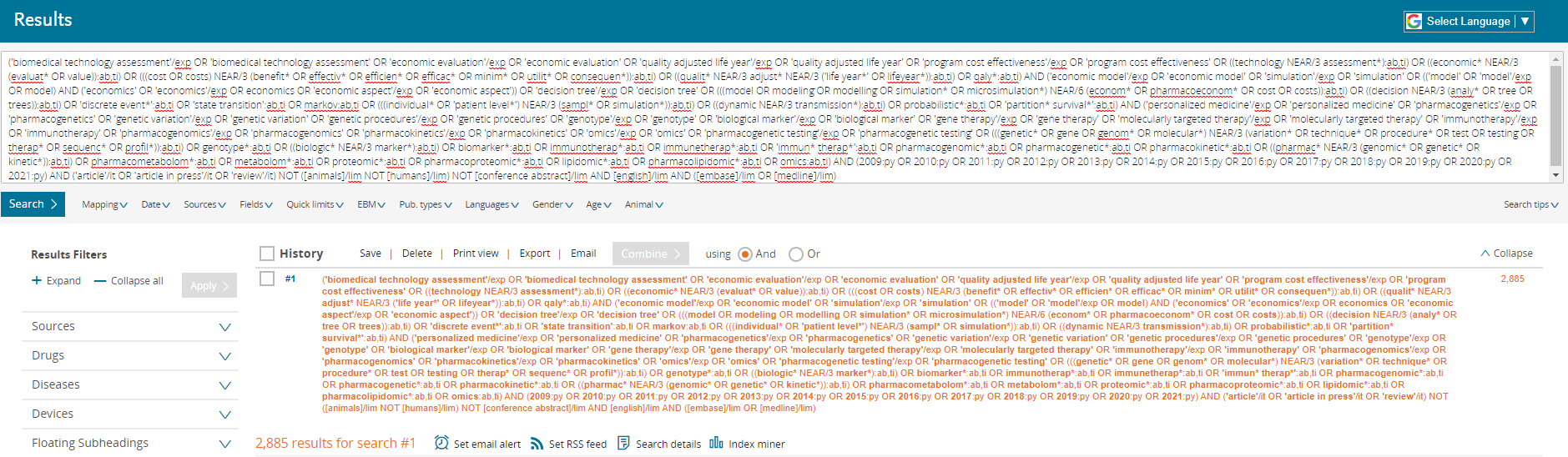


**Web of Science**

TS=((((technology NEAR/2 assessment*) OR (economic* NEAR/2 (evaluat* OR value)) OR ((cost OR costs) NEAR/2 (benefit* OR effectiv* OR efficien* OR efficac* OR minim* OR utilit* OR consequen*)) OR (qualit* NEAR/2 adjust* NEAR/2 (life-year* OR lifeyear*)) OR qaly*))

AND ((((model OR modeling OR modelling OR simulation* OR microsimulation*) NEAR/5 (econom* OR pharmacoeconom* OR cost OR costs)) OR (decision NEAR/2 (analy* OR tree OR trees)) OR discrete-event* OR "state transition" OR markov OR ((individual* OR patient-level*) NEAR/2 (sampl* OR simulation*)) OR (dynamic NEAR/2 transmission*) OR probabilistic* OR partition*-survival*))

AND ((((personalized OR personalised OR individualised OR individualized OR precision OR stratif* OR targeted* OR algorithm*) NEAR/5 (medicine* OR therap* OR treat* OR risk OR regimen* OR dosing* OR duration OR decision*)) OR ((genetic* OR gene OR genom* OR molecular*) NEAR/2 (variation* OR technique* OR procedure* OR test OR testing OR therap* OR sequenc* OR profil*)) OR genotype* OR (biologic* NEAR/2 marker*) OR biomarker* OR immunotherap* OR immunetherap* OR immun*-therap* OR pharmacogenomic* OR pharmacogenetic* OR pharmacokinetic* OR (pharmac* NEAR/2 (genomic* OR genetic* OR kinetic*)) OR (digital NEAR/2 (health OR medicine)) OR mHealth* OR eHealth* OR pharmacometabolom* OR metabolom* OR proteomic* OR pharmacoproteomic* OR lipidomic* OR pharmacolipidomic* OR omics OR (model* NEAR/2 (guide*OR base*) NEAR/2 (medicine* OR therap* OR treat* OR risk OR regimen* OR dosing* OR duration))

OR (risk NEAR/2 score*))) AND (medicine OR health* OR patient* OR hospital* OR therap* OR genetic* OR pharmac* OR virus* OR genotype* OR disease* OR diagnos* OR cancer*) NOT ((animal*OR rat OR rats OR mouse OR mice OR murine OR dog OR dogs OR canine OR cat OR cats OR feline OR rabbit OR cow OR cows OR bovine OR rodent* OR sheep OR ovine OR pig OR swine OR porcine OR veterinar* OR chick* OR zebrafish* OR baboon* OR nonhuman* OR primate* OR cattle* OR gooseOR geese OR duck OR macaque* OR avian* OR bird* OR fish*) NOT (human* OR patient* OR womenOR woman OR men OR man)))

AND ( 2011:py OR 2012:py OR 2013:py OR 2014:py OR 2015:py OR 2016:py OR 2017:py OR 2018:py OR 2019:py OR 2020:py OR 2021:py)

AND DT=(article) AND LA=(english)


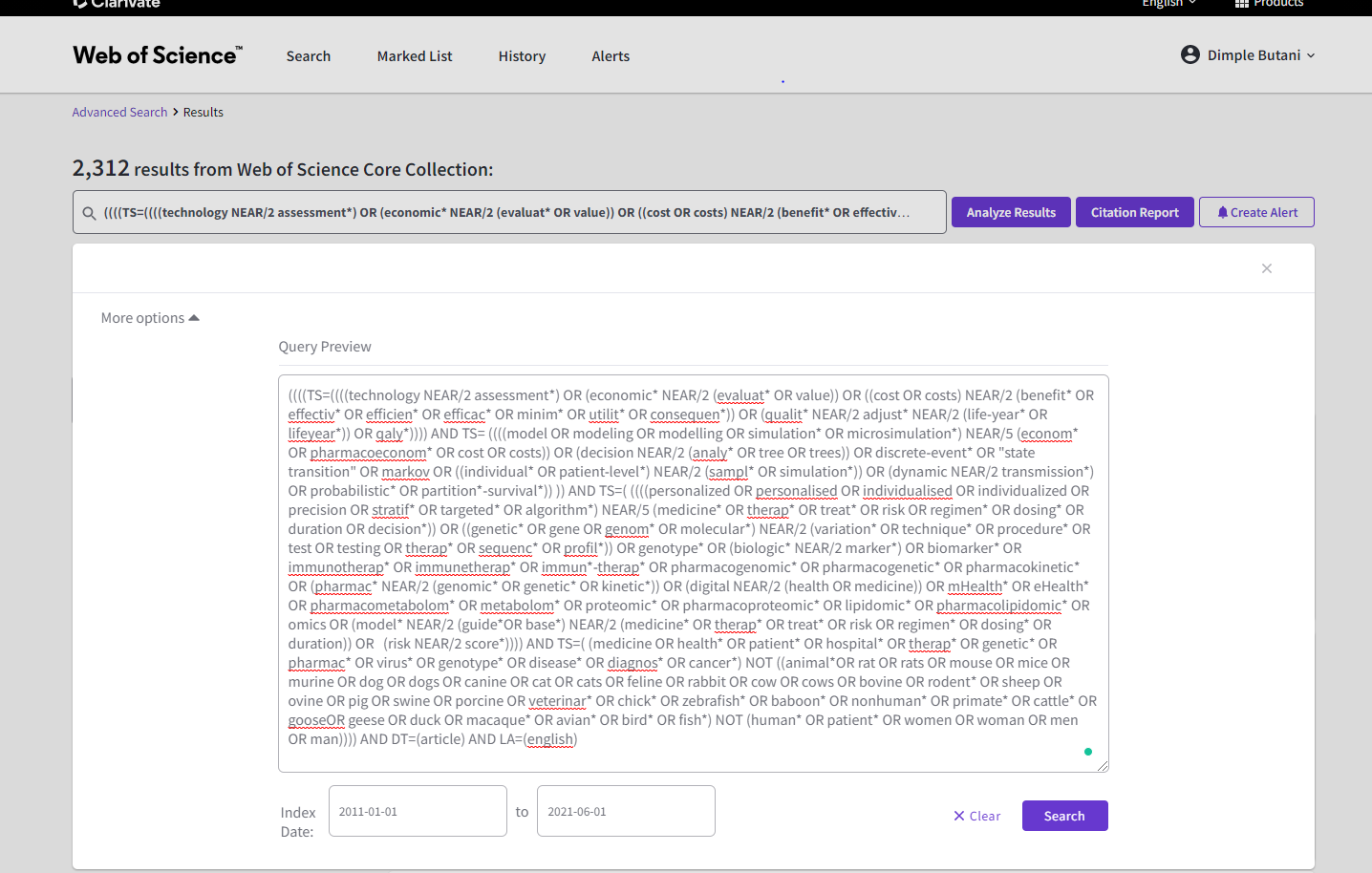

Supplement: Supplementary file 6 [file Data_Sheet_1.DOCX]
