# Supplementary material for: Mapping the value for money of precision medicine: a systematic literature review and meta-analysis
Source: Front Public Health. 2023 Nov 24;11:1151504. doi: 10.3389/fpubh.2023.1151504 (PMC10704154; doi:10.3389/fpubh.2023.1151504)
Supplement: Supplementary file 7 [file Data_Sheet_2.DOCX]

**Appendix 2. Data preparation for meta-analysis of INB**

The primary outcome, INB, is calculated as ∆E × λ − ∆C or ∆E(λ – ICER), comparing PM to conventional test/intervention. A positive INB indicates that the PM is cost-effective compared to conventional care, whereas a negative INB favors conventional care. The variance of INB is calculated as $Var\left( INB \right)=\lambda^{2}\sigma_{\Delta E}^{2}+\sigma_{\Delta c}^{2}-2\lambda Cov(\Delta E, \Delta C)\approx\lambda^{2}\sigma_{\Delta E}^{2}+\sigma_{ICER}^{2}$ . However, different EE report data differently. In the event a study reports incomplete data that are not ready for pooling, data will be simulated. Data are derived and imputed as follows.

***Scenario 1. The EE compares PM to conventional intervention strategy***

INB and its variance are calculated by directly comparing PM to conventional intervention strategy. In case of incomplete reporting: (1) if the variance of ICER is not reported, whereas variances of ∆E and ∆C are reported, Monte Carlo simulation will be performed for 1,000 replications with gamma and log-normal distributions of ∆C and normal distribution of ∆E, and the variance of ICER will be derived; (2) if the study only reports the means of ∆E but not the measure of dispersion, we will derive the variance of ∆E using Monte Carlo simulation of E from both arms for 1,000 replications with normal distribution. If E is not reported, we will take the measures of dispersion of ∆E from another study of the same disease, where the model settings (i.e., intervention, comparator, study time period, region, level of countries’ incomes, model inputs [discounting, time horizon, etc.]) are similar. Meanwhile, we will take the range of ICER from sensitivity analysis, or the 2.5^th^ and 97.5^th^ percentile of ICER from probabilistic sensitivity analysis, as a proxy of 95% CI. Finally, we will derive the variance of INB based on the variance of ∆E and ICER as abovementioned.

Of note, the monetary units of intervention and comparators are dually reported as country-specific and time-specific currencies. To standardize costing data, all INBs in terms of costs are converted to 2021 US dollar ($) adjusted with purchasing power parity (PPP), according to the consumer price index obtained from the World Bank^1^.

***Scenario 2. The EE compares PM to another PM.***

We will extract C and E from both arms, and compare both PMs to the C and E of a conventional intervention strategy from another study, where the disease is the same and study settings are similar. C and E of both PEs and the conventional intervention strategy will be separately simulated for 1,000 replications with normal distribution to estimate INB and its variance.

***Scenario 3. The EE compares PM to other new technology***

We will extract C and E from the PE arm, take the C and E of conventional intervention strategy from another study, and calculate INB as described in Scenario 2.

***Scenario 4. The EE compares PM and conventional intervention strategy to no intervention at all***

First, INB and its variance will be calculated by directing comparing intervention to no intervention. Next, based on Bucher et al.’s theory of indirect treatment effect^2^, the INB of PM versus conventional intervention strategy will be calculated as

$Indirect INB\left( \mathrm{PMvsCon} \right)=INB\left( PMvsNone \right)-INB(ConvsNone)$,

and the variance is

*V*$ar\left[ Indirect INB\left( PMvsCon \right) \right]=Var\left[ INB\left( PMvsNone \right) \right]+Var[INB\left( ConvsNone \right)]$*,* where Con refers to conventional intervention strategy.

**Reference**

1 World Bank. Inflation, consumer prices (annual %) | Data. https://data.worldbank.org/indicator/FP.CPI.TOTL.ZG (accessed Aug 3, 2021).

2 Bucher HC, Guyatt GH, Griffith LE, Walter SD. The results of direct and indirect treatment comparisons in meta-analysis of randomized controlled trials. *J Clin Epidemiol* 1997; **50**: 683–91.
