# Supplementary material for: Mapping the value for money of precision medicine: a systematic literature review and meta-analysis
Source: Front Public Health. 2023 Nov 24;11:1151504. doi: 10.3389/fpubh.2023.1151504 (PMC10704154; doi:10.3389/fpubh.2023.1151504)
Supplement: Supplementary file 8 [file Data_Sheet_3.PDF]

Appendix 1. Full List of CEAs

No.	CEA
1	AlMukdad, S.; Elewa, H.; Arafa, S.; Al-Badriyeh, D., Short- and long-term cost-effectiveness analysis of CYP2C19 genotype-guided therapy, universal clopidogrel, versus universal ticagrelor in post-percutaneous coronary intervention patients in Qatar, International Journal of Cardiology, 2021, 331, 27-34, DOI: 10.1016/j.ijcard.2021.01.044
2	Asti, L.; Hopley, C.; Avelis, C.; Bartsch, S. M.; Mueller, L. E.; Domino, M.; Cox, S. N.; Andrews, J. C.; Randall, S. L.; Stokes-Cawley, O. J.; Asjes, C.; Lee, B. Y., The Potential Clinical and Economic Value of a Human Papillomavirus Primary Screening Test That Additionally Identifies Genotypes 31, 45, 51, and 52 Individually, Sexually Transmitted Diseases, 2021, 48, 5, 370-380, DOI: 10.1097/OLQ.0000000000001327
3	Azardoost, H.; Rahimi, F.; Zeinalian, M.; Rezayatmand, R., Cost-effectiveness analysis of molecular screening to identify lynch syndrome in the patients with colorectal cancer, International Journal of Cancer Management, 2021, 14, 4, e108198, DOI: 10.5812/ijcm.108198
4	Biltaji, E.; Walker, B.; Au, T. H.; Rivers, Z.; Ose, J.; Li, C. I.; Brixner, D. I.; Stenehjem, D. D.; Ulrich, C. M., Can Cost-effectiveness Analysis Inform Genotype-Guided Aspirin Use for Primary Colorectal Cancer Prevention?, Cancer Epidemiology Biomarkers and Prevention, 2021, 30, 6, 1106-1113, DOI: 10.1158/1055-9965.EPI-19-1580
5	Bolous, N. S.; Chen, Y.; Wang, H.; Davidoff, A. M.; Devidas, M.; Jacobs, T. W.; Meagher, M. M.; Nathwani, A. C.; Neufeld, E. J.; Piras, B. A.; Rodriguez-Galindo, C.; Reiss, U. M.; Bhakta, N., The cost-effectiveness of gene therapy for severe hemophilia B: a microsimulation study from the United States Perspective, Blood, 2021, 138, 18, 1677-1690, DOI: 10.1182/blood.2021010864
6	Broekhoff, T. F.; Sweegers, C. C. G.; Krijkamp, E. M.; Mantel-Teeuwisse, A. K.; Leufkens, H. G. M.; Goettsch, W. G.; Vreman, R. A., Early Cost-Effectiveness of Onasemnogene Abeparvovec-xioi (Zolgensma) and Nusinersen (Spinraza) Treatment for Spinal Muscular Atrophy I in The Netherlands With Relapse Scenarios, Value in Health, 2021, 24, 6, 759-769, DOI: 10.1016/j.jval.2020.09.021
7	Cai, Z. L.; Cai, D.; Wang, R. W.; Wang, H.; Yu, Z.; Gao, F.; Liu, Y. S.; Kang, Y. B.; Wu, Z. M., Cost-effectiveness of CYP2C19 genotyping to guide antiplatelet therapy for acute minor stroke and high-risk transient ischemic attack, Scientific Reports, 2021, 11, 1, 7383, DOI: 10.1038/s41598-021-86824-9

8	Correa-Galendi, J. S.; Diz, M. D. E.; Stock, S.; Muller, D., Economic Modelling of Screen-and-Treat Strategies for Brazilian Women at Risk of Hereditary Breast and Ovarian Cancer, Applied Health Economics and Health Policy, 2021, 19, 1, 97-109, DOI: 10.1007/s40258-020-00599-0
9	Crawford, S. A.; Gong, C. L.; Yieh, L.; Randolph, L. M.; Hay, J. W., Diagnosing newborns with suspected mitochondrial disorders: an economic evaluation comparing early exome sequencing to current typical care, Genetics in Medicine, 2021, 23, 1854–1863, DOI: 10.1038/s41436-021-01210-0
10	Fabbri, C.; Kasper, S.; Zohar, J.; Souery, D.; Montgomery, S.; Albani, D.; Forloni, G.; Ferentinos, P.; Rujescu, D.; Mendlewicz, J.; Serretti, A.; Lewis, C. M., Cost-effectiveness of genetic and clinical predictors for choosing combined psychotherapy and pharmacotherapy in major depression, Journal of Affective Disorders, 2021, 279, 722-729, DOI: 10.1016/j.jad.2020.10.049
11	Hendrix, N.; Gulati, R.; Jiao, B.; Kader, A. K.; Ryan, S. T.; Etzioni, R., Clarifying the Trade-Offs of Risk-Stratified Screening for Prostate Cancer: A Cost-Effectiveness Study, American Journal of Epidemiology, 2021, 190, 10, 2064-2074, DOI: 10.1093/aje/kwab155
12	Jiang, W.; He, Z.; Zhang, T.; Guo, C.; Zhao, J.; Zhu, J.; Wu, J.; Yu, X.; Chen, C.; Li, J.; Jiang, J., Cost–effectiveness analysis of ribociclib plus fulvestrant for hormone receptor-positive/human EGF receptor 2-negative breast cancer, Immunotherapy, 2021, 13, 8, 661-668, DOI: 10.2217/imt-2020-0237
13	Jongeneel, G.; Greuter, M. J. E.; van Erning, F. N.; Koopman, M.; Vink, G. R.; Punt, C. J. A.; Coupe, V. M. H., Model-based effectiveness and cost-effectiveness of risk-based selection strategies for adjuvant chemotherapy in Dutch stage II colon cancer patients, Therapeutic Advances in Gastroenterology, 2021, 14, 1756284821995715, DOI: 10.1177/1756284821995715
14	Jung, Y. S.; Frisvold, D.; Dogan, T.; Dogan, M.; Philibert, R., Cost-utility analysis of an integrated genetic/epigenetic test for assessing risk for coronary heart disease, Epigenomics, 2021, 13, 7, 531-547, DOI: 10.2217/epi-2021-0021
15	Kim, J. H.; Tan, D. S. Y.; Chan, M. Y. Y., Cost-effectiveness of CYP2C19-guided antiplatelet therapy for acute coronary syndromes in Singapore, Pharmacogenomics Journal, 2021, 21, 2, 243-250, DOI: 10.1038/s41397-020-00204-6
16	Le, V.; Zhong, L.; Narsipur, N.; Hays, E.; Tran, D. K.; Rosario, K.; Wilson, L., Cost-effectiveness of ribociclib plus endocrine therapy versus placebo plus endocrine therapy in HR-positive, HER2-negative breast cancer, Journal of Managed Care and Specialty Pharmacy, 2021, 27, 3, 327-338, DOI: 10.18553/JMCP.2021.27.3.327

17	Li, W. Q.; Guo, H. F.; Li, L. Y.; Cui, J. W., Comprehensive Comparison Between Adjuvant Targeted Therapy and Chemotherapy for EGFR-Mutant NSCLC Patients: A Cost-Effectiveness Analysis, Frontiers in Oncology, 2021, 11, 619376, DOI: 10.3389/fonc.2021.619376
18	Liu, R.; Oluwole, O. O.; Diakite, I.; Botteman, M. F.; Snider, J. T.; Locke, F. L., Cost effectiveness of axicabtagene ciloleucel versus tisagenlecleucel for adult patients with relapsed or refractory large B-cell lymphoma after two or more lines of systemic therapy in the United States, Journal of Medical Economics, 2021, 24, 1, 458-468, DOI: 10.1080/13696998.2021.1901721
19	Moradi-Lakeh, M.; Yaghoubi, M.; Seitz, P.; Javanbakht, M.; Brock, E., Cost-Effectiveness of Tisagenlecleucel in Paediatric Acute Lymphoblastic Leukaemia (pALL) and Adult Diffuse Large B-Cell Lymphoma (DLBCL) in Switzerland, Advances in Therapy, 2021, 38, 6, 3427-3443, DOI: 10.1007/s12325-021-01767-x
20	Mugwagwa, T.; Abubakar, I.; White, P. J., Using molecular testing and whole-genome sequencing for tuberculosis diagnosis in a low-burden setting: a cost-effectiveness analysis using transmission-dynamic modelling, Thorax, 2021, 76, 3, 281-291, DOI: 10.1136/thoraxjnl-2019-214004
21	Su, D.; Wu, B.; Shi, L., Cost-Effectiveness of Genomic Test-Directed Olaparib for Metastatic Castration-Resistant Prostate Cancer, Frontiers in Pharmacology, 2021, 11, 610601, DOI: 10.3389/fphar.2020.610601
22	To, Y. H.; Degeling, K.; Kosmider, S.; Wong, R.; Lee, M.; Dunn, C.; Gard, G.; Jalali, A.; Wong, V.; Ijzerman, M.; Gibbs, P.; Tie, J., Circulating Tumour DNA as a Potential Cost-Effective Biomarker to Reduce Adjuvant Chemotherapy Overtreatment in Stage II Colorectal Cancer, Pharmacoeconomics, 2021, 39, 8, 953-964, DOI: 10.1007/s40273-021-01047-0
23	Wakase, S.; Teshima, T.; Zhang, J.; Ma, Q. F.; Watanabe, Y.; Yang, H. B.; Qi, C. Z.; Chai, X. L.; Xie, Y. W.; Wu, E. Q.; Igarashi, A., Cost-Effectiveness Analysis of Tisagenlecleucel for the Treatment of Pediatric and Young Adult Patients with Relapsed or Refractory B Cell Acute Lymphoblastic Leukemia in Japan, Transplantation and Cellular Therapy, 2021, 27, 3, 241.e1-241.e11, DOI: 10.1016/j.jtct.2020.12.023
24	Wakase, S.; Teshima, T.; Zhang, J.; Ma, Q.; Fujita, T.; Yang, H.; Chai, X.; Qi, C. Z.; Liu, Q.; Wu, E. Q.; Igarashi, A., Cost Effectiveness Analysis of Tisagenlecleucel for the Treatment of Adult Patients with Relapsed or Refractory Diffuse Large B Cell Lymphoma in Japan, Transplantation and Cellular Therapy, 2021, 27, 6, 506.e1-506.e10, DOI: 10.1016/j.jtct.2021.03.005
25	Wong, J. Z. Y.; Chai, J. H.; Yeoh, Y. S.; Riza, N. K. M.; Liu, J.; Teo, Y. Y.; Wee, H. L.; Hartman, M., Cost effectiveness analysis of a polygenic risk tailored breast cancer screening programme in Singapore, Bmc Health Services Research, 2021, 21, 1, 379, DOI: 10.1186/s12913-021-06396-2

26	Yeh, J. M.; Stout, N. K.; Chaudhry, A.; Christensen, K. D.; Gooch, M.; McMahon, P. M.; O'Brien, G.; Rehman, N.; Zawatsky, C. L. B.; Green, R. C.; Lu, C. Y.; Rehm, H. L.; Williams, M. S.; Diller, L.; Wu, A. C., Universal newborn genetic screening for pediatric cancer predisposition syndromes: model-based insights, Genetics in Medicine, 2021, 23, 1366–1371, DOI: 10.1038/s41436-021-01124-x
27	Yuliwulandari, R.; Shin, J. G.; Kristin, E.; Suyatna, F. D.; Prahasto, I. D.; Prayuni, K.; Mahasirimongkol, S.; Cavallari, L. H.; Mitropoulou, C.; Patrinos, G. P.; Hao, J.; Williams, M. S.; Snyder, S. R., Cost-effectiveness analysis of genotyping for HLA-B*15:02 in Indonesian patients with epilepsy using a generic model, Pharmacogenomics Journal, 2021, 21, 4, 476-483, DOI: 10.1038/s41397-021-00225-9
28	Zhu, Y.; Moriarty, J. P.; Swanson, K. M.; Takahashi, P. Y.; Bielinski, S. J.; Weinshilboum, R.; Wang, L. W.; Borah, B. J., A model-based cost-effectiveness analysis of pharmacogenomic panel testing in cardiovascular disease management: preemptive, reactive, or none?, Genetics in Medicine, 2021, 23, 3, 461-470, DOI: 10.1038/s41436-020-00995-w
29	Aguiar, P.; Roitberg, F.; Lopes, G.; del Giglio, A., Distinct models to assess the cost-effectiveness of EGFR-tyrosine kinase inhibitors for the treatment of metastatic non-small cell lung cancer in the context of the Brazilian Unified Health Care System, Jornal Brasileiro De Pneumologia, 2020, 46, 4, e20180255, DOI: 10.36416/1806-3756/e20180255
30	Alkhatib, N.; Sweitzer, N. K.; Lee, C. S.; Erstad, B.; Slack, M.; Gharaibeh, M.; Karnes, J.; Klimecki, W.; Ramos, K.; Abraham, I., Ex Ante Economic Evaluation of Arg389 Genetically Targeted Treatment with Bucindolol versus Empirical Treatment with Carvedilol in NYHA III/IV Heart Failure, American Journal of Cardiovascular Drugs, 2020, 21, 2, 205-217, DOI: 10.1007/s40256-020-00425-x
31	Banerjee, S.; Kumar, A.; Lopez, N.; Zhao, B. Q.; Tang, C. M.; Yebra, M.; Yoon, H.; Murphy, J. D.; Sicklick, J. K., Cost-effectiveness Analysis of Genetic Testing and Tailored First-Line Therapy for Patients With Metastatic Gastrointestinal Stromal Tumors, Jama Network Open, 2020, 3, 9, e2013565, DOI: 10.1001/jamanetworkopen.2020.13565
32	Cher, B. P.; Gan, K. Y.; Aziz, M. I. A.; Lin, L.; Hwang, W. Y. K.; Poon, L. M.; Ng, K., Cost utility analysis of tisagenlecleucel vs salvage chemotherapy in the treatment of relapsed/refractory diffuse large B-cell lymphoma from Singapore's healthcare system perspective, Journal of Medical Economics, 2020, 23, 11, 1321-1329, DOI: 10.1080/13696998.2020.1808981
33	Cook, K.; Forbes, S. P.; Adamski, K.; Ma, J. N. J.; Chawla, A.; Garrison, L. P., Assessing the potential cost-effectiveness of a gene therapy for the treatment of hemophilia A, Journal of Medical Economics, 2020, 23, 5, 501-512, DOI: 10.1080/13696998.2020.1721508

34	Duarte, H. A.; Babigumira, J. B.; Enns, E. A.; Stauffer, D. C.; Shafer, R. W.; Beck, I. A.; Garrison, L. P.; Chung, M. H.; Frenkel, L. M.; Bendavid, E., Cost-effectiveness analysis of pre-ART HIV drug resistance testing in Kenyan women, EClinicalMedicine, 2020, 22, 100355, DOI: 10.1016/j.eclinm.2020.100355
35	Dymond, A.; Davies, H.; Mealing, S.; Pollit, V.; Coll, F.; Brown, N. M.; Peacock, S. J., Genomic Surveillance of Methicillin-resistant Staphylococcus aureus: A Mathematical Early Modeling Study of Cost-effectiveness, Clinical Infectious Diseases, 2020, 70, 8, 1613-1619, DOI: 10.1093/cid/ciz480
36	Fawsitt, C. G.; Vickerman, P.; Cooke, G. S.; Welton, N. J.; Barnes, E.; Ball, J.; Brainard, D.; Burgess, G.; Dillon, J.; Foster, G.; Gore, C.; Guha, N.; Halford, R.; Whitby, K.; Holmes, C.; Howe, A.; Hudson, E.; Hutchinson, S.; Irving, W.; Khakoo, S.; Klenerman, P.; Martin, N.; Massetto, B.; Mbisa, T.; McHutchison, J.; McKeating, J.; McLauchlan, J.; Miners, A.; Murray, A.; Shaw, P.; Simmonds, P.; Spencer, C.; Thomson, E.; Zitzmann, N., Cost-Effectiveness Analysis of Baseline Testing for Resistance-Associated Polymorphisms to Optimize Treatment Outcome in Genotype 1 Noncirrhotic Treatment-Naive Patients With Chronic Hepatitis C Virus, Value in Health, 2020, 23, 2, 180-190, DOI: 10.1016/j.jval.2019.08.012
37	Furzer, J.; Gupta, S.; Nathan, P. C.; Schechter, T.; Pole, J. D.; Krueger, J.; Pechlivanoglou, P., Cost-effectiveness of Tisagenlecleucel vs Standard Care in High-risk Relapsed Pediatric Acute Lymphoblastic Leukemia in Canada, Jama Oncology, 2020, 6, 3, 393-401, DOI: 10.1001/jamaoncol.2019.5909
38	Guzauskas, G. F.; Garbett, S.; Zhou, Z.; Spencer, S. J.; Smith, H. S.; Hao, J.; Hassen, D.; Snyder, S. R.; Graves, J. A.; Peterson, J. F.; Williams, M. S.; Veenstra, D. L., Cost-effectiveness of Population-Wide Genomic Screening for Hereditary Breast and Ovarian Cancer in the United States, Jama Network Open, 2020, 3, 10, e2022874, DOI: 10.1001/jamanetworkopen.2020.22874
39	Hyle, E. P.; Scott, J. A.; Sax, P. E.; Millham, L. R. I.; Dugdale, C. M.; Weinstein, M. C.; Freedberg, K. A.; Walensky, R. P., Clinical Impact and Cost-effectiveness of Genotype Testing at Human Immunodeficiency Virus Diagnosis in the United States, Clinical Infectious Diseases, 2020, 70, 7, 1353-1363, DOI: 10.1093/cid/ciz372
40	Ibarrondo, O.; Alvarez-Lopez, I.; Freundlich, F.; Arrospide, A.; Galve-Calvo, E.; Gutierrez-Toribio, M.; Plazaola, A.; Mar, J., Probabilistic cost-utility analysis and expected value of perfect information for the Oncotype multigenic test: a discrete event simulation model, Gaceta Sanitaria, 2020, 34, 1, 61-68, DOI: 10.1016/j.gaceta.2018.07.012

41	Kapoor, R.; So, J. B. Y.; Zhu, F.; Too, H. P.; Yeoh, K. G.; Yoong, J. S. Y., Evaluating the Use of microRNA Blood Tests for Gastric Cancer Screening in a Stratified Population-Level Screening Program: An Early Model-Based Cost-Effectiveness Analysis, Value in Health, 2020, 23, 9, 1171-1179, DOI: 10.1016/j.jval.2020.04.1829
42	Kim, H.; Vargo, J. A.; Smith, K. J.; Beriwal, S., Cost-Effectiveness Analysis of Biological Signature DCISionRT Use for DCIS Treatment, Clinical Breast Cancer, 2020, 21, 3, E271-E278, DOI: 10.1016/j.clbc.2020.10.007
43	Krepline, A. N.; Geurts, J. L.; George, B.; Kamgar, M.; Madhavan, S.; Erickson, B. A.; Hall, W. A.; Grif, M. O.; Evans, D. B.; Tsai, S. S.; Kim, R. Y., Cost-effectiveness analysis of universal germline testing for patients with pancreatic cancer, Surgery, 2020, 169, 3, 629-635, DOI: 10.1016/j.surg.2020.06.038
44	Lauren, B.; Ostvar, S.; Silver, E.; Ingram, M.; Oh, A.; Kumble, L.; Laszkowska, M.; Chu, J. N.; Hershman, D. L.; Manji, G.; Neugut, A. I.; Hur, C., Cost-Effectiveness Analysis of Biomarker-Guided Treatment for Metastatic Gastric Cancer in the Second-Line Setting, Journal of Oncology, 2020, 2020, 2198960, DOI: 10.1155/2020/2198960
45	Limdi, N. A.; Cavallari, L. H.; Lee, C. R.; Hillegass, W. B.; Holmes, A. M.; Skaar, T. C.; Pisu, M.; Dillon, C.; Beitelshees, A. L.; Empey, P. E.; Duarte, J. D.; Diaby, V.; Gong, Y.; Johnson, J. A.; Graves, J.; Garbett, S.; Zhou, Z. L.; Peterson, J. F., Cost-effectiveness of CYP2C19-guided antiplatelet therapy in patients with acute coronary syndrome and percutaneous coronary intervention informed by real-world data, Pharmacogenomics Journal, 2020, 20, 5, 724-735, DOI: 10.1038/s41397-020-0162-5
46	Lipton, J. H.; Zargar, M.; Warner, E.; Greenblatt, E. E.; Lee, E.; Chan, K. K. W.; Wong, W. W. L., Cost effectiveness of in vitro fertilisation and preimplantation genetic testing to prevent transmission of BRCA1/2 mutations, Human Reproduction, 2020, 35, 2, 434-445, DOI: 10.1093/humrep/dez203
47	Narasimhalu, K.; Ang, Y. K.; Tan, D. S. Y.; De Silva, D. A.; Tan, K. B., Cost Effectiveness of Genotype-Guided Antiplatelet Therapy in Asian Ischemic Stroke Patients: Ticagrelor as an Alternative to Clopidogrel in Patients with CYP2C19 Loss of Function Mutations, Clinical Drug Investigation, 2020, 40, 11, 1063-1070, DOI: 10.1007/s40261-020-00970-y
48	Ontario Health (Quality), Gene Expression Profiling Tests for Early-Stage Invasive Breast Cancer: A Health Technology Assessment, Ontario Health Technology Assessment Series, 2020, 20, 10, 1-234, PMID: 32284770
49	Pruis, S. L.; Jeon, Y. K.; Pearce, F.; Thong, B. Y. H.; Aziz, M. I. A., Cost-effectiveness of sequential urate lowering therapies for the management of gout in Singapore, Journal of Medical Economics, 2020, 23, 8, 838-847, DOI: 10.1080/13696998.2020.1757456

50	Rens, N. E.; Uyl-de Groot, C. A.; Goldhaber-Fiebert, J. D.; Croda, J.; Andrews, J. R., Cost-effectiveness of a Pharmacogenomic Test for Stratified Isoniazid Dosing in Treatment of Active Tuberculosis, Clinical Infectious Diseases, 2020, 71, 12, 3136-3143, DOI: 10.1093/cid/ciz1212
51	Retel, V. P.; Byng, D.; Linn, S. C.; Jozwiak, K.; Koffijberg, H.; Rutgers, E. J.; Cardoso, F.; Piccart, M. J.; Poncet, C.; van't Veer, L. J.; van Harten, W. H., Cost-effectiveness analysis of the 70-gene signature compared with clinical assessment in breast cancer based on a randomised controlled trial, European Journal of Cancer, 2020, 137, 193-203, DOI: 10.1016/j.ejca.2020.07.002
52	Ribera Santasusana, J.M.; de Andrés Saldaña, A.; García-Muñoz, N.; Gostkorzewicz, J.; Martínez Llinàs, D.; Díaz de Heredia, C., Cost-effectiveness analysis of tisagenlecleucel in the treatment of relapsed or refractory B-cell acute lymphoblastic Leukaemia in children and young adults in Spain, ClinicoEconomics and Outcomes Research, 2020, 12, 253-264, DOI: 10.2147/CEOR.S241880
53	Snowsill, T. M.; Ryan, N. A. J.; Crosbie, E. J., Cost-Effectiveness of the Manchester Approach to Identifying Lynch Syndrome in Women with Endometrial Cancer , Journal of Clinical Medicine, 2020, 9, 6, 1664, DOI: 10.3390/jcm9061664
54	Tanner, J. A.; Davies, P. E.; Overall, C. C.; Grima, D.; Nam, J.; Dechairo, B. M., Cost–effectiveness of combinatorial pharmacogenomic testing for depression from the Canadian public payer perspective, Pharmacogenomics, 2020, 21, 8, 521-531, DOI: 10.2217/pgs-2020-0012
55	Teng, G. G.; Tan-Koi, W. C.; Dong, D.; Sung, C., Is HLA-B*58:01 genotyping cost effective in guiding allopurinol use in gout patients with chronic kidney disease?, Pharmacogenomics, 2020, 21, 4, 279-291, DOI: 10.2217/pgs-2019-0160
56	Uhrmann, M. F.; Lorenz, B.; Gissel, C., Cost Effectiveness of Voretigene Neparvovec for RPE65-Mediated Inherited Retinal Degeneration in Germany, Translational Vision Science & Technology, 2020, 9, 9, 17, DOI: 10.1167/tvst.9.9.17
57	Viriato, D.; Bennett, N.; Sidhu, R.; Hancock, E.; Lomax, H.; Trueman, D.; MacLaren, R. E., An Economic Evaluation of Voretigene Neparvovec for the Treatment of Biallelic RPE65-Mediated Inherited Retinal Dystrophies in the UK, Advances in Therapy, 2020, 37, 3, 1233-1247, DOI: 10.1007/s12325-020-01243-y
58	Wei, X. X.; Sun, H.; Zhuang, J.; Weng, X. H.; Zheng, B.; Lin, Q. W.; Zhang, G. F.; Cai, J. Q., Cost-effectiveness Analysis of CYP2D6*10 Pharmacogenetic Testing to Guide the Adjuvant Endocrine Therapy for Postmenopausal Women with Estrogen Receptor Positive Early Breast Cancer in China, Clinical Drug Investigation, 2020, 40, 1, 25-32, DOI: 10.1007/s40261-019-00842-0

59	Wherry, K.; Williamson, I.; Chapman, R. H.; Kuntz, K. M., Cost-Effectiveness of Ivacaftor Therapy for Treatment of Cystic Fibrosis Patients With the G551D Gating Mutation, Value in Health, 2020, 23, 10, 1332-1339, DOI: 10.1016/j.jval.2020.05.016
60	Wu, B.; Shi, L. Z., Frontline BRAF Testing-Guided Treatment for Advanced Melanoma in the Era of Immunotherapies: A Cost-Utility Analysis Based on Long-term Survival Data, Jama Dermatology, 2020, 156, 11, 1177-1184, DOI: 10.1001/jamadermatol.2020.2398
61	Cai, H. F.; Zhang, L. F.; Li, N.; Chen, S.; Zheng, B.; Yang, J.; Weng, L. Z.; Liu, M. B., Cost-effectiveness of Osimertinib as First-line Treatment and Sequential Therapy for EGFR Mutation-positive Non-small Cell Lung Cancer in China, Clinical Therapeutics, 2019, 41, 2, 280-290, DOI: 10.1016/j.clinthera.2018.12.007
62	Catchpool, M.; Ramchand, J.; Martyn, M.; Hare, D. L.; James, P. A.; Trainer, A. H.; Knight, J.; Goranitis, I., A cost-effectiveness model of genetic testing and periodical clinical screening for the evaluation of families with dilated cardiomyopathy, Genetics in Medicine, 2019, 21, 12, 2815-2822, DOI: 10.1038/s41436-019-0582-2
63	Chang, E. M.; Punglia, R. S.; Steinberg, M. L.; Raldow, A. C., Cost Effectiveness of the Oncotype DX Genomic Prostate Score for Guiding Treatment Decisions in Patients With Early Stage Prostate Cancer, Urology, 2019, 126, 89-95, DOI: 10.1016/j.urology.2018.12.016
64	Choi, H.; Mohit, B., Cost-effectiveness of screening for HLA-B*1502 prior to initiation of carbamazepine in epilepsy patients of Asian ancestry in the United States, Epilepsia, 2019, 60, 7, 1472-1481, DOI: 10.1111/epi.16053
65	Chugh, Y.; Dhiman, R. K.; Premkumar, M.; Prinja, S.; Grover, G. S.; Bahuguna, P., Real-world cost-effectiveness of pan- genotypic Sofosbuvir-Velpatasvir combination versus genotype dependent directly acting anti-viral drugs for treatment of hepatitis C patients in the universal coverage scheme of Punjab state in India, Plos One, 2019, 14, 8, e0221769, DOI: 10.1371/journal.pone.0221769
66	Criss, S. D.; Weaver, D. T.; Sheehan, D. F.; Lee, R. J.; Pandharipande, P. V.; Kong, C. Y., Effect of PD-L1 testing on the cost-effectiveness and budget impact of pembrolizumab for advanced urothelial carcinoma of the bladder in the United States, Urologic Oncology: Seminars and Original Investigations, 2019, 37, 3, 180.e11-180.e18, DOI: 10.1016/j.urolonc.2018.11.016
67	Dong, O. M.; Wheeler, S. B.; Cruden, G.; Lee, C. R.; Voora, D.; Dusetzina, S. B.; Wiltshire, T., Cost-Effectiveness of Multigene Pharmacogenetic Testing in Patients With Acute Coronary Syndrome After Percutaneous Coronary Intervention, Value in Health, 2019, 23, 1, 61-73, DOI: 10.1016/j.jval.2019.08.002

68	Fu, Y.; Zhang, X. Y.; Qin, S. B.; Nie, X. Y.; Shi, L. W.; Shao, H.; Liu, J., Cost-effectiveness of CYP2C19 LOF-guided antiplatelet therapy in Chinese patients with acute coronary syndrome, Pharmacogenomics, 2019, 21, 1, 33-42, DOI: 10.2217/pgs-2019-0050
69	GoodSmith, M. S.; Skandari, M. R.; Huang, E. S.; Naylor, R. N., The Impact of Biomarker Screening and Cascade Genetic Testing on the Cost-Effectiveness of MODY Genetic Testing, Diabetes Care, 2019, 42, 12, 2247-2255, DOI: 10.2337/dc19-0486
70	Hannouf, M. B.; Zaric, G. S.; Blanchette, P.; Brezden-Masley, C.; Paulden, M.; McCabe, C.; Raphael, J.; Brackstone, M., Cost-effectiveness analysis of multigene expression profiling assays to guide adjuvant therapy decisions in women with invasive early-stage breast cancer, Pharmacogenomics Journal, 2019, 20, 1, 27-46, DOI: 10.1038/s41397-019-0089-x
71	Hao, J.; Critchley-Thorne, R.; Diehl, D. L.; Snyder, S. R., A Cost-Effectiveness Analysis Of An Adenocarcinoma Risk Prediction Multi-Biomarker Assay For Patients With Barrett's Esophagus, ClinicoEconomics and Outcomes Research, 2019, 11, 623-635, DOI: 10.2147/CEOR.S221741
72	Hart, M. R.; Garrison, L. P.; Doyle, D. L.; Jarvik, G. P.; Watkins, J.; Devine, B., Projected Cost-Effectiveness for 2 Gene-Drug Pairs Using a Multigene Panel for Patients Undergoing Percutaneous Coronary Intervention, Value in Health, 2019, 22, 11, 1231-1239, DOI: 10.1016/j.jval.2019.05.015
73	Hoskins, P.; Eccleston, A.; Hurry, M.; Dyer, M., Targeted surgical prevention of epithelial ovarian cancer is cost effective and saves money in BRCA mutation carrying family members of women with epithelial ovarian cancer. A Canadian model, Gynecologic Oncology, 2019, 153, 1, 87-91, DOI: 10.1016/j.ygyno.2019.01.018
74	Johnson, S.; Buessing, M.; O'Connell, T.; Pitluck, S.; Ciulla, T. A., Cost-effectiveness of Voretigene Neparvovec-rzyl vs Standard Care for RPE65-Mediated Inherited Retinal Disease, Jama Ophthalmology, 2019, 137, 10, 1115-1123, DOI: 10.1001/jamaophthalmol.2019.2512
75	Kim, K.; Touchette, D. R.; Cavallari, L. H.; Ardati, A. K.; DiDomenico, R. J., Cost-Effectiveness of Strategies to Personalize the Selection of P2Y12 Inhibitors in Patients with Acute Coronary Syndrome, Cardiovascular Drugs and Therapy, 2019, 33, 5, 533-546, DOI: 10.1007/s10557-019-06896-8
76	Lin, J. K.; Muffly, L. S.; Spinner, M. A.; Barnes, J. I.; Owens, D. K.; Goldhaber-Fiebert, J. D., Cost effectiveness of chimeric antigen receptor T-cell therapy in multiply relapsed or refractory adult large B-cell lymphoma, Journal of Clinical Oncology, 2019, 37, 24, 2105-2119, DOI: 10.1200/JCO.18.02079

77	Loong, H. H.; Wong, C. K. H.; Leung, L. K. S.; Dhankhar, P.; Insinga, R. P.; Chandwani, S.; Hsu, D. C.; Lee, M. Y. K.; Huang, M.; Pellissier, J.; Rai, A.; Achra, M.; Tan, S. C., Cost Effectiveness of PD-L1-Based Test-and-Treat Strategy with Pembrolizumab as the First-Line Treatment for Metastatic NSCLC in Hong Kong with Pembrolizumab as the First-Line Treatment for Metastatic NSCLC in Hong Kong, PharmacoEconomics - Open, 2019, 4, 2, 235-247, DOI: 10.1007/s41669-019-00178-7
78	Moya-Alarcón, C.; González-Domínguez, A.; Simon, S.; Pérez-Román, I.; González-Martín, A.; Bayo-Lozano, E.; Sánchez-Heras, A. B., Cost–utility analysis of germline BRCA1/2 testing in women with high-grade epithelial ovarian cancer in Spain , Clinical and Translational Oncology, 2019, 21, 8, 1076-1084, DOI: 10.1007/s12094-018-02026-2
79	Müller, D.; Danner, M.; Schmutzler, R.; Engel, C.; Wassermann, K.; Stollenwerk, B.; Stock, S.; Rhiem, K., Economic modeling of risk-adapted screen-and-treat strategies in women at high risk for breast or ovarian cancer, European Journal of Health Economics, 2019, 20, 5, 739-750, DOI: 10.1007/s10198-019-01038-1
80	Özmen, V.; Çakar, B.; Gökmen, E.; Özdoğan, M.; Güler, N.; Uras, C.; Ok, E.; Demircan, O.; Işıkdoğan, A.; Saip, P., Cost effectiveness of Gene Expression Profiling in Patients with Early-Stage Breast Cancer in a Middle- Income Country, Turkey: Results of a Prospective Multicenter Study , Meme Sagligi Dergisi / Journal of Breast Health, 2019, 15, 3, 183-190, DOI: 10.5152/ejbh.2019.4761
81	Ramirez, S. P.; del Monte-Millan, M.; Lopez-Tarruella, S.; Janez, N. M.; Marquez-Rodas, I.; Samper, F. L.; Peron, Y. I.; Terres, C. R.; Rodriguez, D. R.; Garcia-Saenz, J. A.; Anton, F. M.; Aunon, P. Z.; Yustos, M. A.; Alvarez, M. A. L.; Gil, E. M. C.; Sanchez, L. M.; Gonzalez, M. J. E.; Martinez, J. A. G.; Sanchez, C. J.; Muino, C. B.; Adrian, S. G.; Galindo, J. R. C.; Maganto, V. V.; Martin, M., Prospective, multicenter study on the economic and clinical impact of gene-expression assays in early-stage breast cancer from a single region: the PREGECAM registry experience , Clinical & Translational Oncology, 2019, 22, 5, 717-724, DOI: 10.1007/s12094-019-02176-x
82	Rodríguez, C. A.; Posada, M. A.; Alconada, L. A.; Folgar, S. C.; López, R. L.; Martín-Saborido, C., Monitoring treatment response in metastasic colorectal cancer: Economic evaluation of PrediCTC versus computed tomography scan, Global and Regional Health Technology Assessment, 2019, 2019, 1-10, DOI: 10.1177/2284240319858331
83	Saito, S.; Nakazawa, K.; Nagahashi, M.; Ishikawa, T.; Akazawa, K., Cost–effectiveness of BRCA1/2 mutation profiling to target olaparib use in patients with metastatic breast cancer, Personalized Medicine, 2019, 16, 6, 439-448, DOI: 10.2217/pme-2018-0141

84	Sarkar, R. R.; Gloude, N. J.; Schiff, D.; Murphy, J. D., Cost-Effectiveness of Chimeric Antigen Receptor T-Cell Therapy in Pediatric Relapsed/Refractory B-Cell Acute Lymphoblastic Leukemia, Journal of the National Cancer Institute, 2019, 111, 7, 719-726, DOI: 10.1093/jnci/djy193
85	Sluiter, R. L.; Janzing, J. G. E.; van der Wilt, G. J.; Kievit, W.; Teichert, M., An economic model of the cost-utility of pre-emptive genetic testing to support pharmacotherapy in patients with major depression in primary care, Pharmacogenomics Journal, 2019, 19, 5, 480-489, DOI: 10.1038/s41397-019-0070-8
86	South, E.; Cox, E.; Meader, N.; Woolacott, N.; Griffin, S., Strimvelis® for Treating Severe Combined Immunodeficiency Caused by Adenosine Deaminase Deficiency: An Evidence Review Group Perspective of a NICE Highly Specialised Technology Evaluation, PharmacoEconomics - Open, 2019, 3, 2, 151-161, DOI: 10.1007/s41669-018-0102-3
87	Steuten, L.; Goulart, B.; Meropol, N. J.; Pritchard, D.; Ramsey, S. D., Cost Effectiveness of Multigene Panel Sequencing for Patients With Advanced Non–Small-Cell Lung Cancer, JCO Clinical Cancer Informatics, 2019, 3, 1-10, DOI: 10.1200/CCI.19.00002
88	Sun, L.; Brentnall, A.; Patel, S.; Buist, D. S. M.; Bowles, E. J. A.; Evans, D. G. R.; Eccles, D.; Hopper, J.; Li, S.; Southey, M.; Duffy, S.; Cuzick, J.; Silva, I. D.; Miners, A.; Sadique, Z.; Yang, L.; Legood, R.; Manchanda, R., A Cost-effectiveness Analysis of Multigene Testing for All Patients With Breast Cancer, Jama Oncology, 2019, 5, 12, 1718-1730, DOI: 10.1001/jamaoncol.2019.3323
89	Sutherland, C. S.; Ademi, Z.; Michaud, J.; Schur, N.; Lingg, M.; Bhadhuri, A.; Pache, T. D.; Bitzer, J.; Suchon, P.; Albert, V.; Hersberger, K. E.; Tanackovic, G.; Schwenkglenks, M., Economic evaluation of a novel genetic screening test for risk of venous thromboembolism compared with standard of care in women considering combined hormonal contraception in Switzerland, Bmj Open, 2019, 9, 11, e031325, DOI: 10.1136/bmjopen-2019-031325
90	Torchia, M. T.; Austin, D. C.; Kunkel, S. T.; Dwyer, K. W.; Moschetti, W. E., Next-Generation Sequencing vs Culture-Based Methods for Diagnosing Periprosthetic Joint Infection After Total Knee Arthroplasty: A Cost-Effectiveness Analysis, Journal of Arthroplasty, 2019, 34, 7, 1333-1341, DOI: 10.1016/j.arth.2019.03.029
91	Tremblay, G.; Rousseau, B.; Marquis, M.; Beaubois, C.; Sauvageau, G.; Hebert, J., Cost-Effectiveness Analysis of a HMGA2 Prognostic Test for Acute Myeloid Leukemia in a Canadian Setting, Applied Health Economics and Health Policy, 2019, 17, 6, 827-839, DOI: 10.1007/s40258-019-00503-5

92	Verhoef, L. M.; Bos, D. P. G.; van den Ende, C. H. M.; van den Hoogen, F. H. J.; Fautrel, B.; Hulscher, M. E.; Kievit, W.; den Broeder, A. A., Cost-effectiveness of five different anti-tumour necrosis factor tapering strategies in rheumatoid arthritis: a modelling study, Scandinavian Journal of Rheumatology, 2019, 48, 6, 439-447, DOI: 10.1080/03009742.2019.1613674
93	Wei, X. X.; Cai, J. Q.; Sun, H.; Li, N.; Xu, C. X.; Zhang, G. F.; Sui, Y. X.; Zhuang, J.; Zheng, B., Cost–effectiveness analysis of UGT1A1*6/*28 genotyping for preventing FOLFIRI-induced severe neutropenia in Chinese colorectal cancer patients, Pharmacogenomics, 2019, 20, 4, 241-250, DOI: 10.2217/pgs-2018-0138
94	Wei, X.; Cai, J.; Zhuang, J.; Zheng, B.; Sui, Y.; Zhang, G.; Lin, Y.; Sun, H., CYP2D6∗10 pharmacogenetic-guided SERM could be a cost-effective strategy in Chinese patients with hormone receptor-positive breast cancer, Pharmacogenomics, 2019, 21, 1, 43-53, DOI: 10.2217/pgs-2019-0073
95	Whittington, M. D.; McQueen, R. B.; Ollendorf, D. A.; Kumar, V. M.; Chapman, R. H.; Tice, J. A.; Pearson, S. D.; Campbell, J. D., Long-term Survival and Cost-effectiveness Associated With Axicabtagene Ciloleucel vs Chemotherapy for Treatment of B-Cell Lymphoma, Jama Network Open, 2019, 2, 2, e190035, DOI: 10.1001/jamanetworkopen.2019.0035
96	You, R. X.; Liu, J. Y.; Wu, D. B. C.; Qian, X. Y.; Lyu, B. X.; Zhang, Y.; Luo, N., Cost-Effectiveness Analysis Of EGFR Mutation Testing And Afatinib Versus Gemcitabine-Cisplatin As First-Line Therapy For Advanced Non-Small-Cell Lung Cancer In China, Cancer Management and Research, 2019, 11, 10239-10248, DOI: 10.2147/CMAR.S219722
97	Zanocco, K. A.; Wang, M. M.; Yeh, M. W.; Livhits, M. J., Selective use of Molecular Testing Based on Sonographic Features of Cytologically Indeterminate Thyroid Nodules: A Decision Analysis, World Journal of Surgery, 2019, 44, 2, 393-401, DOI: 10.1007/s00268-019-05177-7
98	Alkhatib, N. S.; Ramos, K.; Slack, M.; Erstad, B.; Gharaibeh, M.; Klimecki, W.; Karnes, J. H.; Sweitzer, N. K.; Abraham, I., Ex ante economic evaluation of genetic testing for the ARG389 beta1-adrenergic receptor polymorphism to support bucindolol treatment decisions in Stage III/IV heart failure, Expert Review of Precision Medicine and Drug Development, 2018, 3, 5, 319-329, DOI: 10.1080/23808993.2018.1526079
99	Balentine, C. J.; Vanness, D. J.; Schneider, D. F., Cost-effectiveness of lobectomy versus genetic testing (Afirma®) for indeterminate thyroid nodules: Considering the costs of surveillance, Surgery (United States), 2018, 163, 1, 88-96, DOI: 10.1016/j.surg.2017.10.004

100	Chandler, Y.; Schechter, C. B.; Jayasekera, J.; Near, A.; O'Neill, S. C.; Isaacs, C.; Phelps, C. E.; Ray, G. T.; Lieu, T. A.; Ramsey, S.; Mandelblatt, J. S., Cost Effectiveness of Gene Expression Profile Testing in Community Practice, Journal of Clinical Oncology, 2018, 36, 6, 554-562, DOI: 10.1200/JCO.2017.74.5034
101	Chen, Q. S.; Staton, A. D.; Ayer, T.; Goldstein, D. A.; Koff, J. L.; Flowers, C. R., Exploring the potential cost-effectiveness of precision medicine treatment strategies for diffuse large B-cell lymphoma, Leukemia & Lymphoma, 2018, 59, 7, 1700-1709, DOI: 10.1080/10428194.2017.1390230
102	Chong, H. Y.; Lim, Y. H.; Prawjaeng, J.; Tassaneeyakul, W.; Mohamed, Z.; Chaiyakunapruk, N., Cost-effectiveness analysis of HLA-B*58:01 genetic testing before initiation of allopurinol therapy to prevent allopurinol-induced Stevens-Johnson syndrome/toxic epidermal necrolysis in a Malaysian population, Pharmacogenetics and Genomics, 2018, 28, 2, 56-67, DOI: 10.1097/FPC.0000000000000319
103	Goel, A.; Chen, Q. S.; Chhatwal, J.; Aggarwal, R., Cost-effectiveness of generic pan-genotypic sofosbuvir/velpatasvir versus genotype-dependent directacting antivirals for hepatitis C treatment, Journal of Gastroenterology and Hepatology, 2018, 33, 12, 2029-2036, DOI: 10.1111/jgh.14301
104	Groessl, E. J.; Tally, S. R.; Hillery, N.; Maciel, A.; Garces, J. A., Cost-Effectiveness of a Pharmacogenetic Test to Guide Treatment for Major Depressive Disorder, Journal of Managed Care & Specialty Pharmacy, 2018, 24, 8, 726-734, DOI: 10.18553/jmcp.2018.24.8.726
105	Harty, G.; Jarrett, J.; Jofre-Bonet, M., Consequences of Biomarker Analysis on the Cost-Effectiveness of Cetuximab in Combination with FOLFIRI as a First-Line Treatment of Metastatic Colorectal Cancer: Personalised Medicine at Work, Applied Health Economics and Health Policy, 2018, 16, 4, 515-525, DOI: 10.1007/s40258-018-0395-5
106	Kwon, J. S.; Tinker, A. V.; Hanley, G. E.; Pansegrau, G.; Sun, S.; Carey, M. S.; Schrader, I., BRCA mutation testing for first-degree relatives of women with high-grade serous ovarian cancer, Gynecologic Oncology, 2018, 152, 3, 459-464, DOI: 10.1016/j.ygyno.2018.10.014
107	Lin, J. K.; Lerman, B. J.; Barnes, J. I.; Boursiquot, B. C.; Tan, Y. J.; Robinson, A. Q. L.; Davis, K. L.; Owens, D. K.; Goldhaber-Fiebert, J. D., Cost Effectiveness of Chimeric Antigen Receptor T-Cell Therapy in Relapsed or Refractory Pediatric B-Cell Acute Lymphoblastic Leukemia, Journal of Clinical Oncology, 2018, 36, 32, 3192-3202, DOI: 10.1200/JCO.2018.79.0642

108	Lotan, Y.; Woldu, S. L.; Sanli, O.; Black, P.; Milowsky, M. I., Modelling cost-effectiveness of a biomarker-based approach to neoadjuvant chemotherapy for muscle-invasive bladder cancer, Bju International, 2018, 122, 3, 434-440, DOI: 10.1111/bju.14220
109	Lu, S.; Yu, Y. F.; Fu, S. J.; Ren, H. Y., Cost-effectiveness of ALK testing and first-line crizotinib therapy for non-small-cell lung cancer in China, Plos One, 2018, 13, 10, e0205827, DOI: 10.1371/journal.pone.0205827
110	Machin, N.; Ragni, M. V.; Smith, K. J., Gene therapy in hemophilia A: a cost-effectiveness analysis, Blood Advances, 2018, 2, 14, 1792-1798, DOI: 10.1182/bloodadvances.2018021345
111	McKay, A. J.; Hogan, H.; Humphries, S. E.; Marks, D.; Ray, K. K.; Miners, A., Universal screening at age 1–2 years as an adjunct to cascade testing for familial hypercholesterolaemia in the UK: A cost-utility analysis, Atherosclerosis, 2018, 275, 434-443, DOI: 10.1016/j.atherosclerosis.2018.05.047
112	Okere, A. N.; Ezendu, K.; Berthe, A.; Diaby, V., An Evaluation of the Cost-effectiveness of Comprehensive MTM Integrated with Point-of-Care Phenotypic and Genetic Testing for US Elderly Patients After Percutaneous Coronary Intervention, Journal of Managed Care & Specialty Pharmacy, 2018, 24, 2, 142-152, DOI: 10.18553/jmcp.2018.24.2.142
113	Pashayan, N.; Morris, S.; Gilbert, F. J.; Pharoah, P. D. P., cost-effectiveness and benefit-to-harm ratio of risk-stratified screening for breast cancer a life-table model, Jama Oncology, 2018, 4, 11, 1504-1510, DOI: 10.1001/jamaoncol.2018.1901
114	Pelczarska, A.; Jakubczyk, M.; Jakubiak-Lasocka, J.; Banach, M.; Mysliwiec, M.; Gruchala, M.; Niewada, M., The cost-effectiveness of screening strategies for familial hypercholesterolaemia in Poland, Atherosclerosis, 2018, 270, 132-138, DOI: 10.1016/j.atherosclerosis.2018.01.036
115	Retel, V. P.; Steuten, L. M. G.; Foppen, M. H. G.; Mewes, J. C.; Lindenberg, M. A.; Haanen, Jbag; van Harten, W. H., Early cost-effectiveness of tumor infiltrating lymphocytes (TIL) for second line treatment in advanced melanoma: a model-based economic evaluation, Bmc Cancer, 2018, 18, 895, DOI: 10.1186/s12885-018-4788-5
116	Saramago, P.; Yang, H.; Llewellyn, A.; Palmer, S.; Simmonds, M.; Griffin, S., High-throughput, non-invasive prenatal testing for fetal Rhesus D genotype to guide antenatal prophylaxis with anti-D immunoglobulin: a cost-effectiveness analysis, Bjog-an International Journal of Obstetrics and Gynaecology, 2018, 125, 11, 1414-1422, DOI: 10.1111/1471-0528.15152

117	Sathianathen, N. J.; Kuntz, K. M.; Alarid-Escudero, F.; Lawrentschuk, N. L.; Bolton, D. M.; Murphy, D. G.; Weight, C. J.; Konety, B. R., Incorporating Biomarkers into the Primary Prostate Biopsy Setting: A Cost-Effectiveness Analysis, Journal of Urology, 2018, 200, 6, 1215-1220, DOI: 10.1016/j.juro.2018.06.016
118	Sluiter, R. L.; Kievit, W.; van der Wilt, G. J.; Schene, A. H.; Teichert, M.; Coenen, M. J. H.; Schellekens, A., Cost-Effectiveness Analysis of Genotype-Guided Treatment Allocation in Patients with Alcohol Use Disorders Using Naltrexone or Acamprosate, Using a Modeling Approach, European Addiction Research, 2018, 24, 5, 245-254, DOI: 10.1159/000494127
119	Wang, Y.; Yan, B. P.; Liew, D.; Lee, V. W. Y., Cost-effectiveness of cytochrome P450 2C19*2 genotype-guided selection of clopidogrel or ticagrelor in Chinese patients with acute coronary syndrome, Pharmacogenomics Journal, 2018, 18, 1, 113-120, DOI: 10.1038/tpj.2016.94
120	Whittington, M. D.; McQueen, R. B.; Ollendorf, D. A.; Kumar, V. M.; Chapman, R. H.; Tice, J. A.; Pearson, S. D.; Campbell, J. D., Long-term Survival and Value of Chimeric Antigen Receptor T-Cell Therapy for Pediatric Patients with Relapsed or Refractory Leukemia, JAMA Pediatrics, 2018, 172, 12, 1161-1168, DOI: 10.1001/jamapediatrics.2018.2530
121	Wu, B.; Gu, X. H.; Zhang, Q., Cost-Effectiveness of Osimertinib for EGFR Mutation-Positive Non-Small Cell Lung Cancer after Progression following First-Line EGFR TKI Therapy, Journal of Thoracic Oncology, 2018, 13, 2, 184-193, DOI: 10.1016/j.jtho.2017.10.012
122	Zargar, M.; McFarlane, T.; Chan, K. K. W.; Wong, W. W. L., Cost-Effectiveness of Nivolumab in Recurrent Metastatic Head and Neck Squamous Cell Carcinoma, Oncologist, 2018, 23, 2, 225-233, DOI: 10.1634/theoncologist.2017-0277
123	Zimmermann, M.; Lubinga, S. J.; Banken, R.; Rind, D.; Cramer, G.; Synnott, P. G.; Chapman, R. H.; Khan, S.; Carlson, J., Cost Utility of Voretigene Neparvovec for Biallelic RPE65-Mediated Inherited Retinal Disease, Value in Health, 2018, 22, 2, 161-167, DOI: 10.1016/j.jval.2018.09.2841
124	Aguiar, P. N.; Perry, L. A.; Penny-Dimri, J.; Babiker, H.; Tadokoro, H.; de Mello, R. A.; Lopes, G. L., The effect of PD-L1 testing on the cost-effectiveness and economic impact of immune checkpoint inhibitors for the second-line treatment of NSCLC, Annals of Oncology, 2017, 28, 9, 2256-2263, DOI: 10.1093/annonc/mdx305
125	Buchanan, J.; Wordsworth, S.; Clifford, R.; Robbe, P.; Taylor, J. C.; Schuh, A.; Knight, S. J. L., Using Genomic Information to Guide Ibrutinib Treatment Decisions in Chronic Lymphocytic Leukaemia: A Cost-Effectiveness Analysis, Pharmacoeconomics, 2017, 35, 8, 845-858, DOI: 10.1007/s40273-017-0519-z

126	de Graaff, B.; Neil, A.; Si, L.; Yee, K. C.; Sanderson, K.; Gurrin, L.; Palmer, A. J., Cost-Effectiveness of Different Population Screening Strategies for Hereditary Haemochromatosis in Australia, Applied Health Economics and Health Policy, 2017, 15, 4, 521-534, DOI: 10.1007/s40258-016-0297-3
127	Doble, B.; John, T.; Thomas, D.; Fellowes, A.; Fox, S.; Lorgelly, P., Cost-effectiveness of precision medicine in the fourth-line treatment of metastatic lung adenocarcinoma: An early decision analytic model of multiplex targeted sequencing, Lung Cancer, 2017, 107, 22-35, DOI: 10.1016/j.lungcan.2016.05.024
128	Eccleston, A.; Bentley, A.; Dyer, M.; Strydom, A.; Vereecken, W.; George, A.; Rahman, N., A Cost-Effectiveness Evaluation of Germline BRCA1 and BRCA2 Testing in UK Women with Ovarian Cancer, Value in Health, 2017, 20, 4, 567-576, DOI: 10.1016/j.jval.2017.01.004
129	Feller-Kopman, D.; Liu, S.; Geisler, B. P.; DeCamp, M. M.; Pietzsch, J. B., Cost-Effectiveness of a Bronchial Genomic Classifier for the Diagnostic Evaluation of Lung Cancer, Journal of Thoracic Oncology, 2017, 12, 8, 1223-1232, DOI: 10.1016/j.jtho.2017.04.030
130	Gray, E.; Donten, A.; Karssemeijer, N.; van Gils, C.; Evans, D. G.; Astley, S.; Payne, K., Evaluation of a Stratified National Breast Screening Program in the United Kingdom: An Early Model-Based Cost-Effectiveness Analysis, Value in Health, 2017, 20, 8, 1100-1109, DOI: 10.1016/j.jval.2017.04.012
131	Jahn, B.; Rochau, U.; Kurzthaler, C.; Hubalek, M.; Miksad, R.; Sroczynski, G.; Paulden, M.; Bundo, M.; Stenehjem, D.; Brixner, D.; Krahn, M.; Siebert, U., Personalized treatment of women with early breast cancer: a risk-group specific cost-effectiveness analysis of adjuvant chemotherapy accounting for companion prognostic tests OncotypeDX and Adjuvant!Online, Bmc Cancer, 2017, 17, 1, 685, DOI: 10.1186/s12885-017-3603-z
132	Jiang, M. H.; You, J. H. S., CYP2C19 LOF and GOF-Guided Antiplatelet Therapy in Patients with Acute Coronary Syndrome: A Cost-Effectiveness Analysis, Cardiovascular Drugs and Therapy, 2017, 31, 1, 39-49, DOI: 10.1007/s10557-016-6705-y
133	Ke, C. H.; Chung, W. H.; Wen, Y. H.; Huang, Y. B.; Chuang, H. Y.; Tain, Y. L.; Wang, Y. C. L.; Wu, C. C.; Hsu, C. N., Cost-effectiveness Analysis for Genotyping before Allopurinol Treatment to Prevent Severe Cutaneous Adverse Drug Reactions, Journal of Rheumatology, 2017, 44, 6, 835-843, DOI: 10.3899/jrheum.151476
134	Kim, D. J.; Kim, H. S.; Oh, M.; Kim, E. Y.; Shin, J. G., Cost Effectiveness of Genotype-Guided Warfarin Dosing in Patients with Mechanical Heart Valve Replacement Under the Fee-for-Service System , Applied Health Economics and Health Policy, 2017, 15, 5, 657-667, DOI: 10.1007/s40258-017-0317-y

135	Lázaro, P.; Pérez de Isla, L.; Watts, G. F.; Alonso, R.; Norman, R.; Muñiz, O.; Fuentes, F.; Mata, N.; López-Miranda, J.; González-Juanatey, J. R.; Díaz-Díaz, J. L.; Blasco, A. J.; Mata, P., Cost-effectiveness of a cascade screening program for the early detection of familial hypercholesterolemia, Journal of Clinical Lipidology, 2017, 11, 1, 260-271, DOI: 10.1016/j.jacl.2017.01.002
136	Li, Y. H.; Arellano, A. R.; Bare, L. A.; Bender, R. A.; Strom, C. M.; Devlin, J. J., A Multigene Test Could Cost-Effectively Help Extend Life Expectancy for Women at Risk of Hereditary Breast Cancer, Value in Health, 2017, 20, 4, 547-555, DOI: 10.1016/j.jval.2017.01.006
137	Lobo, J. M.; Trifiletti, D. M.; Sturz, V. N.; Dicker, A. P.; Buerki, C.; Davicioni, E.; Cooperberg, M. R.; Karnes, R. J.; Jenkins, R. B.; Den, R. B.; Showalter, T. N., Cost-effectiveness of the Decipher Genomic Classifier to Guide Individualized Decisions for Early Radiation Therapy After Prostatectomy for Prostate Cancer, Clinical Genitourinary Cancer, 2017, 15, 3, E299-E309, DOI: 10.1016/j.clgc.2016.08.012
138	Lu, S.; Ye, M.; Ding, L. M.; Tan, F. L.; Fu, J.; Wu, B., Cost-effectiveness of gefitinib, icotinib, and pemetrexed-based chemotherapy as first-line treatments for advanced non-small cell lung cancer in China, Oncotarget, 2017, 8, 6, 9996-10006, DOI: 10.18632/oncotarget.14310
139	Martes-Martinez, C.; Mendez-Sepulveda, C.; Millan-Molina, J.; French-Kim, M.; Marin-Centeno, H.; Rivera-Miranda, G. C.; Hernandez-Munoz, J. J.; Duconge-Soler, J., Cost-Utility Study of Warfarin Genotyping in the VACHS Affiliated Anticoagulation Clinic of Puerto Rico, Puerto Rico Health Sciences Journal, 2017, 36, 3, 165-172, PMID: 28915306
140	Najafzadeh, M.; Garces, J. A.; Maciel, A., Economic Evaluation of Implementing a Novel Pharmacogenomic Test (IDgenetix ®) to Guide Treatment of Patients with Depression and/or Anxiety , Pharmacoeconomics, 2017, 35, 12, 1297-1310, DOI: 10.1007/s40273-017-0587-0
141	Nguyen, H. V.; Finkelstein, E. A.; Mital, S.; Gardner, D. S. L., Incremental cost-effectiveness of algorithm-driven genetic testing versus no testing for Maturity Onset Diabetes of the Young (MODY) in Singapore, Journal of Medical Genetics, 2017, 54, 11, 747-753, DOI: 10.1136/jmedgenet-2017-104670
142	Plumpton, C. O.; Alfirevic, A.; Pirmohamed, M.; Hughes, D. A., Cost effectiveness analysis of HLA-B*58:01 genotyping prior to initiation of allopurinol for gout, Rheumatology, 2017, 56, 10, 1729-1739, DOI: 10.1093/rheumatology/kex253

143	Saito, S.; Kameyama, H.; Muneoka, Y.; Okuda, S.; Wakai, T.; Akazawa, K., Cost-effectiveness analysis of the use of comprehensive molecular profiling before initiating monoclonal antibody therapy against metastatic colorectal cancer in Japan, Journal of Cancer Policy, 2017, 12, 61-66, DOI: 10.1016/j.jcpo.2017.03.008
144	Walter, E.; Dellago, H.; Grillari, J.; Dimai, H. P.; Hack, M., Cost-utility analysis of fracture risk assessment using microRNAs compared with standard tools and no monitoring in the Austrian female population, Bone, 2017, 108, 44-54, DOI: 10.1016/j.bone.2017.12.017
145	Ward, M. C.; Shah, C.; Adelstein, D. J.; Geiger, J. L.; Miller, J. A.; Koyfman, S. A.; Singer, M. E., Cost-effectiveness of nivolumab for recurrent or metastatic head and neck cancer, Oral Oncology, 2017, 74, 49-55, DOI: 10.1016/j.oraloncology.2017.09.017
146	Alagoz, O.; Durham, D.; Kasirajan, K., Cost-effectiveness of one-time genetic testing to minimize lifetime adverse drug reactions, Pharmacogenomics Journal, 2016, 16, 2, 129-136, DOI: 10.1038/tpj.2015.39
147	Berm, E. J. J.; Gout-Zwart, J. J.; Luttjeboer, J.; Wilffert, B.; Postma, M. J., A Model Based Cost-Effectiveness Analysis of Routine Genotyping for CYP2D6 among Older, Depressed Inpatients Starting Nortriptyline Pharmacotherapy, Plos One, 2016, 11, 12, e0169065, DOI: 10.1371/journal.pone.0169065
148	Chen, Y. E.; Kao, S. S.; Chung, R. H., Cost-Effectiveness Analysis of Different Genetic Testing Strategies for Lynch Syndrome in Taiwan, Plos One, 2016, 11, 8, e0160599, DOI: 10.1371/journal.pone.0160599
149	Chen, Z. B.; Liew, D.; Kwan, P., Real-world cost-effectiveness of pharmacogenetic screening for epilepsy treatment, Neurology, 2016, 86, 12, 1086-1094, DOI: 10.1212/WNL.0000000000002484
150	Cressman, S.; Karsan, A.; Hogge, D. E.; McPherson, E.; Bolbocean, C.; Regier, D. A.; Peacock, S. J., Economic impact of genomic diagnostics for intermediate-risk acute myeloid leukaemia, British Journal of Haematology, 2016, 174, 4, 526-535, DOI: 10.1111/bjh.14076
151	Cromwell, I.; Regier, D. A.; Peacock, S. J.; Poh, C. F., Cost-Effectiveness Analysis of Using Loss of Heterozygosity to Manage Premalignant Oral Dysplasia in British Columbia, Canada, Oncologist, 2016, 21, 9, 1099-1106, DOI: 10.1634/theoncologist.2015-0433
152	Das, A.; Callenberg, K. M.; Styn, M. A.; Jackson, S. A., Endoscopic ablation is a cost-effective cancer preventative therapy in patients with Barrett's esophagus who have elevated genomic instability, Endoscopy International Open, 2016, 4, 5, E549-E559, DOI: 10.1055/s-0042-103415

153	Ding, Y.; Thompson, J. D.; Kobrynski, L.; Ojodu, J.; Zarbalian, G.; Grosse, S. D., Cost-Effectiveness/Cost-Benefit Analysis of Newborn Screening for Severe Combined Immune Deficiency in Washington State, Journal of Pediatrics, 2016, 172, 127-135, DOI: 10.1016/j.jpeds.2016.01.029
154	Elbasha, E. H.; Robertson, M. N.; Nwankwo, C., The cost-effectiveness of testing for NS5a resistance-associated polymorphisms at baseline in genotype 1a-infected (treatment-naïve and treatment-experienced) subjects treated with all-oral elbasvir/grazoprevir regimens in the United States, Alimentary Pharmacology & Therapeutics, 2016, 45, 3, 455-467, DOI: 10.1111/apt.13882
155	Felix, J. C.; Lacey, M. J.; Miller, J. D.; Lenhart, G. M.; Spitzer, M.; Kulkarni, R., The Clinical and Economic Benefits of Co-Testing Versus Primary HPV Testing for Cervical Cancer Screening: A Modeling Analysis, Journal of Women's Health, 2016, 25, 6, 606-616, DOI: 10.1089/jwh.2015.5708
156	Forde, G. K.; Hornberger, J.; Michalopoulos, S.; Bristow, R. E., Cost-effectiveness analysis of a multivariate index assay compared to modified American College of Obstetricians and Gynecologists criteria and CA-125 in the triage of women with adnexal masses , Current Medical Research and Opinion, 2016, 32, 2, 321-329, DOI: 10.1185/03007995.2015.1123679
157	Hannouf, M. B.; Winquist, E.; Mahmud, S. M.; Brackstone, M.; Sarma, S.; Rodrigues, G.; Rogan, P.; Hoch, J. S.; Zaric, G. S., Cost-effectiveness of using a gene expression profiling test to aid in identifying the primary tumour in patients with cancer of unknown primary, Pharmacogenomics Journal, 2016, 17, 3, 286-300, DOI: 10.1038/tpj.2015.94
158	Jiang, M. H.; You, J. H. S., Cost-effectiveness analysis of personalized antiplatelet therapy in patients with acute coronary syndrome, Pharmacogenomics, 2016, 17, 7, 701-713, DOI: 10.2217/pgs-2016-0008
159	Lim, E. A.; Lee, H.; Bae, E.; Lim, J.; Shin, Y. K.; Choi, S. E., Economic Evaluation of Companion Diagnostic Testing for EGFR Mutations and First-Line Targeted Therapy in Advanced Non-Small Cell Lung Cancer Patients in South Korea, Plos One, 2016, 11, 8, e0160155, DOI: 10.1371/journal.pone.0160155
160	Lu, S.; Zhang, J.; Ye, M.; Wang, B. A.; Wu, B., Economic analysis of ALK testing and crizotinib therapy for advanced non-small-cell lung cancer, Pharmacogenomics, 2016, 17, 9, 985-994, DOI: 10.2217/pgs-2016-0017
161	Marguet, S.; Mazouni, C.; Ramaekers, B. L. T.; Dunant, A.; Kates, R.; Jacobs, V. R.; Joore, M. A.; Harbeck, N.; Bonastre, J., European cost-effectiveness study of uPA/PAI-1 biomarkers to guide adjuvant chemotherapy decisions in breast cancer, European Journal of Cancer, 2016, 63, 168-179, DOI: 10.1016/j.ejca.2016.05.013

162	Pedersen, K.; Burger, E. A.; Sy, S.; Kristiansen, I. S.; Kim, J. J., Cost-effective management of women with minor cervical lesions: Revisiting the application of HPV DNA testing, Gynecologic Oncology, 2016, 143, 2, 326-333, DOI: 10.1016/j.ygyno.2016.08.231
163	Verhoef, T. I.; Redekop, W. K.; Langenskiold, S.; Kamali, F.; Wadelius, M.; Burnside, G.; Maitland-van der Zee, A. H.; Hughes, D. A.; Pirmohamed, M., Cost-effectiveness of pharmacogenetic-guided dosing of warfarin in the United Kingdom and Sweden, Pharmacogenomics Journal, 2016, 16, 5, 478-484, DOI: 10.1038/tpj.2016.41
164	Wallbillich, J. J.; Forde, B.; Havrilesky, L. J.; Cohn, D. E., A personalized paradigm in the treatment of platinum-resistant ovarian cancer – A cost utility analysis of genomic-based versus cytotoxic therapy., Gynecologic Oncology, 2016, 142, 1, 144-149, DOI: 10.1016/j.ygyno.2016.04.024
165	Wu, A. C.; Gay, C.; Rett, M. D.; Stout, N.; Weiss, S. T.; Fuhlbrigge, A. L., Pharmacogenomic test that predicts to inhaled corticosteroids in adults with asthma likely to be cost-saving, Pharmacogenomics, 2016, 16, 6, 591-600, DOI: 10.2217/pgs.15.28
166	Wu, J. X.; Lam, R.; Levin, M.; Rao, J. Y.; Sullivan, P. S.; Yeh, M. W., Effect of malignancy rates on cost-effectiveness of routine gene expression classifier testing for indeterminate thyroid nodules, Surgery, 2016, 159, 1, 118-126, DOI: 10.1016/j.surg.2015.05.035
167	Zhao, Y. J.; Khoo, A. L.; Lin, L.; Teng, M.; Koh, C. J.; Lim, S. G.; Lim, B. P.; Dan, Y. Y., Cost-effectiveness of strategy-based approach to treatment of genotype 1 chronic hepatitis C, Journal of Gastroenterology and Hepatology, 2016, 31, 9, 1628-1637, DOI: 10.1111/jgh.13341
168	Bargallo-Rocha, J. E.; Lara-Medina, F.; Perez-Sanchez, V.; Vazquez-Romo, R.; Villarreal-Garza, C.; Martinez-Said, H.; Shaw-Dulin, R. J.; Mohar-Betancourt, A.; Hunt, B.; Plun-Favreau, J.; Valentine, W. J., Cost-Effectiveness of the 21-Gene Breast Cancer Assay in Mexico, Advances in Therapy, 2015, 32, 3, 239-253, DOI: 10.1007/s12325-015-0190-8
169	Blank, P. R.; Filipits, M.; Dubsky, P.; Gutzwiller, F.; Lux, M. P.; Brase, J. C.; Weber, K. E.; Rudas, M.; Greil, R.; Loibl, S.; Szucs, T. D.; Kronenwett, R.; Schwenkglenks, M.; Gnant, M., Cost-Effectiveness Analysis of Prognostic Gene Expression Signature-Based Stratification of Early Breast Cancer Patients, Pharmacoeconomics, 2015, 33, 2, 179-190, DOI: 10.1007/s40273-014-0227-x
170	Bock, J. A.; Fairley, K. J.; Smith, R. E.; Maeng, D. D.; Pitcavage, J. M.; Inverso, N. A.; Williams, M. S., Cost-Effectiveness of IL28B Genotype-Guided Protease Inhibitor Triple Therapy versus Standard of Care Treatment in Patients with Hepatitis C Genotypes 2 or 3 Infection, Public Health Genomics, 2015, 17, 5-6, 306-319, DOI: 10.1159/000365939

171	Butzke, B.; Oduncu, F. S.; Severin, F.; Pfeufer, A.; Heinemann, V.; Giessen-Jung, C.; Stollenwerk, B.; Rogowski, W. H., The cost-effectiveness of UGT1A1 genotyping before colorectal cancer treatment with irinotecan from the perspective of the German statutory health insurance, Acta Oncologica, 2015, 55, 3, 318-328, DOI: 10.3109/0284186X.2015.1053983
172	Chen, C. X.; Hay, J. W., Cost-effectiveness analysis of alternative screening and treatment strategies for heterozygous familial hypercholesterolemia in the United States, International Journal of Cardiology, 2015, 181, 417-424, DOI: 10.1016/j.ijcard.2014.12.070
173	Dong, D.; Tan-Koi, W. C.; Teng, G. G.; Finkelstein, E.; Sung, C., Cost-effectiveness analysis of genotyping for HLA-B∗5801 and an enhanced safety program in gout patients starting allopurinol in Singapore, Pharmacogenomics, 2015, 16, 16, 1781-1793, DOI: 10.2217/pgs.15.125
174	Gallego, C. J.; Shirts, B. H.; Bennette, C. S.; Guzauskas, G.; Amendola, L. M.; Horike-Pyne, M.; Hisama, F. M.; Pritchard, C. C.; Grady, W. M.; Burke, W.; Jarvik, G. P.; Veenstra, D. L., Next-generation sequencing panels for the diagnosis of colorectal cancer and polyposis syndromes: A cost-effectiveness analysis, Journal of Clinical Oncology, 2015, 33, 18, 2084-2091, DOI: 10.1200/JCO.2014.59.3665
175	Hornberger, J.; Li, Q. Y.; Quinn, B., Cost-Effectiveness of Combinatorial Pharmacogenomic Testing for Treatment-Resistant Major Depressive Disorder Patients, American Journal of Managed Care, 2015, 21, 6, e357-e365, PMID: 26247576
176	Huh, W. K.; Williams, E.; Huang, J.; Bramley, T.; Poulios, N., Cost Effectiveness of Human Papillomavirus-16/18 Genotyping in Cervical Cancer Screening, Applied Health Economics and Health Policy, 2015, 13, 1, 95-107, DOI: 10.1007/s40258-014-0135-4
177	Huxley, N.; Jones-Hughes, T.; Coelho, H.; Snowsill, T.; Cooper, C.; Meng, Y.; Hyde, C.; Mujica-Mota, R., A systematic review and economic evaluation of intraoperative tests [RD-100i one-step nucleic acid amplification (OSNA) system and Metasin test] for detecting sentinel lymph node metastases in breast cancer, Health Technology Assessment, 2015, 19, 2, DOI: 10.3310/hta19020
178	Jahn, B.; Rochau, U.; Kurzthaler, C.; Hubalek, M.; Miksad, R.; Sroczynski, G.; Paulden, M.; Kluibenschadl, M.; Krahn, M.; Siebert, U., Cost effectiveness of personalized treatment in women with early breast cancer: the application of OncotypeDX and Adjuvant! Online to guide adjuvant chemotherapy in Austria, Springerplus, 2015, 4, 752, DOI: 10.1186/s40064-015-1440-6
179	Jiang, M. H.; You, J. H. S., CYP2C19 genotype plus platelet reactivity-guided antiplatelet therapy in acute coronary syndrome patients: a decision analysis, Pharmacogenetics and Genomics, 2015, 25, 12, 609-617, DOI: 10.1097/FPC.0000000000000177

180	Kapoor, R.; Martinez-Vega, R.; Dong, D.; Tan, S. Y.; Leo, Y. S.; Lee, C. C.; Sung, C.; Ng, O. T.; Archuleta, S.; Teo, Y. Y., Reducing hypersensitivity reactions with HLA-B*5701 genotyping before abacavir prescription: clinically useful but is it cost-effective in Singapore?, Pharmacogenetics and Genomics, 2015, 25, 2, 60-72, DOI: 10.1097/FPC.0000000000000107
181	Katz, G.; Romano, O.; Foa, C.; Vataire, A. L.; Chantelard, J. V.; Herve, R.; Barletta, H.; Durieux, A.; Martin, J. P.; Salmon, R., Economic Impact of Gene Expression Profiling in Patients with Early-Stage Breast Cancer in France, Plos One, 2015, 10, 6, e0128880, DOI: 10.1371/journal.pone.0128880
182	Li, Y. H.; Bare, L.; Bender, R.; Sninsky, J.; Wilson, L.; Devlin, J.; Waldman, F., Cost Effectiveness of Sequencing 34 Cancer-Associated Genes as an Aid for Treatment Selection in Patients with Metastatic Melanoma, Molecular Diagnosis & Therapy, 2015, 19, 3, 169-177, DOI: 10.1007/s40291-015-0140-9
183	Luime, J. J.; Buisman, L. R.; Oppe, M.; Hazes, J. M. W.; Rutten-van Molken, Mpmh, Cost-Effectiveness Model for Evaluating New Diagnostic Tests in the Evaluation of Patients With Inflammatory Arthritis at Risk of Having Rheumatoid Arthritis, Arthritis Care & Research, 2015, 68, 7, 927-935, DOI: 10.1002/acr.22776
184	Miquel-Cases, A.; Steuten, L. M. G.; Retel, V. P.; van Harten, W. H., Early stage cost-effectiveness analysis of a BRCA1-like test to detect triple negative breast cancers responsive to high dose alkylating chemotherapy, Breast, 2015, 24, 4, 397-405, DOI: 10.1016/j.breast.2015.03.002
185	Narita, Y.; Matsushima, Y.; Shiroiwa, T.; Chiba, K.; Nakanishi, Y.; Kurokawa, T.; Urushihara, H., Cost-effectiveness analysis of EGFR mutation testing and gefitinib as first-line therapy for non-small cell lung cancer, Lung Cancer, 2015, 90, 1, 71-77, DOI: 10.1016/j.lungcan.2015.07.006
186	Nicholson, A.; Mahon, J.; Boland, A.; Beale, S.; Dwan, K.; Fleeman, N.; Hockenhull, J.; Dundar, Y., The clinical effectiveness and cost-effectiveness of the PROGENSA (R) prostate cancer antigen 3 assay and the Prostate Health Index in the diagnosis of prostate cancer: a systematic review and economic evaluation, Health Technology Assessment, 2015, 19, 87, DOI: 10.3310/hta19870
187	Plumpton, C. O.; Yip, V. L. M.; Alfirevic, A.; Marson, A. G.; Pirmohamed, M.; Hughes, D. A., Cost-effectiveness of screening for HLA-A*31:01 prior to initiation of carbamazepine in epilepsy, Epilepsia, 2015, 56, 4, 556-563, DOI: 10.1111/epi.12937
188	Romanus, D.; Cardarella, S.; Cutler, D.; Landrum, M. B.; Lindeman, N. I.; Gazelle, G. S., Cost-Effectiveness of Multiplexed Predictive Biomarker Screening in Non-Small-Cell Lung Cancer, Journal of Thoracic Oncology, 2015, 10, 4, 586-594, DOI: 10.1097/JTO.0000000000000474

189	Roth, J. A.; Ramsey, S. D.; Carlson, J. J., Cost-Effectiveness of a Biopsy-Based 8-Protein Prostate Cancer Prognostic Assay to Optimize Treatment Decision Making in Gleason 3+3 and 3+4 Early Stage Prostate Cancer, Oncologist, 2015, 20, 12, 1355-1364, DOI: 10.1634/theoncologist.2015-0214
190	Rubio-Terres, C.; Soria, J. M.; Morange, P. E.; Souto, J. C.; Suchon, P.; Mateo, J.; Saut, N.; Rubio-Rodriguez, D.; Sala, J.; Gracia, A.; Pich, S.; Salas, E., Economic Analysis of Thrombo inCode, a Clinical-Genetic Function for Assessing the Risk of Venous Thromboembolism, Applied Health Economics and Health Policy, 2015, 13, 2, 233-242, DOI: 10.1007/s40258-015-0153-x
191	Schackman, B. R.; Haas, D. W.; Park, S. S.; Li, X. C.; Freedberg, K. A., Cost–effectiveness of CYP2B6 genotyping to optimize efavirenz dosing in HIV clinical practice, Pharmacogenomics, 2015, 16, 18, 2007-2018, DOI: 10.2217/pgs.15.142
192	Schremser, K.; Rogowski, W. H.; Adler-Reichel, S.; Tufman, A. L. H.; Huber, R. M.; Stollenwerk, B., Cost-Effectiveness of an Individualized First-Line Treatment Strategy Offering Erlotinib Based on EGFR Mutation Testing in Advanced Lung Adenocarcinoma Patients in Germany, Pharmacoeconomics, 2015, 33, 11, 1215-1228, DOI: 10.1007/s40273-015-0305-8
193	Severin, F.; Stollenwerk, B.; Holinski-Feder, E.; Meyer, E.; Heinemann, V.; Giessen-Jung, C.; Rogowski, W., Economic evaluation of genetic screening for Lynch syndrome in Germany, Genetics in Medicine, 2015, 17, 10, 765-773, DOI: 10.1038/gim.2014.190
194	Shiffman, D.; Perez, M. V.; Bare, L. A.; Louie, J. Z.; Arellano, A. R.; Devlin, J. J., Genetic risk for atrial fibrillation could motivate patient adherence to warfarin therapy: a cost effectiveness analysis, Bmc Cardiovascular Disorders, 2015, 15, 104, DOI: 10.1186/s12872-015-0100-7
195	Snowsill, T.; Huxley, N.; Hoyle, M.; Jones-Hughes, T.; Coelho, H.; Cooper, C.; Frayling, I.; Hyde, C., A model-based assessment of the cost-utility of strategies to identify Lynch syndrome in early-onset colorectal cancer patients, Bmc Cancer, 2015, 15, 313, DOI: 10.1186/s12885-015-1254-5
196	You, J. H. S., Universal versus genotype-guided use of direct oral anticoagulants in atrial fibrillation patients: a decision analysis, Pharmacogenomics, 2015, 16, 10, 1089-1100, DOI: 10.2217/PGS.15.64
197	Ademi, Z.; Watts, G. F.; Pang, J.; Sijbrands, E. J. G.; van Bockxmeer, F. M.; O'Leary, P.; Geelhoed, E.; Liew, D., Cascade screening based on genetic testing is cost-effective: Evidence for the implementation of models of care for familial hypercholesterolemia, Journal of Clinical Lipidology, 2014, 8, 4, 390-400, DOI: 10.1016/j.jacl.2014.05.008

198	Bonastre, J.; Marguet, S.; Lueza, B.; Michiels, S.; Delaloge, S.; Saghatchian, M.; Lux, L. J.; Posey, R. E.; Daniels, L. S.; Henke, D. C.; Durham, C.; Jonas, D. E.; Lohr, K. N., Cost Effectiveness of Molecular Profiling for Adjuvant Decision Making in Patients With Node-Negative Breast Cancer, Journal of Clinical Oncology, 2014, 32, 31, 3513-3519, DOI: 10.1200/JCO.2013.54.9931
199	Chong, H. Y.; Saokaew, S.; Dumrongprat, K.; Permsuwan, U.; Wu, D. B. C.; Sritara, P.; Chaiyakunapruk, N., Cost-effectiveness analysis of pharmacogenetic-guided warfarin dosing in Thailand, Thrombosis Research, 2014, 134, 6, 1278-1284, DOI: 10.1016/j.thromres.2014.10.006
200	Curl, P.; Vujic, I.; van 't Veer, L. J.; Ortiz-Urda, S.; Kahn, J. G., Cost-Effectiveness of Treatment Strategies for BRAFMutated Metastatic Melanoma, Plos One, 2014, 9, 9, e107255, DOI: 10.1371/journal.pone.0107255
201	Dan, Y. Y.; Ferrante, S. A.; Elbasha, E. H.; Hsu, T. Y., Cost-effectiveness of boceprevir co-administration versus pegylated interferon-α2b and ribavirin only for patients with hepatitis C genotype 1 in Singapore, Antiviral Therapy, 2014, 20, 2, 209-216, DOI: 10.3851/IMP2825
202	Djalalov, S.; Beca, J.; Hoch, J. S.; Krahn, M.; Tsao, M. S.; Cutz, J. C.; Leighl, N. B., Cost Effectiveness of EML4-ALK Fusion Testing and First-Line Crizotinib Treatment for Patients With Advanced ALK-Positive Non-Small-Cell Lung Cancer, Journal of Clinical Oncology, 2014, 32, 10, 1012-1019, DOI: 10.1200/JCO.2013.53.1186
203	Green, L. E.; Dinh, T. A.; Hinds, D. A.; Walser, B. L.; Allman, R., Economic evaluation of using a genetic test to direct breast cancer chemoprevention in white women with a previous breast biopsy, Applied Health Economics and Health Policy, 2014, 12, 2, 203-217, DOI: 10.1007/s40258-014-0089-6
204	Holko, P.; Kawalec, P., Economic evaluation of sipuleucel-T immunotherapy in castration-resistant prostate cancer, Expert Review of Anticancer Therapy, 2014, 14, 1, 63-73, DOI: 10.1586/14737140.2014.856270
205	Kazi, D. S.; Garber, A. M.; Shah, R. U.; Dudley, R. A.; Mell, M. W.; Rhee, C.; Moshkevich, S.; Boothroyd, D. B.; Owens, D. K.; Hlatky, M. A., Cost-Effectiveness of Genotype-Guided and Dual Antiplatelet Therapies in Acute Coronary Syndrome, Annals of Internal Medicine, 2014, 160, 4, 221-232, DOI: 10.7326/M13-1999
206	Ladabaum, U.; Alvarez-Osorio, L.; Rösch, T.; Brueggenjuergen, B., Cost-effectiveness of colorectal cancer screening in Germany: Current endoscopic and fecal testing strategies versus plasma methylated Septin 9 DNA 1, Endoscopy International Open, 2014, 2, 2, E96-E104, DOI: 10.1055/s-0034-1377182

207	Lee, L.; How, J.; Tabah, R. J.; Mitmaker, E. J., Cost-Effectiveness of Molecular Testing for Thyroid Nodules With Atypia of Undetermined Significance Cytology, Journal of Clinical Endocrinology & Metabolism, 2014, 99, 8, 2674-2682, DOI: 10.1210/jc.2014-1219
208	Mitropoulou, C.; Fragoulakis, V.; Bozina, N.; Vozikis, A.; Supe, S.; Bozina, T.; Poljakovic, Z.; van Schaik, R. H.; Patrinos, G. P., Economic evaluation of pharmacogenomic-guided warfarin treatment for elderly Croatian atrial fibrillation patients with ischemic stroke, Pharmacogenomics, 2014, 16, 2, 137-148, DOI: 10.2217/pgs.14.167
209	Naylor, R. N.; John, P. M.; Winn, A. N.; Carmody, D.; Greeley, S. A. W.; Philipson, L. H.; Bell, G. I.; Huang, E. S., Cost-effectiveness of MODY genetic testing: translating genomic advances into practical health applications, Diabetes Care, 2014, 37, 1, 202-209, DOI: 10.2337/dc13-0410
210	Patel, V.; Lin, F. J.; Ojo, O.; Rao, S.; Yu, S.; Zhan, L.; Touchette, D. R., Cost-utility analysis of genotype-guided antiplatelet therapy in patients with moderate-to- high risk acute coronary syndrome and planned percutaneous coronary intervention, Pharmacy Practice, 2014, 12, 3, 438, DOI: 10.4321/S1886-36552014000300007
211	Permsuwan, U.; Niamhun, N.; Tanatip, N.; Thongprasert, S., Epidermal growth factor receptor mutation testing in Thailand: A cost-utility analysis, Value in Health Regional Issues, 2014, 3, 1, 39-43, DOI: 10.1016/j.vhri.2013.12.001
212	Phelps, C. E.; O'Sullivan, A. K.; Ladapo, J. A.; Weinstein, M. C.; Leahy, K.; Douglas, P. S., Cost effectiveness of a gene expression score and myocardial perfusion imaging for diagnosis of coronary artery disease, American Heart Journal, 2014, 167, 5, 697-706.e2, DOI: 10.1016/j.ahj.2014.02.005
213	Pink, J.; Pirmohamed, M.; Lane, S.; Hughes, D. A., Cost-Effectiveness of Pharmacogenetics-Guided Warfarin Therapy vs. Alternative Anticoagulation in Atrial Fibrillation, Clinical Pharmacology and Therapeutics, 2014, 95, 2, 199-207, DOI: 10.1038/clpt.2013.190
214	Rejon-Parrilla, J. C.; Nuijten, M.; Redekop, W. K.; Gaultney, J. G., Economic evaluation of the use of a pharmacogenetic diagnostic test in schizophrenia, Health Policy and Technology, 2014, 3, 4, 314-324, DOI: 10.1016/j.hlpt.2014.08.004
215	Roth, J. A.; Billings, P.; Ramsey, S. D.; Dumanois, R.; Carlson, J. J., Cost-Effectiveness of a 14-Gene Risk Score Assay to Target Adjuvant Chemotherapy in Early Stage Non-Squamous Non-Small Cell Lung Cancer, Oncologist, 2014, 19, 5, 466-476, DOI: 10.1634/theoncologist.2013-0357
216	Saokaew, S.; Tassaneeyakul, W.; Maenthaisong, R.; Chaiyakunapruk, N., Cost-Effectiveness Analysis of HLA-B*5801 Testing in Preventing Allopurinol-Induced SJS/TEN in Thai Population, Plos One, 2014, 9, 4, e94294, DOI: 10.1371/journal.pone.0094294

217	Sax, P. E.; Sypek, A.; Berkowitz, B. K.; Morris, B. L.; Losina, E.; Paltiel, A. D.; Kelly, K. A.; Seage, G. R.; Walensky, R. P.; Weinstein, M. C.; Eron, J.; Freedberg, K. A., HIV cure strategies: How good must they be to improve on current antiretroviral therapy?, Plos One, 2014, 9, 11, e113031, DOI: 10.1371/journal.pone.0113031
218	Segui, M. A.; Crespo, C.; Cortes, J.; Lluch, A.; Brosa, M.; Becerra, V.; Chiavenna, S. M.; Gracia, A., Genomic profile of breast cancer: cost–effectiveness analysis from the Spanish National Healthcare System perspective, Expert Review of Pharmacoeconomics & Outcomes Research, 2014, 14, 6, 889-899, DOI: 10.1586/14737167.2014.957185
219	Verhoef, T. I.; Redekop, W. K.; de Boer, A.; Maitland-van der Zee, A. H., Economic evaluation of a pharmacogenetic dosing algorithm for coumarin anticoagulants in The Netherlands, Pharmacogenomics, 2014, 16, 2, 101-114, DOI: 10.2217/pgs.14.149
220	Vriens, D.; Adang, E. M. M.; Netea-Maier, R. T.; Smit, J. W. A.; de Wilt, J. H. W.; Oyen, W. J. G.; de Geus-Oei, L. F., Cost-Effectiveness of FDG-PET/CT for Cytologically Indeterminate Thyroid Nodules: A Decision Analytic Approach, Journal of Clinical Endocrinology & Metabolism, 2014, 99, 9, 3263-3274, DOI: 10.1210/jc.2013-3483
221	Yamauchi, H.; Nakagawa, C.; Yamashige, S.; Takei, H.; Yagata, H.; Yoshida, A.; Hayashi, N.; Hornberger, J.; Yu, T.; Chao, C.; Yoshizawa, C.; Nakamura, S., Societal cost-effectiveness analysis of the 21-gene assay in estrogen-receptor-positive, lymph-node-negative early-stage breast cancer in Japan, Bmc Health Services Research, 2014, 14, 372, DOI: 10.1186/1472-6963-14-372
222	Compagni, A.; Melegaro, A.; Tarricone, R., Genetic Screening for the Predisposition to Venous Thromboembolism: A Cost-Utility Analysis of Clinical Practice in the Italian Health Care System, Value in Health, 2013, 16, 6, 909-921, DOI: 10.1016/j.jval.2013.05.003
223	Garrison Jr, L. P.; Lalla, D.; Brammer, M.; Babigumira, J. B.; Wang, B.; Perez, E. A., Assessing the potential cost-effectiveness of retesting IHC0, IHC1+, or FISH-negative early stage breast cancer patients for HER2 status, Cancer, 2013, 119, 17, 3113-3122, DOI: 10.1002/cncr.28196
224	Ghatnekar, O.; Andersson, R.; Svensson, M.; Persson, U.; Ringdahl, U.; Zeilon, P.; Borrebaeck, C. A. K., Modelling the benefits of early diagnosis of pancreatic cancer using a biomarker signature, International Journal of Cancer, 2013, 133, 10, 2392-2397, DOI: 10.1002/ijc.28256
225	Govers, T. M.; Takes, R. P.; Karakullukcu, M. B.; Hannink, G.; Merkx, M. A. W.; Grutters, J. P. C.; Rovers, M. M., Management of the N0 neck in early stage oral squamous cell cancer: A modeling study of the cost-effectiveness, Oral Oncology, 2013, 49, 8, 771-777, DOI: 10.1016/j.oraloncology.2013.05.001

226	Holt, S.; Bertelli, G.; Humphreys, I.; Valentine, W.; Durrani, S.; Pudney, D.; Rolles, M.; Moe, M.; Khawaja, S.; Sharaiha, Y.; Brinkworth, E.; Whelan, S.; Jones, S.; Bennett, H.; Phillips, C. J., A decision impact, decision conflict and economic assessment of routine Oncotype DX testing of 146 women with node-negative or pNImi, ER-positive breast cancer in the UK, British Journal of Cancer, 2013, 108, 11, 2250-2258, DOI: 10.1038/bjc.2013.207
227	Hornberger, J.; Degtiar, I.; Gutierrez, H.; Shewade, A.; Henner, W. D.; Becker, S.; Varadachary, G.; Raab, S., Cost-Effectiveness of Gene-Expression Profiling for Tumor-Site Origin, Value in Health, 2013, 16, 1, 46-56, DOI: 10.1016/j.jval.2012.09.005
228	Jacobs, V. R.; Kates, R. E.; Kantelhardt, E.; Vetter, M.; Wuerstlein, R.; Fischer, T.; Schmitt, M.; Jaenicke, F.; Untch, M.; Thomssen, C.; Harbeck, N., Health economic impact of risk group selection according to ASCO-recommended biomarkers uPA/PAI-1 in node-negative primary breast cancer, Breast Cancer Research and Treatment, 2013, 138, 3, 839-850, DOI: 10.1007/s10549-013-2496-z
229	Kansal, A. R.; Shaul, A. J.; Stern, S.; Busam, K.; Doucet, C. A.; Chalfin, D. B., Cost–effectiveness of a FISH assay for the diagnosis of melanoma in the USA, Expert Review of Pharmacoeconomics and Outcomes Research, 2013, 13, 3, 371-380, DOI: 10.1586/erp.13.22
230	Ladabaum, U.; Allen, J.; Wandell, M.; Ramsey, S., Colorectal Cancer Screening with Blood-Based Biomarkers: Cost-Effectiveness of Methylated Septin 9 DNA versus Current Strategies, Cancer Epidemiology Biomarkers & Prevention, 2013, 22, 9, 1567-1576, DOI: 10.1158/1055-9965.EPI-13-0204
231	Lieberthal, R. D.; Dudash, K.; Axelrod, R.; Goldfarb, N. I., An economic model to value companion diagnostics in non-small-cell lung cancer, Personalized Medicine, 2013, 10, 2, 139-147, DOI: 10.2217/PME.13.7
232	Nelson, R. E.; Stenehjem, D.; Akerley, W., A comparison of individualized treatment guided by VeriStrat with standard of care treatment strategies in patients receiving second-line treatment for advanced non-small cell lung cancer: A cost-utility analysis, Lung Cancer, 2013, 82, 3, 461-468, DOI: 10.1016/j.lungcan.2013.08.021
233	Nshimyumukiza, L.; Duplantie, J.; Gagnon, M.; Douville, X.; Fournier, D.; Lindsay, C.; Parent, M.; Milot, A.; Giguere, Y.; Gagne, C.; Rousseau, F.; Reinharz, D., Dabigatran versus warfarin under standard or pharmacogenetic-guided management for the prevention of stroke and systemic thromboembolism in patients with atrial fibrillation: a cost/utility analysis using an analytic decision model, Thrombosis Journal, 2013, 11, 1, 14, DOI: 10.1186/1477-9560-11-14

234	Parthan, A.; Leahy, K. J.; O'Sullivan, A. K.; Iakoubova, O. A.; Bare, L. A.; Devlin, J. J.; Weinstein, M. C., Cost Effectiveness of Targeted High-dose Atorvastatin Therapy Following Genotype Testing in Patients with Acute Coronary Syndrome, Pharmacoeconomics, 2013, 31, 6, 519-531, DOI: 10.1007/s40273-013-0054-5
235	Paulden, M.; Franek, J.; Pham, B.; Bedard, P. L.; Trudeau, M.; Krahn, M., Cost-effectiveness of the 21-gene assay for guiding adjuvant chemotherapy decisions in early breast cancer., Value in Health, 2013, 16, 5, 729-739, DOI: 10.1016/j.jval.2013.03.1625
236	Rattanavipapong, W.; Koopitakkajorn, T.; Praditsitthikorn, N.; Mahasirimongkol, S.; Teerawattananon, Y., Economic evaluation of HLA-B*15:02 screening for carbamazepine-induced severe adverse drug reactions in Thailand, Epilepsia, 2013, 54, 9, 1628-1638, DOI: 10.1111/epi.12325
237	Reed, S. D.; Dinan, M. A.; Schulman, K. A.; Lyman, G. H., Cost-effectiveness of the 21-gene recurrence score assay in the context of multifactorial decision making to guide chemotherapy for early-stage breast cancer, Genetics in Medicine, 2013, 15, 3, 203-211, DOI: 10.1038/gim.2012.119
238	Retel, V. P.; Joore, M. A.; Drukker, C. A.; Bueno-de-Mesquita, J. M.; Knauer, M.; van Tinteren, H.; Linn, S. C.; van Harten, W. H., Prospective cost-effectiveness analysis of genomic profiling in breast cancer, European Journal of Cancer, 2013, 49, 18, 3773-3779, DOI: 10.1016/j.ejca.2013.08.001
239	Schackman, B. R.; Haas, D. W.; Becker, J. E.; Berkowitz, B. K.; Sax, P. E.; Daar, E. S.; Ribaudo, H. J.; Freedberg, K. A., Cost-effectiveness analysis of UGT1A1 genetic testing to inform antiretroviral prescribing in HIV disease, Antiviral Therapy, 2013, 18, 3, 399-408, DOI: 10.3851/IMP2500
240	Sorich, M. J.; Horowitz, J. D.; Sorich, W.; Wiese, M. D.; Pekarsky, B.; Karnon, J. D., Cost-effectiveness of using CYP2C19 genotype to guide selection of clopidogrel or ticagrelor in Australia, Pharmacogenomics, 2013, 14, 16, 2013-2021, DOI: 10.2217/PGS.13.164
241	Thompson, A. J.; Newman, W. G.; Elliott, R. A.; Roberts, S. A.; Tricker, K.; Payne, K., The Cost-Effectiveness of a Pharmacogenetic Test: A Trial-Based Evaluation of TPMT Genotyping for Azathioprine, Value in Health, 2013, 17, 1, 22-33, DOI: 10.1016/j.jval.2013.10.007
242	Verhoef, T. I.; Redekop, W. K.; Veenstra, D. L.; Thariani, R.; Beltman, P. A.; van Schie, R. M. F.; de Boer, A.; Maitland-van der Zee, A. H., Cost-effectiveness of pharmacogenetic-guided dosing of phenprocoumon in atrial fibrillation, Pharmacogenomics, 2013, 14, 8, 869-883, DOI: 10.2217/PGS.13.74

243	Ward, S.; Scope, A.; Rafia, R.; Pandor, A.; Harnan, S.; Evans, P.; Wyld, L., Gene expression profiling and expanded immunohistochemistry tests to guide the use of adjuvant chemotherapy in breast cancer management: a systematic review and cost-effectiveness analysis, Health Technology Assessment, 2013, 17, 44, DOI: 10.3310/hta17440
244	You, J. H., Pharmacogenetic-guided selection of warfarin versus novel oral anticoagulants for stroke prevention in patients with atrial fibrillation: a cost-effectiveness analysis, Pharmacogenetics and Genomics, 2013, 24, 1, 6-14, DOI: 10.1097/FPC.0000000000000014
245	Zhu, J.; Li, T.; Wang, X. H.; Ye, M.; Cai, J.; Xu, Y. J.; Wu, B., Gene-guided Gefitinib switch maintenance therapy for patients with advanced EGFR mutation-positive Non-small cell lung cancer: an economic analysis, Bmc Cancer, 2013, 13, 39, DOI: 10.1186/1471-2407-13-39
246	Behl, A. S.; Goddard, K. A. B.; Flottemesch, T. J.; Veenstra, D.; Meenan, R. T.; Lin, J. S.; Maciosek, M. V., Cost-effectiveness analysis of screening for KRAS and BRAF mutations in metastatic colorectal cancer, Journal of the National Cancer Institute, 2012, 104, 23, 1785-1795, DOI: 10.1093/jnci/djs433
247	Blohmer, J. U.; Rezai, M.; Kümmel, S.; Kühn, T.; Warm, M.; Friedrichs, K.; Benkow, A.; Valentine, W. J.; Eiermann, W., Using the 21-gene assay to guide adjuvant chemotherapy decision-making in early-stage breast cancer: A cost-effectiveness evaluation in the German setting, Journal of Medical Economics, 2012, 16, 1, 30-40, DOI: 10.3111/13696998.2012.722572
248	De Lima Lopes, G.; Segel, J. E.; Tan, D. S. W.; Do, Y. K.; Mok, T.; Finkelstein, E. A., Cost-Effectiveness of Epidermal Growth Factor Receptor Mutation Testing and First-Line Treatment With Gefitinib for Patients With Advanced Adenocarcinoma of the Lung, Cancer, 2012, 118, 4, 1032-1039, DOI: 10.1002/cncr.26372
249	Djalalov, S.; Yong, J.; Beca, J.; Black, S.; Saposnik, G.; Musa, Z.; Siminovitch, K.; Moretti, M.; Hoch, J. S., Genetic Testing in Combination with Preventive Donepezil Treatment for Patients with Amnestic Mild Cognitive Impairment, Molecular Diagnosis & Therapy, 2012, 16, 6, 389-399, DOI: 10.1007/s40291-012-0010-7
250	Dong, D.; Sung, C.; Finkelstein, E. A., Cost-effectiveness of HLA-B*1502 genotyping in adult patients with newly diagnosed epilepsy in Singapore, Neurology, 2012, 79, 12, 1259-1267, DOI: 10.1212/WNL.0b013e31826aac73
251	Hall, P. S.; McCabe, C.; Stein, R. C.; Cameron, D.,<br>Economic Evaluation of Genomic Test-Directed Chemotherapy for Early-Stage Lymph Node-Positive Breast Cancer, Jnci-Journal of the National Cancer Institute, 2012, 104, 1, 56-66, DOI: 10.1093/jnci/djr484

252	Handorf, E. A.; McElligott, S.; Vachani, A.; Langer, C. J.; Demeter, M. B.; Armstrong, K.; Asch, D. A., Cost Effectiveness of Personalized Therapy for First-Line Treatment of Stage IV and Recurrent Incurable Adenocarcinoma of the Lung, Journal of Oncology Practice, 2012, 8, 5, 267-274, DOI: 10.1200/JOP.2011.000502
253	Ingles, J.; McGaughran, J.; Scuffham, P. A.; Atherton, J.; Semsarian, C., A cost-effectiveness model of genetic testing for the evaluation of families with hypertrophic cardiomyopathy, Heart, 2012, 98, 8, 625-630, DOI: 10.1136/heartjnl-2011-300368
254	Lala, A.; Berger, J. S.; Sharma, G.; Hochman, J. S.; Braithwaite, R. S.; Ladapo, J. A., Genetic testing in patients with acute coronary syndrome undergoing percutaneous coronary intervention: a cost-effectiveness analysis, Journal of Thrombosis and Haemostasis, 2012, 11, 1, 81-91, DOI: 10.1111/jth.12059
255	Liu, S.; Cipriano, L. E.; Holodniy, M.; Owens, D. K.; Goldhaber-Fiebert, J. D., New Protease Inhibitors for the Treatment of Chronic Hepatitis C A Cost-Effectiveness Analysis, Annals of Internal Medicine, 2012, 156, 4, 279-U68, DOI: 10.7326/0003-4819-156-4-201202210-00005
256	Najafzadeh, M.; Marra, C. A.; Lynd, L. D.; Wiseman, S. M., Cost-Effectiveness of Using a Molecular Diagnostic Test to Improve Preoperative Diagnosis of Thyroid Cancer, Value in Health, 2012, 15, 8, 1005-1013, DOI: 10.1016/j.jval.2012.06.017
257	Panattoni, L.; Brown, P. M.; Te Ao, B.; Webster, M.; Gladding, P., The Cost Effectiveness of Genetic Testing for CYP2C19 Variants to Guide Thienopyridine Treatment in Patients with Acute Coronary Syndromes A New Zealand Evaluation, Pharmacoeconomics, 2012, 30, 11, 1067-1084, DOI: 10.2165/11595080-000000000-00000
258	Perlis, R. H.; Patrick, A.; Smoller, J. W.; Wang, P. S., When is pharmacogenetic testing for antidepressant response ready for the clinic? A cost-effectiveness analysis based on data from the STAR*D study , Neuropsychopharmacology, 2012, 34, 10, 2227-2236, DOI: 10.1038/npp.2009.50
259	Retèl, V. P.; Joore, M. A.; Van Harten, W. H., Head-to-head comparison of the 70-gene signature versus the 21-gene assay: Cost-effectiveness and the effect of compliance, Breast Cancer Research and Treatment, 2012, 131, 2, 627-636, DOI: 10.1007/s10549-011-1769-7
260	Shiffman, D.; Slawsky, K.; Fusfeld, L.; Devlin, J. J.; Goss, T. F., Cost-Effectiveness Model of Use of Genetic Testing as an Aid in Assessing the Likely Benefit of Aspirin Therapy for Primary Prevention of Cardiovascular Disease, Clinical Therapeutics, 2012, 34, 6, 1387-1394, DOI: 10.1016/j.clinthera.2012.04.004

261	Wang, G.; Kuppermann, M.; Kim, B.; Phillips, K. A.; Ladabaum, U., Influence of patient preferences on the cost-effectiveness of screening for lynch syndrome, Journal of Oncology Practice, 2012, 8, 3S, e24s-e30s, DOI: 10.1200/JOP.2011.000535
262	Wang, V. W.; Koh, P. K.; Chow, W. L.; Lim, J. F. Y., Predictive genetic testing of first degree relatives of mutation carriers is a cost-effective strategy in preventing hereditary non-polyposis colorectal cancer in Singapore, Familial Cancer, 2012, 11, 2, 279-289, DOI: 10.1007/s10689-012-9513-y
263	Wong, I. O. L.; Tsang, J. W. H.; Cowling, B. J.; Leung, G. M., Optimizing resource allocation for breast cancer prevention and care among Hong Kong Chinese women, Cancer, 2012, 118, 18, 4394-4403, DOI: 10.1002/cncr.27448
264	Yang, M.; Rajan, S.; Issa, A. M., Cost effectiveness of gene expression profiling for early stage breast cancer, Cancer, 2012, 118, 20, 5163-5170, DOI: 10.1002/cncr.27443
265	You, J. H. S.; Tsui, K. K. N.; Wong, R. S. M.; Cheng, G., Cost-Effectiveness of Dabigatran versus Genotype-Guided Management of Warfarin Therapy for Stroke Prevention in Patients with Atrial Fibrillation, Plos One, 2012, 7, 6, e39640, DOI: 10.1371/journal.pone.0039640
266	Blank, P. R.; Moch, H.; Szucs, T. D.; Schwenkglenks, M., KRAS and BRAF mutation analysis in metastatic colorectal cancer: A cost-effectiveness analysis from a Swiss perspective, Clinical Cancer Research, 2011, 17, 19, 6338-6346, DOI: 10.1158/1078-0432.CCR-10-2267
267	Chen, M. K.; Hung, H. F.; Duffy, S.; Yen, A. M. F.; Chen, H. H., Cost-effectiveness analysis for Pap smear screening and human papillomavirus DNA testing and vaccination, Journal of Evaluation in Clinical Practice, 2011, 17, 6, 1050-1058, DOI: 10.1111/j.1365-2753.2010.01453.x
268	Crespin, D. J.; Federspiel, J. J.; Biddle, A. K.; Jonas, D. E.; Rossi, J. S., Ticagrelor versus Genotype-Driven Antiplatelet Therapy for Secondary Prevention after Acute Coronary Syndrome A Cost-Effectiveness Analysis, Value in Health, 2011, 14, 4, 483-491, DOI: 10.1016/j.jval.2010.11.012
269	Dinh, T. A.; Rosner, B. I.; Atwood, J. C.; Boland, C. R.; Syngal, S.; Vasen, H. F. A.; Gruber, S. B.; Burt, R. W., Health Benefits and Cost-Effectiveness of Primary Genetic Screening for Lynch Syndrome in the General Population, Cancer Prevention Research, 2011, 4, 1, 9-22, DOI: 10.1158/1940-6207.CAPR-10-0262
270	Greeley, S. A.; John, P. M.; Winn, A. N.; Ornelas, J.; Lipton, R. B.; Philipson, L. H.; Bell, G. I.; Huang, E. S., The cost-effectiveness of personalized genetic medicine: the case of genetic testing in neonatal diabetes, Diabetes Care, 2011, 34, 3, 622-627, DOI: 10.2337/dc10-1616

271	Ladabaum, U.; Wang, G.; Terdiman, J.; Blanco, A.; Kuppermann, M.; Boland, C. R.; Ford, J.; Elkin, E.; Phillips, K. A., Strategies to identify the Lynch syndrome among patients with colorectal cancer: a cost-effectiveness analysis, Annals of Internal Medicine, 2011, 155, 2, 69-79, DOI: 10.7326/0003-4819-155-2-201107190-00002
272	Li, H.; Robinson, K. A.; Anton, B.; Saldanha, I. J.; Ladenson, P. W., Cost-Effectiveness of a Novel Molecular Test for Cytologically Indeterminate Thyroid Nodules, Journal of Clinical Endocrinology & Metabolism, 2011, 96, 11, E1719-E1726, DOI: 10.1210/jc.2011-0459
273	Nherera, L.; Marks, D.; Minhas, R.; Thorogood, M.; Humphries, S. E., Probabilistic cost-effectiveness analysis of cascade screening for familial hypercholesterolaemia using alternative diagnostic and identification strategies, Heart, 2011, 97, 14, 1175-1181, DOI: 10.1136/hrt.2010.213975
274	Perez, M. V.; Kumarasamy, N. A.; Owens, D. K.; Wang, P. J.; Hlatky, M. A., Cost-Effectiveness of Genetic Testing in Family Members of Patients With Long-QT Syndrome, Circulation-Cardiovascular Quality and Outcomes, 2011, 4, 1, 76-84, DOI: 10.1161/CIRCOUTCOMES.110.957365
275	Woods, B.; Veenstra, D.; Hawkins, N., Prioritizing Pharmacogenetic Research: A Value of Information Analysis of CYP2D6 Testing to Guide Breast Cancer Treatment, Value in Health, 2011, 14, 8, 989-1001, DOI: 10.1016/j.jval.2011.05.048
