# Supplementary material for: Mapping the value for money of precision medicine: a systematic literature review and meta-analysis
Source: Front Public Health. 2023 Nov 24;11:1151504. doi: 10.3389/fpubh.2023.1151504 (PMC10704154; doi:10.3389/fpubh.2023.1151504)
Supplement: Supplementary file 9 [file Image_1.PDF]

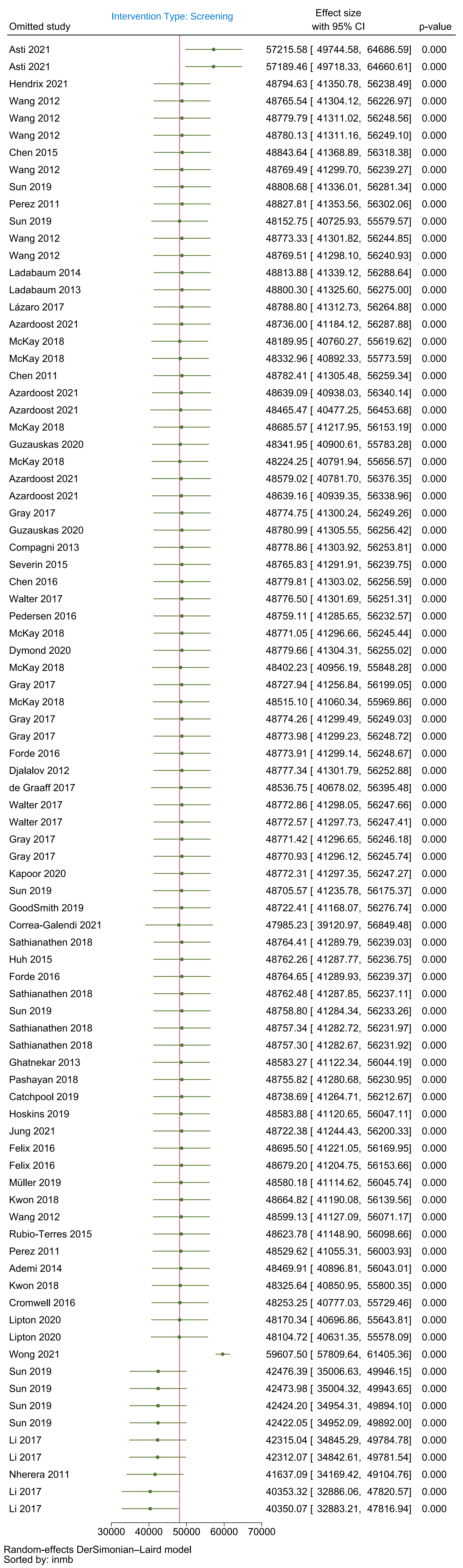
Omitted study
Intervention Type: Screening
Effect size with 95% CI
p-value
Asti 2021 57215.58 [49744.58, 64686.59] 0.000
Asti 2021 57189.46 [49718.33, 64660.61] 0.000
Hendrix 2021 48794.63 [41350.78, 56238.49] 0.000
Wang 2012 48765.54 [41304.12, 56226.97] 0.000
Wang 2012 48779.79 [41311.02, 56248.56] 0.000
Wang 2012 48780.13 [41311.16, 56249.10] 0.000
Chen 2015 48843.64 [41368.89, 56318.38] 0.000
Wang 2012 48769.49 [41299.70, 56239.27] 0.000
Sun 2019 48808.68 [41336.01, 56281.34] 0.000
Perez 2011 48827.81 [41353.56, 56302.06] 0.000
Sun 2019 48152.75 [40725.93, 55579.57] 0.000
Wang 2012 48773.33 [41301.82, 56244.85] 0.000
Wang 2012 48769.51 [41298.10, 56240.93] 0.000
Ladabaum 2014 48813.88 [41339.12, 56288.64] 0.000
Ladabaum 2013 48800.30 [41325.60, 56275.00] 0.000
Lázaro 2017 48788.80 [41312.73, 56264.88] 0.000
Azardoost 2021 48736.00 [41184.12, 56287.88] 0.000
McKay 2018 48189.95 [40760.27, 55619.62] 0.000
McKay 2018 48332.96 [40892.33, 55773.59] 0.000
Chen 2011 48782.41 [41305.48, 56259.34] 0.000
Azardoost 2021 48639.09 [40938.03, 56340.14] 0.000
Azardoost 2021 48465.47 [40477.25, 56453.68] 0.000
McKay 2018 48685.57 [41217.95, 56153.19] 0.000
Guzauskas 2020 48341.95 [40900.61, 55783.28] 0.000
McKay 2018 48224.25 [40791.94, 55656.57] 0.000
Azardoost 2021 48579.02 [40781.70, 56376.35] 0.000
Azardoost 2021 48639.16 [40939.35, 56338.96] 0.000
Gray 2017 48774.75 [41300.24, 56249.26] 0.000
Guzauskas 2020 48780.99 [41305.55, 56256.42] 0.000
Compagni 2013 48778.86 [41303.92, 56253.81] 0.000
Severin 2015 48765.83 [41291.91, 56239.75] 0.000
Chen 2016 48779.81 [41303.02, 56256.59] 0.000
Walter 2017 48776.50 [41301.69, 56251.31] 0.000
Pedersen 2016 48759.11 [41285.65, 56232.57] 0.000
McKay 2018 48771.05 [41296.66, 56245.44] 0.000
Dymond 2020 48779.66 [41304.31, 56255.02] 0.000
McKay 2018 48402.23 [40956.19, 55848.28] 0.000
Gray 2017 48727.94 [41256.84, 56199.05] 0.000
McKay 2018 48515.10 [41060.34, 55969.86] 0.000
Gray 2017 48774.26 [41299.49, 56249.03] 0.000
Gray 2017 48773.98 [41299.23, 56248.72] 0.000
Forde 2016 48773.91 [41299.14, 56248.67] 0.000
Djalalov 2012 48777.34 [41301.79, 56252.88] 0.000
de Graaff 2017 48536.75 [40678.02, 56395.48] 0.000
Walter 2017 48772.86 [41298.05, 56247.66] 0.000
Walter 2017 48772.57 [41297.73, 56247.41] 0.000
Gray 2017 48771.42 [41296.65, 56246.18] 0.000
Gray 2017 48770.93 [41296.12, 56245.74] 0.000
Kapoor 2020 48772.31 [41297.35, 56247.27] 0.000
Sun 2019 48705.57 [41235.78, 56175.37] 0.000
GoodSmith 2019 48722.41 [41168.07, 56276.74] 0.000
Correa-Galendi 2021 47985.23 [39120.97, 56849.48] 0.000
Sathianathen 2018 48764.41 [41289.79, 56239.03] 0.000
Huh 2015 48762.26 [41287.77, 56236.75] 0.000
Forde 2016 48764.65 [41289.93, 56239.37] 0.000
Sathianathen 2018 48762.48 [41287.85, 56237.11] 0.000
Sun 2019 48758.80 [41284.34, 56233.26] 0.000
Sathianathen 2018 48757.34 [41282.72, 56231.97] 0.000
Sathianathen 2018 48757.30 [41282.67, 56231.92] 0.000
Ghatnekar 2013 48583.27 [41122.34, 56044.19] 0.000
Pashayan 2018 48755.82 [41280.68, 56230.95] 0.000
Catchpool 2019 48738.69 [41264.71, 56212.67] 0.000
Hoskins 2019 48583.88 [41120.65, 56047.11] 0.000
Jung 2021 48722.38 [41244.43, 56200.33] 0.000
Felix 2016 48695.50 [41221.05, 56169.95] 0.000
Felix 2016 48679.20 [41204.75, 56153.66] 0.000
Müller 2019 48580.18 [41114.62, 56045.74] 0.000
Kwon 2018 48664.82 [41190.08, 56139.56] 0.000
Wang 2012 48599.13 [41127.09, 56071.17] 0.000
Rubio-Terres 2015 48623.78 [41148.90, 56098.66] 0.000
Perez 2011 48529.62 [41055.31, 56003.93] 0.000
Ademi 2014 48469.91 [40896.81, 56043.01] 0.000
Kwon 2018 48325.64 [40850.95, 55800.35] 0.000
Cromwell 2016 48253.25 [40777.03, 55729.46] 0.000
Lipton 2020 48170.34 [40696.86, 55643.81] 0.000
Lipton 2020 48104.72 [40631.35, 55578.09] 0.000
Wong 2021 59607.50 [57809.64, 61405.36] 0.000
Sun 2019 42476.39 [35006.63, 49946.15] 0.000
Sun 2019 42473.98 [35004.32, 49943.65] 0.000
Sun 2019 42424.20 [34954.31, 49894.10] 0.000
Sun 2019 42422.05 [34952.09, 49892.00] 0.000
Li 2017 42315.04 [34845.29, 49784.78] 0.000
Li 2017 42312.07 [34842.61, 49781.54] 0.000
Nherera 2011 41637.09 [34169.42, 49104.76] 0.000
Li 2017 40353.32 [32886.06, 47820.57] 0.000
Li 2017 40350.07 [32883.21, 47816.94] 0.000
30000 40000 50000 60000 70000
Random-effects DerSimonian–Laird model
Sorted by: inmb

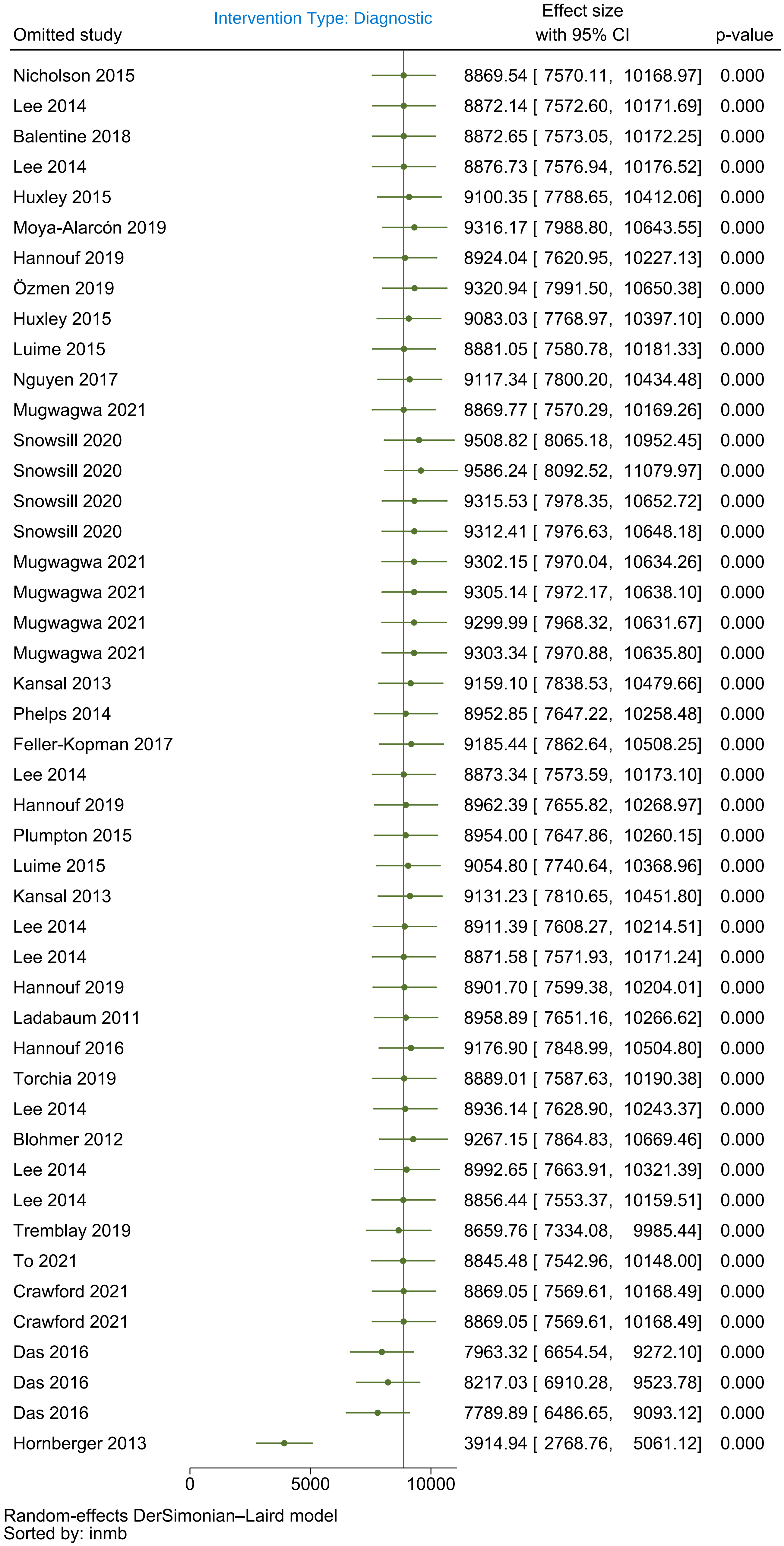
Omitted study
Intervention Type: Diagnostic
Effect size with 95% CI
p-value
Nicholson 2015 8869.54 [7570.11, 10168.97] 0.000
Lee 2014 8872.14 [7572.60, 10171.69] 0.000
Balentine 2018 8872.65 [7573.05, 10172.25] 0.000
Lee 2014 8876.73 [7576.94, 10176.52] 0.000
Huxley 2015 9100.35 [7788.65, 10412.06] 0.000
Moya-Alarcón 2019 9316.17 [7988.80, 10643.55] 0.000
Hannouf 2019 8924.04 [7620.95, 10227.13] 0.000
Özmen 2019 9320.94 [7991.50, 10650.38] 0.000
Huxley 2015 9083.03 [7768.97, 10397.10] 0.000
Luime 2015 8881.05 [7580.78, 10181.33] 0.000
Nguyen 2017 9117.34 [7800.20, 10434.48] 0.000
Mugwagwa 2021 8869.77 [7570.29, 10169.26] 0.000
Snowsill 2020 9508.82 [8065.18, 10952.45] 0.000
Snowsill 2020 9586.24 [8092.52, 11079.97] 0.000
Snowsill 2020 9315.53 [7978.35, 10652.72] 0.000
Snowsill 2020 9312.41 [7976.63, 10648.18] 0.000
Mugwagwa 2021 9302.15 [7970.04, 10634.26] 0.000
Mugwagwa 2021 9305.14 [7972.17, 10638.10] 0.000
Mugwagwa 2021 9299.99 [7968.32, 10631.67] 0.000
Mugwagwa 2021 9303.34 [7970.88, 10635.80] 0.000
Kansal 2013 9159.10 [7838.53, 10479.66] 0.000
Phelps 2014 8952.85 [7647.22, 10258.48] 0.000
Feller-Kopman 2017 9185.44 [7862.64, 10508.25] 0.000
Lee 2014 8873.34 [7573.59, 10173.10] 0.000
Hannouf 2019 8962.39 [7655.82, 10268.97] 0.000
Plumpton 2015 8954.00 [7647.86, 10260.15] 0.000
Luime 2015 9054.80 [7740.64, 10368.96] 0.000
Kansal 2013 9131.23 [7810.65, 10451.80] 0.000
Lee 2014 8911.39 [7608.27, 10214.51] 0.000
Lee 2014 8871.58 [7571.93, 10171.24] 0.000
Hannouf 2019 8901.70 [7599.38, 10204.01] 0.000
Ladabaum 2011 8958.89 [7651.16, 10266.62] 0.000
Hannouf 2016 9176.90 [7848.99, 10504.80] 0.000
Torchia 2019 8889.01 [7587.63, 10190.38] 0.000
Lee 2014 8936.14 [7628.90, 10243.37] 0.000
Blohmer 2012 9267.15 [7864.83, 10669.46] 0.000
Lee 2014 8992.65 [7663.91, 10321.39] 0.000
Lee 2014 8856.44 [7553.37, 10159.51] 0.000
Tremblay 2019 8659.76 [7334.08, 9985.44] 0.000
To 2021 8845.48 [7542.96, 10148.00] 0.000
Crawford 2021 8869.05 [7569.61, 10168.49] 0.000
Crawford 2021 8869.05 [7569.61, 10168.49] 0.000
Das 2016 7963.32 [6654.54, 9272.10] 0.000
Das 2016 8217.03 [6910.28, 9523.78] 0.000
Das 2016 7789.89 [6486.65, 9093.12] 0.000
Hornberger 2013 3914.94 [2768.76, 5061.12] 0.000
0
5000
10000
Random-effects DerSimonian–Laird model
Sorted by: inmb

Intervention Type: Prognostic

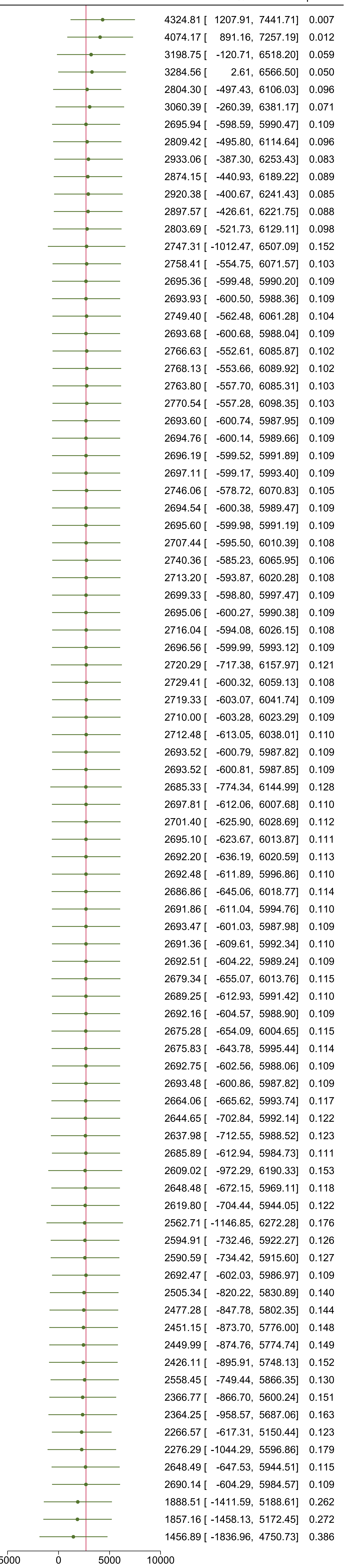

Omitted study	Effect size with 95% CI	p-value
Yang 2012	4324.81 [1207.91, 7441.71]	0.007
Yang 2012	4074.17 [891.16, 7257.19]	0.012
Marguet 2016	3198.75 [-120.71, 6518.20]	0.059
Zhu 2013	3284.56 [2.61, 6566.50]	0.050
Loong 2019	2804.30 [-497.43, 6106.03]	0.096
Aguiar 2017	3060.39 [-260.39, 6381.17]	0.071
Wallbillich 2016	2695.94 [-598.59, 5990.47]	0.109
Marguet 2016	2809.42 [-495.80, 6114.64]	0.096
Zhao 2016	2933.06 [-387.30, 6253.43]	0.083
Marguet 2016	2874.15 [-440.93, 6189.22]	0.089
Zhao 2016	2920.38 [-400.67, 6241.43]	0.085
Jahn 2017	2897.57 [-426.61, 6221.75]	0.088
Su 2021	2803.69 [-521.73, 6129.11]	0.098
Vriens 2014	2747.31 [-1012.47, 6507.09]	0.152
Govers 2013	2758.41 [-554.75, 6071.57]	0.103
Govers 2013	2695.36 [-599.48, 5990.20]	0.109
Doble 2017	2693.93 [-600.50, 5988.36]	0.109
Lobo 2017	2749.40 [-562.48, 6061.28]	0.104
Doble 2017	2693.68 [-600.68, 5988.04]	0.109
Naylor 2014	2766.63 [-552.61, 6085.87]	0.102
Paulden 2013	2768.13 [-553.66, 6089.92]	0.102
Bonastre 2014	2763.80 [-557.70, 6085.31]	0.103
Vriens 2014	2770.54 [-557.28, 6098.35]	0.103
Wu 2016	2693.60 [-600.74, 5987.95]	0.109
Lotan 2018	2694.76 [-600.14, 5989.66]	0.109
Hyle 2020	2696.19 [-599.52, 5991.89]	0.109
Biltaji 2021	2697.11 [-599.17, 5993.40]	0.109
Chandler 2018	2746.06 [-578.72, 6070.83]	0.105
Hao 2019	2694.54 [-600.38, 5989.47]	0.109
Biltaji 2021	2695.60 [-599.98, 5991.19]	0.109
Bonastre 2014	2707.44 [-595.50, 6010.39]	0.108
Ward 2013	2740.36 [-585.23, 6065.95]	0.106
Biltaji 2021	2713.20 [-593.87, 6020.28]	0.108
Biltaji 2021	2699.33 [-598.80, 5997.47]	0.109
Retel 2020	2695.06 [-600.27, 5990.38]	0.109
Marguet 2016	2716.04 [-594.08, 6026.15]	0.108
Lotan 2018	2696.56 [-599.99, 5993.12]	0.109
Bargallo-Rocha 2015	2720.29 [-717.38, 6157.97]	0.121
Ibarrondo 2020	2729.41 [-600.32, 6059.13]	0.108
Jongeneel 2021	2719.33 [-603.07, 6041.74]	0.109
Marguet 2016	2710.00 [-603.28, 6023.29]	0.109
Ontario Health (Quality) 2020	2712.48 [-613.05, 6038.01]	0.110
Retel 2020	2693.52 [-600.79, 5987.82]	0.109
Retel 2020	2693.52 [-600.81, 5987.85]	0.109
Eccleston 2017	2685.33 [-774.34, 6144.99]	0.128
Roth 2014	2697.81 [-612.06, 6007.68]	0.110
Ward 2013	2701.40 [-625.90, 6028.69]	0.112
Chang 2019	2695.10 [-623.67, 6013.87]	0.111
Ontario Health (Quality) 2020	2692.20 [-636.19, 6020.59]	0.113
Woods 2011	2692.48 [-611.89, 5996.86]	0.110
Roth 2015	2686.86 [-645.06, 6018.77]	0.114
Marguet 2016	2691.86 [-611.04, 5994.76]	0.110
Retel 2020	2693.47 [-601.03, 5987.98]	0.109
Marguet 2016	2691.36 [-609.61, 5992.34]	0.110
Woods 2011	2692.51 [-604.22, 5989.24]	0.109
Cressman 2016	2679.34 [-655.07, 6013.76]	0.115
Marguet 2016	2689.25 [-612.93, 5991.42]	0.110
Blank 2015	2692.16 [-604.57, 5988.90]	0.109
Ibarrondo 2020	2675.28 [-654.09, 6004.65]	0.115
Jongeneel 2021	2675.83 [-643.78, 5995.44]	0.114
Retel 2020	2692.75 [-602.56, 5988.06]	0.109
Retel 2020	2693.48 [-600.86, 5987.82]	0.109
Holt 2013	2664.06 [-665.62, 5993.74]	0.117
Li 2011	2644.65 [-702.84, 5992.14]	0.122
Ontario Health (Quality) 2020	2637.98 [-712.55, 5988.52]	0.123
Marguet 2016	2685.89 [-612.94, 5984.73]	0.111
Reed 2013	2609.02 [-972.29, 6190.33]	0.153
Hall 2012	2648.48 [-672.15, 5969.11]	0.118
Katz 2015	2619.80 [-704.44, 5944.05]	0.122
Ontario Health (Quality) 2020	2562.71 [-1146.85, 6272.28]	0.176
Katz 2015	2594.91 [-732.46, 5922.27]	0.126
Yamauchi 2014	2590.59 [-734.42, 5915.60]	0.127
Su 2021	2692.47 [-602.03, 5986.97]	0.109
Segui 2014	2505.34 [-820.22, 5830.89]	0.140
Marguet 2016	2477.28 [-847.78, 5802.35]	0.144
Lobo 2017	2451.15 [-873.70, 5776.00]	0.148
Jahn 2015	2449.99 [-874.76, 5774.74]	0.149
Marguet 2016	2426.11 [-895.91, 5748.13]	0.152
Lotan 2018	2558.45 [-749.44, 5866.35]	0.130
Retel 2013	2366.77 [-866.70, 5600.24]	0.151
Segui 2014	2364.25 [-958.57, 5687.06]	0.163
Rodríguez 2019	2266.57 [-617.31, 5150.44]	0.123
Marguet 2016	2276.29 [-1044.29, 5596.86]	0.179
Garrison Jr 2013	2648.49 [-647.53, 5944.51]	0.115
Garrison Jr 2013	2690.14 [-604.29, 5984.57]	0.109
Retèl 2012	1888.51 [-1411.59, 5188.61]	0.262
Retèl 2012	1857.16 [-1458.13, 5172.45]	0.272
Greeley 2011	1456.89 [-1836.96, 4750.73]	0.386

Random-effects DerSimonian–Laird model
Sorted by: inmb

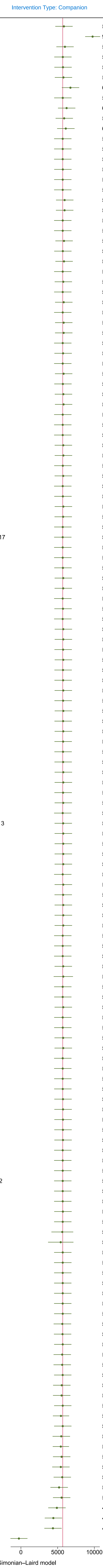

Omitted study	Effect size with 95% CI	p-value
Wu 2020	5855.77 [4710.27, 7001.26]	0.000
Blank 2011	9759.43 [8777.42, 10741.44]	0.000
Berm 2016	5997.66 [4851.52, 7143.79]	0.000
Wu 2020	5708.82 [4563.47, 6854.18]	0.000
Wu 2020	5727.01 [4581.57, 6872.46]	0.000
Wu 2020	5784.31 [4638.60, 6930.03]	0.000
Banerjee 2020	6745.99 [5597.62, 7894.36]	0.000
Wu 2020	5699.53 [4554.19, 6844.87]	0.000
Wu 2018	6223.10 [5073.90, 7372.31]	0.000
Wu 2018	5896.35 [4748.71, 7043.99]	0.000
Lauren 2020	6109.25 [4957.00, 7261.50]	0.000
Behl 2012	5693.40 [4548.10, 6838.70]	0.000
Behl 2012	5693.47 [4548.17, 6838.77]	0.000
Liu 2012	5693.73 [4548.42, 6839.04]	0.000
You 2012	5707.23 [4561.60, 6852.86]	0.000
Buchanan 2017	5696.33 [4550.95, 6841.71]	0.000
Harty 2018	5694.01 [4548.69, 6839.33]	0.000
Wu 2018	5956.84 [4804.28, 7109.41]	0.000
Cai 2019	5946.39 [4793.79, 7098.99]	0.000
Butzke 2015	5693.24 [4547.94, 6838.54]	0.000
Harty 2018	5693.21 [4547.91, 6838.51]	0.000
Wu 2018	5869.35 [4717.09, 7021.61]	0.000
Handorf 2012	5703.92 [4558.19, 6849.65]	0.000
Zhu 2021	5870.03 [4717.40, 7022.67]	0.000
Verhoef 2019	5693.27 [4547.97, 6838.58]	0.000
Criss 2019	5767.16 [4618.29, 6916.04]	0.000
Nelson 2013	5693.31 [4548.01, 6838.62]	0.000
Handorf 2012	5828.60 [4676.14, 6981.06]	0.000
Harty 2018	5693.63 [4548.30, 6838.95]	0.000
Alkhatib 2020	5823.14 [4670.30, 6975.98]	0.000
You 2019	5818.85 [4665.94, 6971.76]	0.000
Liu 2012	5693.88 [4548.54, 6839.22]	0.000
Crespin 2011	5737.48 [4589.46, 6885.50]	0.000
Romanus 2015	5693.29 [4547.98, 6838.59]	0.000
Saito 2019	5811.77 [4659.10, 6964.44]	0.000
Okere 2018	5714.02 [4567.37, 6860.67]	0.000
Dong 2015	5783.31 [4632.04, 6934.57]	0.000
Chong 2018	5805.80 [4661.94, 6949.67]	0.000
Limdi 2020	5694.47 [4549.09, 6839.86]	0.000
Pink 2014	5693.26 [4547.95, 6838.56]	0.000
Djalalov 2014	5693.77 [4548.42, 6839.11]	0.000
Yuliwulandari 2021	5770.26 [4618.15, 6922.36]	0.000
Goel 2018	5781.08 [4627.56, 6934.59]	0.000
Parthan 2013	5709.58 [4562.79, 6856.36]	0.000
Perlis 2012	5763.94 [4612.02, 6915.87]	0.000
Kapoor 2015	5693.22 [4547.92, 6838.52]	0.000
Kim 2019	5712.70 [4565.55, 6859.85]	0.000
Lu 2016	5774.46 [4621.39, 6927.52]	0.000
Dong 2015	5749.06 [4598.37, 6899.76]	0.000
Mitropoulou 2014	5702.00 [4555.85, 6848.15]	0.000
Martes-Martinez 2017	5694.41 [4548.99, 6839.82]	0.000
Lu 2018	5693.85 [4548.49, 6839.21]	0.000
Romanus 2015	5693.26 [4547.95, 6838.56]	0.000
Teng 2020	5717.47 [4569.80, 6865.14]	0.000
Thompson 2013	5763.03 [4610.91, 6915.15]	0.000
Dong 2015	5755.46 [4604.06, 6906.86]	0.000
Saito 2017	5693.25 [4547.95, 6838.56]	0.000
Alagoz 2016	5730.05 [4581.12, 6878.97]	0.000
Schackman 2013	5693.22 [4547.92, 6838.52]	0.000
Chong 2014	5769.69 [4616.83, 6922.56]	0.000
Chong 2014	5769.69 [4616.82, 6922.56]	0.000
Sluiter 2019	5746.32 [4595.75, 6896.89]	0.000
Plumpton 2017	5770.56 [4617.48, 6923.63]	0.000
Chen 2016	5736.15 [4586.55, 6885.74]	0.000
Saramago 2018	5747.07 [4596.36, 6897.77]	0.000
Duarte 2020	5764.79 [4612.31, 6917.27]	0.000
Saramago 2018	5746.57 [4595.92, 6897.23]	0.000
Saokaew 2014	5770.81 [4616.88, 6924.73]	0.000
Saramago 2018	5744.37 [4593.93, 6894.81]	0.000
Sutherland 2019	5768.18 [4615.34, 6921.02]	0.000
Saramago 2018	5746.61 [4595.94, 6897.28]	0.000
Ke 2017	5735.01 [4585.51, 6884.52]	0.000
Saramago 2018	5745.14 [4594.62, 6895.67]	0.000
Verhoef 2014	5768.24 [4615.38, 6921.10]	0.000
Rens 2020	5770.51 [4616.66, 6924.35]	0.000
Verhoef 2014	5737.89 [4588.08, 6887.69]	0.000
Verhoef 2016	5762.97 [4610.62, 6915.33]	0.000
Pink 2014	5693.30 [4548.00, 6838.61]	0.000
Nshimyumukiza 2013	5756.78 [4605.02, 6908.54]	0.000
Rens 2020	5768.79 [4615.70, 6921.87]	0.000
Kim 2017	5767.37 [4614.42, 6920.31]	0.000
Verhoef 2016	5764.67 [4612.00, 6917.33]	0.000
Kim 2021	5767.83 [4614.74, 6920.91]	0.000
Lieberthal 2013	5693.90 [4548.53, 6839.27]	0.000
Sluiter 2018	5767.74 [4614.63, 6920.86]	0.000
Dong 2012	5750.12 [4598.85, 6901.39]	0.000
Cai 2021	5767.37 [4613.60, 6921.14]	0.000
Rens 2020	5766.12 [4613.12, 6919.11]	0.000
Verhoef 2013	5766.51 [4613.33, 6919.69]	0.000
Kazi 2014	5693.37 [4548.05, 6838.69]	0.000
Schremser 2015	5695.35 [4549.82, 6840.88]	0.000
Nelson 2013	5693.30 [4547.99, 6838.61]	0.000
Li 2015	5760.94 [4608.12, 6913.77]	0.000
Chugh 2019	5766.71 [4488.09, 7045.32]	0.000
Butzke 2015	5693.29 [4547.98, 6838.60]	0.000
Wei 2019	5697.19 [4551.43, 6842.95]	0.000
Wang 2018	5759.33 [4605.76, 6912.90]	0.000
Okere 2018	5697.07 [4551.31, 6842.83]	0.000
Kazi 2014	5697.44 [4551.63, 6843.24]	0.000
Patel 2014	5747.56 [4595.78, 6899.33]	0.000
Chugh 2019	5758.51 [4600.75, 6916.27]	0.000
Wu 2016	5694.25 [4548.82, 6839.69]	0.000
Panattoni 2012	5693.21 [4547.91, 6838.51]	0.000
Jiang 2016	5696.80 [4550.95, 6842.65]	0.000
Jiang 2015	5701.63 [4554.94, 6848.32]	0.000
Permsuwan 2014	5725.65 [4574.89, 6876.41]	0.000
Rejon-Parrilla 2014	5734.79 [4582.11, 6887.47]	0.000
Dong 2019	5728.31 [4576.78, 6879.84]	0.000
Dong 2019	5721.56 [4570.94, 6872.17]	0.000
Lim 2016	5730.10 [4576.94, 6883.26]	0.000
Kim 2019	5696.12 [4550.11, 6842.13]	0.000
Kim 2019	5693.33 [4548.00, 6838.67]	0.000
Liu 2012	5693.36 [4548.01, 6838.70]	0.000
De Lima Lopes 2012	5693.70 [4548.25, 6839.15]	0.000
Jiang 2015	5694.43 [4548.75, 6840.11]	0.000
Kim 2019	5694.72 [4548.94, 6840.50]	0.000
Sorich 2013	5713.97 [4561.79, 6866.14]	0.000
You 2015	5693.72 [4548.19, 6839.25]	0.000
Jiang 2016	5632.74 [4180.92, 7084.57]	0.000
Jiang 2016	5416.55 [3723.01, 7110.09]	0.000
Nelson 2013	5693.21 [4547.89, 6838.52]	0.000
Jiang 2017	5691.42 [4538.22, 6844.62]	0.000
Buchanan 2017	5693.03 [4547.65, 6838.40]	0.000
Liu 2012	5692.15 [4546.64, 6837.66]	0.000
Groessl 2018	5692.80 [4547.42, 6838.18]	0.000
Elbasha 2016	5688.52 [4542.37, 6834.67]	0.000
You 2013	5691.67 [4546.15, 6837.20]	0.000
Tanner 2020	5641.95 [4481.35, 6802.55]	0.000
Panattoni 2012	5666.21 [4517.45, 6814.98]	0.000
You 2012	5690.68 [4545.12, 6836.24]	0.000
Narasimhalu 2020	5622.67 [4470.08, 6775.27]	0.000
Li 2015	5619.65 [4466.84, 6772.46]	0.000
Fawsitt 2020	5687.04 [4541.38, 6832.70]	0.000
Alkhatib 2018	5561.60 [4408.80, 6714.40]	0.000
Wei 2019	5543.04 [4390.67, 6695.42]	0.000
Hart 2019	5692.43 [4547.10, 6837.77]	0.000
Wei 2020	5451.14 [4381.52, 6520.76]	0.000
Schackman 2015	5692.61 [4547.29, 6837.93]	0.000
Alkhatib 2018	5493.79 [4342.01, 6645.57]	0.000
Hornberger 2015	5432.26 [4359.74, 6504.77]	0.000
Hart 2019	5541.46 [4390.90, 6692.02]	0.000
Pruis 2020	5434.90 [4283.49, 6586.31]	0.000
Pruis 2020	5627.31 [4480.55, 6774.08]	0.000
Choi 2019	5198.61 [4046.59, 6350.62]	0.000
Wu 2020	5538.60 [4391.78, 6685.43]	0.000
Chen 2018	4897.86 [3746.48, 6049.24]	0.000
Steuten 2019	4415.10 [3265.60, 5564.60]	0.000
Steuten 2019	4362.15 [3212.57, 5511.72]	0.000
Bock 2015	-277.13 [-1400.85, 846.58]	0.629

Random-effects DerSimonian–Laird model
Sorted by: inmb

Intervention Type: Gene Therapy

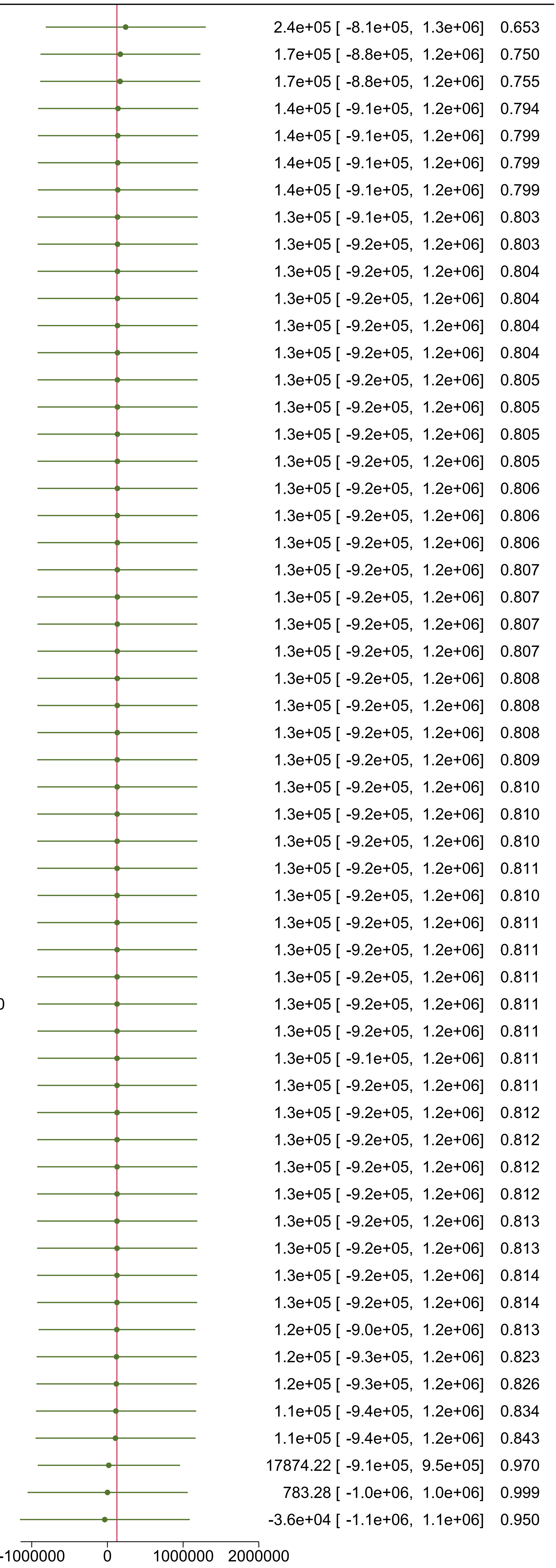

Omitted study	Effect size with 95% CI	p-value
Wherry 2020	2.4e+05 [-8.1e+05, 1.3e+06]	0.653
Broekhoff 2021	1.7e+05 [-8.8e+05, 1.2e+06]	0.750
Broekhoff 2021	1.7e+05 [-8.8e+05, 1.2e+06]	0.755
Uhrmann 2020	1.4e+05 [-9.1e+05, 1.2e+06]	0.794
Zimmermann 2018	1.4e+05 [-9.1e+05, 1.2e+06]	0.799
Viriato 2020	1.4e+05 [-9.1e+05, 1.2e+06]	0.799
South 2019	1.4e+05 [-9.1e+05, 1.2e+06]	0.799
Lin 2019	1.3e+05 [-9.1e+05, 1.2e+06]	0.803
Jiang 2021	1.3e+05 [-9.2e+05, 1.2e+06]	0.803
Lin 2018	1.3e+05 [-9.2e+05, 1.2e+06]	0.804
Lin 2018	1.3e+05 [-9.2e+05, 1.2e+06]	0.804
Lin 2019	1.3e+05 [-9.2e+05, 1.2e+06]	0.804
Lin 2019	1.3e+05 [-9.2e+05, 1.2e+06]	0.804
Furzer 2020	1.3e+05 [-9.2e+05, 1.2e+06]	0.805
Lin 2019	1.3e+05 [-9.2e+05, 1.2e+06]	0.805
Lin 2019	1.3e+05 [-9.2e+05, 1.2e+06]	0.805
Whittington 2019	1.3e+05 [-9.2e+05, 1.2e+06]	0.805
Lin 2019	1.3e+05 [-9.2e+05, 1.2e+06]	0.806
Cher 2020	1.3e+05 [-9.2e+05, 1.2e+06]	0.806
Curl 2014	1.3e+05 [-9.2e+05, 1.2e+06]	0.806
Whittington 2019	1.3e+05 [-9.2e+05, 1.2e+06]	0.807
Lin 2018	1.3e+05 [-9.2e+05, 1.2e+06]	0.807
Liu 2021	1.3e+05 [-9.2e+05, 1.2e+06]	0.807
Le 2021	1.3e+05 [-9.2e+05, 1.2e+06]	0.807
Whittington 2019	1.3e+05 [-9.2e+05, 1.2e+06]	0.808
Curl 2014	1.3e+05 [-9.2e+05, 1.2e+06]	0.808
Whittington 2019	1.3e+05 [-9.2e+05, 1.2e+06]	0.808
Holko 2014	1.3e+05 [-9.2e+05, 1.2e+06]	0.809
Whittington 2019	1.3e+05 [-9.2e+05, 1.2e+06]	0.810
Sarkar 2019	1.3e+05 [-9.2e+05, 1.2e+06]	0.810
Sax 2014	1.3e+05 [-9.2e+05, 1.2e+06]	0.810
Behl 2012	1.3e+05 [-9.2e+05, 1.2e+06]	0.811
Wakase 2021	1.3e+05 [-9.2e+05, 1.2e+06]	0.810
Liu 2021	1.3e+05 [-9.2e+05, 1.2e+06]	0.811
Ward 2017	1.3e+05 [-9.2e+05, 1.2e+06]	0.811
Lin 2018	1.3e+05 [-9.2e+05, 1.2e+06]	0.811
Ribera Santasusana 2020	1.3e+05 [-9.2e+05, 1.2e+06]	0.811
Zargar 2018	1.3e+05 [-9.2e+05, 1.2e+06]	0.811
Lu 2017	1.3e+05 [-9.1e+05, 1.2e+06]	0.811
Fu 2019	1.3e+05 [-9.2e+05, 1.2e+06]	0.811
Li 2021	1.3e+05 [-9.2e+05, 1.2e+06]	0.812
Lieberthal 2013	1.3e+05 [-9.2e+05, 1.2e+06]	0.812
AlMukdad 2021	1.3e+05 [-9.2e+05, 1.2e+06]	0.812
AlMukdad 2021	1.3e+05 [-9.2e+05, 1.2e+06]	0.812
Retel 2018	1.3e+05 [-9.2e+05, 1.2e+06]	0.813
Dan 2014	1.3e+05 [-9.2e+05, 1.2e+06]	0.813
Dan 2014	1.3e+05 [-9.2e+05, 1.2e+06]	0.814
Whittington 2018	1.3e+05 [-9.2e+05, 1.2e+06]	0.814
Wakase 2021	1.2e+05 [-9.0e+05, 1.2e+06]	0.813
Moradi-Lakeh 2021	1.2e+05 [-9.3e+05, 1.2e+06]	0.823
South 2019	1.2e+05 [-9.3e+05, 1.2e+06]	0.826
Machin 2018	1.1e+05 [-9.4e+05, 1.2e+06]	0.834
Johnson 2019	1.1e+05 [-9.4e+05, 1.2e+06]	0.843
Bolous 2021	17874.22 [-9.1e+05, 9.5e+05]	0.970
Cook 2020	783.28 [-1.0e+06, 1.0e+06]	0.999
Bolous 2021	-3.6e+04 [-1.1e+06, 1.1e+06]	0.950

Random-effects DerSimonian–Laird model
Sorted by: inmb

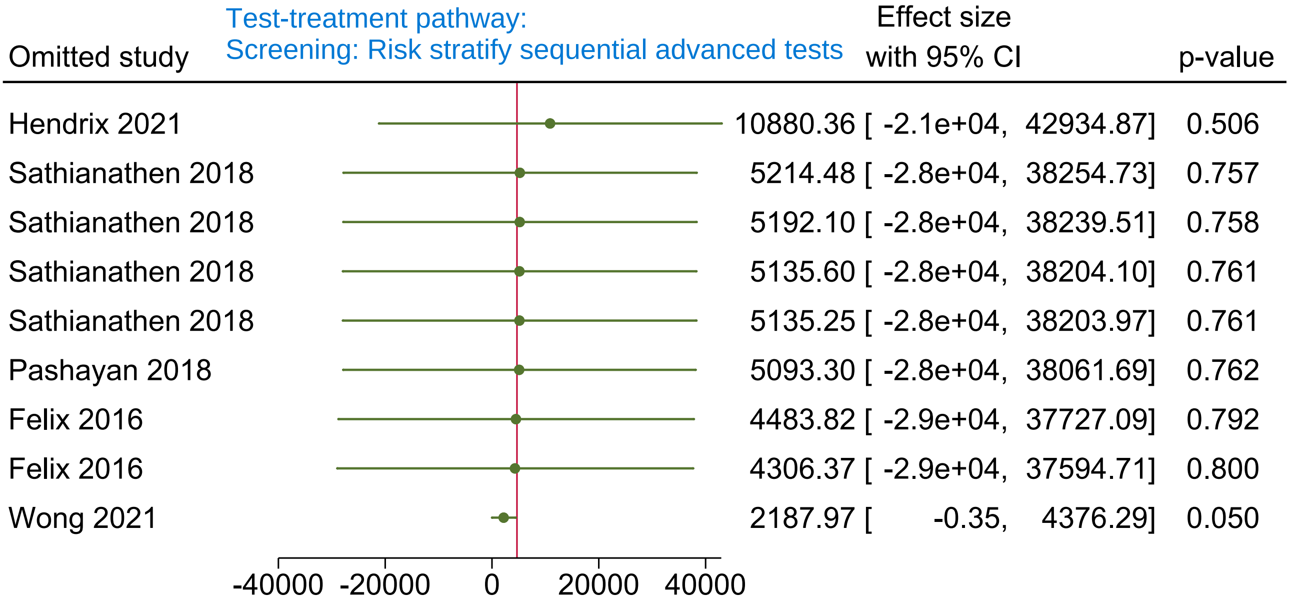

Random-effects DerSimonian–Laird model
Sorted by: inmb

Test-treatment pathway:
Screening: Minimize the likelihood of conditions occurring later in life

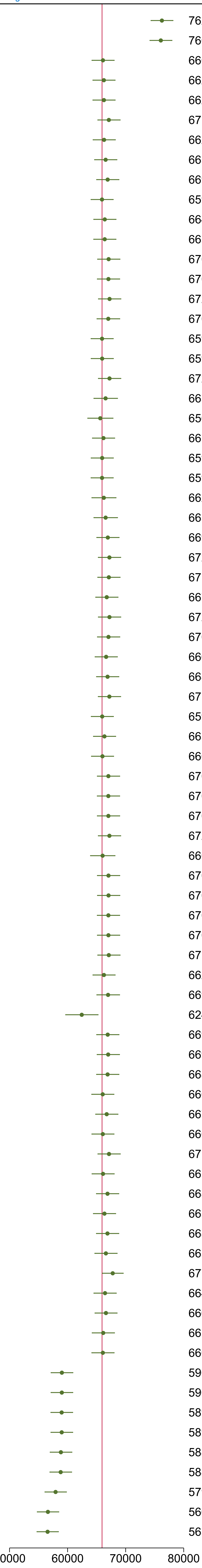

Omitted study	Effect size with 95% CI	p-value
Asti 2021	76242.89 [74368.34, 78117.45]	0.000
Asti 2021	76059.77 [74183.59, 77935.95]	0.000
Wang 2012	66093.57 [64197.17, 67989.97]	0.000
Wang 2012	66251.91 [64353.21, 68150.62]	0.000
Wang 2012	66259.92 [64361.10, 68158.75]	0.000
Chen 2015	67110.98 [65200.78, 69021.19]	0.000
Wang 2012	66292.57 [64393.20, 68191.95]	0.000
Sun 2019	66554.95 [64652.05, 68457.83]	0.000
Perez 2011	66905.80 [64998.19, 68813.41]	0.000
Sun 2019	65932.35 [64037.78, 67826.92]	0.000
Wang 2012	66407.66 [64506.59, 68308.73]	0.000
Wang 2012	66397.18 [64496.23, 68298.12]	0.000
Ladabaum 2014	67076.19 [65165.94, 68986.44]	0.000
Ladabaum 2013	67037.62 [65127.67, 68947.56]	0.000
Lázaro 2017	67228.73 [65315.35, 69142.12]	0.000
Azardoost 2021	67007.93 [65061.20, 68954.66]	0.000
McKay 2018	65935.87 [64041.25, 67830.49]	0.000
McKay 2018	65954.45 [64059.56, 67849.34]	0.000
Chen 2011	67221.39 [65307.58, 69135.21]	0.000
Azardoost 2021	66549.20 [64533.58, 68564.80]	0.000
Azardoost 2021	65628.24 [63466.53, 67789.97]	0.000
McKay 2018	66179.95 [64281.84, 68078.06]	0.000
Guzauskas 2020	65956.01 [64061.10, 67850.92]	0.000
McKay 2018	65939.51 [64044.83, 67834.18]	0.000
Azardoost 2021	66245.46 [64182.91, 68308.01]	0.000
Azardoost 2021	66551.46 [64536.36, 68566.56]	0.000
Gray 2017	66932.61 [65023.74, 68841.47]	0.000
Guzauskas 2020	67204.62 [65291.70, 69117.54]	0.000
Compagni 2013	67107.03 [65195.64, 69018.41]	0.000
Severin 2015	66749.84 [64843.56, 68656.11]	0.000
Chen 2016	67217.38 [65303.63, 69131.12]	0.000
Walter 2017	67049.28 [65138.71, 68959.85]	0.000
Pedersen 2016	66644.17 [64739.40, 68548.95]	0.000
McKay 2018	66889.70 [64981.41, 68797.99]	0.000
Dymond 2020	67197.97 [65285.15, 69110.79]	0.000
McKay 2018	65968.29 [64073.20, 67863.38]	0.000
Gray 2017	66351.07 [64450.49, 68251.66]	0.000
McKay 2018	66004.74 [64109.13, 67900.35]	0.000
Gray 2017	67031.63 [65121.27, 68941.97]	0.000
Gray 2017	67021.79 [65111.58, 68932.00]	0.000
Forde 2016	67029.34 [65119.01, 68939.66]	0.000
Djalalov 2012	67203.80 [65290.76, 69116.83]	0.000
de Graaff 2017	66036.98 [63942.68, 68131.28]	0.000
Walter 2017	67044.95 [65134.37, 68955.52]	0.000
Walter 2017	67057.95 [65147.17, 68968.72]	0.000
Gray 2017	67025.77 [65115.46, 68936.09]	0.000
Gray 2017	67041.48 [65130.91, 68952.04]	0.000
Kapoor 2020	67103.46 [65191.99, 69014.93]	0.000
Sun 2019	66265.16 [64365.77, 68164.54]	0.000
GoodSmith 2019	66980.31 [65032.14, 68928.48]	0.000
Correa-Galendi 2021	62440.09 [59663.08, 65217.11]	0.000
Huh 2015	66913.77 [65004.97, 68822.57]	0.000
Forde 2016	66998.09 [65088.06, 68908.12]	0.000
Sun 2019	66898.75 [64990.11, 68807.38]	0.000
Ghatnekar 2013	66051.62 [64155.30, 67947.94]	0.000
Catchpool 2019	66738.45 [64831.94, 68644.96]	0.000
Hoskins 2019	66073.86 [64177.12, 67970.60]	0.000
Jung 2021	67132.05 [65217.93, 69046.16]	0.000
Müller 2019	66104.12 [64206.77, 68001.46]	0.000
Kwon 2018	66879.38 [64969.23, 68789.53]	0.000
Wang 2012	66347.95 [64446.15, 68249.75]	0.000
Rubio-Terres 2015	66874.23 [64963.24, 68785.20]	0.000
Perez 2011	66599.00 [64691.11, 68506.89]	0.000
Ademi 2014	67773.56 [65995.32, 69551.81]	0.000
Kwon 2018	66444.09 [64534.24, 68353.95]	0.000
Cromwell 2016	66604.77 [64717.50, 68492.05]	0.000
Lipton 2020	66142.11 [64237.33, 68046.89]	0.000
Lipton 2020	66080.99 [64176.53, 67985.46]	0.000
Sun 2019	59019.58 [57160.58, 60878.57]	0.000
Sun 2019	59005.57 [57147.60, 60863.54]	0.000
Sun 2019	58983.40 [57122.99, 60843.82]	0.000
Sun 2019	58990.07 [57128.98, 60851.16]	0.000
Li 2017	58853.74 [56994.81, 60712.68]	0.000
Li 2017	58814.52 [56958.63, 60670.41]	0.000
Nherera 2011	57936.21 [56099.07, 59773.34]	0.000
Li 2017	56614.41 [54781.21, 58447.61]	0.000
Li 2017	56559.92 [54730.96, 58388.89]	0.000

Random-effects DerSimonian–Laird model
Sorted by: inmb

Omitted study	Test-treatment pathway: Early-stage diagnostics	Effect size with 95% CI	p-value
Nicholson 2015		8869.54 [7570.11, 10168.97]	0.000
Lee 2014		8872.14 [7572.60, 10171.69]	0.000
Balentine 2018		8872.65 [7573.05, 10172.25]	0.000
Lee 2014		8876.73 [7576.94, 10176.52]	0.000
Huxley 2015		9100.35 [7788.65, 10412.06]	0.000
Moya-Alarcón 2019		9316.17 [7988.80, 10643.55]	0.000
Hannouf 2019		8924.04 [7620.95, 10227.13]	0.000
Özmen 2019		9320.94 [7991.50, 10650.38]	0.000
Huxley 2015		9083.03 [7768.97, 10397.10]	0.000
Luime 2015		8881.05 [7580.78, 10181.33]	0.000
Nguyen 2017		9117.34 [7800.20, 10434.48]	0.000
Mugwagwa 2021		8869.77 [7570.29, 10169.26]	0.000
Snowsill 2020		9508.82 [8065.18, 10952.45]	0.000
Snowsill 2020		9586.24 [8092.52, 11079.97]	0.000
Snowsill 2020		9315.53 [7978.35, 10652.72]	0.000
Snowsill 2020		9312.41 [7976.63, 10648.18]	0.000
Mugwagwa 2021		9302.15 [7970.04, 10634.26]	0.000
Mugwagwa 2021		9305.14 [7972.17, 10638.10]	0.000
Mugwagwa 2021		9299.99 [7968.32, 10631.67]	0.000
Mugwagwa 2021		9303.34 [7970.88, 10635.80]	0.000
Kansal 2013		9159.10 [7838.53, 10479.66]	0.000
Phelps 2014		8952.85 [7647.22, 10258.48]	0.000
Feller-Kopman 2017		9185.44 [7862.64, 10508.25]	0.000
Lee 2014		8873.34 [7573.59, 10173.10]	0.000
Hannouf 2019		8962.39 [7655.82, 10268.97]	0.000
Plumpton 2015		8954.00 [7647.86, 10260.15]	0.000
Luime 2015		9054.80 [7740.64, 10368.96]	0.000
Kansal 2013		9131.23 [7810.65, 10451.80]	0.000
Lee 2014		8911.39 [7608.27, 10214.51]	0.000
Lee 2014		8871.58 [7571.93, 10171.24]	0.000
Hannouf 2019		8901.70 [7599.38, 10204.01]	0.000
Ladabaum 2011		8958.89 [7651.16, 10266.62]	0.000
Hannouf 2016		9176.90 [7848.99, 10504.80]	0.000
Torchia 2019		8889.01 [7587.63, 10190.38]	0.000
Lee 2014		8936.14 [7628.90, 10243.37]	0.000
Blohmer 2012		9267.15 [7864.83, 10669.46]	0.000
Lee 2014		8992.65 [7663.91, 10321.39]	0.000
Lee 2014		8856.44 [7553.37, 10159.51]	0.000
Tremblay 2019		8659.76 [7334.08, 9985.44]	0.000
To 2021		8845.48 [7542.96, 10148.00]	0.000
Crawford 2021		8869.05 [7569.61, 10168.49]	0.000
Crawford 2021		8869.05 [7569.61, 10168.49]	0.000
Das 2016		7963.32 [6654.54, 9272.10]	0.000
Das 2016		8217.03 [6910.28, 9523.78]	0.000
Das 2016		7789.89 [6486.65, 9093.12]	0.000
Hornberger 2013		3914.94 [2768.76, 5061.12]	0.000

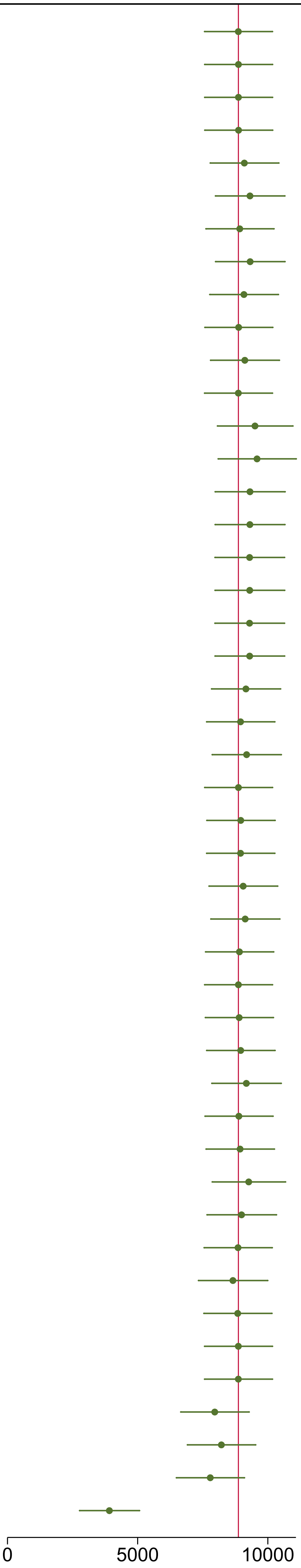

Random-effects DerSimonian–Laird model
Sorted by: inmb

Test-treatment pathway:
Prognostic: Initiate the use of preventive or advanced therapies

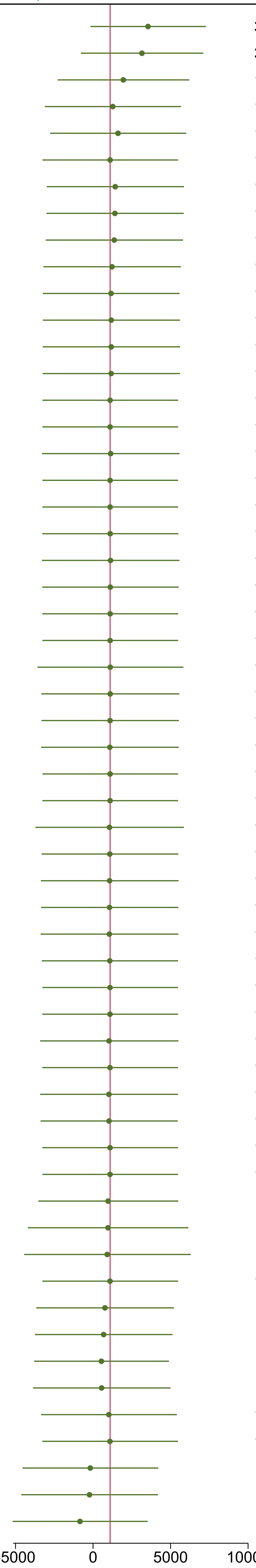

Omitted study	Effect size with 95% CI	p-value
Yang 2012	3546.99 [-131.54, 7225.51]	0.059
Yang 2012	3156.09 [-748.41, 7060.59]	0.113
Zhu 2013	1950.38 [-2250.73, 6151.49]	0.363
Loong 2019	1272.68 [-3073.86, 5619.22]	0.566
Aguiar 2017	1611.72 [-2734.67, 5958.11]	0.467
Wallbillich 2016	1103.21 [-3227.58, 5434.00]	0.618
Zhao 2016	1431.74 [-2951.04, 5814.53]	0.522
Zhao 2016	1410.36 [-2973.68, 5794.40]	0.528
Jahn 2017	1368.23 [-3021.14, 5757.61]	0.541
Su 2021	1223.52 [-3169.02, 5616.06]	0.585
Lobo 2017	1161.84 [-3205.46, 5529.14]	0.602
Naylor 2014	1175.98 [-3205.32, 5557.27]	0.599
Paulden 2013	1174.01 [-3212.01, 5560.02]	0.600
Bonastre 2014	1167.79 [-3217.70, 5553.29]	0.602
Hyle 2020	1101.66 [-3231.79, 5435.12]	0.618
Biltaji 2021	1102.29 [-3232.46, 5437.03]	0.618
Chandler 2018	1136.13 [-3255.67, 5527.92]	0.612
Hao 2019	1100.14 [-3231.56, 5431.84]	0.619
Biltaji 2021	1100.86 [-3232.31, 5434.04]	0.619
Bonastre 2014	1108.68 [-3240.52, 5457.88]	0.617
Ward 2013	1126.75 [-3266.77, 5520.27]	0.615
Biltaji 2021	1111.42 [-3246.32, 5469.17]	0.617
Biltaji 2021	1103.00 [-3235.85, 5441.84]	0.618
Retel 2020	1100.36 [-3232.24, 5432.95]	0.619
Bargallo-Rocha 2015	1112.63 [-3551.16, 5776.42]	0.640
Ibarrondo 2020	1109.17 [-3294.10, 5512.45]	0.622
Jongeneel 2021	1097.88 [-3289.44, 5485.19]	0.624
Ontario Health (Quality) 2020	1084.21 [-3309.42, 5477.83]	0.629
Retel 2020	1099.40 [-3230.89, 5429.69]	0.619
Retel 2020	1099.37 [-3230.98, 5429.72]	0.619
Eccleston 2017	1062.96 [-3681.76, 5807.68]	0.661
Roth 2014	1082.02 [-3281.35, 5445.39]	0.627
Ward 2013	1066.64 [-3331.24, 5464.52]	0.635
Chang 2019	1065.11 [-3315.44, 5445.66]	0.634
Ontario Health (Quality) 2020	1052.52 [-3348.29, 5453.34]	0.639
Woods 2011	1081.58 [-3270.61, 5433.78]	0.626
Retel 2020	1098.99 [-3231.76, 5429.74]	0.619
Woods 2011	1093.68 [-3242.06, 5429.42]	0.621
Cressman 2016	1033.50 [-3384.15, 5451.14]	0.647
Blank 2015	1093.07 [-3242.68, 5428.82]	0.621
Ibarrondo 2020	1026.77 [-3377.07, 5430.62]	0.648
Jongeneel 2021	1033.99 [-3348.16, 5416.15]	0.644
Retel 2020	1096.41 [-3236.16, 5428.98]	0.620
Retel 2020	1099.28 [-3231.09, 5429.65]	0.619
Ontario Health (Quality) 2020	973.22 [-3496.32, 5442.77]	0.670
Reed 2013	966.71 [-4161.73, 6095.14]	0.712
Ontario Health (Quality) 2020	917.65 [-4408.29, 6243.59]	0.736
Su 2021	1097.26 [-3233.47, 5428.00]	0.619
Segui 2014	767.65 [-3628.31, 5163.61]	0.732
Jahn 2015	683.18 [-3711.72, 5078.07]	0.761
Retel 2013	540.82 [-3764.08, 4845.73]	0.806
Segui 2014	552.20 [-3838.29, 4942.70]	0.805
Garrison Jr 2013	1019.29 [-3314.86, 5353.44]	0.645
Garrison Jr 2013	1093.36 [-3237.22, 5423.93]	0.621
Retèl 2012	-177.05 [-4511.93, 4157.84]	0.936
Retèl 2012	-226.94 [-4596.89, 4143.00]	0.919
Greeley 2011	-839.88 [-5154.06, 3474.31]	0.703

Random-effects DerSimonian–Laird model
Sorted by: inmb

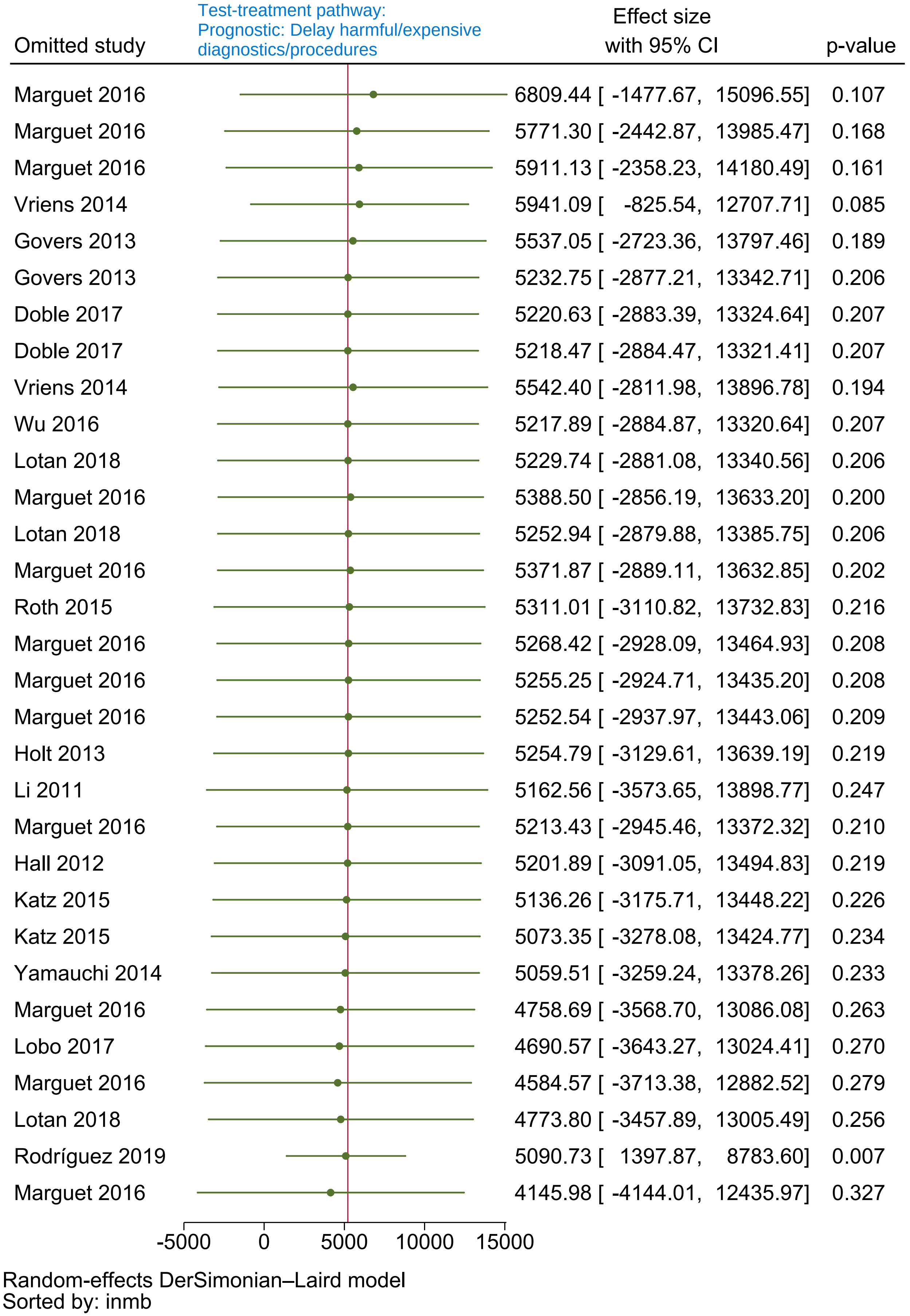

Omitted study
Test-treatment pathway:
Prognostic: Delay harmful/expensive diagnostics/procedures
Effect size with 95% CI
p-value
Marguet 2016 6809.44 [-1477.67, 15096.55] 0.107
Marguet 2016 5771.30 [-2442.87, 13985.47] 0.168
Marguet 2016 5911.13 [-2358.23, 14180.49] 0.161
Vriens 2014 5941.09 [-825.54, 12707.71] 0.085
Govers 2013 5537.05 [-2723.36, 13797.46] 0.189
Govers 2013 5232.75 [-2877.21, 13342.71] 0.206
Doble 2017 5220.63 [-2883.39, 13324.64] 0.207
Doble 2017 5218.47 [-2884.47, 13321.41] 0.207
Vriens 2014 5542.40 [-2811.98, 13896.78] 0.194
Wu 2016 5217.89 [-2884.87, 13320.64] 0.207
Lotan 2018 5229.74 [-2881.08, 13340.56] 0.206
Marguet 2016 5388.50 [-2856.19, 13633.20] 0.200
Lotan 2018 5252.94 [-2879.88, 13385.75] 0.206
Marguet 2016 5371.87 [-2889.11, 13632.85] 0.202
Roth 2015 5311.01 [-3110.82, 13732.83] 0.216
Marguet 2016 5268.42 [-2928.09, 13464.93] 0.208
Marguet 2016 5255.25 [-2924.71, 13435.20] 0.208
Marguet 2016 5252.54 [-2937.97, 13443.06] 0.209
Holt 2013 5254.79 [-3129.61, 13639.19] 0.219
Li 2011 5162.56 [-3573.65, 13898.77] 0.247
Marguet 2016 5213.43 [-2945.46, 13372.32] 0.210
Hall 2012 5201.89 [-3091.05, 13494.83] 0.219
Katz 2015 5136.26 [-3175.71, 13448.22] 0.226
Katz 2015 5073.35 [-3278.08, 13424.77] 0.234
Yamauchi 2014 5059.51 [-3259.24, 13378.26] 0.233
Marguet 2016 4758.69 [-3568.70, 13086.08] 0.263
Lobo 2017 4690.57 [-3643.27, 13024.41] 0.270
Marguet 2016 4584.57 [-3713.38, 12882.52] 0.279
Lotan 2018 4773.80 [-3457.89, 13005.49] 0.256
Rodríguez 2019 5090.73 [1397.87, 8783.60] 0.007
Marguet 2016 4145.98 [-4144.01, 12435.97] 0.327
-5000
0
5000
10000
15000
Random-effects DerSimonian–Laird model
Sorted by: inmb

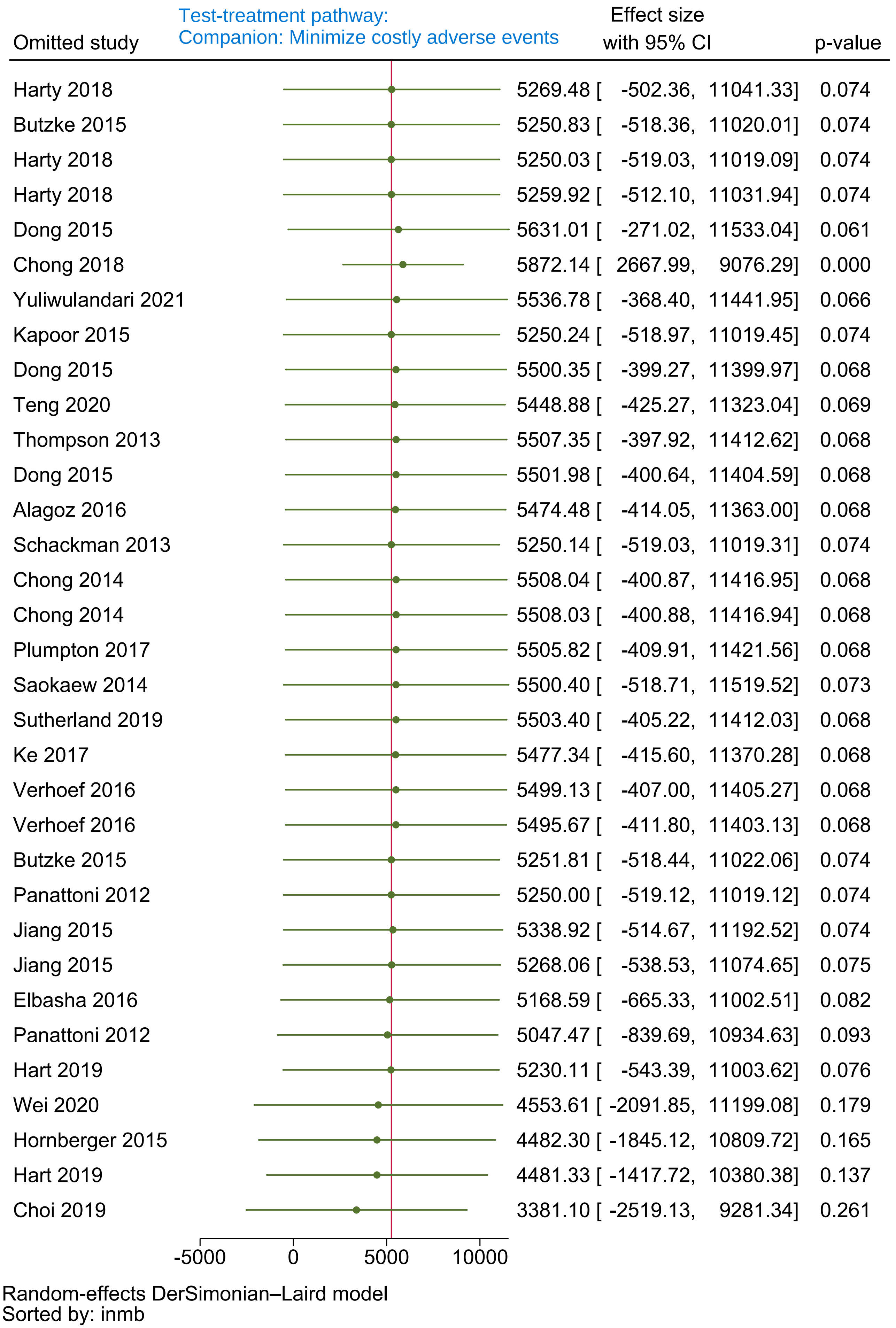

Test-treatment pathway:
Companion: Minimize costly adverse events
Omitted study
Effect size with 95% CI
p-value
Harty 2018 5269.48 [-502.36, 11041.33] 0.074
Butzke 2015 5250.83 [-518.36, 11020.01] 0.074
Harty 2018 5250.03 [-519.03, 11019.09] 0.074
Harty 2018 5259.92 [-512.10, 11031.94] 0.074
Dong 2015 5631.01 [-271.02, 11533.04] 0.061
Chong 2018 5872.14 [2667.99, 9076.29] 0.000
Yuliwulandari 2021 5536.78 [-368.40, 11441.95] 0.066
Kapoor 2015 5250.24 [-518.97, 11019.45] 0.074
Dong 2015 5500.35 [-399.27, 11399.97] 0.068
Teng 2020 5448.88 [-425.27, 11323.04] 0.069
Thompson 2013 5507.35 [-397.92, 11412.62] 0.068
Dong 2015 5501.98 [-400.64, 11404.59] 0.068
Alagoz 2016 5474.48 [-414.05, 11363.00] 0.068
Schackman 2013 5250.14 [-519.03, 11019.31] 0.074
Chong 2014 5508.04 [-400.87, 11416.95] 0.068
Chong 2014 5508.03 [-400.88, 11416.94] 0.068
Plumpton 2017 5505.82 [-409.91, 11421.56] 0.068
Saokaew 2014 5500.40 [-518.71, 11519.52] 0.073
Sutherland 2019 5503.40 [-405.22, 11412.03] 0.068
Ke 2017 5477.34 [-415.60, 11370.28] 0.068
Verhoef 2016 5499.13 [-407.00, 11405.27] 0.068
Verhoef 2016 5495.67 [-411.80, 11403.13] 0.068
Butzke 2015 5251.81 [-518.44, 11022.06] 0.074
Panattoni 2012 5250.00 [-519.12, 11019.12] 0.074
Jiang 2015 5338.92 [-514.67, 11192.52] 0.074
Jiang 2015 5268.06 [-538.53, 11074.65] 0.075
Elbasha 2016 5168.59 [-665.33, 11002.51] 0.082
Panattoni 2012 5047.47 [-839.69, 10934.63] 0.093
Hart 2019 5230.11 [-543.39, 11003.62] 0.076
Wei 2020 4553.61 [-2091.85, 11199.08] 0.179
Hornberger 2015 4482.30 [-1845.12, 10809.72] 0.165
Hart 2019 4481.33 [-1417.72, 10380.38] 0.137
Choi 2019 3381.10 [-2519.13, 9281.34] 0.261
-5000
0
5000
10000
Random-effects DerSimonian–Laird model
Sorted by: inmb

Omitted study	Test-treatment pathway: Companion: Optimize drug responses	Effect size with 95% CI	p-value
Wu 2020		5583.39 [4501.22, 6665.56]	0.000
Blank 2011		10172.65 [9384.42, 10960.88]	0.000
Berm 2016		5720.09 [4637.36, 6802.82]	0.000
Wu 2020		5450.70 [4368.59, 6532.82]	0.000
Wu 2020		5467.07 [4384.88, 6549.26]	0.000
Wu 2020		5519.50 [4437.09, 6601.92]	0.000
Banerjee 2020		6861.00 [5776.41, 7945.59]	0.000
Wu 2020		5442.40 [4360.29, 6524.50]	0.000
Wu 2018		6016.26 [4930.21, 7102.31]	0.000
Wu 2018		5639.25 [4554.97, 6723.52]	0.000
Lauren 2020		5997.95 [4907.10, 7088.80]	0.000
Behl 2012		5436.93 [4354.86, 6519.01]	0.000
Behl 2012		5436.99 [4354.92, 6519.07]	0.000
Liu 2012		5437.22 [4355.14, 6519.31]	0.000
You 2012		5449.34 [4366.98, 6531.69]	0.000
Buchanan 2017		5439.53 [4357.38, 6521.67]	0.000
Wu 2018		5790.99 [4699.63, 6882.34]	0.000
Cai 2019		5777.75 [4686.33, 6869.18]	0.000
Wu 2018		5665.20 [4574.46, 6755.94]	0.000
Handorf 2012		5446.34 [4363.90, 6528.78]	0.000
Zhu 2021		5672.30 [4580.84, 6763.76]	0.000
Verhoef 2019		5436.82 [4354.75, 6518.89]	0.000
Criss 2019		5514.18 [4428.49, 6599.87]	0.000
Nelson 2013		5436.86 [4354.78, 6518.93]	0.000
Handorf 2012		5613.55 [4522.44, 6704.65]	0.000
Alkhatib 2020		5610.80 [4518.96, 6702.65]	0.000
You 2019		5605.74 [4513.77, 6697.71]	0.000
Liu 2012		5437.35 [4355.24, 6519.45]	0.000
Crespin 2011		5480.73 [4396.03, 6565.43]	0.000
Romanus 2015		5436.83 [4354.75, 6518.91]	0.000
Saito 2019		5593.14 [4501.64, 6684.64]	0.000
Okere 2018		5456.00 [4372.71, 6539.29]	0.000
Limdi 2020		5437.86 [4355.71, 6520.01]	0.000
Pink 2014		5436.80 [4354.73, 6518.88]	0.000
Djalalov 2014		5437.24 [4355.13, 6519.35]	0.000
Goel 2018		5554.08 [4461.86, 6646.29]	0.000
Parthan 2013		5451.81 [4368.40, 6535.23]	0.000
Perlis 2012		5524.06 [4433.93, 6614.19]	0.000
Kim 2019		5454.98 [4371.20, 6538.76]	0.000
Lu 2016		5544.91 [4452.70, 6637.13]	0.000
Mitropoulou 2014		5444.58 [4361.76, 6527.40]	0.000
Martes-Martinez 2017		5437.79 [4355.62, 6519.96]	0.000
Lu 2018		5437.31 [4355.19, 6519.43]	0.000
Romanus 2015		5436.80 [4354.73, 6518.88]	0.000
Saito 2017		5436.80 [4354.72, 6518.88]	0.000
Sluiter 2019		5496.71 [4408.75, 6584.66]	0.000
Chen 2016		5482.47 [4395.87, 6569.06]	0.000
Saramago 2018		5498.02 [4409.87, 6586.17]	0.000
Duarte 2020		5528.24 [4437.10, 6619.37]	0.000
Saramago 2018		5497.27 [4409.19, 6585.35]	0.000
Saramago 2018		5494.02 [4406.25, 6581.78]	0.000
Saramago 2018		5497.35 [4409.26, 6585.44]	0.000
Saramago 2018		5495.16 [4407.29, 6583.04]	0.000
Verhoef 2014		5535.17 [4443.31, 6627.04]	0.000
Rens 2020		5540.41 [4447.67, 6633.15]	0.000
Verhoef 2014		5484.89 [4398.01, 6571.77]	0.000
Pink 2014		5436.84 [4354.76, 6518.93]	0.000
Nshimyumukiza 2013		5514.07 [4424.23, 6603.91]	0.000
Rens 2020		5537.10 [4444.86, 6629.34]	0.000
Kim 2017		5534.46 [4442.44, 6626.47]	0.000
Kim 2021		5535.75 [4443.51, 6627.99]	0.000
Lieberthal 2013		5437.35 [4355.22, 6519.49]	0.000
Sluiter 2018		5535.73 [4443.46, 6628.00]	0.000
Dong 2012		5503.70 [4414.67, 6592.72]	0.000
Cai 2021		5536.13 [4443.37, 6628.90]	0.000
Rens 2020		5533.01 [4440.90, 6625.12]	0.000
Verhoef 2013		5534.16 [4441.82, 6626.50]	0.000
Kazi 2014		5436.90 [4354.81, 6518.99]	0.000
Schremser 2015		5438.60 [4356.34, 6520.87]	0.000
Nelson 2013		5436.84 [4354.76, 6518.92]	0.000
Li 2015		5524.97 [4433.17, 6616.76]	0.000
Chugh 2019		5710.94 [4519.80, 6902.08]	0.000
Wei 2019		5440.21 [4357.74, 6522.67]	0.000
Wang 2018		5524.87 [4432.16, 6617.57]	0.000
Okere 2018		5440.10 [4357.63, 6522.57]	0.000
Kazi 2014		5440.42 [4357.91, 6522.93]	0.000
Patel 2014		5502.39 [4412.52, 6592.27]	0.000
Chugh 2019		5530.47 [4434.06, 6626.88]	0.000
Wu 2016		5437.65 [4355.46, 6519.83]	0.000
Jiang 2016		5439.82 [4357.28, 6522.37]	0.000
Permsuwan 2014		5472.61 [4384.37, 6560.84]	0.000
Rejon-Parrilla 2014		5488.68 [4397.17, 6580.19]	0.000
Dong 2019		5477.32 [4387.86, 6566.77]	0.000
Dong 2019		5467.52 [4379.50, 6555.53]	0.000
Lim 2016		5483.53 [4391.18, 6575.87]	0.000
Kim 2019		5439.15 [4356.46, 6521.85]	0.000
Kim 2019		5436.86 [4354.76, 6518.96]	0.000
Liu 2012		5436.88 [4354.77, 6518.99]	0.000
De Lima Lopes 2012		5437.14 [4354.94, 6519.34]	0.000
Kim 2019		5437.94 [4355.46, 6520.43]	0.000
Sorich 2013		5459.92 [4369.36, 6550.49]	0.000
You 2015		5437.13 [4354.86, 6519.40]	0.000
Jiang 2016		5787.43 [4351.69, 7223.18]	0.000
Jiang 2016		5702.73 [4054.84, 7350.61]	0.000
Nelson 2013		5436.75 [4354.67, 6518.84]	0.000
Jiang 2017		5429.80 [4337.40, 6522.19]	0.000
Buchanan 2017		5436.57 [4354.44, 6518.71]	0.000
Liu 2012		5435.72 [4353.47, 6517.97]	0.000
Groessl 2018		5436.37 [4354.23, 6518.51]	0.000
You 2013		5435.29 [4353.02, 6517.55]	0.000
Tanner 2020		5350.16 [4260.57, 6439.74]	0.000
You 2012		5434.37 [4352.07, 6516.67]	0.000
Narasimhalu 2020		5337.12 [4245.76, 6428.47]	0.000
Li 2015		5331.26 [4239.49, 6423.02]	0.000
Fawsitt 2020		5431.02 [4348.63, 6513.40]	0.000
Alkhatib 2018		5248.64 [4156.87, 6340.40]	0.000
Wei 2019		5220.23 [4129.24, 6311.21]	0.000
Schackman 2015		5436.22 [4354.13, 6518.31]	0.000
Alkhatib 2018		5149.61 [4059.59, 6239.62]	0.000
Pruis 2020		5110.30 [4021.04, 6199.56]	0.000
Pruis 2020		5373.01 [4289.61, 6456.40]	0.000
Wu 2020		5287.41 [4203.96, 6370.85]	0.000
Chen 2018		4376.27 [3286.97, 5465.58]	0.000
Steuten 2019		3712.31 [2626.03, 4798.59]	0.000
Steuten 2019		3654.46 [2568.08, 4740.83]	0.000
Bock 2015		-2043.37 [-3087.12, -999.62]	0.000

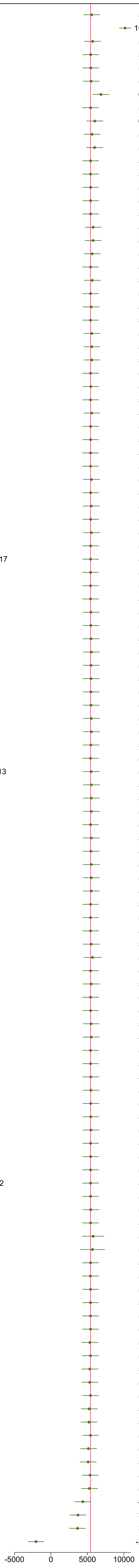

Random-effects DerSimonian–Laird model
Sorted by: inmb

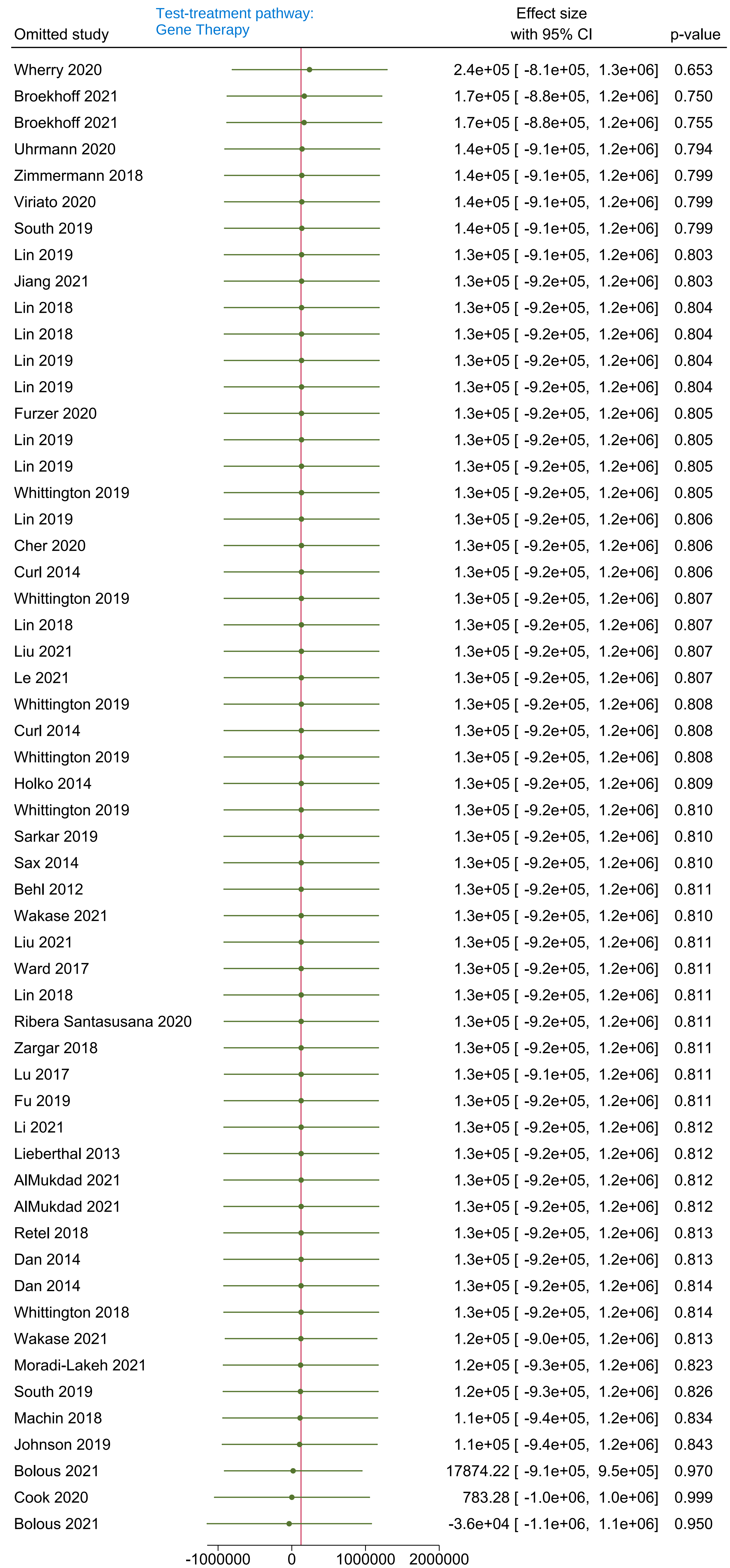

Random-effects DerSimonian–Laird model
Sorted by: inmb

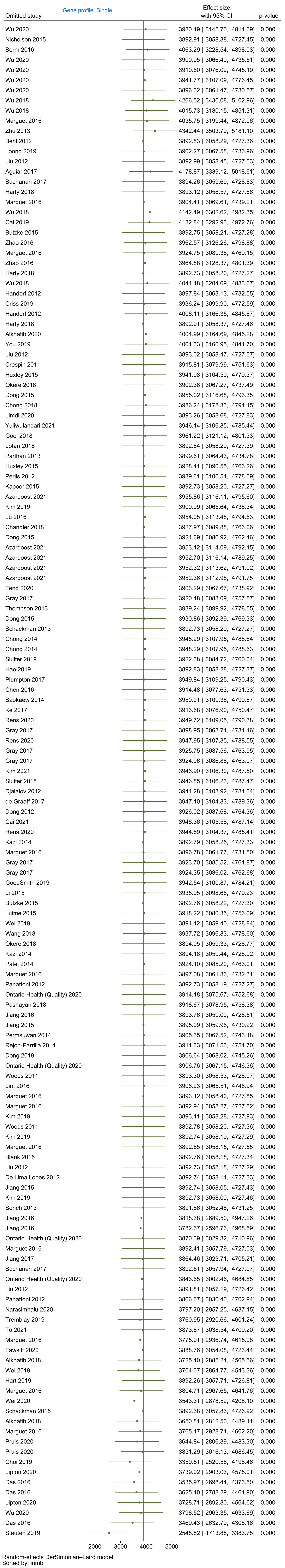
Gene profile: Single
Omitted study
Effect size with 95% CI
p-value
Wu 2020 3980.19 [3145.70, 4814.69] 0.000
Nicholson 2015 3892.91 [3058.38, 4727.45] 0.000
Berm 2016 4063.29 [3228.54, 4898.03] 0.000
Wu 2020 3900.95 [3066.40, 4735.51] 0.000
Wu 2020 3910.60 [3076.02, 4745.19] 0.000
Wu 2020 3941.77 [3107.09, 4776.45] 0.000
Wu 2020 3896.02 [3061.47, 4730.57] 0.000
Wu 2018 4266.52 [3430.08, 5102.96] 0.000
Wu 2018 4015.73 [3180.15, 4851.31] 0.000
Marguet 2016 4035.75 [3199.44, 4872.06] 0.000
Zhu 2013 4342.44 [3503.79, 5181.10] 0.000
Behl 2012 3892.83 [3058.29, 4727.36] 0.000
Loong 2019 3902.27 [3067.58, 4736.96] 0.000
Liu 2012 3892.99 [3058.45, 4727.53] 0.000
Aguiar 2017 4178.87 [3339.12, 5018.61] 0.000
Buchanan 2017 3894.26 [3059.69, 4728.83] 0.000
Harty 2018 3893.12 [3058.57, 4727.66] 0.000
Marguet 2016 3904.41 [3069.61, 4739.21] 0.000
Wu 2018 4142.49 [3302.62, 4982.35] 0.000
Cai 2019 4132.84 [3292.93, 4972.76] 0.000
Butzke 2015 3892.75 [3058.21, 4727.28] 0.000
Zhao 2016 3962.57 [3126.26, 4798.88] 0.000
Marguet 2016 3924.75 [3089.36, 4760.15] 0.000
Zhao 2016 3964.88 [3128.37, 4801.39] 0.000
Harty 2018 3892.73 [3058.20, 4727.27] 0.000
Wu 2018 4044.18 [3204.69, 4883.67] 0.000
Handorf 2012 3897.84 [3063.13, 4732.55] 0.000
Criss 2019 3936.24 [3099.90, 4772.59] 0.000
Handorf 2012 4006.11 [3166.35, 4845.87] 0.000
Harty 2018 3892.91 [3058.37, 4727.46] 0.000
Alkhatib 2020 4004.99 [3164.69, 4845.28] 0.000
You 2019 4001.33 [3160.95, 4841.70] 0.000
Liu 2012 3893.02 [3058.47, 4727.57] 0.000
Crespin 2011 3915.81 [3079.99, 4751.63] 0.000
Huxley 2015 3941.98 [3104.59, 4779.37] 0.000
Okere 2018 3902.38 [3067.27, 4737.49] 0.000
Dong 2015 3955.02 [3116.68, 4793.35] 0.000
Chong 2018 3986.24 [3178.33, 4794.15] 0.000
Limdi 2020 3893.26 [3058.68, 4727.83] 0.000
Yuliwulandari 2021 3946.14 [3106.85, 4785.44] 0.000
Goel 2018 3961.22 [3121.12, 4801.33] 0.000
Lotan 2018 3892.84 [3058.29, 4727.39] 0.000
Parthan 2013 3899.61 [3064.43, 4734.78] 0.000
Huxley 2015 3928.41 [3090.55, 4766.26] 0.000
Perlis 2012 3939.61 [3100.54, 4778.69] 0.000
Kapoor 2015 3892.73 [3058.20, 4727.27] 0.000
Azardoost 2021 3955.86 [3116.11, 4795.60] 0.000
Kim 2019 3900.99 [3065.64, 4736.34] 0.000
Lu 2016 3954.05 [3113.48, 4794.63] 0.000
Chandler 2018 3927.97 [3089.88, 4766.06] 0.000
Dong 2015 3924.69 [3086.92, 4762.46] 0.000
Azardoost 2021 3953.12 [3114.09, 4792.15] 0.000
Azardoost 2021 3952.70 [3116.14, 4789.25] 0.000
Azardoost 2021 3952.32 [3113.62, 4791.02] 0.000
Azardoost 2021 3952.36 [3112.98, 4791.75] 0.000
Teng 2020 3903.29 [3067.67, 4738.92] 0.000
Gray 2017 3920.48 [3083.09, 4757.87] 0.000
Thompson 2013 3939.24 [3099.92, 4778.55] 0.000
Dong 2015 3930.86 [3092.39, 4769.33] 0.000
Schackman 2013 3892.73 [3058.20, 4727.27] 0.000
Chong 2014 3948.29 [3107.95, 4788.64] 0.000
Chong 2014 3948.29 [3107.95, 4788.63] 0.000
Sluiter 2019 3922.38 [3084.72, 4760.04] 0.000
Hao 2019 3892.83 [3058.28, 4727.37] 0.000
Plumpton 2017 3949.84 [3109.25, 4790.43] 0.000
Chen 2016 3914.48 [3077.63, 4751.33] 0.000
Saokaew 2014 3950.01 [3109.36, 4790.67] 0.000
Ke 2017 3913.68 [3076.90, 4750.47] 0.000
Rens 2020 3949.72 [3109.05, 4790.38] 0.000
Gray 2017 3898.95 [3063.74, 4734.16] 0.000
Rens 2020 3947.95 [3107.35, 4788.55] 0.000
Gray 2017 3925.75 [3087.56, 4763.95] 0.000
Gray 2017 3924.96 [3086.86, 4763.07] 0.000
Kim 2021 3946.90 [3106.30, 4787.50] 0.000
Sluiter 2018 3946.85 [3106.23, 4787.47] 0.000
Djalalov 2012 3944.28 [3103.92, 4784.64] 0.000
de Graaff 2017 3947.10 [3104.83, 4789.36] 0.000
Dong 2012 3926.02 [3087.68, 4764.36] 0.000
Cai 2021 3946.36 [3105.58, 4787.14] 0.000
Rens 2020 3944.89 [3104.37, 4785.41] 0.000
Kazi 2014 3892.79 [3058.25, 4727.33] 0.000
Marguet 2016 3896.78 [3061.77, 4731.80] 0.000
Gray 2017 3923.70 [3085.52, 4761.87] 0.000
Gray 2017 3924.35 [3086.02, 4762.68] 0.000
GoodSmith 2019 3942.54 [3100.87, 4784.21] 0.000
Li 2015 3938.95 [3098.66, 4779.23] 0.000
Butzke 2015 3892.76 [3058.22, 4727.30] 0.000
Luime 2015 3918.22 [3080.35, 4756.09] 0.000
Wei 2019 3894.12 [3059.40, 4728.84] 0.000
Wang 2018 3937.72 [3096.83, 4778.60] 0.000
Okere 2018 3894.05 [3059.33, 4728.77] 0.000
Kazi 2014 3894.18 [3059.44, 4728.92] 0.000
Patel 2014 3924.10 [3085.20, 4763.01] 0.000
Marguet 2016 3897.08 [3061.86, 4732.31] 0.000
Panattoni 2012 3892.73 [3058.19, 4727.27] 0.000
Ontario Health (Quality) 2020 3914.18 [3075.67, 4752.68] 0.000
Pashayan 2018 3918.67 [3078.95, 4758.38] 0.000
Jiang 2016 3893.76 [3059.00, 4728.51] 0.000
Jiang 2015 3895.09 [3059.96, 4730.22] 0.000
Permsuwan 2014 3905.35 [3067.52, 4743.18] 0.000
Rejon-Parrilla 2014 3911.63 [3071.56, 4751.70] 0.000
Dong 2019 3906.64 [3068.02, 4745.26] 0.000
Ontario Health (Quality) 2020 3906.76 [3067.15, 4746.36] 0.000
Woods 2011 3893.30 [3058.53, 4728.07] 0.000
Lim 2016 3906.23 [3065.51, 4746.94] 0.000
Marguet 2016 3893.12 [3058.40, 4727.85] 0.000
Marguet 2016 3892.94 [3058.27, 4727.62] 0.000
Kim 2019 3893.11 [3058.28, 4727.93] 0.000
Woods 2011 3892.78 [3058.20, 4727.36] 0.000
Kim 2019 3892.74 [3058.19, 4727.29] 0.000
Marguet 2016 3892.85 [3058.15, 4727.55] 0.000
Blank 2015 3892.76 [3058.18, 4727.34] 0.000
Liu 2012 3892.73 [3058.18, 4727.29] 0.000
De Lima Lopes 2012 3892.74 [3058.14, 4727.33] 0.000
Jiang 2015 3892.74 [3058.05, 4727.43] 0.000
Kim 2019 3892.73 [3058.00, 4727.46] 0.000
Sorich 2013 3891.86 [3052.48, 4731.25] 0.000
Jiang 2016 3818.38 [2689.50, 4947.26] 0.000
Jiang 2016 3782.67 [2596.76, 4968.59] 0.000
Ontario Health (Quality) 2020 3870.39 [3029.82, 4710.96] 0.000
Marguet 2016 3892.41 [3057.79, 4727.03] 0.000
Jiang 2017 3864.46 [3023.71, 4705.21] 0.000
Buchanan 2017 3892.51 [3057.94, 4727.07] 0.000
Ontario Health (Quality) 2020 3843.65 [3002.46, 4684.85] 0.000
Liu 2012 3891.81 [3057.19, 4726.42] 0.000
Panattoni 2012 3866.67 [3030.40, 4702.94] 0.000
Narasimhalu 2020 3797.20 [2957.25, 4637.15] 0.000
Tremblay 2019 3760.95 [2920.66, 4601.24] 0.000
To 2021 3873.87 [3038.54, 4709.20] 0.000
Marguet 2016 3775.91 [2936.74, 4615.08] 0.000
Fawsitt 2020 3888.76 [3054.08, 4723.44] 0.000
Alkhatib 2018 3725.40 [2885.24, 4565.56] 0.000
Wei 2019 3704.07 [2864.77, 4543.36] 0.000
Hart 2019 3892.26 [3057.71, 4726.81] 0.000
Marguet 2016 3804.71 [2967.65, 4641.76] 0.000
Wei 2020 3543.31 [2878.52, 4208.10] 0.000
Schackman 2015 3892.38 [3057.83, 4726.92] 0.000
Alkhatib 2018 3650.81 [2812.50, 4489.11] 0.000
Marguet 2016 3765.47 [2928.74, 4602.20] 0.000
Pruis 2020 3644.84 [2806.39, 4483.30] 0.000
Pruis 2020 3851.29 [3016.13, 4686.45] 0.000
Choi 2019 3359.51 [2520.56, 4198.46] 0.000
Lipton 2020 3739.02 [2903.03, 4575.01] 0.000
Das 2016 3535.97 [2698.44, 4373.50] 0.000
Das 2016 3625.10 [2788.29, 4461.90] 0.000
Lipton 2020 3728.71 [2892.80, 4564.62] 0.000
Wu 2020 3798.52 [2963.35, 4633.69] 0.000
Das 2016 3469.43 [2632.70, 4306.16] 0.000
Steuten 2019 2548.82 [1713.88, 3383.75] 0.000
2000
3000
4000
5000
Random-effects DerSimonian–Laird model
Sorted by: inmb

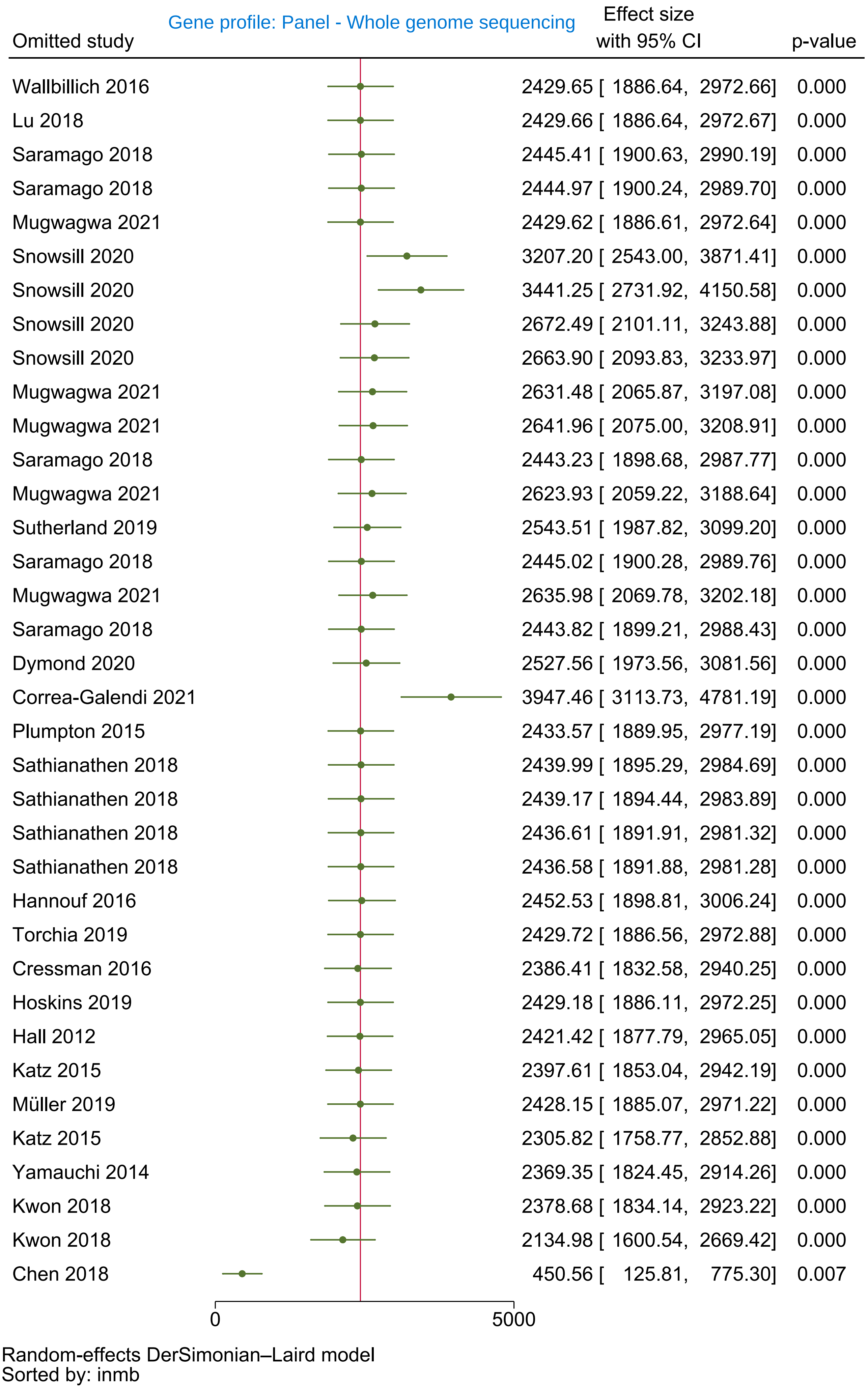
Gene profile: Panel - Whole genome sequencing
Omitted study
Effect size with 95% CI
p-value
Wallbillich 2016 2429.65 [1886.64, 2972.66] 0.000
Lu 2018 2429.66 [1886.64, 2972.67] 0.000
Saramago 2018 2445.41 [1900.63, 2990.19] 0.000
Saramago 2018 2444.97 [1900.24, 2989.70] 0.000
Mugwagwa 2021 2429.62 [1886.61, 2972.64] 0.000
Snowsill 2020 3207.20 [2543.00, 3871.41] 0.000
Snowsill 2020 3441.25 [2731.92, 4150.58] 0.000
Snowsill 2020 2672.49 [2101.11, 3243.88] 0.000
Snowsill 2020 2663.90 [2093.83, 3233.97] 0.000
Mugwagwa 2021 2631.48 [2065.87, 3197.08] 0.000
Mugwagwa 2021 2641.96 [2075.00, 3208.91] 0.000
Saramago 2018 2443.23 [1898.68, 2987.77] 0.000
Mugwagwa 2021 2623.93 [2059.22, 3188.64] 0.000
Sutherland 2019 2543.51 [1987.82, 3099.20] 0.000
Saramago 2018 2445.02 [1900.28, 2989.76] 0.000
Mugwagwa 2021 2635.98 [2069.78, 3202.18] 0.000
Saramago 2018 2443.82 [1899.21, 2988.43] 0.000
Dymond 2020 2527.56 [1973.56, 3081.56] 0.000
Correa-Galendi 2021 3947.46 [3113.73, 4781.19] 0.000
Plumpton 2015 2433.57 [1889.95, 2977.19] 0.000
Sathianathen 2018 2439.99 [1895.29, 2984.69] 0.000
Sathianathen 2018 2439.17 [1894.44, 2983.89] 0.000
Sathianathen 2018 2436.61 [1891.91, 2981.32] 0.000
Sathianathen 2018 2436.58 [1891.88, 2981.28] 0.000
Hannouf 2016 2452.53 [1898.81, 3006.24] 0.000
Torchia 2019 2429.72 [1886.56, 2972.88] 0.000
Cressman 2016 2386.41 [1832.58, 2940.25] 0.000
Hoskins 2019 2429.18 [1886.11, 2972.25] 0.000
Hall 2012 2421.42 [1877.79, 2965.05] 0.000
Katz 2015 2397.61 [1853.04, 2942.19] 0.000
Müller 2019 2428.15 [1885.07, 2971.22] 0.000
Katz 2015 2305.82 [1758.77, 2852.88] 0.000
Yamauchi 2014 2369.35 [1824.45, 2914.26] 0.000
Kwon 2018 2378.68 [1834.14, 2923.22] 0.000
Kwon 2018 2134.98 [1600.54, 2669.42] 0.000
Chen 2018 450.56 [125.81, 775.30] 0.007
0
5000
Random-effects DerSimonian–Laird model
Sorted by: inmb

Gene profile: Panel - Whole exome sequencing

Omitted study	Effect size with 95% CI	p-value
Guzauskas 2020	-3.49 [-1783.98, 1777.00]	0.997
Guzauskas 2020	2129.19 [-7879.51, 12137.89]	0.677
Catchpool 2019	-73.04 [-1881.78, 1735.71]	0.937
Crawford 2021	-3.67 [-1783.88, 1776.53]	0.997
Crawford 2021	-3.67 [-1783.88, 1776.53]	0.997

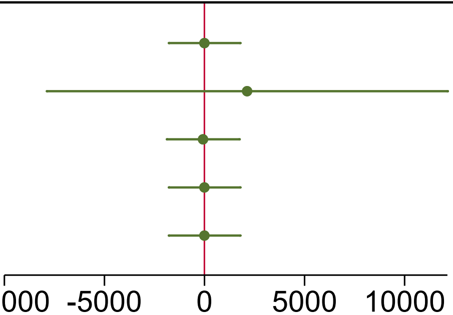

Random-effects DerSimonian–Laird model
Sorted by: inmb

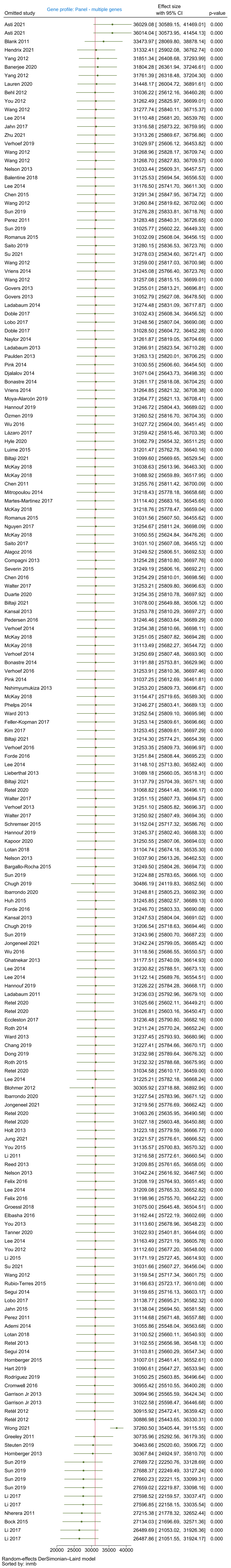
Omitted study
Gene profile: Panel - multiple genes
Effect size with 95% CI
p-value
Asti 2021 36029.08 [30589.15, 41469.01] 0.000
Asti 2021 36014.04 [30573.95, 41454.13] 0.000
Blank 2011 33473.97 [28069.80, 38878.14] 0.000
Hendrix 2021 31332.41 [25902.08, 36762.74] 0.000
Yang 2012 31851.34 [26408.68, 37293.99] 0.000
Banerjee 2020 31804.28 [26361.94, 37246.61] 0.000
Yang 2012 31761.39 [26318.48, 37204.30] 0.000
Lauren 2020 31448.17 [26004.72, 36891.61] 0.000
Behl 2012 31036.22 [25612.16, 36460.28] 0.000
You 2012 31262.49 [25825.97, 36699.01] 0.000
Wang 2012 31277.74 [25840.11, 36715.37] 0.000
Lee 2014 31110.48 [25681.20, 36539.76] 0.000
Jahn 2017 31316.58 [25873.22, 36759.95] 0.000
Zhu 2021 31313.26 [25869.67, 36756.86] 0.000
Verhoef 2019 31029.97 [25606.12, 36453.82] 0.000
Wang 2012 31268.96 [25828.17, 36709.74] 0.000
Wang 2012 31268.70 [25827.83, 36709.57] 0.000
Nelson 2013 31033.44 [25609.31, 36457.57] 0.000
Balentine 2018 31125.53 [25694.54, 36556.53] 0.000
Lee 2014 31176.50 [25741.70, 36611.30] 0.000
Chen 2015 31291.34 [25847.95, 36734.72] 0.000
Wang 2012 31260.84 [25819.62, 36702.06] 0.000
Sun 2019 31276.28 [25833.81, 36718.76] 0.000
Perez 2011 31283.48 [25840.31, 36726.65] 0.000
Sun 2019 31025.77 [25602.22, 36449.33] 0.000
Romanus 2015 31032.09 [25608.04, 36456.15] 0.000
Saito 2019 31280.15 [25836.53, 36723.76] 0.000
Su 2021 31278.03 [25834.60, 36721.47] 0.000
Wang 2012 31259.00 [25817.03, 36700.98] 0.000
Vriens 2014 31245.08 [25766.40, 36723.76] 0.000
Wang 2012 31257.08 [25815.15, 36699.01] 0.000
Govers 2013 31255.01 [25813.21, 36696.81] 0.000
Govers 2013 31052.79 [25627.08, 36478.50] 0.000
Ladabaum 2014 31274.48 [25831.09, 36717.87] 0.000
Doble 2017 31032.43 [25608.34, 36456.52] 0.000
Lobo 2017 31248.56 [25807.04, 36690.08] 0.000
Doble 2017 31028.50 [25604.72, 36452.28] 0.000
Naylor 2014 31261.87 [25819.05, 36704.69] 0.000
Ladabaum 2013 31266.91 [25823.54, 36710.28] 0.000
Paulden 2013 31263.13 [25820.01, 36706.25] 0.000
Pink 2014 31030.55 [25606.60, 36454.50] 0.000
Djalalov 2014 31071.04 [25643.73, 36498.35] 0.000
Bonastre 2014 31261.17 [25818.08, 36704.25] 0.000
Vriens 2014 31264.85 [25821.32, 36708.38] 0.000
Moya-Alarcón 2019 31264.77 [25821.13, 36708.41] 0.000
Hannouf 2019 31246.72 [25804.43, 36689.02] 0.000
Özmen 2019 31260.52 [25816.70, 36704.35] 0.000
Wu 2016 31027.72 [25604.00, 36451.45] 0.000
Lázaro 2017 31259.42 [25815.46, 36703.38] 0.000
Hyle 2020 31082.79 [25654.32, 36511.25] 0.000
Luime 2015 31201.47 [25762.78, 36640.16] 0.000
Biltaji 2021 31099.60 [25669.65, 36529.54] 0.000
McKay 2018 31038.63 [25613.96, 36463.30] 0.000
McKay 2018 31088.92 [25659.89, 36517.95] 0.000
Chen 2011 31255.76 [25811.42, 36700.09] 0.000
Mitropoulou 2014 31218.43 [25778.18, 36658.68] 0.000
Martes-Martinez 2017 31114.40 [25683.16, 36545.65] 0.000
McKay 2018 31218.76 [25778.47, 36659.04] 0.000
Romanus 2015 31031.56 [25607.50, 36455.62] 0.000
Nguyen 2017 31254.67 [25811.24, 36698.09] 0.000
McKay 2018 31050.55 [25624.84, 36476.26] 0.000
Saito 2017 31031.10 [25607.08, 36455.12] 0.000
Alagoz 2016 31249.52 [25806.51, 36692.53] 0.000
Compagni 2013 31254.28 [25810.80, 36697.76] 0.000
Severin 2015 31249.19 [25806.16, 36692.21] 0.000
Chen 2016 31254.29 [25810.01, 36698.56] 0.000
Walter 2017 31253.21 [25809.80, 36696.63] 0.000
Duarte 2020 31254.35 [25810.78, 36697.92] 0.000
Biltaji 2021 31078.00 [25649.88, 36506.12] 0.000
Kansal 2013 31253.78 [25810.29, 36697.27] 0.000
Pedersen 2016 31246.46 [25803.64, 36689.29] 0.000
Verhoef 2014 31254.38 [25810.66, 36698.11] 0.000
McKay 2018 31251.05 [25807.82, 36694.28] 0.000
McKay 2018 31113.49 [25682.27, 36544.72] 0.000
Verhoef 2014 31250.69 [25807.48, 36693.90] 0.000
Bonastre 2014 31191.88 [25753.81, 36629.96] 0.000
Verhoef 2016 31253.91 [25810.36, 36697.46] 0.000
Pink 2014 31037.25 [25612.69, 36461.81] 0.000
Nshimyumukiza 2013 31253.20 [25809.73, 36696.67] 0.000
McKay 2018 31154.47 [25719.65, 36589.30] 0.000
Phelps 2014 31246.27 [25803.41, 36689.13] 0.000
Ward 2013 31252.54 [25809.10, 36695.98] 0.000
Feller-Kopman 2017 31253.14 [25809.61, 36696.66] 0.000
Kim 2017 31253.45 [25809.61, 36697.29] 0.000
Biltaji 2021 31214.30 [25774.21, 36654.39] 0.000
Verhoef 2016 31253.35 [25809.73, 36696.97] 0.000
Forde 2016 31251.84 [25808.44, 36695.23] 0.000
Lee 2014 31148.10 [25713.80, 36582.40] 0.000
Lieberthal 2013 31089.18 [25660.05, 36518.31] 0.000
Biltaji 2021 31137.79 [25704.39, 36571.18] 0.000
Retel 2020 31068.82 [25641.48, 36496.17] 0.000
Walter 2017 31251.15 [25807.73, 36694.57] 0.000
Verhoef 2013 31251.10 [25805.82, 36696.37] 0.000
Walter 2017 31250.92 [25807.49, 36694.35] 0.000
Schremser 2015 31152.04 [25717.32, 36586.76] 0.000
Hannouf 2019 31245.37 [25802.40, 36688.33] 0.000
Kapoor 2020 31250.55 [25807.06, 36694.03] 0.000
Lotan 2018 31104.74 [25674.18, 36535.30] 0.000
Nelson 2013 31037.90 [25613.26, 36462.53] 0.000
Bargallo-Rocha 2015 31249.50 [25804.26, 36694.73] 0.000
Sun 2019 31224.88 [25783.65, 36666.10] 0.000
Chugh 2019 30486.19 [24119.83, 36852.56] 0.000
Ibarrondo 2020 31248.81 [25805.23, 36692.39] 0.000
Huh 2015 31245.85 [25802.57, 36689.13] 0.000
Forde 2016 31246.70 [25803.33, 36690.08] 0.000
Kansal 2013 31247.53 [25804.04, 36691.02] 0.000
Chugh 2019 31206.54 [25718.63, 36694.46] 0.000
Sun 2019 31243.96 [25800.70, 36687.23] 0.000
Jongeneel 2021 31242.24 [25799.05, 36685.42] 0.000
Wu 2016 31118.56 [25686.55, 36550.57] 0.000
Ghatnekar 2013 31177.51 [25740.09, 36614.93] 0.000
Lee 2014 31230.82 [25788.51, 36673.13] 0.000
Lee 2014 31122.14 [25689.76, 36554.51] 0.000
Hannouf 2019 31226.22 [25784.28, 36668.17] 0.000
Ladabaum 2011 31236.03 [25792.96, 36679.10] 0.000
Retel 2020 31025.66 [25602.11, 36449.21] 0.000
Retel 2020 31026.81 [25603.16, 36450.47] 0.000
Eccleston 2017 31236.48 [25790.80, 36682.16] 0.000
Roth 2014 31211.24 [25770.24, 36652.24] 0.000
Ward 2013 31237.45 [25793.93, 36680.96] 0.000
Chang 2019 31227.41 [25784.66, 36670.17] 0.000
Dong 2019 31232.98 [25789.64, 36676.32] 0.000
Roth 2015 31232.32 [25788.68, 36675.95] 0.000
Retel 2020 31034.58 [25610.17, 36459.00] 0.000
Lee 2014 31225.21 [25782.18, 36668.24] 0.000
Blohmer 2012 30305.92 [23718.88, 36892.95] 0.000
Ibarrondo 2020 31227.54 [25783.96, 36671.12] 0.000
Jongeneel 2021 31219.56 [25776.69, 36662.42] 0.000
Retel 2020 31063.26 [25635.95, 36490.58] 0.000
Retel 2020 31027.18 [25603.48, 36450.88] 0.000
Holt 2013 31223.18 [25779.59, 36666.77] 0.000
Jung 2021 31221.57 [25776.61, 36666.52] 0.000
You 2015 31135.57 [25700.83, 36570.32] 0.000
Li 2011 31216.58 [25772.61, 36660.54] 0.000
Reed 2013 31209.85 [25761.65, 36658.05] 0.000
Nelson 2013 31042.24 [25616.92, 36467.56] 0.000
Felix 2016 31208.19 [25764.93, 36651.45] 0.000
Lee 2014 31209.08 [25765.33, 36652.82] 0.000
Felix 2016 31198.96 [25755.70, 36642.22] 0.000
Groessl 2018 31075.00 [25645.48, 36504.51] 0.000
Elbasha 2016 31162.44 [25722.19, 36602.69] 0.000
You 2013 31113.60 [25678.96, 36548.23] 0.000
Tanner 2020 31022.93 [25401.81, 36644.05] 0.000
Lee 2014 31163.49 [25721.19, 36605.78] 0.000
You 2012 31112.60 [25677.20, 36548.00] 0.000
Li 2015 31171.19 [25727.45, 36614.93] 0.000
Su 2021 31031.66 [25607.27, 36456.04] 0.000
Wang 2012 31159.54 [25717.34, 36601.75] 0.000
Rubio-Terres 2015 31166.63 [25723.17, 36610.08] 0.000
Segui 2014 31159.65 [25716.13, 36603.17] 0.000
Lobo 2017 31138.77 [25695.21, 36582.32] 0.000
Jahn 2015 31138.04 [25694.50, 36581.58] 0.000
Perez 2011 31114.68 [25671.48, 36557.88] 0.000
Ademi 2014 31055.86 [25548.04, 36563.68] 0.000
Lotan 2018 31100.52 [25660.11, 36540.93] 0.000
Retel 2013 31102.55 [25656.98, 36548.13] 0.000
Segui 2014 31103.81 [25660.29, 36547.34] 0.000
Hornberger 2015 31007.01 [25461.41, 36552.61] 0.000
Hart 2019 31090.61 [25647.27, 36533.94] 0.000
Rodríguez 2019 31050.25 [25603.85, 36496.64] 0.000
Cromwell 2016 30955.42 [25510.55, 36400.28] 0.000
Garrison Jr 2013 30994.96 [25565.59, 36424.34] 0.000
Garrison Jr 2013 31022.58 [25598.47, 36446.68] 0.000
Retèl 2012 30915.92 [25472.41, 36359.42] 0.000
Retèl 2012 30886.98 [25443.65, 36330.31] 0.000
Wong 2021 37260.50 [35405.44, 39115.55] 0.000
Greeley 2011 30735.96 [25292.56, 36179.35] 0.000
Steuten 2019 30463.66 [25020.60, 35906.72] 0.000
Hornberger 2013 30367.84 [24924.97, 35810.70] 0.000
Sun 2019 27689.72 [22250.76, 33128.69] 0.000
Sun 2019 27688.37 [22249.49, 33127.24] 0.000
Sun 2019 27660.23 [22221.15, 33099.31] 0.000
Sun 2019 27659.02 [22219.87, 33098.16] 0.000
Li 2017 27598.52 [22159.57, 33037.47] 0.000
Li 2017 27596.85 [22158.15, 33035.54] 0.000
Nherera 2011 27215.38 [21778.32, 32652.44] 0.000
Bock 2015 27134.03 [21696.69, 32571.36] 0.000
Li 2017 26489.69 [21053.02, 31926.36] 0.000
Li 2017 26487.86 [21051.55, 31924.17] 0.000
20000 25000 30000 35000 40000
Random-effects DerSimonian–Laird model
Sorted by: inmb

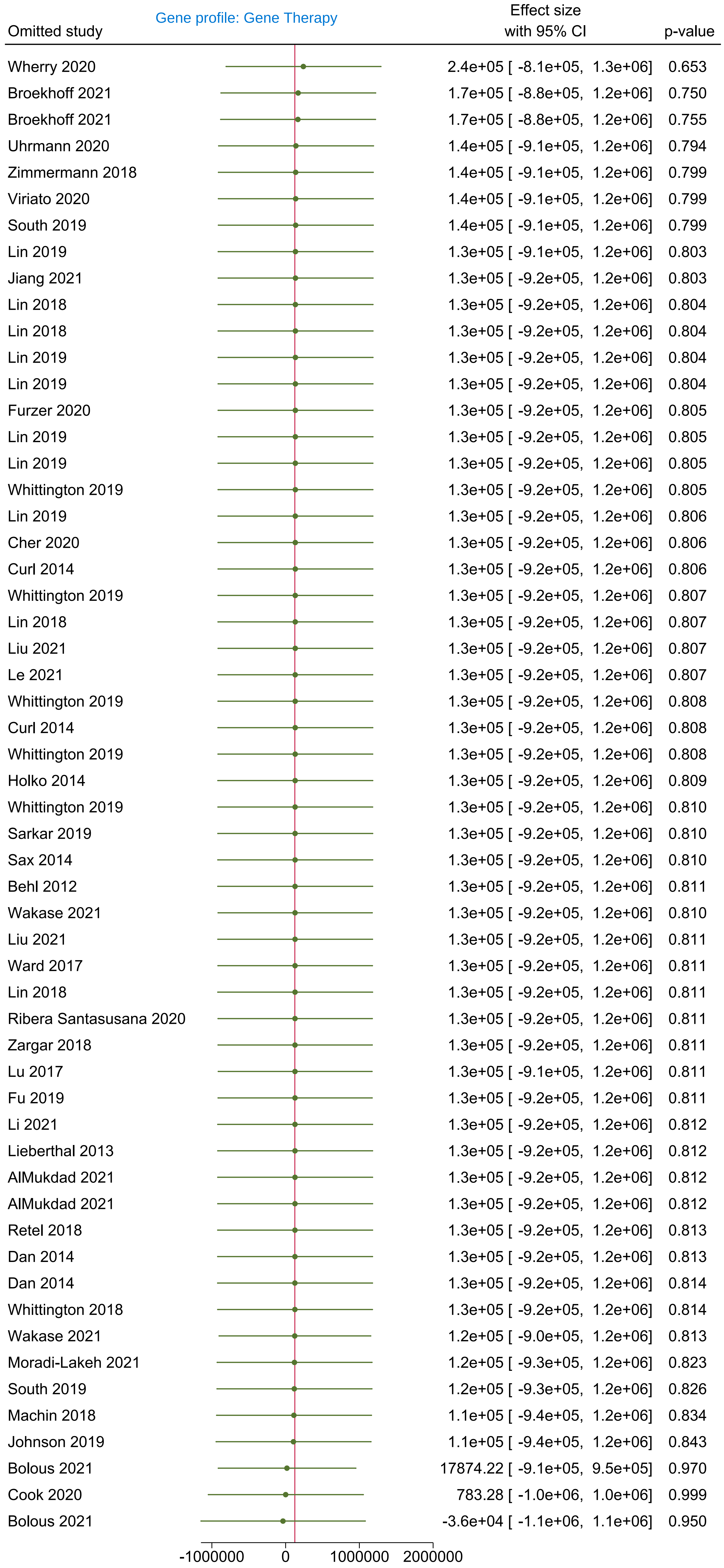

Random-effects DerSimonian–Laird model
Sorted by: inmb

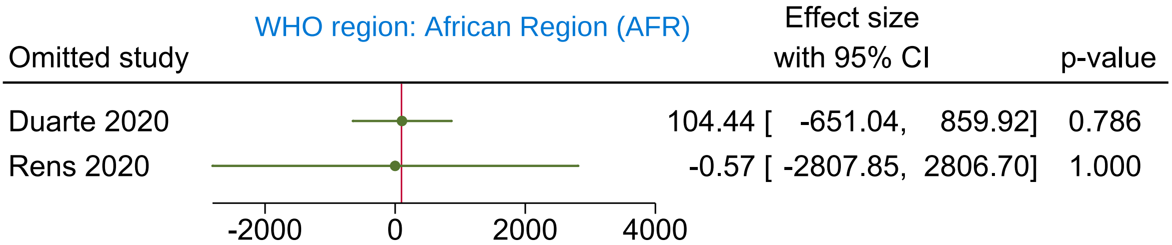

Random-effects DerSimonian–Laird model
Sorted by: inmb

WHO region: Region of the Americas (AMR)

Omitted study	Effect size with 95% CI	p-value
Wherry 2020	1.1e+05 [-49.89, 2.2e+05]	0.050
Asti 2021	81393.51 [-2.7e+04, 1.9e+05]	0.141
Asti 2021	81386.80 [-2.7e+04, 1.9e+05]	0.141
Zimmermann 2018	78964.19 [-2.9e+04, 1.9e+05]	0.152
Lin 2019	79875.51 [-2.8e+04, 1.9e+05]	0.148
Jiang 2021	79815.16 [-2.8e+04, 1.9e+05]	0.149
Lin 2018	79651.13 [-2.9e+04, 1.9e+05]	0.149
Wu 2020	79738.74 [-2.9e+04, 1.9e+05]	0.149
Lin 2018	79733.41 [-2.9e+04, 1.9e+05]	0.149
Lin 2019	79407.39 [-2.9e+04, 1.9e+05]	0.151
Lin 2019	79651.09 [-2.9e+04, 1.9e+05]	0.149
Furzer 2020	79528.46 [-2.9e+04, 1.9e+05]	0.150
Lin 2019	79498.98 [-2.9e+04, 1.9e+05]	0.150
Lin 2019	79484.55 [-2.9e+04, 1.9e+05]	0.150
Whittington 2019	79455.70 [-2.9e+04, 1.9e+05]	0.150
Lin 2019	79358.65 [-2.9e+04, 1.9e+05]	0.151
Curl 2014	79273.39 [-2.9e+04, 1.9e+05]	0.151
Whittington 2019	79182.87 [-2.9e+04, 1.9e+05]	0.152
Lin 2018	79139.00 [-2.9e+04, 1.9e+05]	0.152
Liu 2021	79107.87 [-2.9e+04, 1.9e+05]	0.152
Le 2021	78997.45 [-2.9e+04, 1.9e+05]	0.153
Whittington 2019	78991.16 [-2.9e+04, 1.9e+05]	0.153
Curl 2014	78907.80 [-2.9e+04, 1.9e+05]	0.153
Whittington 2019	78893.31 [-2.9e+04, 1.9e+05]	0.153
Wu 2020	78879.05 [-2.9e+04, 1.9e+05]	0.153
Wu 2020	78798.64 [-3.0e+04, 1.9e+05]	0.154
Wu 2020	78772.50 [-3.0e+04, 1.9e+05]	0.154
Holko 2014	78696.02 [-3.0e+04, 1.9e+05]	0.154
Whittington 2019	78647.34 [-3.0e+04, 1.9e+05]	0.155
Yang 2012	78609.38 [-3.0e+04, 1.9e+05]	0.155
Banerjee 2020	78578.16 [-3.0e+04, 1.9e+05]	0.155
Wu 2020	78571.92 [-3.0e+04, 1.9e+05]	0.155
Wu 2018	78553.85 [-3.0e+04, 1.9e+05]	0.155
Yang 2012	78550.78 [-3.0e+04, 1.9e+05]	0.155
Sarkar 2019	78538.32 [-3.0e+04, 1.9e+05]	0.155
Sax 2014	78513.51 [-3.0e+04, 1.9e+05]	0.155
Wu 2018	78429.38 [-3.0e+04, 1.9e+05]	0.156
Behl 2012	78315.06 [-3.0e+04, 1.9e+05]	0.156
Liu 2021	78373.68 [-3.0e+04, 1.9e+05]	0.156
Ward 2017	78363.27 [-3.0e+04, 1.9e+05]	0.156
Lauren 2020	78346.34 [-3.0e+04, 1.9e+05]	0.156
Behl 2012	78303.91 [-3.0e+04, 1.9e+05]	0.156
Behl 2012	78309.48 [-3.0e+04, 1.9e+05]	0.156
Lin 2018	78335.44 [-3.0e+04, 1.9e+05]	0.156
Liu 2012	78302.01 [-3.0e+04, 1.9e+05]	0.156
You 2012	78308.38 [-3.0e+04, 1.9e+05]	0.156
Wang 2012	78303.77 [-3.0e+04, 1.9e+05]	0.156
Aguiar 2017	78300.05 [-3.0e+04, 1.9e+05]	0.156
Wallbillich 2016	78278.72 [-3.0e+04, 1.9e+05]	0.157
Zargar 2018	78264.67 [-3.0e+04, 1.9e+05]	0.157
Lee 2014	78262.05 [-3.0e+04, 1.9e+05]	0.157
Handorf 2012	78260.84 [-3.0e+04, 1.9e+05]	0.157
Zhu 2021	78258.34 [-3.0e+04, 1.9e+05]	0.157
Wang 2012	78254.55 [-3.0e+04, 1.9e+05]	0.157
Wang 2012	78253.45 [-3.0e+04, 1.9e+05]	0.157
Criss 2019	78249.46 [-3.0e+04, 1.9e+05]	0.157
Nelson 2013	78225.13 [-3.0e+04, 1.9e+05]	0.157
Balentine 2018	78244.43 [-3.0e+04, 1.9e+05]	0.157
Lee 2014	78244.95 [-3.0e+04, 1.9e+05]	0.157
Chen 2015	78245.26 [-3.0e+04, 1.9e+05]	0.157
Handorf 2012	78244.46 [-3.0e+04, 1.9e+05]	0.157
Wang 2012	78244.01 [-3.0e+04, 1.9e+05]	0.157
Perez 2011	78241.91 [-3.0e+04, 1.9e+05]	0.157
Alkhatib 2020	78239.68 [-3.0e+04, 1.9e+05]	0.157
Liu 2012	78234.77 [-3.0e+04, 1.9e+05]	0.157
Crespin 2011	78237.38 [-3.0e+04, 1.9e+05]	0.157
Romanus 2015	78214.09 [-3.0e+04, 1.9e+05]	0.157
Su 2021	78236.18 [-3.0e+04, 1.9e+05]	0.157
Wang 2012	78235.63 [-3.0e+04, 1.9e+05]	0.157
Okere 2018	78234.91 [-3.0e+04, 1.9e+05]	0.157
Wang 2012	78234.65 [-3.0e+04, 1.9e+05]	0.157
Lobo 2017	78232.22 [-3.0e+04, 1.9e+05]	0.157
Limdi 2020	78230.07 [-3.0e+04, 1.9e+05]	0.157
Naylor 2014	78230.38 [-3.0e+04, 1.9e+05]	0.157
Ladabaum 2013	78229.33 [-3.0e+04, 1.9e+05]	0.157
Paulden 2013	78228.77 [-3.0e+04, 1.9e+05]	0.157
Djalalov 2014	78225.37 [-3.0e+04, 1.9e+05]	0.157
Hannouf 2019	78224.26 [-3.0e+04, 1.9e+05]	0.157
Wu 2016	78166.59 [-3.0e+04, 1.9e+05]	0.157
Lotan 2018	78219.06 [-3.0e+04, 1.9e+05]	0.157
Parthan 2013	78222.84 [-3.0e+04, 1.9e+05]	0.157
Hyle 2020	78220.27 [-3.0e+04, 1.9e+05]	0.157
Kim 2019	78221.58 [-3.0e+04, 1.9e+05]	0.157
Biltaji 2021	78220.13 [-3.0e+04, 1.9e+05]	0.157
Chandler 2018	78221.12 [-3.0e+04, 1.9e+05]	0.157
Martes-Martinez 2017	78220.05 [-3.0e+04, 1.9e+05]	0.157
Guzauskas 2020	78219.45 [-3.0e+04, 1.9e+05]	0.157
Romanus 2015	78199.04 [-3.0e+04, 1.9e+05]	0.157
Alagoz 2016	78220.53 [-3.0e+04, 1.9e+05]	0.157
Guzauskas 2020	78220.30 [-3.0e+04, 1.9e+05]	0.157
Hao 2019	78215.81 [-3.0e+04, 1.9e+05]	0.157
Biltaji 2021	78217.91 [-3.0e+04, 1.9e+05]	0.157
Kansal 2013	78219.92 [-3.0e+04, 1.9e+05]	0.157
Nshimyumukiza 2013	78219.63 [-3.0e+04, 1.9e+05]	0.157
Phelps 2014	78219.51 [-3.0e+04, 1.9e+05]	0.157
Feller-Kopman 2017	78219.33 [-3.0e+04, 1.9e+05]	0.157
Biltaji 2021	78219.13 [-3.0e+04, 1.9e+05]	0.157
Forde 2016	78219.21 [-3.0e+04, 1.9e+05]	0.157
Lee 2014	78218.58 [-3.0e+04, 1.9e+05]	0.157
Lieberthal 2013	78217.50 [-3.0e+04, 1.9e+05]	0.157
Biltaji 2021	78218.44 [-3.0e+04, 1.9e+05]	0.157
Djalalov 2012	78218.93 [-3.0e+04, 1.9e+05]	0.157
Rens 2020	78218.64 [-3.0e+04, 1.9e+05]	0.157
Kazi 2014	78212.03 [-3.0e+04, 1.9e+05]	0.157
Hannouf 2019	78218.05 [-3.0e+04, 1.9e+05]	0.157
Lotan 2018	78216.61 [-3.0e+04, 1.9e+05]	0.157
Nelson 2013	78207.25 [-3.0e+04, 1.9e+05]	0.157
Bargallo-Rocha 2015	78217.23 [-3.0e+04, 1.9e+05]	0.157
GoodSmith 2019	78211.63 [-3.0e+04, 1.9e+05]	0.158
Li 2015	78217.31 [-3.0e+04, 1.9e+05]	0.157
Correa-Galendi 2021	76303.59 [-1.7e+05, 3.2e+05]	0.548
Sathianathen 2018	78216.31 [-3.0e+04, 1.9e+05]	0.157
Huh 2015	78216.09 [-3.0e+04, 1.9e+05]	0.157
Forde 2016	78215.98 [-3.0e+04, 1.9e+05]	0.157
Kansal 2013	78215.83 [-3.0e+04, 1.9e+05]	0.157
Okere 2018	78215.49 [-3.0e+04, 1.9e+05]	0.157
Kazi 2014	78215.46 [-3.0e+04, 1.9e+05]	0.157
Patel 2014	78215.63 [-3.0e+04, 1.9e+05]	0.157
Sathianathen 2018	78215.55 [-3.0e+04, 1.9e+05]	0.157
Wu 2016	78212.95 [-3.0e+04, 1.9e+05]	0.157
Sathianathen 2018	78213.66 [-3.0e+04, 1.9e+05]	0.157
Sathianathen 2018	78213.65 [-3.0e+04, 1.9e+05]	0.157
Lee 2014	78213.11 [-3.0e+04, 1.9e+05]	0.157
Lee 2014	78212.38 [-3.0e+04, 1.9e+05]	0.157
Hannouf 2019	78212.65 [-3.0e+04, 1.9e+05]	0.157
Ontario Health (Quality) 2020	78212.05 [-3.0e+04, 1.9e+05]	0.157
Ladabaum 2011	78211.05 [-3.0e+04, 1.9e+05]	0.157
Hannouf 2016	78211.00 [-3.0e+04, 1.9e+05]	0.157
Jiang 2016	78210.38 [-3.0e+04, 1.9e+05]	0.157
Lieberthal 2013	78208.23 [-3.0e+04, 1.9e+05]	0.157
Torchia 2019	78209.34 [-3.0e+04, 1.9e+05]	0.157
Roth 2014	78209.09 [-3.0e+04, 1.9e+05]	0.157
Dong 2019	78208.03 [-3.0e+04, 1.9e+05]	0.157
Chang 2019	78207.48 [-3.0e+04, 1.9e+05]	0.157
Dong 2019	78207.21 [-3.0e+04, 1.9e+05]	0.157
Ontario Health (Quality) 2020	78206.75 [-3.0e+04, 1.9e+05]	0.157
Roth 2015	78205.49 [-3.0e+04, 1.9e+05]	0.157
Lee 2014	78204.09 [-3.0e+04, 1.9e+05]	0.157
Kim 2019	78203.68 [-3.0e+04, 1.9e+05]	0.157
Cressman 2016	78203.65 [-3.0e+04, 1.9e+05]	0.157
Kim 2019	78200.02 [-3.0e+04, 1.9e+05]	0.157
Liu 2012	78199.16 [-3.0e+04, 1.9e+05]	0.157
Kim 2019	78200.98 [-3.0e+04, 1.9e+05]	0.157
Retel 2020	78140.45 [-3.0e+04, 1.9e+05]	0.157
Hoskins 2019	78199.15 [-3.0e+04, 1.9e+05]	0.157
Jung 2021	78198.91 [-3.0e+04, 1.9e+05]	0.157
You 2015	78198.13 [-3.0e+04, 1.9e+05]	0.157
Jiang 2016	77958.28 [-3.9e+04, 2.0e+05]	0.192
Jiang 2016	76388.68 [-1.6e+05, 3.1e+05]	0.519
Li 2011	78195.23 [-3.0e+04, 1.9e+05]	0.157
Ontario Health (Quality) 2020	78193.63 [-3.0e+04, 1.9e+05]	0.157
Reed 2013	78192.73 [-3.0e+04, 1.9e+05]	0.157
Nelson 2013	78187.13 [-3.0e+04, 1.9e+05]	0.157
Jiang 2017	78191.66 [-3.0e+04, 1.9e+05]	0.157
Felix 2016	78191.30 [-3.0e+04, 1.9e+05]	0.157
Lee 2014	78190.29 [-3.0e+04, 1.9e+05]	0.157
Felix 2016	78185.18 [-3.0e+04, 1.9e+05]	0.157
Ontario Health (Quality) 2020	78184.00 [-3.0e+04, 1.9e+05]	0.157
Kwon 2018	78178.70 [-3.0e+04, 1.9e+05]	0.157
Liu 2012	78178.09 [-3.0e+04, 1.9e+05]	0.157
Groessl 2018	78177.20 [-3.0e+04, 1.9e+05]	0.157
Elbasha 2016	78177.21 [-3.0e+04, 1.9e+05]	0.157
You 2013	78173.53 [-3.0e+04, 1.9e+05]	0.157
Tanner 2020	78149.39 [-3.1e+04, 1.9e+05]	0.160
Lee 2014	78166.33 [-3.0e+04, 1.9e+05]	0.157
You 2012	78165.84 [-3.0e+04, 1.9e+05]	0.157
Li 2015	78165.55 [-3.0e+04, 1.9e+05]	0.157
Su 2021	78153.10 [-3.0e+04, 1.9e+05]	0.157
Tremblay 2019	78155.95 [-3.0e+04, 1.9e+05]	0.157
Alkhatib 2018	78145.30 [-3.0e+04, 1.9e+05]	0.157
Lobo 2017	78144.45 [-3.0e+04, 1.9e+05]	0.157
Hart 2019	78130.57 [-3.0e+04, 1.9e+05]	0.157
Perez 2011	78129.53 [-3.0e+04, 1.9e+05]	0.157
Lotan 2018	78128.27 [-3.0e+04, 1.9e+05]	0.157
Schackman 2015	78118.73 [-3.0e+04, 1.9e+05]	0.157
Alkhatib 2018	78121.45 [-3.0e+04, 1.9e+05]	0.157
Hornberger 2015	78105.51 [-3.1e+04, 1.9e+05]	0.159
Hart 2019	78113.27 [-3.0e+04, 1.9e+05]	0.157
Kwon 2018	78052.38 [-3.0e+04, 1.9e+05]	0.158
Cromwell 2016	78025.06 [-3.0e+04, 1.9e+05]	0.158
Crawford 2021	77954.75 [-3.0e+04, 1.9e+05]	0.158
Choi 2019	78007.70 [-3.0e+04, 1.9e+05]	0.158
Garrison Jr 2013	78001.13 [-3.0e+04, 1.9e+05]	0.158
Garrison Jr 2013	77993.05 [-3.0e+04, 1.9e+05]	0.158
Lipton 2020	77994.66 [-3.0e+04, 1.9e+05]	0.158
Crawford 2021	77928.48 [-3.0e+04, 1.9e+05]	0.158
Das 2016	77975.37 [-3.0e+04, 1.9e+05]	0.158
Das 2016	77974.70 [-3.0e+04, 1.9e+05]	0.158
Lipton 2020	77969.54 [-3.0e+04, 1.9e+05]	0.158
Wu 2020	77916.27 [-3.0e+04, 1.9e+05]	0.159
Chen 2018	77880.95 [-3.0e+04, 1.9e+05]	0.159
Greeley 2011	77880.16 [-3.0e+04, 1.9e+05]	0.159
Das 2016	77855.27 [-3.0e+04, 1.9e+05]	0.159
Whittington 2018	77816.34 [-3.0e+04, 1.9e+05]	0.159
Steuten 2019	77702.62 [-3.1e+04, 1.9e+05]	0.160
Steuten 2019	77675.13 [-3.1e+04, 1.9e+05]	0.160
Hornberger 2013	77639.88 [-3.1e+04, 1.9e+05]	0.160
Sun 2019	75876.87 [-3.2e+04, 1.8e+05]	0.170
Sun 2019	75876.20 [-3.2e+04, 1.8e+05]	0.170
Sun 2019	75856.90 [-3.2e+04, 1.8e+05]	0.170
Sun 2019	75855.91 [-3.2e+04, 1.8e+05]	0.170
Li 2017	75816.20 [-3.2e+04, 1.8e+05]	0.170
Li 2017	75815.81 [-3.2e+04, 1.8e+05]	0.170
Bock 2015	75511.20 [-3.3e+04, 1.8e+05]	0.172
Li 2017	75083.49 [-3.3e+04, 1.8e+05]	0.174
Li 2017	75083.30 [-3.3e+04, 1.8e+05]	0.174
Machin 2018	74226.46 [-3.4e+04, 1.8e+05]	0.179
Johnson 2019	72576.84 [-3.6e+04, 1.8e+05]	0.189
Bolous 2021	65013.80 [54930.99, 75096.61]	0.000
Cook 2020	44731.91 [-6.4e+04, 1.5e+05]	0.418
Bolous 2021	35080.13 [-7.3e+04, 1.4e+05]	0.524

-200000 0 200000 400000

Random-effects DerSimonian–Laird model
Sorted by: inmb

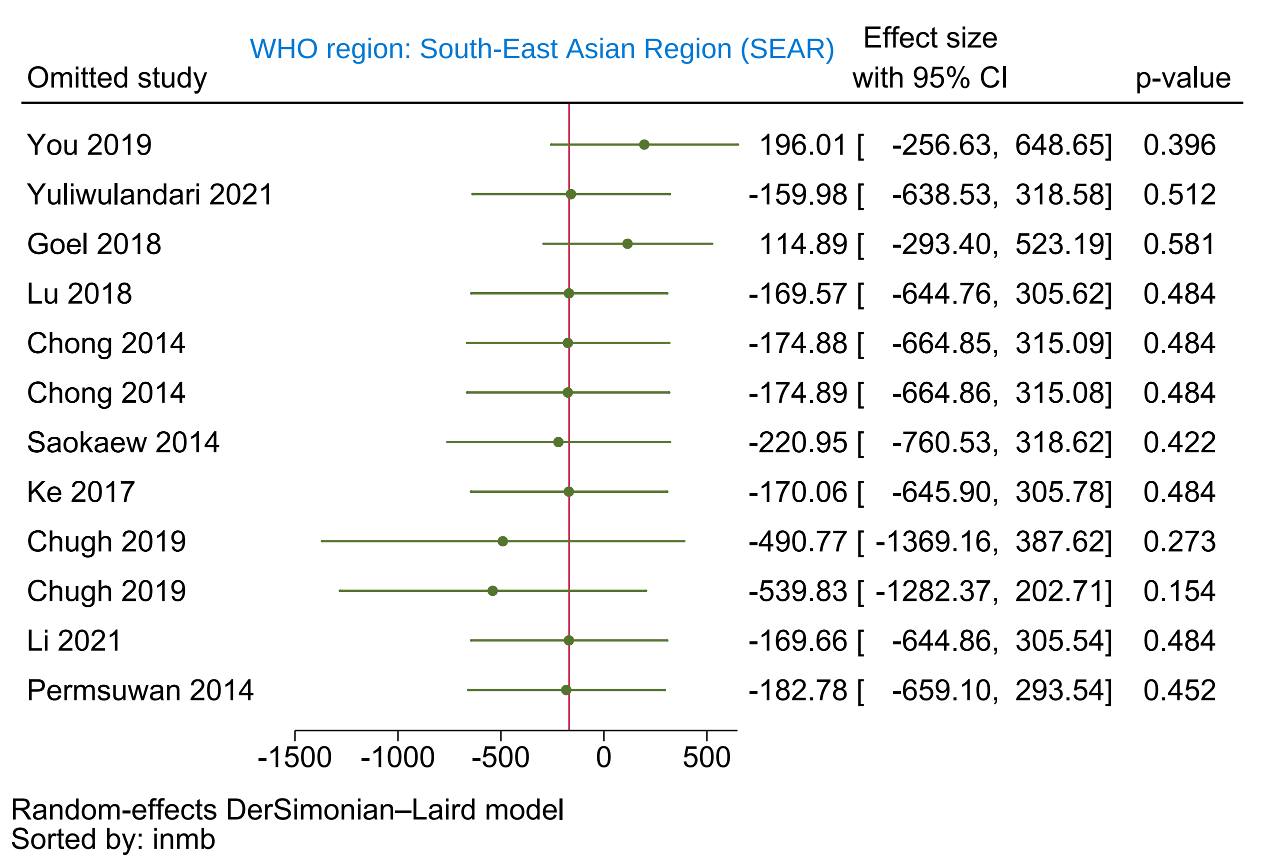
WHO region: South-East Asian Region (SEAR)
Omitted study
Effect size
with 95% CI
p-value
You 2019
196.01 [-256.63, 648.65]
0.396
Yuliwulandari 2021
-159.98 [-638.53, 318.58]
0.512
Goel 2018
114.89 [-293.40, 523.19]
0.581
Lu 2018
-169.57 [-644.76, 305.62]
0.484
Chong 2014
-174.88 [-664.85, 315.09]
0.484
Chong 2014
-174.89 [-664.86, 315.08]
0.484
Saokaew 2014
-220.95 [-760.53, 318.62]
0.422
Ke 2017
-170.06 [-645.90, 305.78]
0.484
Chugh 2019
-490.77 [-1369.16, 387.62]
0.273
Chugh 2019
-539.83 [-1282.37, 202.71]
0.154
Li 2021
-169.66 [-644.86, 305.54]
0.484
Permsuwan 2014
-182.78 [-659.10, 293.54]
0.452
-1500
-1000
-500
0
500
Random-effects DerSimonian–Laird model
Sorted by: inmb

WHO region: European Region (EUR)

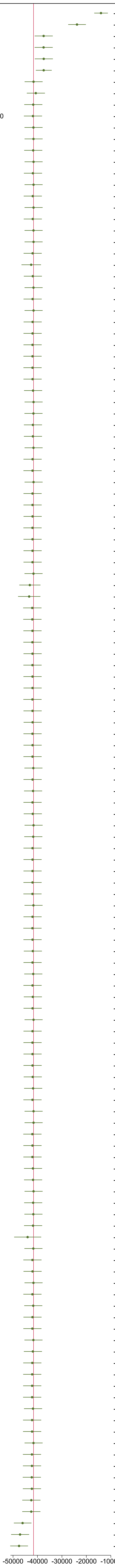

Omitted study	Effect size with 95% CI	p-value
Broekhoff 2021	-1.4e+04 [-1.7e+04, -1.1e+04]	0.000
Broekhoff 2021	-2.4e+04 [-2.7e+04, -2.0e+04]	0.000
Uhrmann 2020	-3.7e+04 [-4.1e+04, -3.4e+04]	0.000
Viriato 2020	-3.7e+04 [-4.1e+04, -3.4e+04]	0.000
South 2019	-3.7e+04 [-4.1e+04, -3.4e+04]	0.000
Blank 2011	-3.7e+04 [-4.1e+04, -3.4e+04]	0.000
Nicholson 2015	-4.2e+04 [-4.5e+04, -3.8e+04]	0.000
Berm 2016	-4.1e+04 [-4.4e+04, -3.7e+04]	0.000
Marguet 2016	-4.2e+04 [-4.5e+04, -3.8e+04]	0.000
Ribera Santasusana 2020	-4.2e+04 [-4.5e+04, -3.8e+04]	0.000
Buchanan 2017	-4.2e+04 [-4.5e+04, -3.8e+04]	0.000
Harty 2018	-4.2e+04 [-4.5e+04, -3.8e+04]	0.000
Marguet 2016	-4.2e+04 [-4.5e+04, -3.8e+04]	0.000
Butzke 2015	-4.2e+04 [-4.5e+04, -3.8e+04]	0.000
Marguet 2016	-4.2e+04 [-4.5e+04, -3.8e+04]	0.000
Harty 2018	-4.2e+04 [-4.5e+04, -3.8e+04]	0.000
Jahn 2017	-4.2e+04 [-4.6e+04, -3.8e+04]	0.000
Verhoef 2019	-4.2e+04 [-4.5e+04, -3.8e+04]	0.000
Sun 2019	-4.2e+04 [-4.6e+04, -3.8e+04]	0.000
Harty 2018	-4.2e+04 [-4.5e+04, -3.8e+04]	0.000
Sun 2019	-4.2e+04 [-4.5e+04, -3.8e+04]	0.000
Huxley 2015	-4.2e+04 [-4.6e+04, -3.8e+04]	0.000
Vriens 2014	-4.3e+04 [-4.6e+04, -3.9e+04]	0.000
Govers 2013	-4.2e+04 [-4.5e+04, -3.8e+04]	0.000
Govers 2013	-4.2e+04 [-4.5e+04, -3.8e+04]	0.000
Ladabaum 2014	-4.2e+04 [-4.6e+04, -3.8e+04]	0.000
Pink 2014	-4.2e+04 [-4.5e+04, -3.8e+04]	0.000
Bonastre 2014	-4.2e+04 [-4.6e+04, -3.8e+04]	0.000
Vriens 2014	-4.2e+04 [-4.6e+04, -3.8e+04]	0.000
Moya-Alarcón 2019	-4.2e+04 [-4.6e+04, -3.8e+04]	0.000
Özmen 2019	-4.2e+04 [-4.6e+04, -3.8e+04]	0.000
Lázaro 2017	-4.2e+04 [-4.6e+04, -3.8e+04]	0.000
Huxley 2015	-4.2e+04 [-4.6e+04, -3.8e+04]	0.000
Luime 2015	-4.2e+04 [-4.5e+04, -3.8e+04]	0.000
McKay 2018	-4.2e+04 [-4.5e+04, -3.8e+04]	0.000
McKay 2018	-4.2e+04 [-4.5e+04, -3.8e+04]	0.000
Mitropoulou 2014	-4.2e+04 [-4.5e+04, -3.8e+04]	0.000
McKay 2018	-4.2e+04 [-4.5e+04, -3.8e+04]	0.000
McKay 2018	-4.2e+04 [-4.5e+04, -3.8e+04]	0.000
Gray 2017	-4.2e+04 [-4.6e+04, -3.8e+04]	0.000
Thompson 2013	-4.2e+04 [-4.6e+04, -3.8e+04]	0.000
Schackman 2013	-4.2e+04 [-4.5e+04, -3.8e+04]	0.000
Compagni 2013	-4.2e+04 [-4.6e+04, -3.8e+04]	0.000
Sluiter 2019	-4.2e+04 [-4.6e+04, -3.8e+04]	0.000
Severin 2015	-4.2e+04 [-4.6e+04, -3.8e+04]	0.000
Plumpton 2017	-4.2e+04 [-4.6e+04, -3.8e+04]	0.000
Walter 2017	-4.2e+04 [-4.6e+04, -3.8e+04]	0.000
Saramago 2018	-4.2e+04 [-4.6e+04, -3.8e+04]	0.000
Saramago 2018	-4.2e+04 [-4.6e+04, -3.8e+04]	0.000
Mugwagwa 2021	-4.2e+04 [-4.5e+04, -3.8e+04]	0.000
Snowsill 2020	-4.3e+04 [-4.7e+04, -3.9e+04]	0.000
Snowsill 2020	-4.3e+04 [-4.8e+04, -3.9e+04]	0.000
Snowsill 2020	-4.2e+04 [-4.6e+04, -3.8e+04]	0.000
Snowsill 2020	-4.2e+04 [-4.6e+04, -3.8e+04]	0.000
Mugwagwa 2021	-4.2e+04 [-4.6e+04, -3.8e+04]	0.000
Mugwagwa 2021	-4.2e+04 [-4.6e+04, -3.8e+04]	0.000
Saramago 2018	-4.2e+04 [-4.6e+04, -3.8e+04]	0.000
Mugwagwa 2021	-4.2e+04 [-4.6e+04, -3.8e+04]	0.000
Sutherland 2019	-4.2e+04 [-4.6e+04, -3.8e+04]	0.000
Saramago 2018	-4.2e+04 [-4.6e+04, -3.8e+04]	0.000
Mugwagwa 2021	-4.2e+04 [-4.6e+04, -3.8e+04]	0.000
Saramago 2018	-4.2e+04 [-4.6e+04, -3.8e+04]	0.000
Pedersen 2016	-4.2e+04 [-4.6e+04, -3.8e+04]	0.000
Verhoef 2014	-4.2e+04 [-4.6e+04, -3.8e+04]	0.000
McKay 2018	-4.2e+04 [-4.6e+04, -3.8e+04]	0.000
Dymond 2020	-4.2e+04 [-4.6e+04, -3.8e+04]	0.000
McKay 2018	-4.2e+04 [-4.5e+04, -3.8e+04]	0.000
Verhoef 2014	-4.2e+04 [-4.6e+04, -3.8e+04]	0.000
Bonastre 2014	-4.2e+04 [-4.5e+04, -3.8e+04]	0.000
Verhoef 2016	-4.2e+04 [-4.6e+04, -3.8e+04]	0.000
Gray 2017	-4.2e+04 [-4.6e+04, -3.8e+04]	0.000
Pink 2014	-4.2e+04 [-4.5e+04, -3.8e+04]	0.000
McKay 2018	-4.2e+04 [-4.5e+04, -3.8e+04]	0.000
Ward 2013	-4.2e+04 [-4.6e+04, -3.8e+04]	0.000
Gray 2017	-4.2e+04 [-4.6e+04, -3.8e+04]	0.000
Gray 2017	-4.2e+04 [-4.6e+04, -3.8e+04]	0.000
Verhoef 2016	-4.2e+04 [-4.6e+04, -3.8e+04]	0.000
Sluiter 2018	-4.2e+04 [-4.6e+04, -3.8e+04]	0.000
Retel 2020	-4.2e+04 [-4.5e+04, -3.8e+04]	0.000
Walter 2017	-4.2e+04 [-4.6e+04, -3.8e+04]	0.000
Verhoef 2013	-4.2e+04 [-4.6e+04, -3.8e+04]	0.000
Walter 2017	-4.2e+04 [-4.6e+04, -3.8e+04]	0.000
Marguet 2016	-4.2e+04 [-4.5e+04, -3.8e+04]	0.000
Gray 2017	-4.2e+04 [-4.6e+04, -3.8e+04]	0.000
Schremser 2015	-4.2e+04 [-4.5e+04, -3.8e+04]	0.000
Gray 2017	-4.2e+04 [-4.6e+04, -3.8e+04]	0.000
Sun 2019	-4.2e+04 [-4.5e+04, -3.8e+04]	0.000
Plumpton 2015	-4.2e+04 [-4.6e+04, -3.8e+04]	0.000
Butzke 2015	-4.2e+04 [-4.5e+04, -3.8e+04]	0.000
Luime 2015	-4.2e+04 [-4.6e+04, -3.8e+04]	0.000
Ibarrondo 2020	-4.2e+04 [-4.6e+04, -3.8e+04]	0.000
Sun 2019	-4.2e+04 [-4.6e+04, -3.8e+04]	0.000
Jongeneel 2021	-4.2e+04 [-4.6e+04, -3.8e+04]	0.000
Marguet 2016	-4.2e+04 [-4.6e+04, -3.8e+04]	0.000
Ghatnekar 2013	-4.2e+04 [-4.5e+04, -3.8e+04]	0.000
Pashayan 2018	-4.2e+04 [-4.6e+04, -3.8e+04]	0.000
Retel 2020	-4.2e+04 [-4.5e+04, -3.8e+04]	0.000
Retel 2020	-4.2e+04 [-4.5e+04, -3.8e+04]	0.000
Eccleston 2017	-4.2e+04 [-4.6e+04, -3.8e+04]	0.000
Ward 2013	-4.2e+04 [-4.6e+04, -3.8e+04]	0.000
Rejon-Parrilla 2014	-4.2e+04 [-4.6e+04, -3.8e+04]	0.000
Woods 2011	-4.2e+04 [-4.5e+04, -3.8e+04]	0.000
Marguet 2016	-4.2e+04 [-4.5e+04, -3.8e+04]	0.000
Retel 2020	-4.2e+04 [-4.5e+04, -3.8e+04]	0.000
Marguet 2016	-4.2e+04 [-4.5e+04, -3.8e+04]	0.000
Woods 2011	-4.2e+04 [-4.5e+04, -3.8e+04]	0.000
Marguet 2016	-4.2e+04 [-4.5e+04, -3.8e+04]	0.000
Blohmer 2012	-4.4e+04 [-4.9e+04, -3.9e+04]	0.000
Blank 2015	-4.2e+04 [-4.5e+04, -3.8e+04]	0.000
Ibarrondo 2020	-4.2e+04 [-4.6e+04, -3.8e+04]	0.000
Jongeneel 2021	-4.2e+04 [-4.6e+04, -3.8e+04]	0.000
Retel 2020	-4.2e+04 [-4.5e+04, -3.8e+04]	0.000
Holt 2013	-4.2e+04 [-4.6e+04, -3.8e+04]	0.000
Marguet 2016	-4.2e+04 [-4.5e+04, -3.8e+04]	0.000
Hall 2012	-4.2e+04 [-4.6e+04, -3.8e+04]	0.000
Katz 2015	-4.2e+04 [-4.6e+04, -3.8e+04]	0.000
Buchanan 2017	-4.2e+04 [-4.5e+04, -3.8e+04]	0.000
Müller 2019	-4.2e+04 [-4.5e+04, -3.8e+04]	0.000
Katz 2015	-4.2e+04 [-4.6e+04, -3.9e+04]	0.000
Rubio-Terres 2015	-4.2e+04 [-4.6e+04, -3.9e+04]	0.000
Segui 2014	-4.2e+04 [-4.6e+04, -3.9e+04]	0.000
Marguet 2016	-4.2e+04 [-4.6e+04, -3.9e+04]	0.000
Fawsitt 2020	-4.2e+04 [-4.5e+04, -3.8e+04]	0.000
Jahn 2015	-4.2e+04 [-4.6e+04, -3.9e+04]	0.000
Marguet 2016	-4.2e+04 [-4.6e+04, -3.9e+04]	0.000
Retel 2018	-4.2e+04 [-4.5e+04, -3.8e+04]	0.000
Retel 2013	-4.2e+04 [-4.6e+04, -3.9e+04]	0.000
Segui 2014	-4.2e+04 [-4.6e+04, -3.9e+04]	0.000
Rodríguez 2019	-4.2e+04 [-4.6e+04, -3.9e+04]	0.000
Marguet 2016	-4.2e+04 [-4.6e+04, -3.9e+04]	0.000
Retèl 2012	-4.3e+04 [-4.6e+04, -3.9e+04]	0.000
Retèl 2012	-4.3e+04 [-4.6e+04, -3.9e+04]	0.000
Moradi-Lakeh 2021	-4.6e+04 [-5.0e+04, -4.3e+04]	0.000
South 2019	-4.7e+04 [-5.1e+04, -4.4e+04]	0.000
Nherera 2011	-4.8e+04 [-5.1e+04, -4.4e+04]	0.000

Random-effects DerSimonian–Laird model
Sorted by: inmb

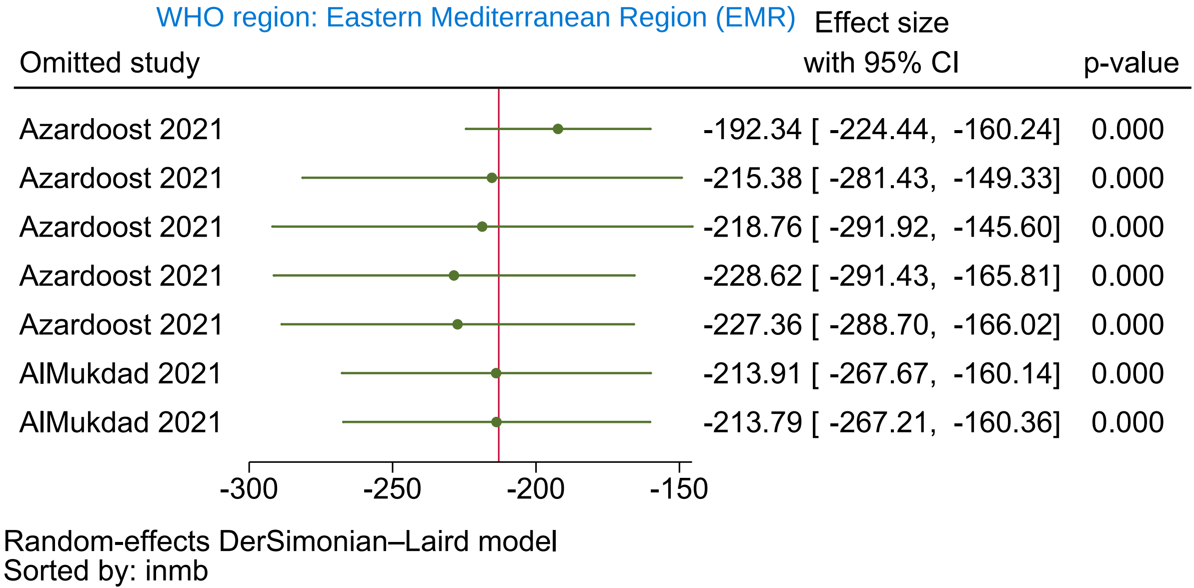
WHO region: Eastern Mediterranean Region (EMR)
Omitted study
Effect size with 95% CI
p-value
Azardoost 2021 -192.34 [-224.44, -160.24] 0.000
Azardoost 2021 -215.38 [-281.43, -149.33] 0.000
Azardoost 2021 -218.76 [-291.92, -145.60] 0.000
Azardoost 2021 -228.62 [-291.43, -165.81] 0.000
Azardoost 2021 -227.36 [-288.70, -166.02] 0.000
AlMukdad 2021 -213.91 [-267.67, -160.14] 0.000
AlMukdad 2021 -213.79 [-267.21, -160.36] 0.000
-300
-250
-200
-150
Random-effects DerSimonian–Laird model
Sorted by: inmb

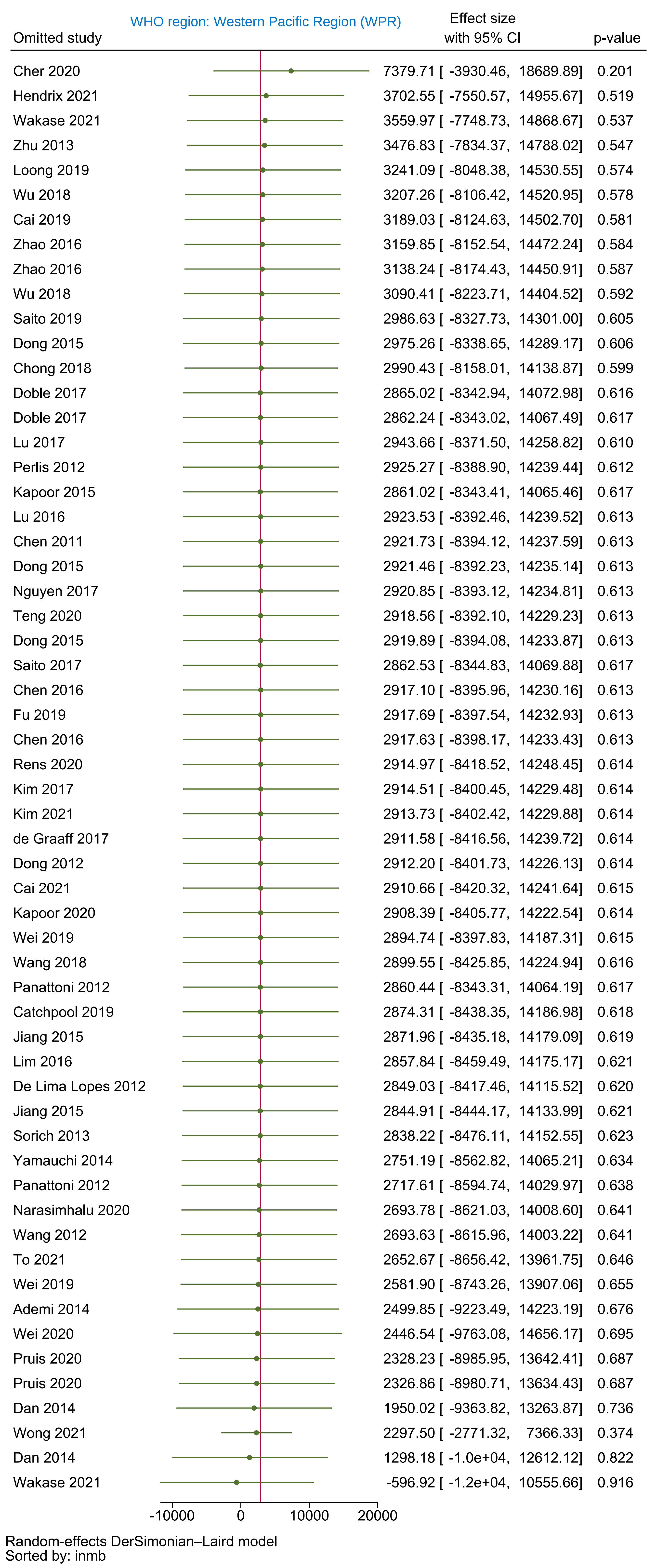

WHO region: Western Pacific Region (WPR)
Omitted study
Effect size with 95% CI
p-value
Cher 2020 7379.71 [-3930.46, 18689.89] 0.201
Hendrix 2021 3702.55 [-7550.57, 14955.67] 0.519
Wakase 2021 3559.97 [-7748.73, 14868.67] 0.537
Zhu 2013 3476.83 [-7834.37, 14788.02] 0.547
Loong 2019 3241.09 [-8048.38, 14530.55] 0.574
Wu 2018 3207.26 [-8106.42, 14520.95] 0.578
Cai 2019 3189.03 [-8124.63, 14502.70] 0.581
Zhao 2016 3159.85 [-8152.54, 14472.24] 0.584
Zhao 2016 3138.24 [-8174.43, 14450.91] 0.587
Wu 2018 3090.41 [-8223.71, 14404.52] 0.592
Saito 2019 2986.63 [-8327.73, 14301.00] 0.605
Dong 2015 2975.26 [-8338.65, 14289.17] 0.606
Chong 2018 2990.43 [-8158.01, 14138.87] 0.599
Doble 2017 2865.02 [-8342.94, 14072.98] 0.616
Doble 2017 2862.24 [-8343.02, 14067.49] 0.617
Lu 2017 2943.66 [-8371.50, 14258.82] 0.610
Perlis 2012 2925.27 [-8388.90, 14239.44] 0.612
Kapoor 2015 2861.02 [-8343.41, 14065.46] 0.617
Lu 2016 2923.53 [-8392.46, 14239.52] 0.613
Chen 2011 2921.73 [-8394.12, 14237.59] 0.613
Dong 2015 2921.46 [-8392.23, 14235.14] 0.613
Nguyen 2017 2920.85 [-8393.12, 14234.81] 0.613
Teng 2020 2918.56 [-8392.10, 14229.23] 0.613
Dong 2015 2919.89 [-8394.08, 14233.87] 0.613
Saito 2017 2862.53 [-8344.83, 14069.88] 0.617
Chen 2016 2917.10 [-8395.96, 14230.16] 0.613
Fu 2019 2917.69 [-8397.54, 14232.93] 0.613
Chen 2016 2917.63 [-8398.17, 14233.43] 0.613
Rens 2020 2914.97 [-8418.52, 14248.45] 0.614
Kim 2017 2914.51 [-8400.45, 14229.48] 0.614
Kim 2021 2913.73 [-8402.42, 14229.88] 0.614
de Graaff 2017 2911.58 [-8416.56, 14239.72] 0.614
Dong 2012 2912.20 [-8401.73, 14226.13] 0.614
Cai 2021 2910.66 [-8420.32, 14241.64] 0.615
Kapoor 2020 2908.39 [-8405.77, 14222.54] 0.614
Wei 2019 2894.74 [-8397.83, 14187.31] 0.615
Wang 2018 2899.55 [-8425.85, 14224.94] 0.616
Panattoni 2012 2860.44 [-8343.31, 14064.19] 0.617
Catchpool 2019 2874.31 [-8438.35, 14186.98] 0.618
Jiang 2015 2871.96 [-8435.18, 14179.09] 0.619
Lim 2016 2857.84 [-8459.49, 14175.17] 0.621
De Lima Lopes 2012 2849.03 [-8417.46, 14115.52] 0.620
Jiang 2015 2844.91 [-8444.17, 14133.99] 0.621
Sorich 2013 2838.22 [-8476.11, 14152.55] 0.623
Yamauchi 2014 2751.19 [-8562.82, 14065.21] 0.634
Panattoni 2012 2717.61 [-8594.74, 14029.97] 0.638
Narasimhalu 2020 2693.78 [-8621.03, 14008.60] 0.641
Wang 2012 2693.63 [-8615.96, 14003.22] 0.641
To 2021 2652.67 [-8656.42, 13961.75] 0.646
Wei 2019 2581.90 [-8743.26, 13907.06] 0.655
Ademi 2014 2499.85 [-9223.49, 14223.19] 0.676
Wei 2020 2446.54 [-9763.08, 14656.17] 0.695
Pruis 2020 2328.23 [-8985.95, 13642.41] 0.687
Pruis 2020 2326.86 [-8980.71, 13634.43] 0.687
Dan 2014 1950.02 [-9363.82, 13263.87] 0.736
Wong 2021 2297.50 [-2771.32, 7366.33] 0.374
Dan 2014 1298.18 [-1.0e+04, 12612.12] 0.822
Wakase 2021 -596.92 [-1.2e+04, 10555.66] 0.916
-10000
0
10000
20000
Random-effects DerSimonian–Laird model
Sorted by: inmb

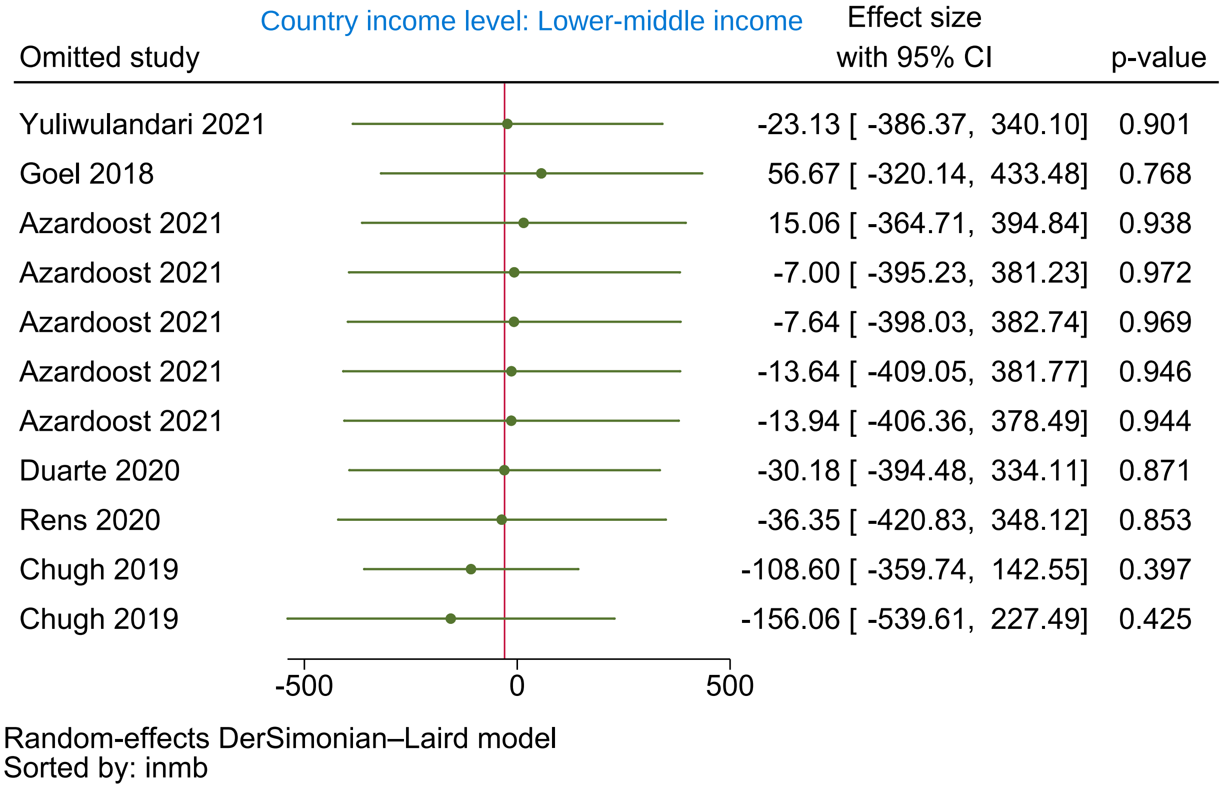
Country income level: Lower-middle income
Omitted study
Effect size with 95% CI
p-value
Yuliwulandari 2021 -23.13 [-386.37, 340.10] 0.901
Goel 2018 56.67 [-320.14, 433.48] 0.768
Azardoost 2021 15.06 [-364.71, 394.84] 0.938
Azardoost 2021 -7.00 [-395.23, 381.23] 0.972
Azardoost 2021 -7.64 [-398.03, 382.74] 0.969
Azardoost 2021 -13.64 [-409.05, 381.77] 0.946
Azardoost 2021 -13.94 [-406.36, 378.49] 0.944
Duarte 2020 -30.18 [-394.48, 334.11] 0.871
Rens 2020 -36.35 [-420.83, 348.12] 0.853
Chugh 2019 -108.60 [-359.74, 142.55] 0.397
Chugh 2019 -156.06 [-539.61, 227.49] 0.425
-500
0
500
Random-effects DerSimonian–Laird model
Sorted by: inmb

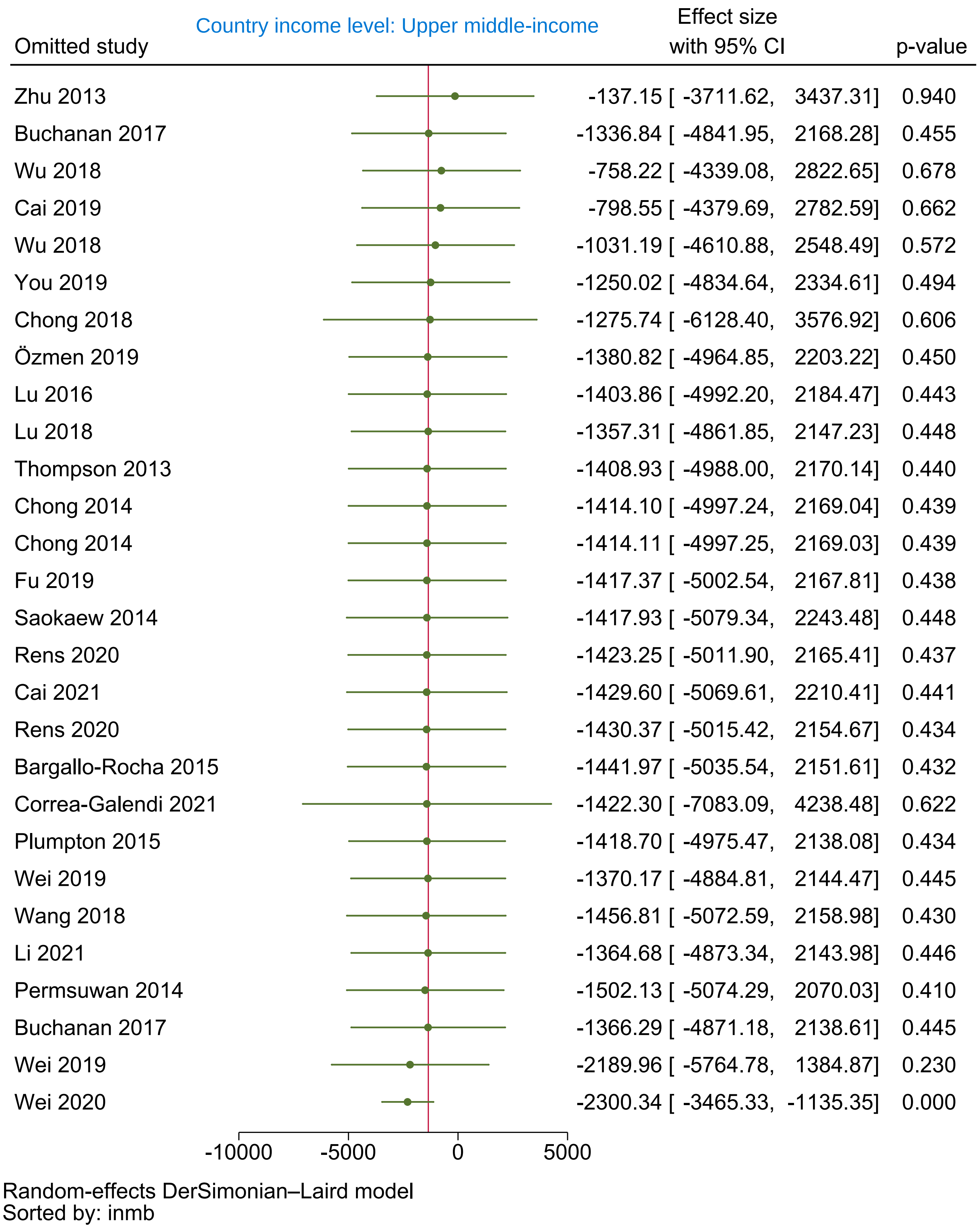
Country income level: Upper middle-income
Omitted study
Effect size with 95% CI
p-value
Zhu 2013 -137.15 [-3711.62, 3437.31] 0.940
Buchanan 2017 -1336.84 [-4841.95, 2168.28] 0.455
Wu 2018 -758.22 [-4339.08, 2822.65] 0.678
Cai 2019 -798.55 [-4379.69, 2782.59] 0.662
Wu 2018 -1031.19 [-4610.88, 2548.49] 0.572
You 2019 -1250.02 [-4834.64, 2334.61] 0.494
Chong 2018 -1275.74 [-6128.40, 3576.92] 0.606
Özmen 2019 -1380.82 [-4964.85, 2203.22] 0.450
Lu 2016 -1403.86 [-4992.20, 2184.47] 0.443
Lu 2018 -1357.31 [-4861.85, 2147.23] 0.448
Thompson 2013 -1408.93 [-4988.00, 2170.14] 0.440
Chong 2014 -1414.10 [-4997.24, 2169.04] 0.439
Chong 2014 -1414.11 [-4997.25, 2169.03] 0.439
Fu 2019 -1417.37 [-5002.54, 2167.81] 0.438
Saokaew 2014 -1417.93 [-5079.34, 2243.48] 0.448
Rens 2020 -1423.25 [-5011.90, 2165.41] 0.437
Cai 2021 -1429.60 [-5069.61, 2210.41] 0.441
Rens 2020 -1430.37 [-5015.42, 2154.67] 0.434
Bargallo-Rocha 2015 -1441.97 [-5035.54, 2151.61] 0.432
Correa-Galendi 2021 -1422.30 [-7083.09, 4238.48] 0.622
Plumpton 2015 -1418.70 [-4975.47, 2138.08] 0.434
Wei 2019 -1370.17 [-4884.81, 2144.47] 0.445
Wang 2018 -1456.81 [-5072.59, 2158.98] 0.430
Li 2021 -1364.68 [-4873.34, 2143.98] 0.446
Permsuwan 2014 -1502.13 [-5074.29, 2070.03] 0.410
Buchanan 2017 -1366.29 [-4871.18, 2138.61] 0.445
Wei 2019 -2189.96 [-5764.78, 1384.87] 0.230
Wei 2020 -2300.34 [-3465.33, -1135.35] 0.000
-10000
-5000
0
5000
Random-effects DerSimonian–Laird model
Sorted by: inmb

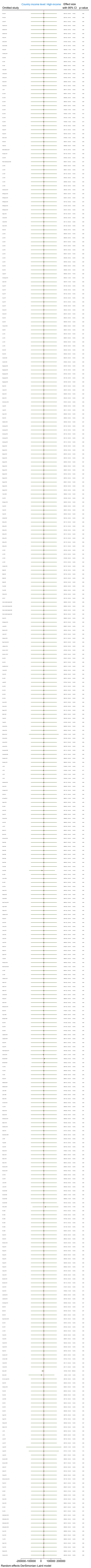

Country income level: High-income
Omitted study
Effect size with 95% CI
p-value
-200000 -100000 0 100000 200000
Random-effects DerSimonian–Laird model

PM Development Stage: Early EE

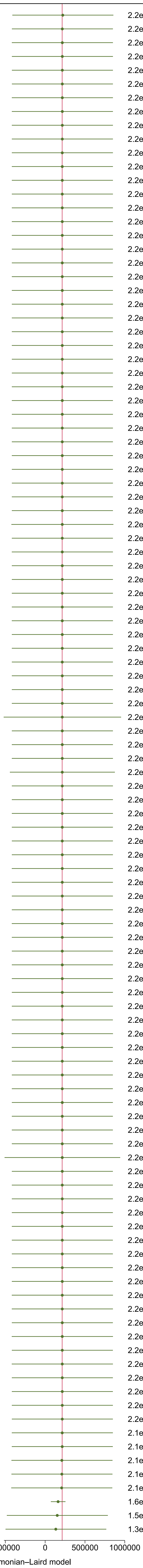

Omitted study	Effect size with 95% CI	p-value
Uhrmann 2020	2.2e+05 [-4.1e+05, 8.5e+05]	0.491
Sax 2014	2.2e+05 [-4.1e+05, 8.5e+05]	0.502
Wakase 2021	2.2e+05 [-4.1e+05, 8.5e+05]	0.502
Marguet 2016	2.2e+05 [-4.1e+05, 8.5e+05]	0.502
You 2012	2.2e+05 [-4.1e+05, 8.5e+05]	0.502
Buchanan 2017	2.2e+05 [-4.1e+05, 8.5e+05]	0.503
Marguet 2016	2.2e+05 [-4.1e+05, 8.5e+05]	0.503
Marguet 2016	2.2e+05 [-4.1e+05, 8.5e+05]	0.503
Handorf 2012	2.2e+05 [-4.1e+05, 8.5e+05]	0.503
Verhoef 2019	2.2e+05 [-4.1e+05, 8.5e+05]	0.503
Nelson 2013	2.2e+05 [-4.1e+05, 8.5e+05]	0.503
Chen 2015	2.2e+05 [-4.1e+05, 8.5e+05]	0.503
Handorf 2012	2.2e+05 [-4.1e+05, 8.5e+05]	0.503
Perez 2011	2.2e+05 [-4.1e+05, 8.5e+05]	0.503
Alkhatib 2020	2.2e+05 [-4.1e+05, 8.5e+05]	0.503
You 2019	2.2e+05 [-4.2e+05, 8.5e+05]	0.503
Saito 2019	2.2e+05 [-4.1e+05, 8.5e+05]	0.503
Ladabaum 2013	2.2e+05 [-4.1e+05, 8.5e+05]	0.503
Wu 2016	2.2e+05 [-4.1e+05, 8.5e+05]	0.503
Lotan 2018	2.2e+05 [-4.1e+05, 8.5e+05]	0.503
Luime 2015	2.2e+05 [-4.1e+05, 8.5e+05]	0.503
Biltaji 2021	2.2e+05 [-4.1e+05, 8.5e+05]	0.503
Mitropoulou 2014	2.2e+05 [-4.1e+05, 8.5e+05]	0.503
Lu 2018	2.2e+05 [-4.1e+05, 8.5e+05]	0.503
Teng 2020	2.2e+05 [-4.1e+05, 8.5e+05]	0.503
Alagoz 2016	2.2e+05 [-4.1e+05, 8.5e+05]	0.503
Chong 2014	2.2e+05 [-4.1e+05, 8.5e+05]	0.503
Chong 2014	2.2e+05 [-4.1e+05, 8.5e+05]	0.503
Sluiter 2019	2.2e+05 [-4.1e+05, 8.5e+05]	0.503
Hao 2019	2.2e+05 [-4.1e+05, 8.5e+05]	0.503
Saramago 2018	2.2e+05 [-4.1e+05, 8.5e+05]	0.503
Biltaji 2021	2.2e+05 [-4.1e+05, 8.5e+05]	0.503
Saramago 2018	2.2e+05 [-4.1e+05, 8.5e+05]	0.503
Saramago 2018	2.2e+05 [-4.1e+05, 8.5e+05]	0.503
Saramago 2018	2.2e+05 [-4.1e+05, 8.5e+05]	0.503
Saramago 2018	2.2e+05 [-4.1e+05, 8.5e+05]	0.503
Verhoef 2014	2.2e+05 [-4.1e+05, 8.5e+05]	0.503
Rens 2020	2.2e+05 [-4.2e+05, 8.5e+05]	0.507
Verhoef 2014	2.2e+05 [-4.1e+05, 8.5e+05]	0.503
Rens 2020	2.2e+05 [-4.2e+05, 8.5e+05]	0.503
Feller-Kopman 2017	2.2e+05 [-4.1e+05, 8.5e+05]	0.503
Biltaji 2021	2.2e+05 [-4.1e+05, 8.5e+05]	0.503
Forde 2016	2.2e+05 [-4.1e+05, 8.5e+05]	0.503
Lieberthal 2013	2.2e+05 [-4.1e+05, 8.5e+05]	0.503
Sluiter 2018	2.2e+05 [-4.2e+05, 8.5e+05]	0.503
Biltaji 2021	2.2e+05 [-4.1e+05, 8.5e+05]	0.503
Rens 2020	2.2e+05 [-4.2e+05, 8.5e+05]	0.503
Marguet 2016	2.2e+05 [-4.1e+05, 8.5e+05]	0.503
Kapoor 2020	2.2e+05 [-4.1e+05, 8.5e+05]	0.503
Lotan 2018	2.2e+05 [-4.1e+05, 8.5e+05]	0.503
Nelson 2013	2.2e+05 [-4.1e+05, 8.5e+05]	0.503
Chugh 2019	2.2e+05 [-5.2e+05, 9.5e+05]	0.565
Plumpton 2015	2.2e+05 [-4.1e+05, 8.5e+05]	0.503
Luime 2015	2.2e+05 [-4.1e+05, 8.5e+05]	0.503
Forde 2016	2.2e+05 [-4.1e+05, 8.5e+05]	0.503
Chugh 2019	2.2e+05 [-4.4e+05, 8.7e+05]	0.519
Li 2021	2.2e+05 [-4.1e+05, 8.5e+05]	0.503
Marguet 2016	2.2e+05 [-4.1e+05, 8.5e+05]	0.503
Wu 2016	2.2e+05 [-4.1e+05, 8.5e+05]	0.503
Ghatnekar 2013	2.2e+05 [-4.1e+05, 8.5e+05]	0.503
Catchpool 2019	2.2e+05 [-4.1e+05, 8.5e+05]	0.503
Lieberthal 2013	2.2e+05 [-4.1e+05, 8.5e+05]	0.503
Eccleston 2017	2.2e+05 [-4.2e+05, 8.5e+05]	0.504
Dong 2019	2.2e+05 [-4.1e+05, 8.5e+05]	0.503
Dong 2019	2.2e+05 [-4.1e+05, 8.5e+05]	0.503
Woods 2011	2.2e+05 [-4.1e+05, 8.5e+05]	0.503
Lim 2016	2.2e+05 [-4.2e+05, 8.5e+05]	0.503
AlMukdad 2021	2.2e+05 [-4.1e+05, 8.5e+05]	0.503
Marguet 2016	2.2e+05 [-4.1e+05, 8.5e+05]	0.503
Marguet 2016	2.2e+05 [-4.1e+05, 8.5e+05]	0.503
Woods 2011	2.2e+05 [-4.1e+05, 8.5e+05]	0.503
Cressman 2016	2.2e+05 [-4.1e+05, 8.5e+05]	0.503
Marguet 2016	2.2e+05 [-4.1e+05, 8.5e+05]	0.503
Blank 2015	2.2e+05 [-4.1e+05, 8.5e+05]	0.503
Jung 2021	2.2e+05 [-4.2e+05, 8.5e+05]	0.503
AlMukdad 2021	2.2e+05 [-4.1e+05, 8.5e+05]	0.503
Marguet 2016	2.2e+05 [-4.1e+05, 8.5e+05]	0.503
Nelson 2013	2.2e+05 [-4.1e+05, 8.5e+05]	0.503
Felix 2016	2.2e+05 [-4.1e+05, 8.5e+05]	0.503
Buchanan 2017	2.2e+05 [-4.1e+05, 8.5e+05]	0.503
Felix 2016	2.2e+05 [-4.1e+05, 8.5e+05]	0.503
Elbasha 2016	2.2e+05 [-4.1e+05, 8.5e+05]	0.503
You 2013	2.2e+05 [-4.1e+05, 8.5e+05]	0.503
Tanner 2020	2.2e+05 [-5.1e+05, 9.4e+05]	0.559
You 2012	2.2e+05 [-4.1e+05, 8.5e+05]	0.503
Marguet 2016	2.2e+05 [-4.1e+05, 8.5e+05]	0.503
Alkhatib 2018	2.2e+05 [-4.2e+05, 8.5e+05]	0.503
Hart 2019	2.2e+05 [-4.2e+05, 8.5e+05]	0.503
Marguet 2016	2.2e+05 [-4.2e+05, 8.5e+05]	0.503
Perez 2011	2.2e+05 [-4.2e+05, 8.5e+05]	0.503
Lotan 2018	2.2e+05 [-4.2e+05, 8.5e+05]	0.503
Retel 2018	2.2e+05 [-4.2e+05, 8.5e+05]	0.503
Schackman 2015	2.2e+05 [-4.2e+05, 8.5e+05]	0.503
Retel 2013	2.2e+05 [-4.2e+05, 8.5e+05]	0.504
Alkhatib 2018	2.2e+05 [-4.2e+05, 8.5e+05]	0.504
Hart 2019	2.2e+05 [-4.2e+05, 8.5e+05]	0.503
Rodríguez 2019	2.2e+05 [-4.2e+05, 8.5e+05]	0.504
Marguet 2016	2.2e+05 [-4.2e+05, 8.5e+05]	0.503
Cromwell 2016	2.2e+05 [-4.2e+05, 8.5e+05]	0.504
Retèl 2012	2.2e+05 [-4.2e+05, 8.5e+05]	0.504
Retèl 2012	2.2e+05 [-4.2e+05, 8.5e+05]	0.504
Das 2016	2.2e+05 [-4.2e+05, 8.5e+05]	0.504
Das 2016	2.2e+05 [-4.2e+05, 8.5e+05]	0.504
Chen 2018	2.1e+05 [-4.2e+05, 8.5e+05]	0.504
Das 2016	2.1e+05 [-4.2e+05, 8.5e+05]	0.504
Nherera 2011	2.1e+05 [-4.2e+05, 8.4e+05]	0.513
Bock 2015	2.1e+05 [-4.2e+05, 8.4e+05]	0.513
Machin 2018	2.1e+05 [-4.2e+05, 8.4e+05]	0.518
Bolous 2021	1.6e+05 [75056.55, 2.5e+05]	0.000
Cook 2020	1.5e+05 [-4.8e+05, 7.8e+05]	0.636
Bolous 2021	1.3e+05 [-4.9e+05, 7.6e+05]	0.676

Random-effects DerSimonian–Laird model
Sorted by: inmb

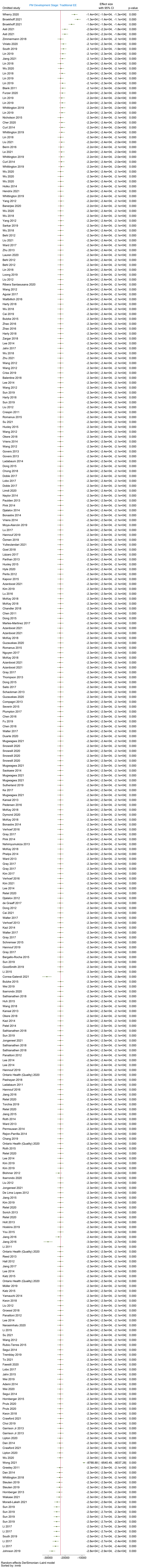

PM Development Stage: Traditional ES
Omitted study
Effect size with 95% CI
p-value
-30000 -20000 -10000
Random-effects DerSimonian–Laird model
Sorted by: mmb

Type of Funders: Public

Omitted study	Effect size with 95% CI	p-value
South 2019	4198.54 [-2961.90, 11358.98]	0.250
Lin 2018	388.64 [-6748.73, 7526.01]	0.915
Lin 2018	2770.42 [-4392.56, 9933.41]	0.448
Blank 2011	2749.60 [-4196.62, 9695.83]	0.438
Furzer 2020	2504.28 [-4649.06, 9657.63]	0.493
Nicholson 2015	286.59 [-6849.69, 7422.87]	0.937
Lin 2018	1053.75 [-6096.83, 8204.33]	0.773
Berm 2016	1603.21 [-5559.03, 8765.44]	0.661
Hendrix 2021	608.36 [-6540.73, 7757.46]	0.868
Yang 2012	941.14 [-6217.22, 8099.50]	0.797
Banerjee 2020	888.66 [-6266.58, 8043.90]	0.808
Wu 2018	837.64 [-6327.02, 8002.29]	0.819
Yang 2012	840.90 [-6319.07, 8000.87]	0.818
Sarkar 2019	814.88 [-6349.74, 7979.51]	0.824
Sax 2014	311.65 [-6826.49, 7449.79]	0.932
Wu 2018	620.91 [-6543.41, 7785.22]	0.865
Behl 2012	278.30 [-6858.27, 7414.86]	0.939
Marguet 2016	510.92 [-6653.86, 7675.70]	0.889
Zhu 2013	507.14 [-6654.16, 7668.44]	0.890
Behl 2012	279.64 [-6857.28, 7416.55]	0.939
Behl 2012	281.66 [-6855.59, 7418.90]	0.938
Lin 2018	472.36 [-6692.52, 7637.25]	0.897
Wang 2012	388.31 [-6770.85, 7547.46]	0.915
Marguet 2016	379.16 [-6781.93, 7540.25]	0.917
Wu 2018	395.54 [-6768.76, 7559.85]	0.914
Cai 2019	388.04 [-6776.17, 7552.25]	0.915
Marguet 2016	367.01 [-6797.06, 7531.07]	0.920
Zargar 2018	352.47 [-6811.07, 7516.00]	0.923
Jahn 2017	347.18 [-6817.96, 7512.32]	0.924
Wu 2018	347.25 [-6817.84, 7512.33]	0.924
Wang 2012	329.23 [-6833.42, 7491.88]	0.928
Wang 2012	327.72 [-6835.02, 7490.46]	0.929
Nelson 2013	275.62 [-6861.77, 7413.01]	0.940
Balentine 2018	295.55 [-6854.60, 7445.71]	0.935
Wang 2012	313.56 [-6849.54, 7476.67]	0.932
Crespin 2011	304.65 [-6859.91, 7469.21]	0.934
Romanus 2015	274.79 [-6862.44, 7412.01]	0.940
Saito 2019	304.43 [-6860.68, 7469.54]	0.934
Su 2021	303.35 [-6861.87, 7468.57]	0.934
Huxley 2015	302.58 [-6862.53, 7467.69]	0.934
Wang 2012	301.09 [-6862.78, 7464.95]	0.934
Vriens 2014	322.65 [-6712.10, 7357.39]	0.928
Wang 2012	299.47 [-6864.35, 7463.29]	0.935
Doble 2017	274.57 [-6862.75, 7411.88]	0.940
Doble 2017	273.94 [-6862.68, 7410.56]	0.940
Limdi 2020	282.86 [-6865.81, 7431.52]	0.938
Naylor 2014	293.12 [-6871.57, 7457.81]	0.936
Paulden 2013	290.58 [-6874.39, 7455.55]	0.937
Pink 2014	274.07 [-6862.94, 7411.07]	0.940
Bonastre 2014	288.78 [-6876.16, 7453.73]	0.937
Vriens 2014	287.73 [-6877.54, 7453.00]	0.937
Lu 2017	286.80 [-6877.80, 7451.41]	0.937
Yuliwulandari 2021	282.15 [-6883.14, 7447.43]	0.938
Lotan 2018	275.01 [-6866.17, 7416.20]	0.940
Lázaro 2017	281.47 [-6883.45, 7446.39]	0.939
Huxley 2015	280.56 [-6884.62, 7445.75]	0.939
Luime 2015	278.20 [-6882.18, 7438.59]	0.939
Martes-Martinez 2017	275.55 [-6874.99, 7426.09]	0.940
Guzauskas 2020	275.07 [-6872.34, 7422.48]	0.940
Romanus 2015	273.69 [-6863.55, 7410.94]	0.940
Gray 2017	277.03 [-6888.10, 7442.16]	0.940
Thompson 2013	277.05 [-6888.24, 7442.35]	0.940
Schackman 2013	273.57 [-6862.76, 7409.90]	0.940
Guzauskas 2020	276.42 [-6888.83, 7441.66]	0.940
Compagni 2013	276.38 [-6888.90, 7441.66]	0.940
Severin 2015	276.13 [-6888.76, 7441.02]	0.940
Plumpton 2017	276.15 [-6888.56, 7440.85]	0.940
Fu 2019	276.02 [-6889.01, 7441.06]	0.940
Chen 2016	276.06 [-6888.75, 7440.87]	0.940
Walter 2017	275.82 [-6889.42, 7441.06]	0.940
Saramago 2018	275.79 [-6889.40, 7440.97]	0.940
Duarte 2020	275.80 [-6889.50, 7441.11]	0.940
Saramago 2018	275.78 [-6889.40, 7440.95]	0.940
Saokaew 2014	277.57 [-6876.73, 7431.88]	0.939
Saramago 2018	275.72 [-6889.43, 7440.88]	0.940
Sutherland 2019	275.76 [-6889.49, 7441.00]	0.940
Saramago 2018	275.72 [-6889.46, 7440.89]	0.940
Ke 2017	275.70 [-6889.31, 7440.70]	0.940
Saramago 2018	275.70 [-6889.46, 7440.86]	0.940
Pedersen 2016	275.64 [-6889.05, 7440.34]	0.940
Dymond 2020	275.62 [-6889.65, 7440.89]	0.940
Rens 2020	277.16 [-6878.63, 7432.96]	0.939
Bonastre 2014	275.12 [-6884.56, 7434.79]	0.940
Gray 2017	275.19 [-6888.50, 7438.88]	0.940
Pink 2014	273.67 [-6864.67, 7412.02]	0.940
Nshimyumukiza 2013	275.22 [-6890.06, 7440.49]	0.940
Rens 2020	275.05 [-6889.66, 7439.75]	0.940
Ward 2013	274.83 [-6890.42, 7440.09]	0.940
Gray 2017	274.68 [-6890.55, 7439.91]	0.940
Gray 2017	274.68 [-6890.54, 7439.90]	0.940
Djalalov 2012	274.09 [-6891.15, 7439.33]	0.940
Cai 2021	274.80 [-6883.91, 7433.52]	0.940
Rens 2020	273.68 [-6891.43, 7438.78]	0.940
Walter 2017	273.51 [-6891.73, 7438.75]	0.940
Verhoef 2013	273.60 [-6890.66, 7437.86]	0.940
Walter 2017	273.09 [-6892.16, 7438.35]	0.940
Marguet 2016	273.01 [-6889.94, 7435.96]	0.940
Gray 2017	272.92 [-6892.31, 7438.15]	0.940
Gray 2017	272.31 [-6892.93, 7437.56]	0.941
Lotan 2018	272.83 [-6876.63, 7422.30]	0.940
Nelson 2013	273.39 [-6865.11, 7411.89]	0.940
GoodSmith 2019	277.90 [-6848.47, 7404.26]	0.939
Luime 2015	269.99 [-6895.21, 7435.19]	0.941
Wei 2019	270.67 [-6888.88, 7430.21]	0.941
Wang 2018	269.27 [-6893.85, 7432.39]	0.941
Marguet 2016	265.89 [-6897.83, 7429.62]	0.942
Wu 2016	269.11 [-6882.60, 7420.81]	0.941
Ghatnekar 2013	266.45 [-6892.46, 7425.36]	0.942
Panattoni 2012	273.49 [-6862.73, 7409.72]	0.940
Ontario Health (Quality) 2020	262.31 [-6902.96, 7427.58]	0.943
Pashayan 2018	260.93 [-6904.43, 7426.28]	0.943
Ladabaum 2011	260.77 [-6904.16, 7425.71]	0.943
Ward 2013	257.29 [-6908.05, 7422.62]	0.944
Permsuwan 2014	256.99 [-6908.21, 7422.19]	0.944
Ontario Health (Quality) 2020	253.27 [-6912.10, 7418.63]	0.945
Lim 2016	251.28 [-6914.62, 7417.18]	0.945
Marguet 2016	255.44 [-6904.20, 7415.08]	0.944
Marguet 2016	255.43 [-6902.51, 7413.37]	0.944
Marguet 2016	252.16 [-6906.92, 7411.23]	0.945
Sorich 2013	243.19 [-6922.18, 7408.56]	0.947
You 2015	250.79 [-6904.76, 7406.35]	0.945
Ontario Health (Quality) 2020	230.85 [-6935.22, 7396.91]	0.950
Marguet 2016	245.90 [-6909.15, 7400.94]	0.946
Reed 2013	229.43 [-6942.30, 7401.16]	0.950
Nelson 2013	267.76 [-6872.18, 7407.70]	0.941
Ontario Health (Quality) 2020	213.90 [-6965.93, 7393.72]	0.953
Müller 2019	224.22 [-6936.95, 7385.39]	0.951
Kwon 2018	205.72 [-6959.55, 7370.99]	0.955
You 2013	223.26 [-6932.15, 7378.67]	0.951
Panattoni 2012	193.81 [-6971.03, 7358.66]	0.958
Su 2021	267.64 [-6870.32, 7405.60]	0.941
Tremblay 2019	166.46 [-6999.95, 7332.87]	0.964
Marguet 2016	158.01 [-7007.48, 7323.51]	0.966
Fawsitt 2020	178.92 [-6979.38, 7337.22]	0.961
Wei 2019	136.53 [-7036.96, 7310.02]	0.970
Hart 2019	240.06 [-6902.75, 7382.86]	0.947
Marguet 2016	126.01 [-7039.12, 7291.13]	0.973
Ademi 2014	66.07 [-7432.28, 7564.42]	0.986
Lotan 2018	135.88 [-7026.38, 7298.15]	0.970
Wei 2020	-7.69 [-7949.18, 7933.80]	0.998
Schackman 2015	246.99 [-6893.83, 7387.82]	0.946
Retel 2013	106.55 [-7069.32, 7282.42]	0.977
Hart 2019	94.98 [-7070.28, 7260.24]	0.979
Marguet 2016	49.86 [-7115.20, 7214.91]	0.989
Kwon 2018	-8.62 [-7173.99, 7156.74]	0.998
Retèl 2012	-101.45 [-7267.29, 7064.38]	0.978
Retèl 2012	-132.83 [-7298.15, 7032.49]	0.971
Wong 2021	138.50 [-3435.42, 3712.43]	0.939
South 2019	-3901.79 [-1.1e+04, 3253.84]	0.285
Nherera 2011	-4235.04 [-1.1e+04, 2905.30]	0.245
Machin 2018	-6077.01 [-1.3e+04, 1083.27]	0.096

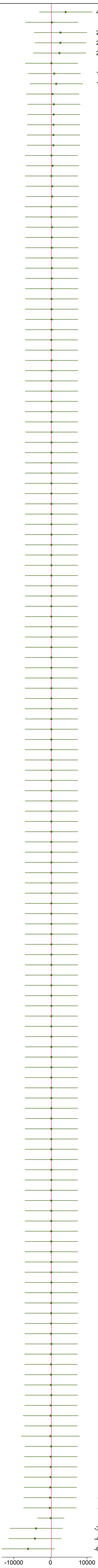

Random-effects DerSimonian–Laird model
Sorted by: inmb

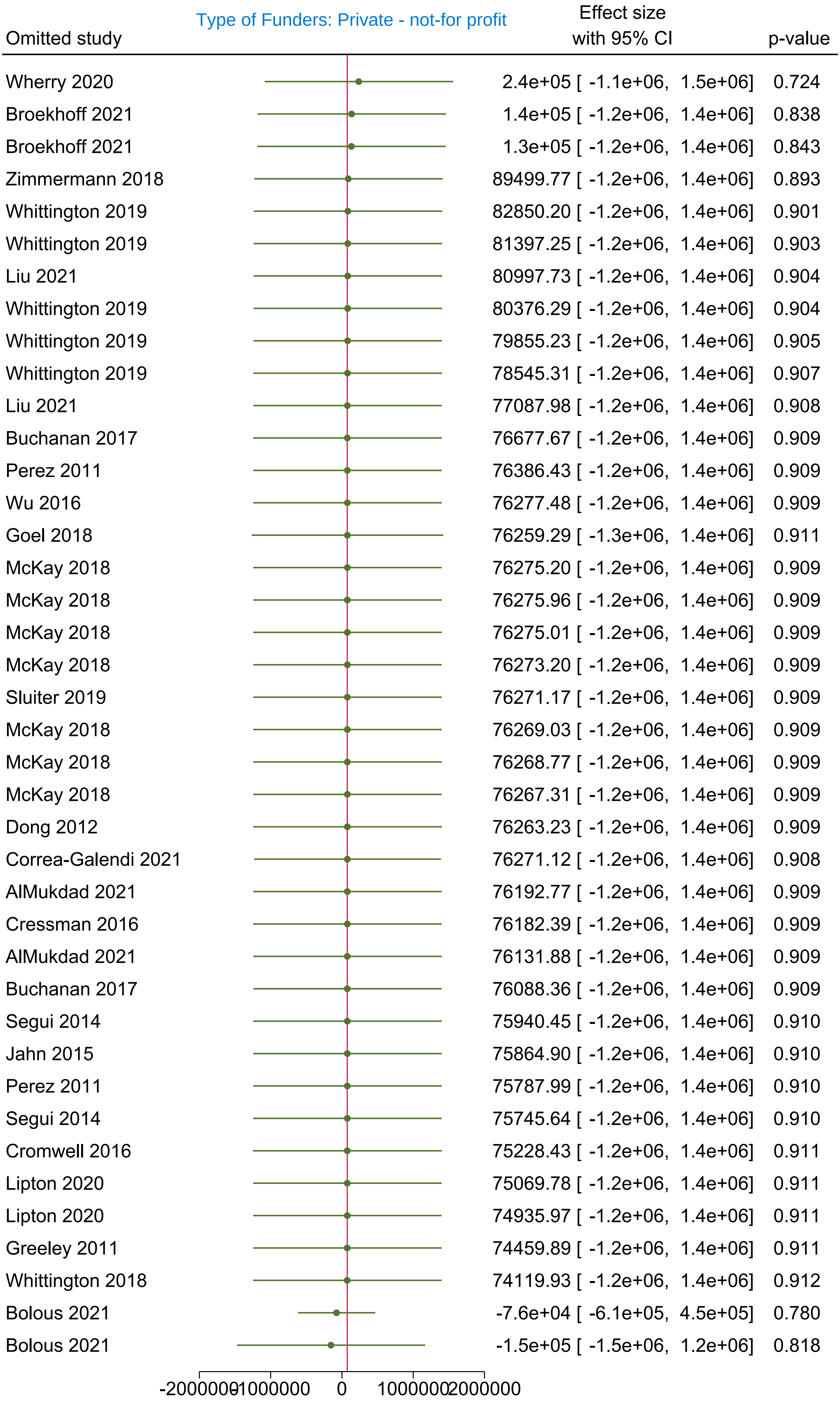

Random-effects DerSimonian–Laird model
Sorted by: inmb

Type of Funders: Private - for-profit

Omitted study	Effect size with 95% CI	p-value
Viriato 2020	1.6e+05 [1.5e+05, 1.7e+05]	0.000
Lin 2019	1.6e+05 [1.5e+05, 1.7e+05]	0.000
Lin 2019	1.5e+05 [1.4e+05, 1.6e+05]	0.000
Lin 2019	1.6e+05 [1.5e+05, 1.7e+05]	0.000
Lin 2019	1.6e+05 [1.5e+05, 1.7e+05]	0.000
Lin 2019	1.6e+05 [1.5e+05, 1.7e+05]	0.000
Lin 2019	1.6e+05 [1.5e+05, 1.7e+05]	0.000
Holko 2014	1.6e+05 [1.5e+05, 1.6e+05]	0.000
Wakase 2021	1.5e+05 [1.5e+05, 1.6e+05]	0.000
Lauren 2020	1.5e+05 [1.5e+05, 1.6e+05]	0.000
Loong 2019	1.5e+05 [1.5e+05, 1.6e+05]	0.000
Ribera Santasusana 2020	1.5e+05 [1.5e+05, 1.6e+05]	0.000
Harty 2018	1.5e+05 [1.4e+05, 1.6e+05]	0.000
Harty 2018	1.5e+05 [1.4e+05, 1.6e+05]	0.000
Handorf 2012	1.5e+05 [1.5e+05, 1.6e+05]	0.000
Zhu 2021	1.5e+05 [1.5e+05, 1.6e+05]	0.000
Handorf 2012	1.5e+05 [1.5e+05, 1.6e+05]	0.000
Harty 2018	1.5e+05 [1.4e+05, 1.6e+05]	0.000
Ladabaum 2014	1.5e+05 [1.5e+05, 1.6e+05]	0.000
Ladabaum 2013	1.5e+05 [1.5e+05, 1.6e+05]	0.000
Moya-Alarcón 2019	1.5e+05 [1.5e+05, 1.6e+05]	0.000
Parthan 2013	1.5e+05 [1.5e+05, 1.6e+05]	0.000
Lu 2016	1.5e+05 [1.5e+05, 1.6e+05]	0.000
Chandler 2018	1.5e+05 [1.5e+05, 1.6e+05]	0.000
Mitropoulou 2014	1.5e+05 [1.5e+05, 1.6e+05]	0.000
Lu 2018	1.5e+05 [1.4e+05, 1.6e+05]	0.000
Nguyen 2017	1.5e+05 [1.5e+05, 1.6e+05]	0.000
Alagoz 2016	1.5e+05 [1.5e+05, 1.6e+05]	0.000
Hao 2019	1.5e+05 [1.4e+05, 1.6e+05]	0.000
Kansal 2013	1.5e+05 [1.5e+05, 1.6e+05]	0.000
Kim 2017	1.5e+05 [1.5e+05, 1.6e+05]	0.000
Forde 2016	1.5e+05 [1.5e+05, 1.6e+05]	0.000
Lieberthal 2013	1.5e+05 [1.4e+05, 1.6e+05]	0.000
Bargallo-Rocha 2015	1.5e+05 [1.5e+05, 1.6e+05]	0.000
Huh 2015	1.5e+05 [1.5e+05, 1.6e+05]	0.000
Forde 2016	1.5e+05 [1.5e+05, 1.6e+05]	0.000
Kansal 2013	1.5e+05 [1.5e+05, 1.6e+05]	0.000
Jongeneel 2021	1.5e+05 [1.5e+05, 1.6e+05]	0.000
Lieberthal 2013	1.5e+05 [1.4e+05, 1.6e+05]	0.000
Torchia 2019	1.5e+05 [1.5e+05, 1.6e+05]	0.000
Eccleston 2017	1.5e+05 [1.5e+05, 1.6e+05]	0.000
Roth 2014	1.5e+05 [1.5e+05, 1.6e+05]	0.000
Woods 2011	1.5e+05 [1.5e+05, 1.6e+05]	0.000
Woods 2011	1.5e+05 [1.4e+05, 1.6e+05]	0.000
Blohmer 2012	1.5e+05 [1.3e+05, 1.6e+05]	0.000
Jongeneel 2021	1.5e+05 [1.5e+05, 1.6e+05]	0.000
De Lima Lopes 2012	1.5e+05 [1.4e+05, 1.6e+05]	0.000
Holt 2013	1.5e+05 [1.5e+05, 1.6e+05]	0.000
Li 2011	1.5e+05 [1.5e+05, 1.6e+05]	0.000
Felix 2016	1.5e+05 [1.5e+05, 1.6e+05]	0.000
Felix 2016	1.5e+05 [1.5e+05, 1.6e+05]	0.000
Yamauchi 2014	1.5e+05 [1.5e+05, 1.6e+05]	0.000
Groessl 2018	1.5e+05 [1.4e+05, 1.6e+05]	0.000
Elbasha 2016	1.5e+05 [1.5e+05, 1.6e+05]	0.000
Tanner 2020	1.5e+05 [1.3e+05, 1.6e+05]	0.000
Hornberger 2015	1.5e+05 [1.4e+05, 1.6e+05]	0.000
Choi 2019	1.5e+05 [1.4e+05, 1.6e+05]	0.000
Garrison Jr 2013	1.5e+05 [1.4e+05, 1.6e+05]	0.000
Garrison Jr 2013	1.5e+05 [1.4e+05, 1.6e+05]	0.000
Dan 2014	1.5e+05 [1.4e+05, 1.6e+05]	0.000
Das 2016	1.5e+05 [1.4e+05, 1.6e+05]	0.000
Das 2016	1.5e+05 [1.4e+05, 1.6e+05]	0.000
Das 2016	1.5e+05 [1.4e+05, 1.6e+05]	0.000
Dan 2014	1.5e+05 [1.4e+05, 1.6e+05]	0.000
Steuten 2019	1.5e+05 [1.4e+05, 1.6e+05]	0.000
Steuten 2019	1.5e+05 [1.4e+05, 1.6e+05]	0.000
Hornberger 2013	1.5e+05 [1.4e+05, 1.6e+05]	0.000
Wakase 2021	1.5e+05 [1.4e+05, 1.6e+05]	0.000
Moradi-Lakeh 2021	1.5e+05 [1.4e+05, 1.6e+05]	0.000
Li 2017	1.5e+05 [1.4e+05, 1.6e+05]	0.000
Li 2017	1.5e+05 [1.4e+05, 1.6e+05]	0.000
Li 2017	1.4e+05 [1.4e+05, 1.5e+05]	0.000
Li 2017	1.4e+05 [1.4e+05, 1.5e+05]	0.000
Johnson 2019	1.4e+05 [1.3e+05, 1.4e+05]	0.000
Cook 2020	48302.92 [43317.92, 53287.91]	0.000

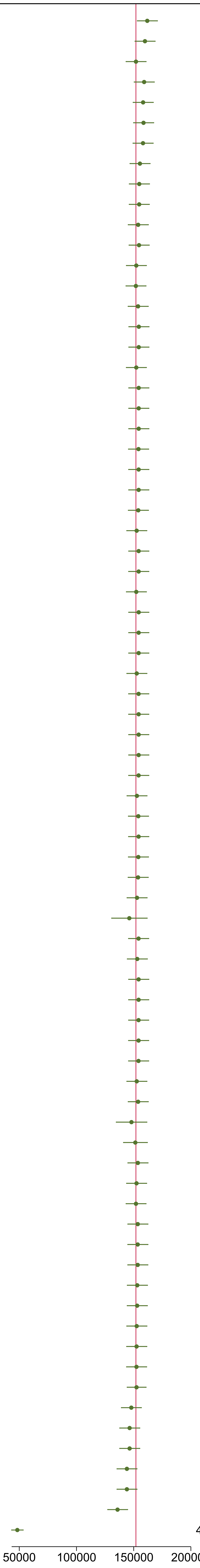

Random-effects DerSimonian–Laird model
Sorted by: inmb

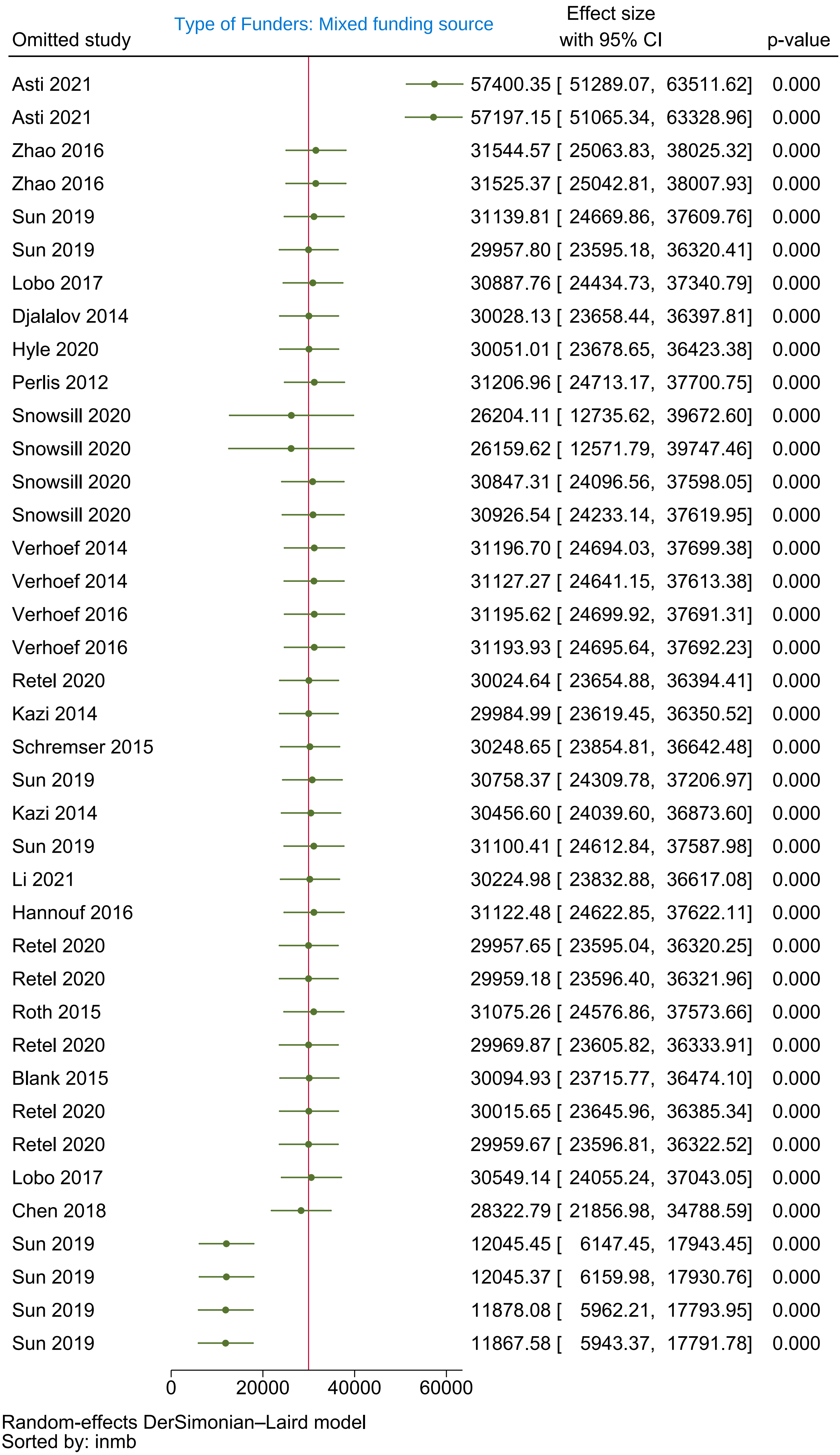
Omitted study
Type of Funders: Mixed funding source
Effect size with 95% CI
p-value
Asti 2021 57400.35 [51289.07, 63511.62] 0.000
Asti 2021 57197.15 [51065.34, 63328.96] 0.000
Zhao 2016 31544.57 [25063.83, 38025.32] 0.000
Zhao 2016 31525.37 [25042.81, 38007.93] 0.000
Sun 2019 31139.81 [24669.86, 37609.76] 0.000
Sun 2019 29957.80 [23595.18, 36320.41] 0.000
Lobo 2017 30887.76 [24434.73, 37340.79] 0.000
Djalalov 2014 30028.13 [23658.44, 36397.81] 0.000
Hyle 2020 30051.01 [23678.65, 36423.38] 0.000
Perlis 2012 31206.96 [24713.17, 37700.75] 0.000
Snowsill 2020 26204.11 [12735.62, 39672.60] 0.000
Snowsill 2020 26159.62 [12571.79, 39747.46] 0.000
Snowsill 2020 30847.31 [24096.56, 37598.05] 0.000
Snowsill 2020 30926.54 [24233.14, 37619.95] 0.000
Verhoef 2014 31196.70 [24694.03, 37699.38] 0.000
Verhoef 2014 31127.27 [24641.15, 37613.38] 0.000
Verhoef 2016 31195.62 [24699.92, 37691.31] 0.000
Verhoef 2016 31193.93 [24695.64, 37692.23] 0.000
Retel 2020 30024.64 [23654.88, 36394.41] 0.000
Kazi 2014 29984.99 [23619.45, 36350.52] 0.000
Schremser 2015 30248.65 [23854.81, 36642.48] 0.000
Sun 2019 30758.37 [24309.78, 37206.97] 0.000
Kazi 2014 30456.60 [24039.60, 36873.60] 0.000
Sun 2019 31100.41 [24612.84, 37587.98] 0.000
Li 2021 30224.98 [23832.88, 36617.08] 0.000
Hannouf 2016 31122.48 [24622.85, 37622.11] 0.000
Retel 2020 29957.65 [23595.04, 36320.25] 0.000
Retel 2020 29959.18 [23596.40, 36321.96] 0.000
Roth 2015 31075.26 [24576.86, 37573.66] 0.000
Retel 2020 29969.87 [23605.82, 36333.91] 0.000
Blank 2015 30094.93 [23715.77, 36474.10] 0.000
Retel 2020 30015.65 [23645.96, 36385.34] 0.000
Retel 2020 29959.67 [23596.81, 36322.52] 0.000
Lobo 2017 30549.14 [24055.24, 37043.05] 0.000
Chen 2018 28322.79 [21856.98, 34788.59] 0.000
Sun 2019 12045.45 [6147.45, 17943.45] 0.000
Sun 2019 12045.37 [6159.98, 17930.76] 0.000
Sun 2019 11878.08 [5962.21, 17793.95] 0.000
Sun 2019 11867.58 [5943.37, 17791.78] 0.000
0
20000
40000
60000
Random-effects DerSimonian–Laird model
Sorted by: inmb

Type of Funders: Not stated

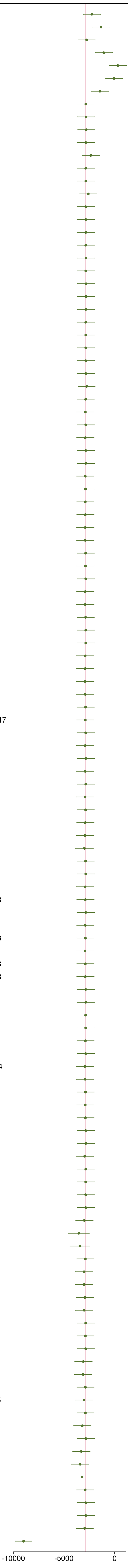

Omitted study	Effect size with 95% CI	p-value
Uhrmann 2020	-2241.38 [-3087.16, -1395.59]	0.000
Jiang 2021	-1338.01 [-2184.17, -491.85]	0.002
Wu 2020	-2759.54 [-3607.13, -1911.95]	0.000
Cher 2020	-1065.71 [-1913.08, -218.33]	0.014
Curl 2014	313.35 [-525.78, 1152.47]	0.464
Le 2021	-51.48 [-884.98, 782.02]	0.904
Curl 2014	-1446.13 [-2295.73, -596.53]	0.001
Wu 2020	-2840.59 [-3688.23, -1992.95]	0.000
Wu 2020	-2831.18 [-3678.85, -1983.51]	0.000
Wu 2020	-2800.59 [-3648.36, -1952.82]	0.000
Wu 2020	-2845.59 [-3693.22, -1997.95]	0.000
Ward 2017	-2360.52 [-3214.27, -1506.76]	0.000
Liu 2012	-2848.50 [-3696.13, -2000.88]	0.000
You 2012	-2843.60 [-3691.35, -1995.84]	0.000
Aguiar 2017	-2598.75 [-3453.43, -1744.07]	0.000
Wallbillich 2016	-2848.58 [-3696.20, -2000.96]	0.000
Butzke 2015	-2848.68 [-3696.30, -2001.06]	0.000
Lee 2014	-2848.24 [-3695.89, -2000.60]	0.000
Verhoef 2019	-2848.68 [-3696.30, -2001.06]	0.000
Criss 2019	-2833.05 [-3682.69, -1983.41]	0.000
Lee 2014	-2848.14 [-3695.85, -2000.42]	0.000
Chen 2015	-2824.47 [-3676.35, -1972.59]	0.000
Alkhatib 2020	-2825.81 [-3681.03, -1970.59]	0.000
You 2019	-2832.96 [-3688.34, -1977.57]	0.000
Liu 2012	-2848.66 [-3696.30, -2001.03]	0.000
Okere 2018	-2848.31 [-3696.54, -2000.08]	0.000
Govers 2013	-2848.46 [-3696.80, -2000.11]	0.000
Govers 2013	-2848.69 [-3696.32, -2001.06]	0.000
Dong 2015	-2847.82 [-3700.02, -1995.62]	0.000
Chong 2018	-2759.46 [-3586.41, -1932.51]	0.000
Hannouf 2019	-2853.32 [-3701.95, -2004.70]	0.000
Özmen 2019	-2886.14 [-3741.52, -2030.75]	0.000
Kapoor 2015	-2848.70 [-3696.32, -2001.08]	0.000
Azardoost 2021	-2893.19 [-3748.03, -2038.35]	0.000
Kim 2019	-2853.89 [-3702.38, -2005.39]	0.000
Biltaji 2021	-2848.91 [-3696.56, -2001.26]	0.000
Chen 2011	-2898.46 [-3754.08, -2042.85]	0.000
Dong 2015	-2872.34 [-3723.77, -2020.91]	0.000
Azardoost 2021	-2893.97 [-3747.95, -2040.00]	0.000
Azardoost 2021	-2886.36 [-3737.85, -2034.86]	0.000
Azardoost 2021	-2893.63 [-3747.18, -2040.09]	0.000
Azardoost 2021	-2895.98 [-3750.26, -2041.70]	0.000
Teng 2020	-2856.20 [-3705.00, -2007.40]	0.000
Dong 2015	-2879.30 [-3731.70, -2026.91]	0.000
Saito 2017	-2848.71 [-3696.33, -2001.09]	0.000
Chong 2014	-2898.55 [-3753.78, -2043.32]	0.000
Chong 2014	-2898.55 [-3753.78, -2043.32]	0.000
Chen 2016	-2866.31 [-3716.57, -2016.06]	0.000
Biltaji 2021	-2848.85 [-3696.49, -2001.21]	0.000
Mugwagwa 2021	-2848.79 [-3696.42, -2001.15]	0.000
Mugwagwa 2021	-2903.28 [-3758.97, -2047.60]	0.000
Mugwagwa 2021	-2903.49 [-3759.19, -2047.78]	0.000
Mugwagwa 2021	-2903.17 [-3758.83, -2047.51]	0.000
Mugwagwa 2021	-2903.43 [-3759.13, -2047.74]	0.000
Phelps 2014	-2860.45 [-3709.76, -2011.14]	0.000
Feller-Kopman 2017	-2892.08 [-3745.84, -2038.32]	0.000
Biltaji 2021	-2851.17 [-3699.14, -2003.21]	0.000
Kim 2021	-2906.21 [-3761.85, -2050.56]	0.000
Lee 2014	-2849.32 [-3697.03, -2001.62]	0.000
Sluiter 2018	-2906.71 [-3762.38, -2051.03]	0.000
Biltaji 2021	-2849.22 [-3696.91, -2001.53]	0.000
de Graaff 2017	-2910.13 [-3766.57, -2053.70]	0.000
Hannouf 2019	-2863.62 [-3713.19, -2014.06]	0.000
Kapoor 2020	-2891.65 [-3744.78, -2038.52]	0.000
Li 2015	-2909.14 [-3764.27, -2054.01]	0.000
Chugh 2019	-2981.11 [-3857.56, -2104.66]	0.000
Plumpton 2015	-2863.87 [-3713.32, -2014.42]	0.000
Butzke 2015	-2848.73 [-3696.35, -2001.10]	0.000
Ibarrondo 2020	-2906.58 [-3760.98, -2052.17]	0.000
Sathianathen 2018	-2880.43 [-3731.77, -2029.09]	0.000
Okere 2018	-2850.40 [-3698.21, -2002.59]	0.000
Patel 2014	-2896.50 [-3749.51, -2043.50]	0.000
Sathianathen 2018	-2882.15 [-3733.52, -2030.77]	0.000
Chugh 2019	-2928.64 [-3786.17, -2071.11]	0.000
Sathianathen 2018	-2885.40 [-3736.76, -2034.05]	0.000
Sathianathen 2018	-2885.37 [-3736.71, -2034.02]	0.000
Lee 2014	-2858.89 [-3707.52, -2010.26]	0.000
Lee 2014	-2849.30 [-3696.98, -2001.62]	0.000
Hannouf 2019	-2856.84 [-3705.25, -2008.43]	0.000
Jiang 2016	-2851.33 [-3699.18, -2003.48]	0.000
Catchpool 2019	-2874.63 [-3724.44, -2024.81]	0.000
Jiang 2015	-2856.31 [-3704.57, -2008.06]	0.000
Rejon-Parrilla 2014	-2938.88 [-3793.66, -2084.10]	0.000
Dong 2019	-2911.44 [-3764.04, -2058.84]	0.000
Chang 2019	-2868.05 [-3717.17, -2018.92]	0.000
Dong 2019	-2896.98 [-3748.31, -2045.64]	0.000
Lee 2014	-2879.48 [-3729.22, -2029.74]	0.000
Kim 2019	-2853.17 [-3701.09, -2005.24]	0.000
Kim 2019	-2848.89 [-3696.52, -2001.26]	0.000
Ibarrondo 2020	-2952.29 [-3806.66, -2097.92]	0.000
Liu 2012	-2848.99 [-3696.63, -2001.35]	0.000
Jiang 2015	-2851.23 [-3699.01, -2003.45]	0.000
Kim 2019	-2851.92 [-3699.74, -2004.10]	0.000
Hoskins 2019	-2852.30 [-3700.14, -2004.47]	0.000
Jung 2021	-2986.21 [-3842.02, -2130.41]	0.000
Jiang 2016	-3528.61 [-4549.79, -2507.42]	0.000
Jiang 2016	-3420.90 [-4408.68, -2433.13]	0.000
Hall 2012	-2888.56 [-3738.20, -2038.93]	0.000
Jiang 2017	-3014.70 [-3870.46, -2158.94]	0.000
Lee 2014	-3010.16 [-3865.36, -2154.97]	0.000
Katz 2015	-2939.27 [-3790.85, -2087.70]	0.000
Katz 2015	-3010.25 [-3864.19, -2156.32]	0.000
Liu 2012	-2851.02 [-3698.72, -2003.32]	0.000
Lee 2014	-2881.78 [-3730.40, -2033.16]	0.000
You 2012	-2852.32 [-3700.04, -2004.59]	0.000
Narasimhalu 2020	-3081.96 [-3936.56, -2227.36]	0.000
Li 2015	-3100.41 [-3955.50, -2245.31]	0.000
Wang 2012	-2880.75 [-3729.31, -2032.19]	0.000
Rubio-Terres 2015	-3022.67 [-3875.29, -2170.05]	0.000
To 2021	-2882.33 [-3730.80, -2033.86]	0.000
Alkhatib 2018	-3192.45 [-4047.58, -2337.32]	0.000
Retel 2018	-2849.06 [-3696.68, -2001.43]	0.000
Alkhatib 2018	-3286.18 [-4139.43, -2432.93]	0.000
Rodríguez 2019	-3404.67 [-4251.88, -2557.46]	0.000
Pruis 2020	-3223.23 [-4075.64, -2370.83]	0.000
Pruis 2020	-2903.10 [-3751.38, -2054.81]	0.000
Crawford 2021	-2848.72 [-3696.34, -2001.10]	0.000
Crawford 2021	-2848.72 [-3696.34, -2001.10]	0.000
Wu 2020	-2959.42 [-3807.72, -2111.12]	0.000
Bock 2015	-8988.61 [-9794.97, -8182.25]	0.000

Random-effects DerSimonian–Laird model
Sorted by: inmb

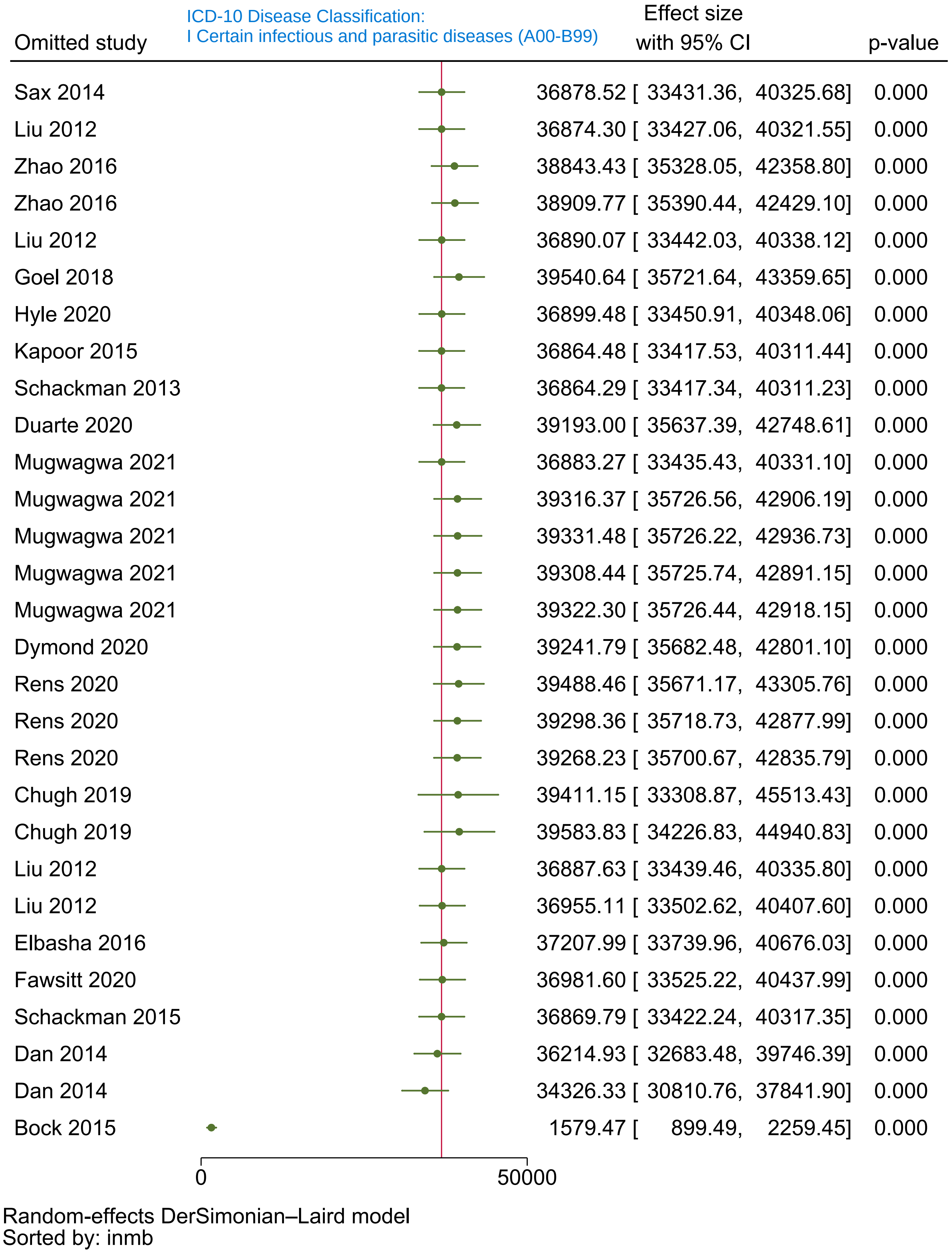
Omitted study
ICD-10 Disease Classification:
I Certain infectious and parasitic diseases (A00-B99)
Effect size with 95% CI
p-value
Sax 2014 36878.52 [33431.36, 40325.68] 0.000
Liu 2012 36874.30 [33427.06, 40321.55] 0.000
Zhao 2016 38843.43 [35328.05, 42358.80] 0.000
Zhao 2016 38909.77 [35390.44, 42429.10] 0.000
Liu 2012 36890.07 [33442.03, 40338.12] 0.000
Goel 2018 39540.64 [35721.64, 43359.65] 0.000
Hyle 2020 36899.48 [33450.91, 40348.06] 0.000
Kapoor 2015 36864.48 [33417.53, 40311.44] 0.000
Schackman 2013 36864.29 [33417.34, 40311.23] 0.000
Duarte 2020 39193.00 [35637.39, 42748.61] 0.000
Mugwagwa 2021 36883.27 [33435.43, 40331.10] 0.000
Mugwagwa 2021 39316.37 [35726.56, 42906.19] 0.000
Mugwagwa 2021 39331.48 [35726.22, 42936.73] 0.000
Mugwagwa 2021 39308.44 [35725.74, 42891.15] 0.000
Mugwagwa 2021 39322.30 [35726.44, 42918.15] 0.000
Dymond 2020 39241.79 [35682.48, 42801.10] 0.000
Rens 2020 39488.46 [35671.17, 43305.76] 0.000
Rens 2020 39298.36 [35718.73, 42877.99] 0.000
Rens 2020 39268.23 [35700.67, 42835.79] 0.000
Chugh 2019 39411.15 [33308.87, 45513.43] 0.000
Chugh 2019 39583.83 [34226.83, 44940.83] 0.000
Liu 2012 36887.63 [33439.46, 40335.80] 0.000
Liu 2012 36955.11 [33502.62, 40407.60] 0.000
Elbasha 2016 37207.99 [33739.96, 40676.03] 0.000
Fawsitt 2020 36981.60 [33525.22, 40437.99] 0.000
Schackman 2015 36869.79 [33422.24, 40317.35] 0.000
Dan 2014 36214.93 [32683.48, 39746.39] 0.000
Dan 2014 34326.33 [30810.76, 37841.90] 0.000
Bock 2015 1579.47 [899.49, 2259.45] 0.000
0
50000
Random-effects DerSimonian–Laird model
Sorted by: inmb

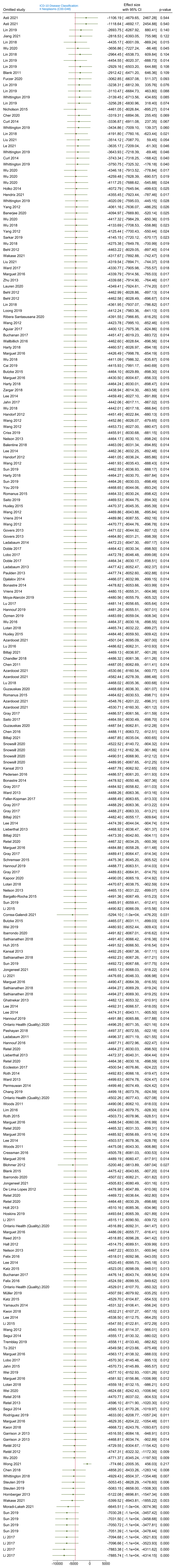

Random-effects DerSimonian–Laird model
Sorted by: inmb

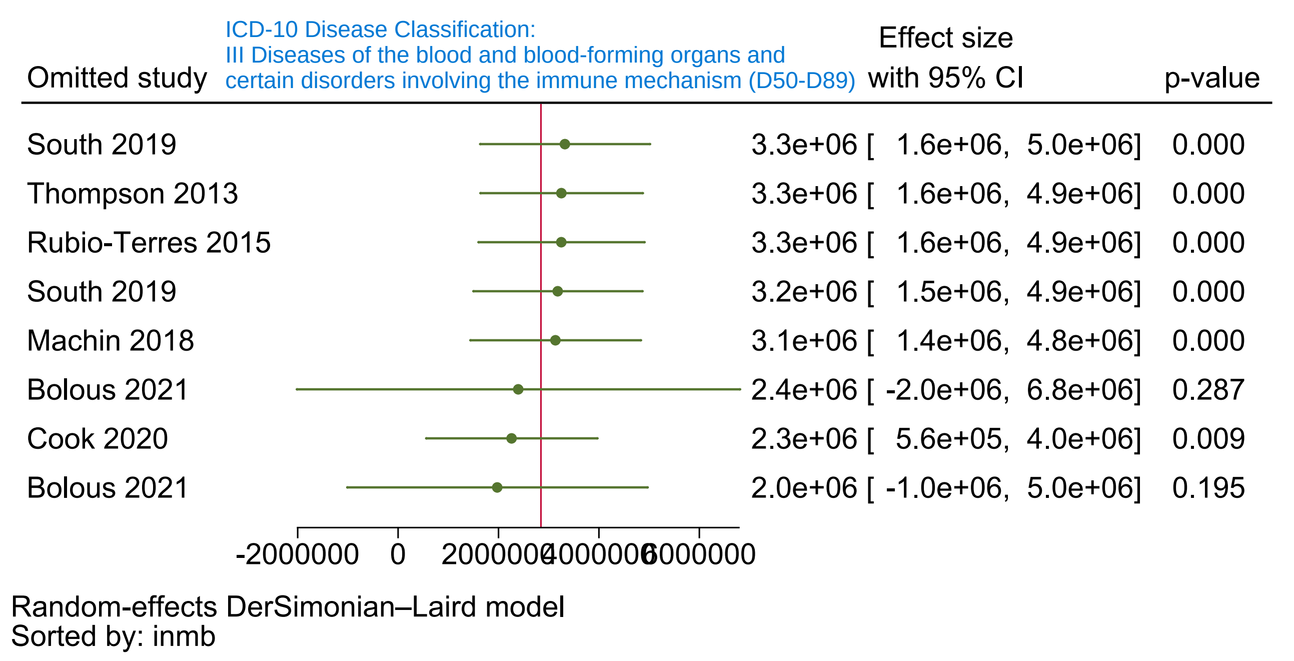
Omitted study
ICD-10 Disease Classification:
III Diseases of the blood and blood-forming organs and
certain disorders involving the immune mechanism (D50-D89)
Effect size
with 95% CI
p-value
South 2019
3.3e+06 [1.6e+06, 5.0e+06] 0.000
Thompson 2013
3.3e+06 [1.6e+06, 4.9e+06] 0.000
Rubio-Terres 2015
3.3e+06 [1.6e+06, 4.9e+06] 0.000
South 2019
3.2e+06 [1.5e+06, 4.9e+06] 0.000
Machin 2018
3.1e+06 [1.4e+06, 4.8e+06] 0.000
Bolous 2021
2.4e+06 [-2.0e+06, 6.8e+06] 0.287
Cook 2020
2.3e+06 [5.6e+05, 4.0e+06] 0.009
Bolous 2021
2.0e+06 [-1.0e+06, 5.0e+06] 0.195
-2000000
0
2000000
4000000
6000000
Random-effects DerSimonian–Laird model
Sorted by: inmb

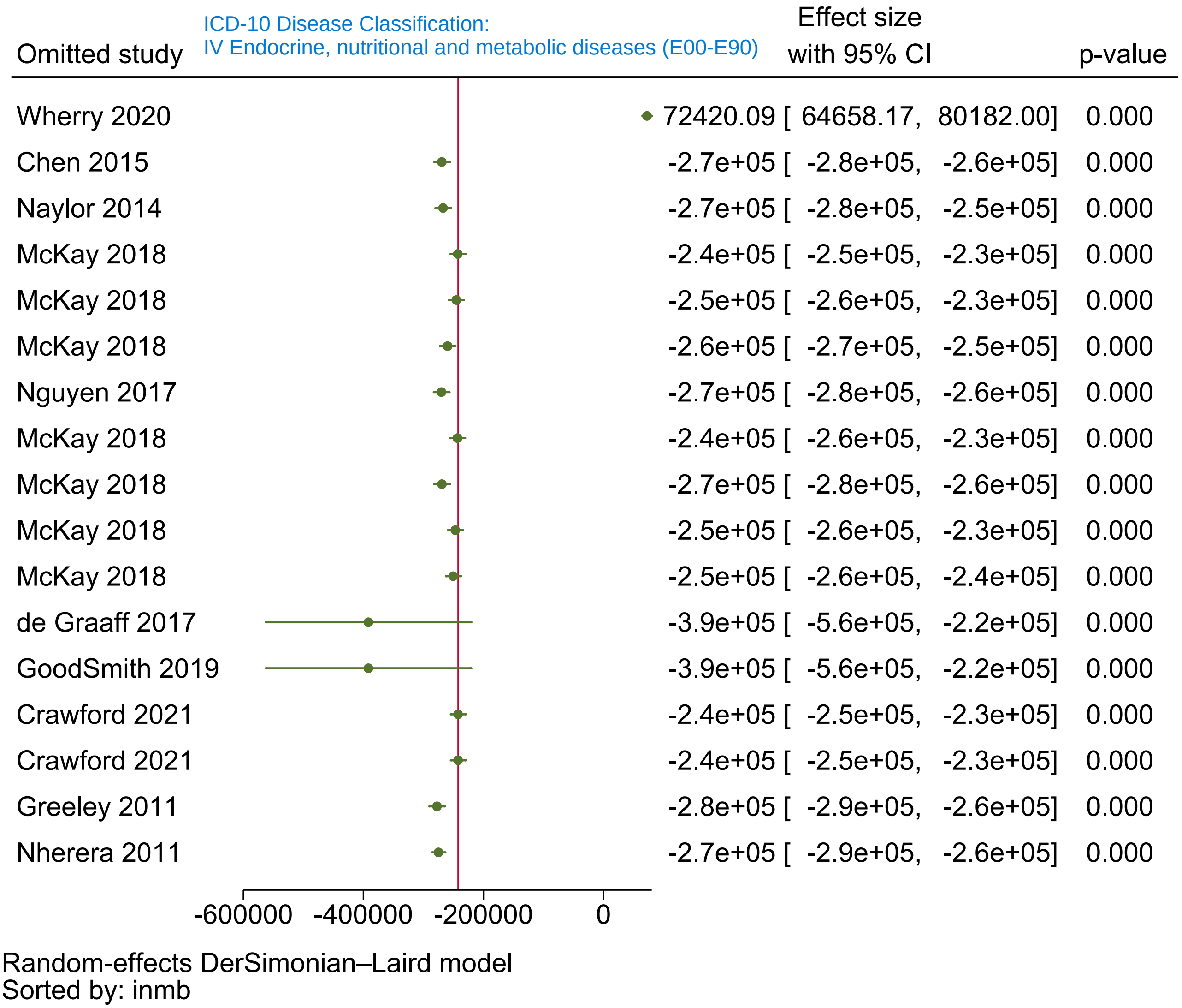
Omitted study
ICD-10 Disease Classification:
IV Endocrine, nutritional and metabolic diseases (E00-E90)
Effect size with 95% CI
p-value
Wherry 2020 72420.09 [64658.17, 80182.00] 0.000
Chen 2015 -2.7e+05 [-2.8e+05, -2.6e+05] 0.000
Naylor 2014 -2.7e+05 [-2.8e+05, -2.5e+05] 0.000
McKay 2018 -2.4e+05 [-2.5e+05, -2.3e+05] 0.000
McKay 2018 -2.5e+05 [-2.6e+05, -2.3e+05] 0.000
McKay 2018 -2.6e+05 [-2.7e+05, -2.5e+05] 0.000
Nguyen 2017 -2.7e+05 [-2.8e+05, -2.6e+05] 0.000
McKay 2018 -2.4e+05 [-2.6e+05, -2.3e+05] 0.000
McKay 2018 -2.7e+05 [-2.8e+05, -2.6e+05] 0.000
McKay 2018 -2.5e+05 [-2.6e+05, -2.3e+05] 0.000
McKay 2018 -2.5e+05 [-2.6e+05, -2.4e+05] 0.000
de Graaff 2017 -3.9e+05 [-5.6e+05, -2.2e+05] 0.000
GoodSmith 2019 -3.9e+05 [-5.6e+05, -2.2e+05] 0.000
Crawford 2021 -2.4e+05 [-2.5e+05, -2.3e+05] 0.000
Crawford 2021 -2.4e+05 [-2.5e+05, -2.3e+05] 0.000
Greeley 2011 -2.8e+05 [-2.9e+05, -2.6e+05] 0.000
Nherera 2011 -2.7e+05 [-2.9e+05, -2.6e+05] 0.000
-600000
-400000
-200000
0
Random-effects DerSimonian–Laird model
Sorted by: inmb

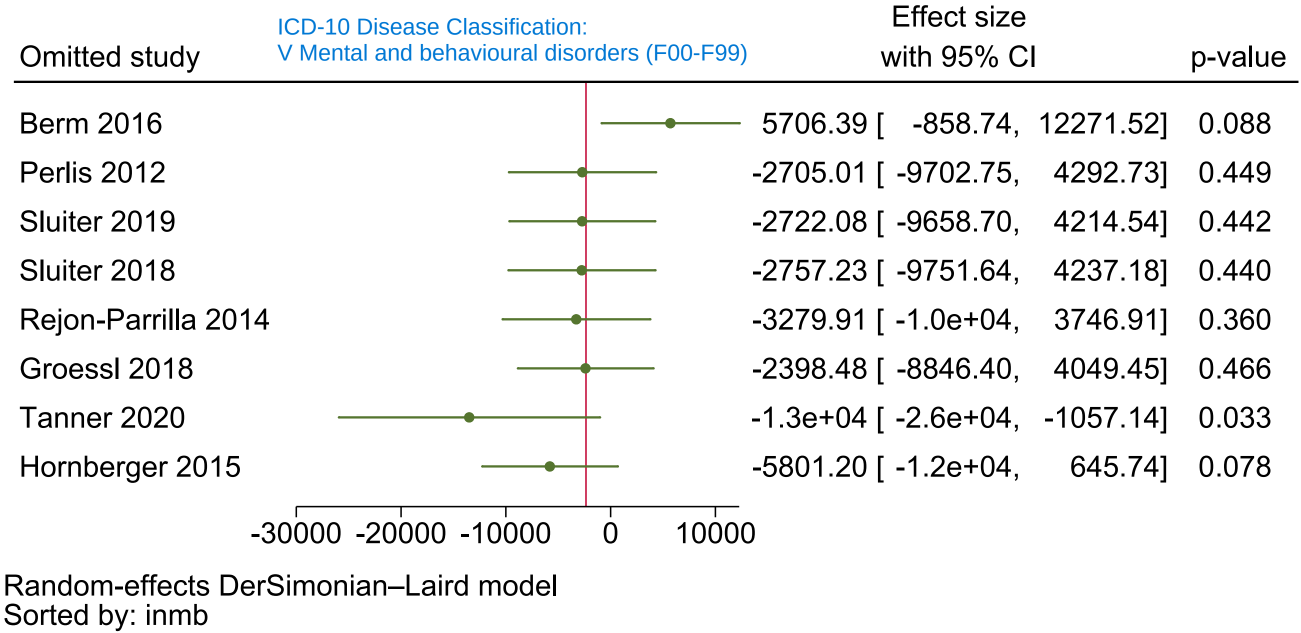
Omitted study
ICD-10 Disease Classification:
V Mental and behavioural disorders (F00-F99)
Effect size
with 95% CI
p-value
Berm 2016
5706.39 [-858.74, 12271.52] 0.088
Perlis 2012
-2705.01 [-9702.75, 4292.73] 0.449
Sluiter 2019
-2722.08 [-9658.70, 4214.54] 0.442
Sluiter 2018
-2757.23 [-9751.64, 4237.18] 0.440
Rejon-Parrilla 2014
-3279.91 [-1.0e+04, 3746.91] 0.360
Groessl 2018
-2398.48 [-8846.40, 4049.45] 0.466
Tanner 2020
-1.3e+04 [-2.6e+04, -1057.14] 0.033
Hornberger 2015
-5801.20 [-1.2e+04, 645.74] 0.078
-30000
-20000
-10000
0
10000
Random-effects DerSimonian–Laird model
Sorted by: inmb

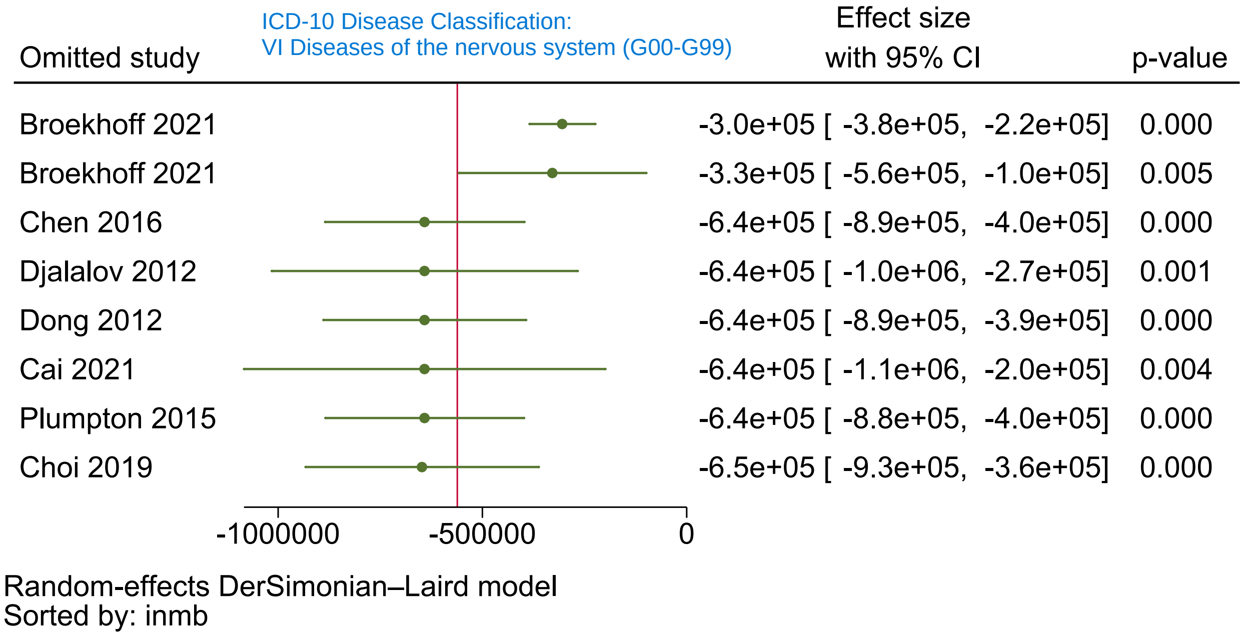
Omitted study
ICD-10 Disease Classification:
VI Diseases of the nervous system (G00-G99)
Effect size
with 95% CI
p-value
Broekhoff 2021
-3.0e+05 [-3.8e+05, -2.2e+05]
0.000
Broekhoff 2021
-3.3e+05 [-5.6e+05, -1.0e+05]
0.005
Chen 2016
-6.4e+05 [-8.9e+05, -4.0e+05]
0.000
Djalalov 2012
-6.4e+05 [-1.0e+06, -2.7e+05]
0.001
Dong 2012
-6.4e+05 [-8.9e+05, -3.9e+05]
0.000
Cai 2021
-6.4e+05 [-1.1e+06, -2.0e+05]
0.004
Plumpton 2015
-6.4e+05 [-8.8e+05, -4.0e+05]
0.000
Choi 2019
-6.5e+05 [-9.3e+05, -3.6e+05]
0.000
-1000000
-500000
0
Random-effects DerSimonian–Laird model
Sorted by: inmb

ICD-10 Disease Classification:
VII Diseases of the eye and adnexa (H00-H59)

Omitted study	Effect size with 95% CI	p-value
Uhrmann 2020	1.4e+05 [-1.3e+06, 1.6e+06]	0.844
Zimmermann 2018	-1749.48 [-1.3e+06, 1.3e+06]	0.998
Viriato 2020	55723.46 [-1.5e+06, 1.6e+06]	0.945
Johnson 2019	-5.9e+05 [-7.8e+05, -4.0e+05]	0.000

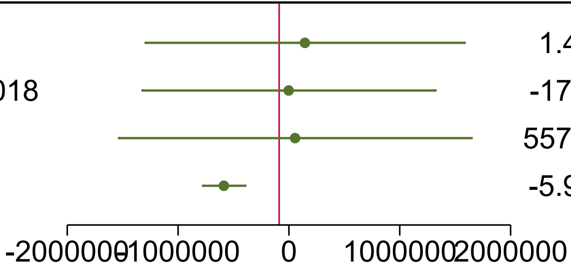

Random-effects DerSimonian–Laird model
Sorted by: inmb

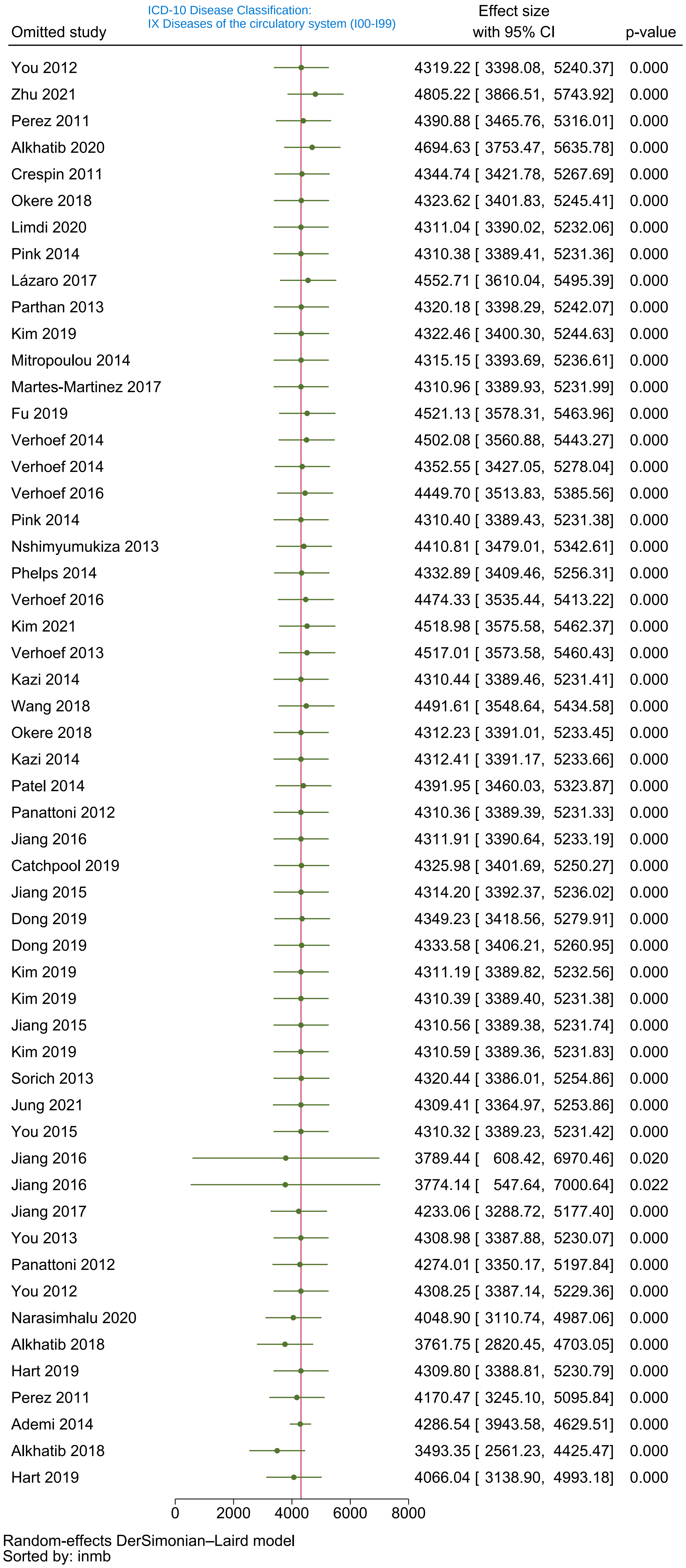
ICD-10 Disease Classification:
IX Diseases of the circulatory system (I00-I99)
Omitted study
Effect size with 95% CI
p-value
You 2012 4319.22 [3398.08, 5240.37] 0.000
Zhu 2021 4805.22 [3866.51, 5743.92] 0.000
Perez 2011 4390.88 [3465.76, 5316.01] 0.000
Alkhatib 2020 4694.63 [3753.47, 5635.78] 0.000
Crespin 2011 4344.74 [3421.78, 5267.69] 0.000
Okere 2018 4323.62 [3401.83, 5245.41] 0.000
Limdi 2020 4311.04 [3390.02, 5232.06] 0.000
Pink 2014 4310.38 [3389.41, 5231.36] 0.000
Lázaro 2017 4552.71 [3610.04, 5495.39] 0.000
Parthan 2013 4320.18 [3398.29, 5242.07] 0.000
Kim 2019 4322.46 [3400.30, 5244.63] 0.000
Mitropoulou 2014 4315.15 [3393.69, 5236.61] 0.000
Martes-Martinez 2017 4310.96 [3389.93, 5231.99] 0.000
Fu 2019 4521.13 [3578.31, 5463.96] 0.000
Verhoef 2014 4502.08 [3560.88, 5443.27] 0.000
Verhoef 2014 4352.55 [3427.05, 5278.04] 0.000
Verhoef 2016 4449.70 [3513.83, 5385.56] 0.000
Pink 2014 4310.40 [3389.43, 5231.38] 0.000
Nshimyumukiza 2013 4410.81 [3479.01, 5342.61] 0.000
Phelps 2014 4332.89 [3409.46, 5256.31] 0.000
Verhoef 2016 4474.33 [3535.44, 5413.22] 0.000
Kim 2021 4518.98 [3575.58, 5462.37] 0.000
Verhoef 2013 4517.01 [3573.58, 5460.43] 0.000
Kazi 2014 4310.44 [3389.46, 5231.41] 0.000
Wang 2018 4491.61 [3548.64, 5434.58] 0.000
Okere 2018 4312.23 [3391.01, 5233.45] 0.000
Kazi 2014 4312.41 [3391.17, 5233.66] 0.000
Patel 2014 4391.95 [3460.03, 5323.87] 0.000
Panattoni 2012 4310.36 [3389.39, 5231.33] 0.000
Jiang 2016 4311.91 [3390.64, 5233.19] 0.000
Catchpool 2019 4325.98 [3401.69, 5250.27] 0.000
Jiang 2015 4314.20 [3392.37, 5236.02] 0.000
Dong 2019 4349.23 [3418.56, 5279.91] 0.000
Dong 2019 4333.58 [3406.21, 5260.95] 0.000
Kim 2019 4311.19 [3389.82, 5232.56] 0.000
Kim 2019 4310.39 [3389.40, 5231.38] 0.000
Jiang 2015 4310.56 [3389.38, 5231.74] 0.000
Kim 2019 4310.59 [3389.36, 5231.83] 0.000
Sorich 2013 4320.44 [3386.01, 5254.86] 0.000
Jung 2021 4309.41 [3364.97, 5253.86] 0.000
You 2015 4310.32 [3389.23, 5231.42] 0.000
Jiang 2016 3789.44 [608.42, 6970.46] 0.020
Jiang 2016 3774.14 [547.64, 7000.64] 0.022
Jiang 2017 4233.06 [3288.72, 5177.40] 0.000
You 2013 4308.98 [3387.88, 5230.07] 0.000
Panattoni 2012 4274.01 [3350.17, 5197.84] 0.000
You 2012 4308.25 [3387.14, 5229.36] 0.000
Narasimhalu 2020 4048.90 [3110.74, 4987.06] 0.000
Alkhatib 2018 3761.75 [2820.45, 4703.05] 0.000
Hart 2019 4309.80 [3388.81, 5230.79] 0.000
Perez 2011 4170.47 [3245.10, 5095.84] 0.000
Ademi 2014 4286.54 [3943.58, 4629.51] 0.000
Alkhatib 2018 3493.35 [2561.23, 4425.47] 0.000
Hart 2019 4066.04 [3138.90, 4993.18] 0.000
0
2000
4000
6000
8000
Random-effects DerSimonian–Laird model
Sorted by: inmb

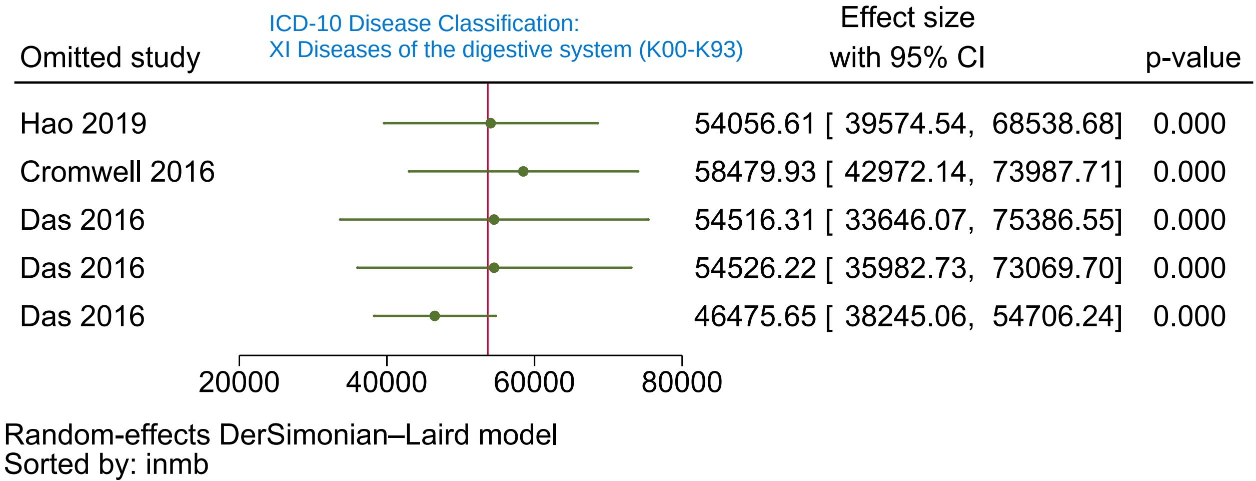
Omitted study
ICD-10 Disease Classification:
XI Diseases of the digestive system (K00-K93)
Effect size
with 95% CI
p-value
Hao 2019
54056.61 [39574.54, 68538.68]
0.000
Cromwell 2016
58479.93 [42972.14, 73987.71]
0.000
Das 2016
54516.31 [33646.07, 75386.55]
0.000
Das 2016
54526.22 [35982.73, 73069.70]
0.000
Das 2016
46475.65 [38245.06, 54706.24]
0.000
20000
40000
60000
80000
Random-effects DerSimonian–Laird model
Sorted by: inmb

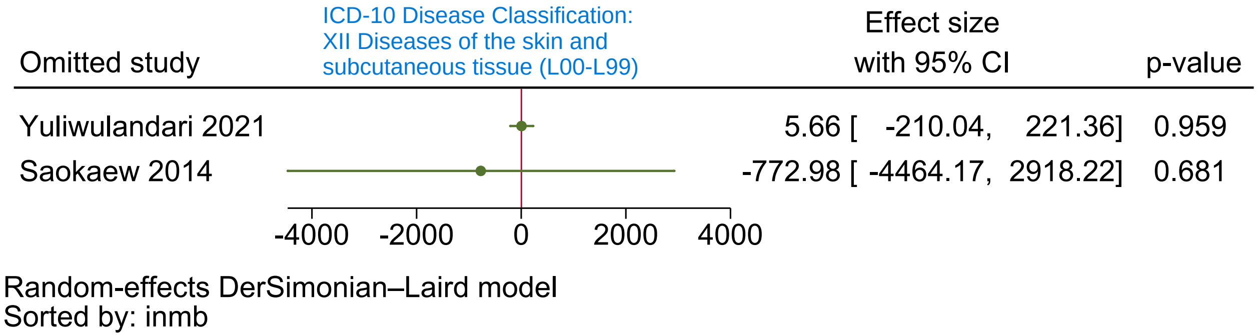
ICD-10 Disease Classification:
XII Diseases of the skin and
subcutaneous tissue (L00-L99)
Omitted study
Effect size
with 95% CI
p-value
Yuliwulandari 2021
5.66 [-210.04, 221.36]
0.959
Saokaew 2014
-772.98 [-4464.17, 2918.22]
0.681
-4000
-2000
0
2000
4000
Random-effects DerSimonian–Laird model
Sorted by: inmb

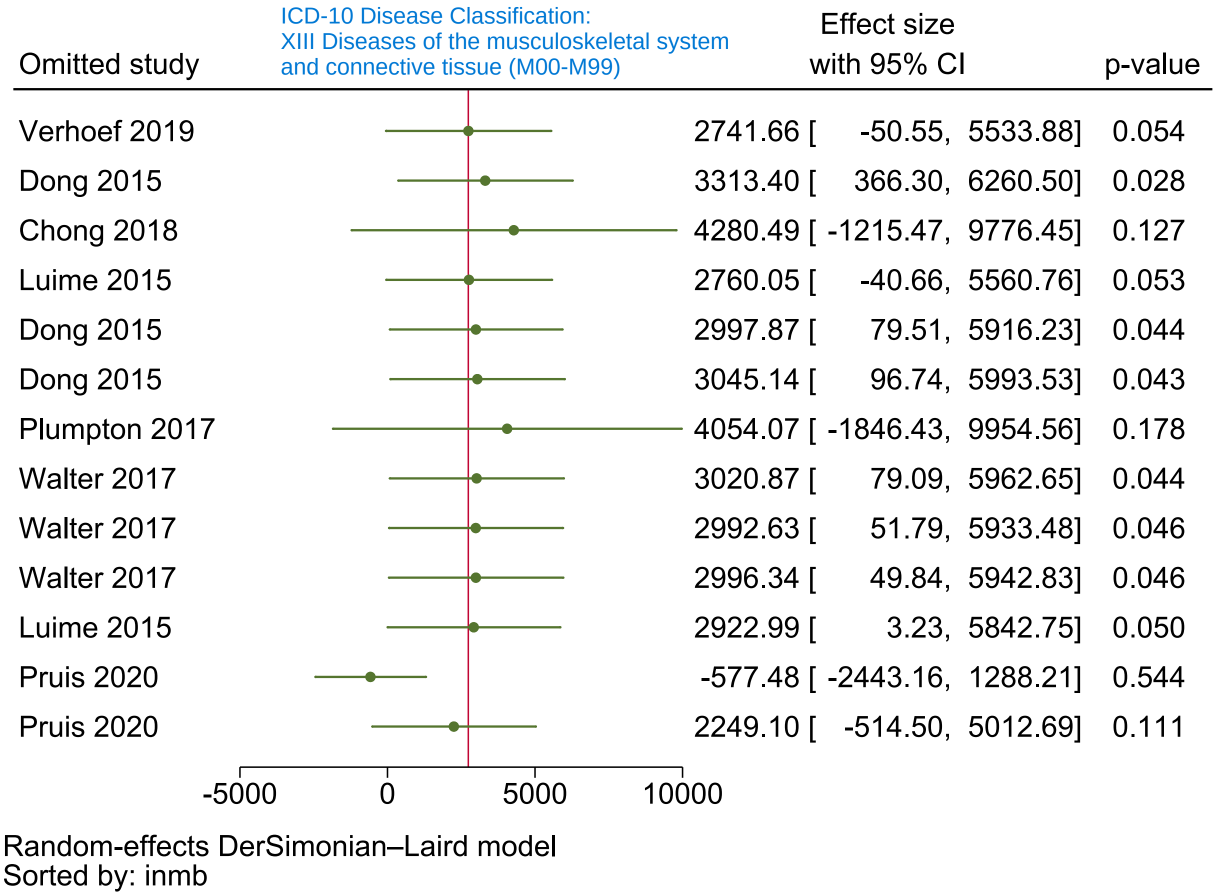
Omitted study
ICD-10 Disease Classification:
XIII Diseases of the musculoskeletal system
and connective tissue (M00-M99)
Effect size
with 95% CI
p-value
Verhoef 2019
2741.66 [-50.55, 5533.88]
0.054
Dong 2015
3313.40 [366.30, 6260.50]
0.028
Chong 2018
4280.49 [-1215.47, 9776.45]
0.127
Luime 2015
2760.05 [-40.66, 5560.76]
0.053
Dong 2015
2997.87 [79.51, 5916.23]
0.044
Dong 2015
3045.14 [96.74, 5993.53]
0.043
Plumpton 2017
4054.07 [-1846.43, 9954.56]
0.178
Walter 2017
3020.87 [79.09, 5962.65]
0.044
Walter 2017
2992.63 [51.79, 5933.48]
0.046
Walter 2017
2996.34 [49.84, 5942.83]
0.046
Luime 2015
2922.99 [3.23, 5842.75]
0.050
Pruis 2020
-577.48 [-2443.16, 1288.21]
0.544
Pruis 2020
2249.10 [-514.50, 5012.69]
0.111
-5000
0
5000
10000
Random-effects DerSimonian–Laird model
Sorted by: inmb

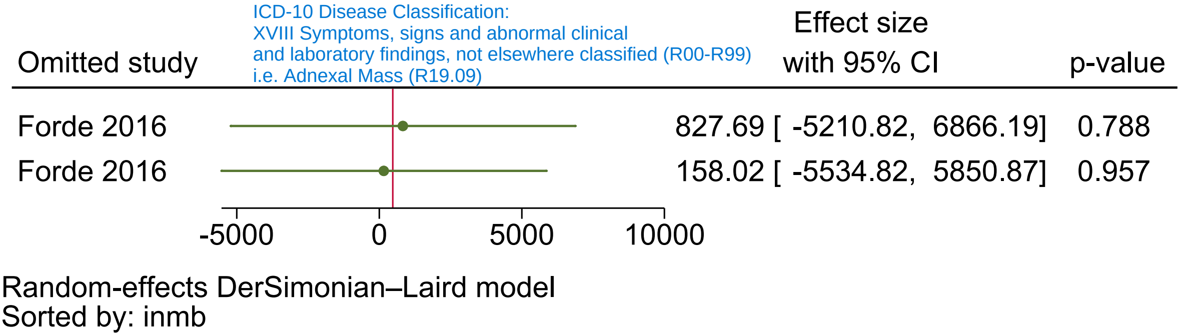

ICD-10 Disease Classification:
XVIII Symptoms, signs and abnormal clinical
and laboratory findings, not elsewhere classified (R00-R99)
i.e. Adnexal Mass (R19.09)
Omitted study
Effect size
with 95% CI
p-value
Forde 2016
827.69 [-5210.82, 6866.19]
0.788
Forde 2016
158.02 [-5534.82, 5850.87]
0.957
-5000
0
5000
10000
Random-effects DerSimonian–Laird model
Sorted by: inmb

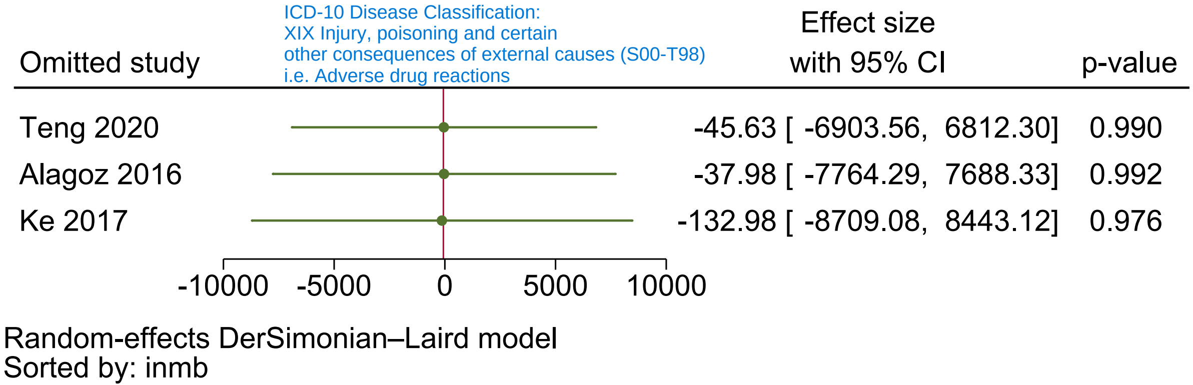
ICD-10 Disease Classification:
XIX Injury, poisoning and certain
other consequences of external causes (S00-T98)
i.e. Adverse drug reactions
Omitted study
Effect size
with 95% CI
p-value
Teng 2020
-45.63 [-6903.56, 6812.30]
0.990
Alagoz 2016
-37.98 [-7764.29, 7688.33]
0.992
Ke 2017
-132.98 [-8709.08, 8443.12]
0.976
-10000
-5000
0
5000
10000
Random-effects DerSimonian–Laird model
Sorted by: inmb

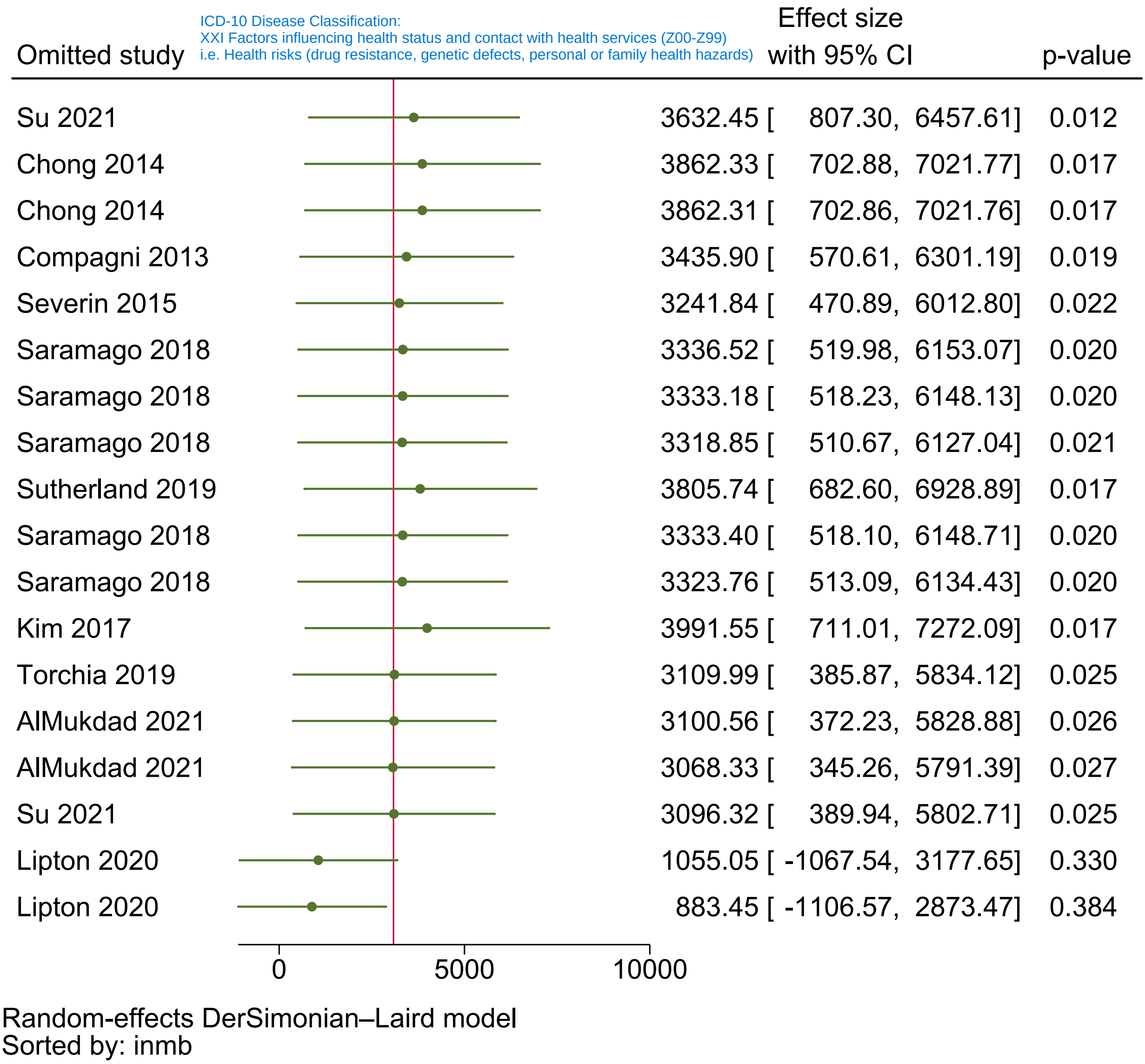
Omitted study
ICD-10 Disease Classification:
XXI Factors influencing health status and contact with health services (Z00-Z99)
i.e. Health risks (drug resistance, genetic defects, personal or family health hazards)
Effect size with 95% CI
p-value
Su 2021 3632.45 [807.30, 6457.61] 0.012
Chong 2014 3862.33 [702.88, 7021.77] 0.017
Chong 2014 3862.31 [702.86, 7021.76] 0.017
Compagni 2013 3435.90 [570.61, 6301.19] 0.019
Severin 2015 3241.84 [470.89, 6012.80] 0.022
Saramago 2018 3336.52 [519.98, 6153.07] 0.020
Saramago 2018 3333.18 [518.23, 6148.13] 0.020
Saramago 2018 3318.85 [510.67, 6127.04] 0.021
Sutherland 2019 3805.74 [682.60, 6928.89] 0.017
Saramago 2018 3333.40 [518.10, 6148.71] 0.020
Saramago 2018 3323.76 [513.09, 6134.43] 0.020
Kim 2017 3991.55 [711.01, 7272.09] 0.017
Torchia 2019 3109.99 [385.87, 5834.12] 0.025
AlMukdad 2021 3100.56 [372.23, 5828.88] 0.026
AlMukdad 2021 3068.33 [345.26, 5791.39] 0.027
Su 2021 3096.32 [389.94, 5802.71] 0.025
Lipton 2020 1055.05 [-1067.54, 3177.65] 0.330
Lipton 2020 883.45 [-1106.57, 2873.47] 0.384
0
5000
10000
Random-effects DerSimonian–Laird model
Sorted by: inmb
